# Impact of random outliers in auto-segmented targets on radiotherapy treatment plans for glioblastoma

**DOI:** 10.1186/s13014-022-02137-9

**Published:** 2022-10-22

**Authors:** Robert Poel, Elias Rüfenacht, Ekin Ermis, Michael Müller, Michael K. Fix, Daniel M. Aebersold, Peter Manser, Mauricio Reyes

**Affiliations:** 1 Department of Radiation Oncology, Inselspital, Bern University Hospital, University of Bern, Freiburgstrasse 18, 3010 Bern, Switzerland; 2 ARTORG Center for Biomedical Research, University of Bern, Bern, Switzerland; 3 Division of Medical Radiation Physics and Department of Radiation Oncology, Inselspital, Bern University Hospital, University of Bern, Bern, Switzerland

**Keywords:** Autosegmentation, Target definition, Dosimetry, False positives, Glioblastoma

## Abstract

**Aims:**

To save time and have more consistent contours, fully automatic segmentation of targets and organs at risk (OAR) is a valuable asset in radiotherapy. Though current deep learning (DL) based models are on par with manual contouring, they are not perfect and typical errors, as false positives, occur frequently and unpredictably. While it is possible to solve this for OARs, it is far from straightforward for target structures. In order to tackle this problem, in this study, we analyzed the occurrence and the possible dose effects of automated delineation outliers.

**Methods:**

First, a set of controlled experiments on synthetically generated outliers on the CT of a glioblastoma (GBM) patient was performed. We analyzed the dosimetric impact on outliers with different location, shape, absolute size and relative size to the main target, resulting in 61 simulated scenarios. Second, multiple segmentation models where trained on a U-Net network based on 80 training sets consisting of GBM cases with annotated gross tumor volume (GTV) and edema structures. On 20 test cases, 5 different trained models and a majority voting method were used to predict the GTV and edema. The amount of outliers on the predictions were determined, as well as their size and distance from the actual target.

**Results:**

We found that plans containing outliers result in an increased dose to healthy brain tissue. The extent of the dose effect is dependent on the relative size, location and the distance to the main targets and involved OARs. Generally, the larger the absolute outlier volume and the distance to the target the higher the potential dose effect. For 120 predicted GTV and edema structures, we found 1887 outliers. After construction of the planning treatment volume (PTV), 137 outliers remained with a mean distance to the target of 38.5 ± 5.0 mm and a mean size of 1010.8 ± 95.6 mm^3^. We also found that majority voting of DL results is capable to reduce outliers.

**Conclusions:**

This study shows that there is a severe risk of false positive outliers in current DL predictions of target structures. Additionally, these errors will have an evident detrimental impact on the dose and therefore could affect treatment outcome.

**Supplementary Information:**

The online version contains supplementary material available at 10.1186/s13014-022-02137-9.

## Introduction

In terms of automation in healthcare, auto-segmentation is an important technique that can be useful in radiology, surgery, study purposes and in particular radiation therapy (RT). In RT, contouring of target volumes and organs at risk (OARs) is daily practice. Much of the work is performed manually but to a certain extent, segmentation software are also used to support the task in suggesting the contours of larger structures. Auto-segmentation and contouring support (e.g. semi-automatic segmentation) have been around for decades. However, the implementation of these techniques is not widespread. Often the auto-segmentation lacks the desired accuracy [1–4], which results in copious manual adjustments and the loss of confidence in such techniques.

The main argument for fully automatic segmentation is that the current practice of manual contouring is very time-consuming for radiation oncology professionals [3, 5–8]. Another advantage is that auto-segmentation contours, compared to manual contouring, will be more consistent and it is hypothesized that this can improve the overall quality of RT planning [3, 9–11].

For the RT treatment of Glioblastoma (GBM, many critical structures, also called organs at risk (OAR), need to be spared from radiation. [12] Most of these structures are small and can only be distinguished on high quality magnetic resonance imaging (MRI) [13, 14]. Contouring in the brain is therefore a difficult and time-consuming process. Additionally, since most currently available auto-segmentation methods are based on CT imaging, they are incapable of distinguishing the different neural structures.

While there is often a clear definition of how to segment an OAR, there is much more debate on how the gross tumor volume (GTV) and clinical tumor volume (CTV) should be defined [15]. The main reason for this is the large variation in shape, size and location of a tumor in relation to the standard human anatomy. Additionally, the target often includes areas that are clinically suspected of being compromised by the tumor, and are not morphologically visual on imaging. Consistent target definition is furthermore hampered by the quality of the imaging and distortion of the anatomy caused by surgical resection that often takes place additional to RT [16].

The latest generation of auto-segmentation methods are based on deep learning [17]. The state of the art methods yield contour results for OARs and targets that are on par with manual contouring [18]. This means contours reside within the range of contour variation based on multiple raters [19, 20]. Still, the results are not perfect in terms of geometric similarity to the “ground truth” and there is no consensus in the judgement of contours among radiation oncology experts [21–27]. Neither are there clear guidelines on the commissioning of the auto-segmentation methods by medical physicists [28]. While most current errors in RT processes are human-made [29], the requirements for approval of software innovations in RT are high and not well suited for recent deep learning based methods [30]. In general, the community’s acceptance of artificial intelligence (AI) applications is poor [28, 31]. In healthcare, a machine is only accepted when it performs consistently better than a human [32].

A typical mistake deep learning-based auto-segmentation can make, are random outliers that can be defined as small segmented islands away from the region of the actual targeted structure. This type of error is best described by the large amount of outliers found in the summary of the Hausdorff distance results from the Brats Challenge [33] (e.g. Fig. 13 in referred publication). Such errors are relatively easy to solve for auto-segmentation of OARs, since shape, location and size priors of these structures can be modeled and incorporated in post-processing routines.

Dealing with random outliers gets more problematic for target definition. Since targets in the brain can appear in different locations and be of different sizes and shapes, infiltrate multiple tissues and even have satellite locations, its segmentation is more prone to inaccuracies than for OARs [34]. In addition, it is not easy to detect random outliers. Common scriptable rules to remove outliers from OARs are typically not valid for tumors. In a metastasized situation, it is even more difficult to determine if one is dealing with a random outlier (false positive) or there might be growing malignant tissue (true positive). Due to the described difficulties, robustness of deep learning-based target definition lags behind OAR segmentation methods. This is reflected by the fact that there are not many commercial products that offer deep learning-based tumor segmentation.

As a solution to improve implementation of auto-segmentation, there are two approaches: (1) Improving accuracy and robustness of deep learning methods. (2) Introduce post-processing techniques and/or QA measures that enable accurate and efficient use of automatic tumor segmentation. In both cases, a first step is to characterize the specific errors that might occur. The question we would like to answer in this study is how much an outlier, when undetected, will affect the dosimetry. Furthermore, we want to characterize the influence of size, shape and location of the outliers on the dose effect. Additionally, we want to identify the occurrences of outliers for a state of the art deep learning approach as well as their size and distance.

## Materials and methods

In this study, the main goal is to determine the impact of random outliers in target definition on the dose distribution of GBM RT plans. The study consists of two parts: (1) Controlled experiments on synthetically generated outliers. (2) Occurrence and dose effect of actual deep learning outliers resulting from state of the art deep learning methods.

### Controlled experiments with synthetically generated outliers

In this first part, we designed a set of controlled experiments to characterize how size, location and shape of outliers affect treatment plan quality.

From a local database containing the planning CT (3 mm slices) and MR images of de-identified GBM cases, a representative case was selected that does not have any intracranial deformation or extensive imaging artefacts. The images of the selected case were imported in the research environment of the treatment planning system (TPS) Eclipse version 15.5 (Varian Medical Systems, Inc.). For this case, a reference planning target volume (PTV) was generated according to the RTOG guidelines [35]. Additionally, 17 OAR volumes were defined according to Scoccianti et al. [13]

**Fig. 1:**
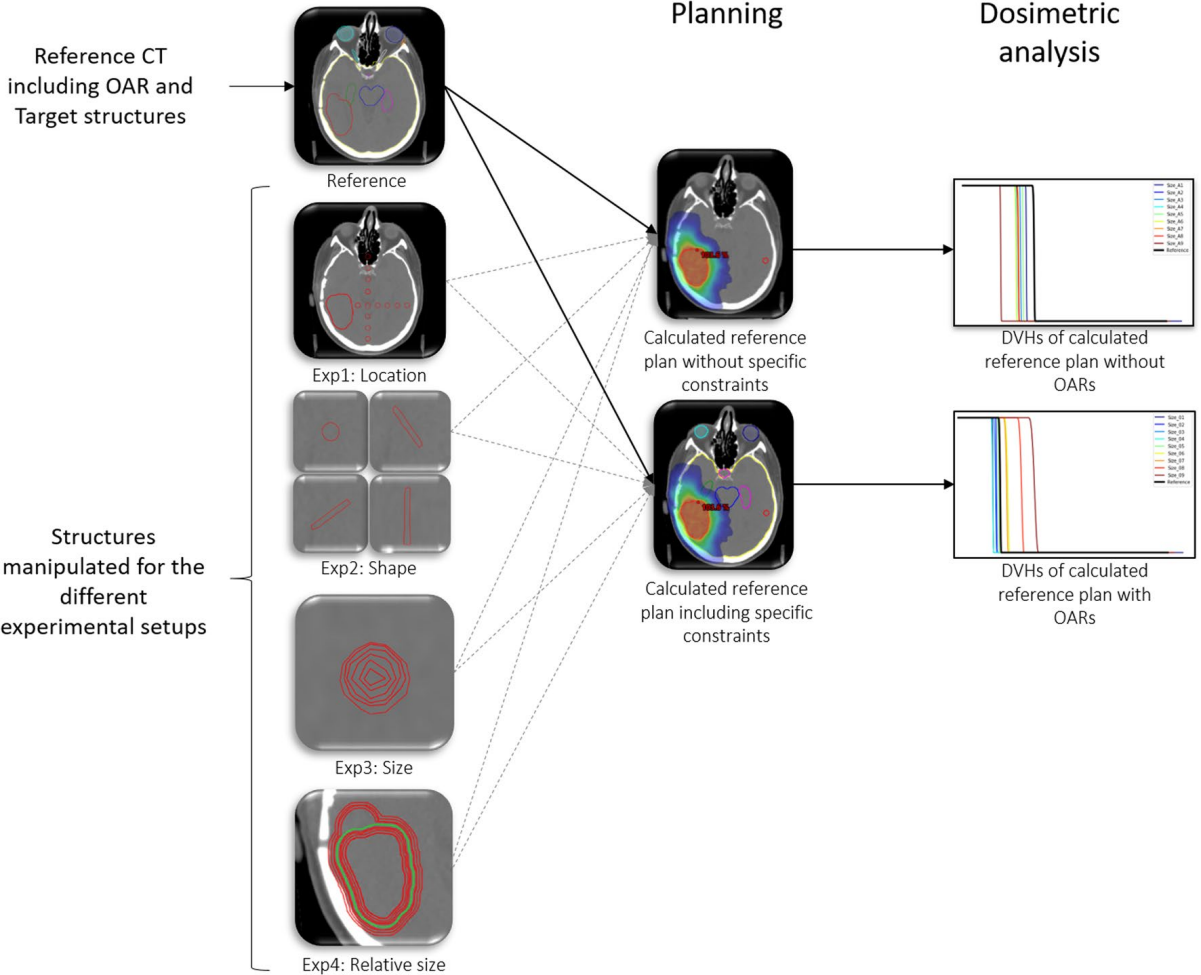
Overview schematic of the 4 experiments. On the top left corner, a planning CT of a representative GBM patient with target volumes and OAR contours is used as a reference. Based on this data, two reference plans were generated. One plan without taking the OARs into consideration during optimization and one plan where dose constraints to the OARs were set according to clinical protocol. Below the reference CT, 4 different experiments were conducted by manually drawing outliers. For each outlier in each experiment a plan including and excluding the OARs from the optimization, is generated with the same optimization objectives as the reference plans. Dosimetric analysis is performed in terms of dose volume histogram (DVH) curves and dose parameters definition. The experimental outcomes are compared amongst each other and against the reference plan

In four different experiments, the targets have been manually adjusted by adding an outlier target that was neither connected nor in the direct vicinity of the reference target but within realistic size and location boundaries. The four experiments represent: (I) different locations of the outlier, (II) different shapes of the outlier, (III) different sizes of the outlier and (IV) relative size to the main target, by changing the size of the target. In a series of planning studies, we determined the effect of random outliers on the dosimetry considering a volumetric modulated arc technique (VMAT) treatment approach (Fig. 1).

#### Experiment 1 – outlier location

**Fig. 2:**
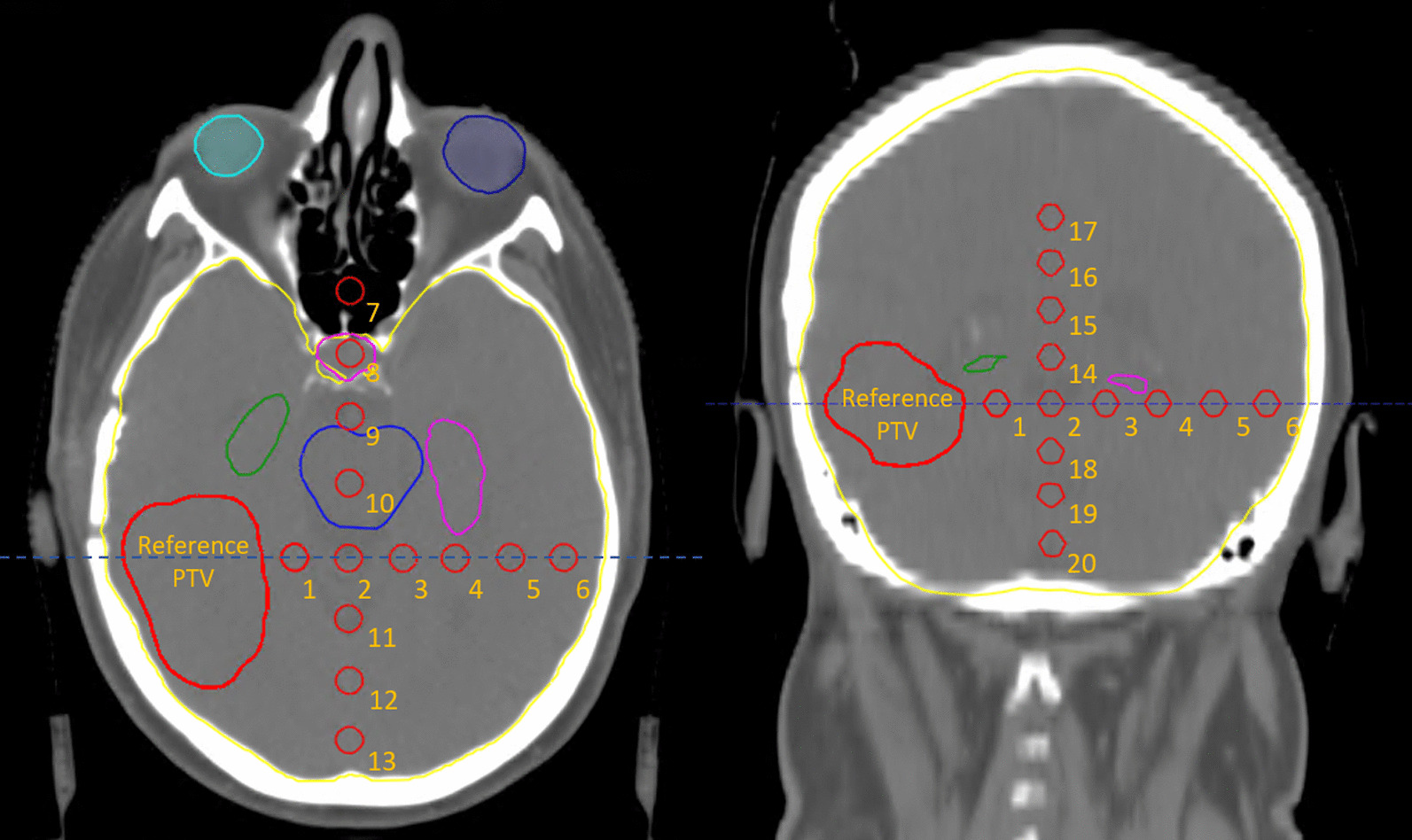
Overview of the setup of experiment 1 on location. The large red volume is the reference PTV. Along three main axes, 20 outlier contour volumes, each of size 13 mm^3^, were generated and labelled 1 to 20. The blue dashed line represents the slice location

We generated 20 small spherical outliers (0.13 cm^3^) that were added to the PTV, at different locations along the three main axes. The outliers have different distances and locations with respect to the PTV and the different OARs (Fig. 2). The goal of this experiment was to determine whether the location of an outlier, and its distance relative to the reference PTV, has a specific influence on the dosimetry.

#### Experiment 2 – shape and orientation

**Fig. 3:**
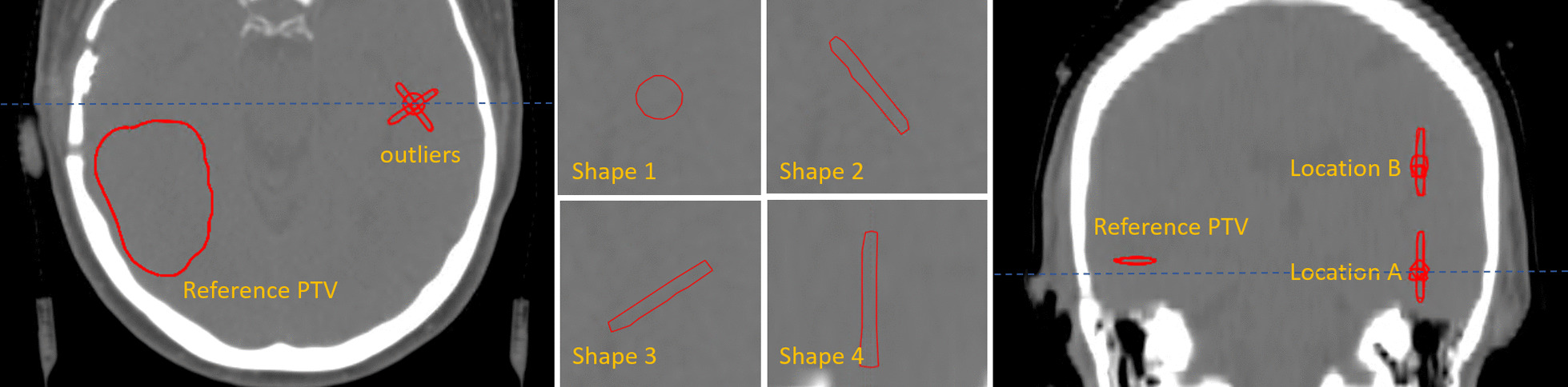
Overview of the setup of experiment 2 on shape. Left: Axial slice through the reference PTV and the caudal location **A** of the 4 different shapes of outliers. Middle: Details of the outlier shapes and orientation. It should be noted that shape 4 is positioned in the cranial-caudal direction. Right: The difference of the axial range of locations A and B can be seen. The blue dashed line represents the slice location

At a given contralateral location in the brain within the range of medium expected dose effects according to experiment 1, 4 different outliers were drawn manually over multiple slices, to have different shapes and orientation while maintaining the same volume and center of mass. The 4 outliers will appear at location A, which is at the same axial plane as the reference PTV and are additionally reproduced at location B, which is located above the axial plane of the reference PTV (Fig. 3).

#### Experiment 3 – outlier size

At two locations from experiment 1, here referred to as location C and location D, 12 different sizes of outliers were generated. The smallest outlier has a voxel volume of 4.2 mm^3^, with sizes increasing incrementally to 186.5 mm^3^. The first outliers, numbered 1 through 5 only cover a single CT slice while the latter outliers, numbered 6 through 12 cover multiple CT slices. With this experiment we aimed at analyzing the effect of outlier size on dosimetry.

**Fig. 4:**
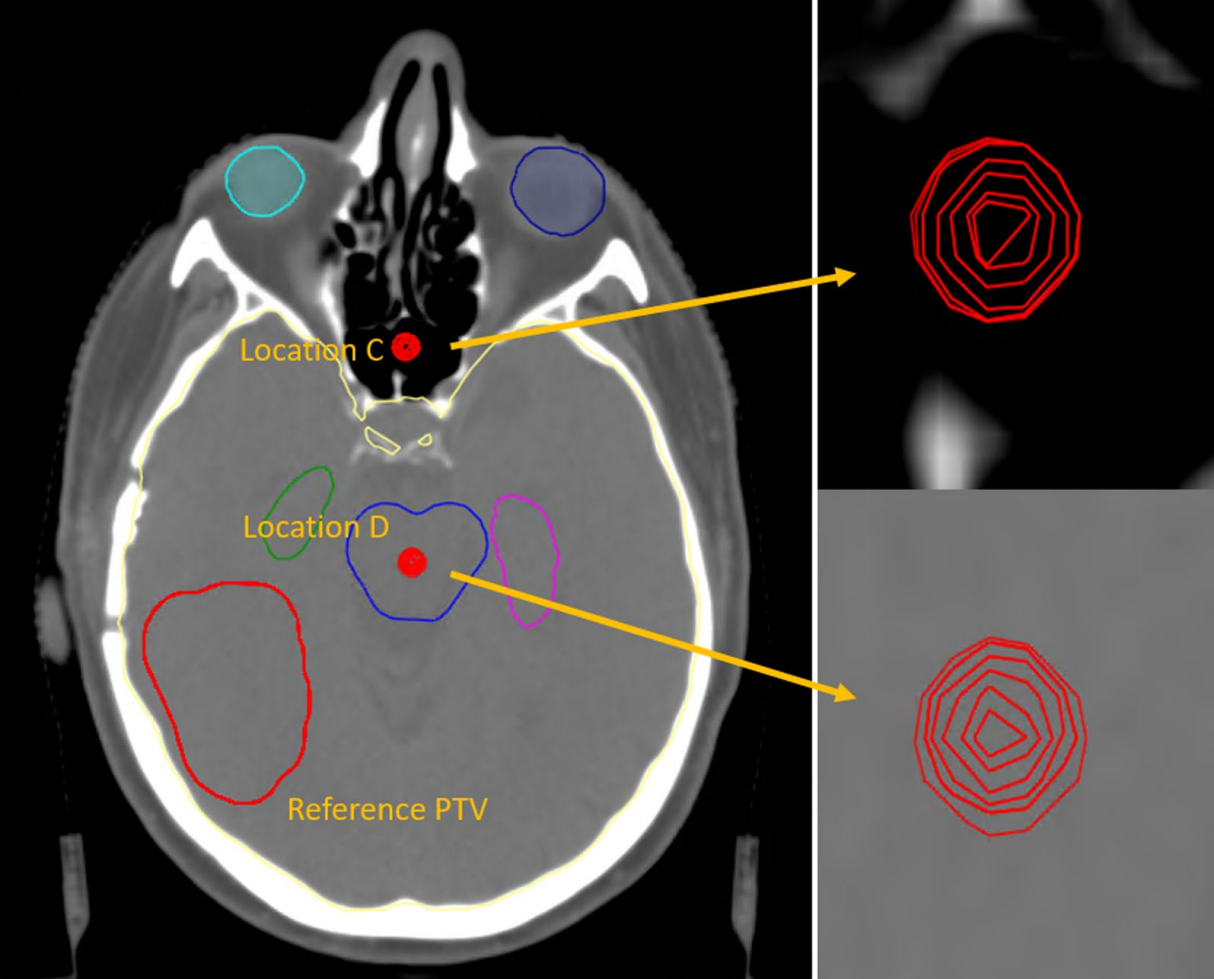
Overview of setup of experiment 3 to determine the effect of outlier size on dosimetry. In red the reference PTV and two locations, C and D of outlier volumes are represented. In both locations, 12 outliers of gradually increasing size were generated. Right: Close up view of the outlier volumes

The volumes of the different outliers were determined as voxel volume (i.e. counting the discretized amount of voxels multiplied by the voxel size) and mesh volume (i.e., geometrically from mesh points) as which is used in the TPS. (Fig. 4)

#### Experiment 4 – outliers relative size to PTV

**Fig. 5:**
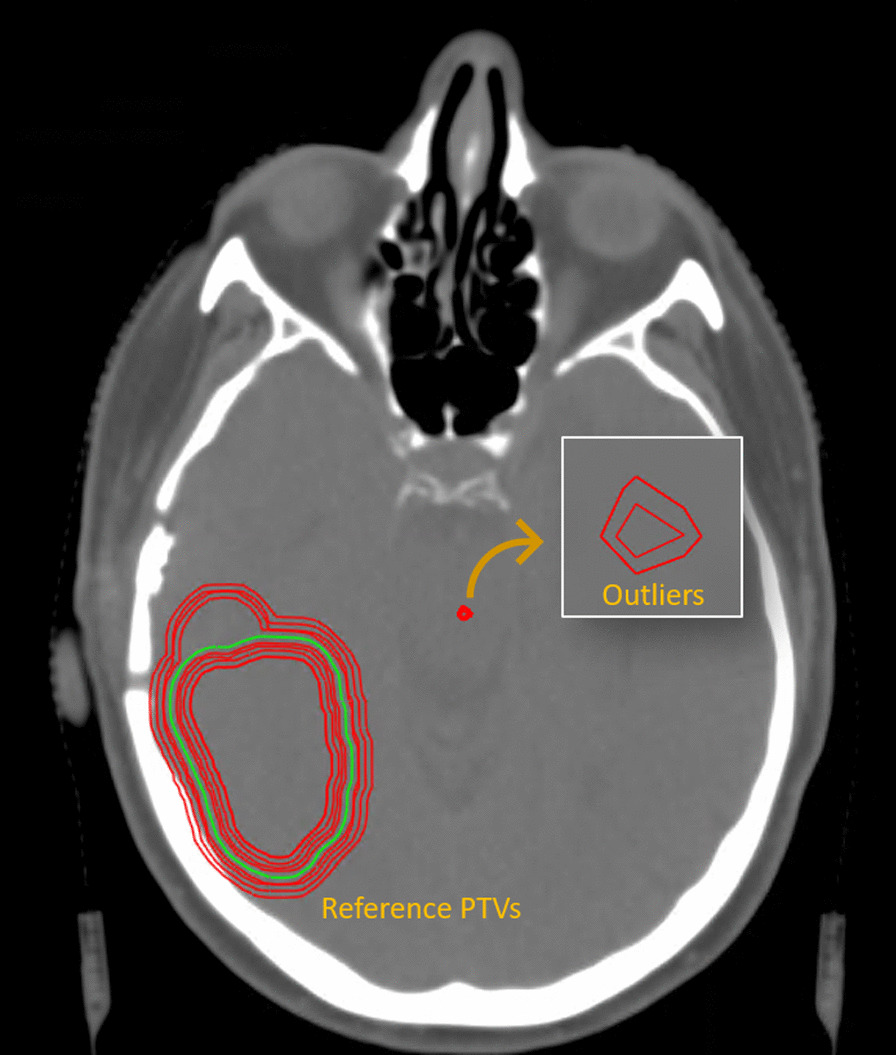
Overview of the setup of experiment 4 to determine the effect of outlier size relative to the PTV size. The smallest two outliers from location D from experiment 3 were used as outlier volumes. In this case the reference PTV (depicted in green) was increased and decreased incrementally with 1 mm margins. The yellow arrow indicates the location of the zoomed area

In experiment 3, the influence of the absolute size of the outlier is investigated. It is expected that the TPS optimizer is also influenced by the relative size of the outlier with respect to the reference PTV. To determine this we selected the two smallest outliers from location D, because this is a location that is in proximity to the target and surrounded by multiple OARs. Additionally, we respectively increased and decreased the reference PTV with increments of a 1 mm isotropic margin. This resulted in 9 different sized reference PTVs of which the original is depicted in green in Fig. 5.

For the analysis, we looked specifically at the dose received by the outlier volumes.

#### Planning

A reference plan was made based on the reference PTV and according to the institutional prescription protocol. A double arc coplanar VMAT plan with 6 MV flattening filter free beams was optimized (Varian photon optimizer version 15.6.05) to deliver 30 times 2 Gy while maximally sparing the OARs. The dose, calculated with the AAA algorithm, was normalized so that 100% of the prescribed dose covers 50% of the PTV. For the experimental plans, which include outliers as part of the PTV, the corresponding reference plan was duplicated and only the PTV structure was substituted to consider the added outliers and the adjusted size. All planning setups and optimization criteria remained the same while the plan was re-optimized once on the reference plans settings, and the dose recalculated.

An additional plan was made without any OARs, to obtain better insights in the dosimetric effects of the outliers without disturbances of any dose constraints due to nearby OARs. The objectives to the PTV were based on the prescription protocol. The only additional constraint was the normal tissue objective (NTO) of the planning system. This plan is called the PTV-only reference plan. Here too, the reference plan was duplicated and only the PTV structure was substituted to consider the added outliers and the adjusted size. All planning setups and optimization criteria remained the same while the plan was re-optimized and the dose recalculated.

#### Analysis

The dose distributions of the experimental plans were compared with those of the reference plan. To determine the differences in dose distributions, dose volume histograms (DVH) of the different structures of the experimental plans are plotted together with the corresponding DVH of the reference plan. This was performed for the PTV and all defined OARs. Additionally, the brain minus the PTV was defined to serve as a measure of the amount of dose to healthy brain tissue.

Besides the DVH curves, the following specific dose parameters were determined: For the PTV, the mean dose, minimum dose, the 95% target coverage and the 98% target coverage. For OARs, the mean dose, maximum point dose, max dose to 1% of the structures volume, and the maximum dose to one cubic centimeter of the structure.

Furthermore, the dose distributions were compared to show specific details in the effects on the dose distribution under the different performed experiments.

### Outlier target segmentations from deep learning data

Besides controlled experiments with manually constructed false positive outliers in the target volume, we constructed target data by means of a deep learning segmentation model. This data reflects outliers resulting from auto-segmentation predictions.

#### Deep learning data

As training data 100 GBM cases were available who received surgery and RT treatment at the Inselspital Bern, University Hospital, but did not have any prior brain pathologies. Of all cases, the GTV and the edema regions were annotated. From the 100 cases, 80 randomly chosen cases were used for training and the remaining 20 cases were used as test dataset. We performed a five-fold cross validation, resulting in five different models, and one ensembling model [36] based on majority voting of these five models. We included this ensembling model to verify the advantages of ensembling, as reported in [37], and whether it was able to improve GBM targets after construction of the PTV. Model training was based on the nnUnet architecture based on the work of Isensee et al. [38]. A transfer learning approach was used, with pre-training model weights based on the HD-GLIO segmentation model trained on 3220 brain tumor MRI examinations [39]. Each model was then fine-tuned on the training dataset (i.e., 80 cases per fold). Technical details of the training procedure can be found in [40, 41].

#### Outlier analysis

In order to determine the number of outliers that are created by the deep learning models, we defined the main structure as the largest connected region of calculated segmentation masks. Each other segmented region disconnected from the main structure was counted as an outlier. This assumption was valid as the dataset only include single-lesion cases. For each case and trained model (i.e., five plus majority voting), the total number of outliers (per case and per model) as well as their size and closest distance to the main target was recorded. We analyzed outliers’ size vs. their distance from the main structure since it is expected that these two parameters play a role in dosimetry metrics.

To assess the impact of deep learning-based outliers on dosimetry, for every automated segmentation, a CTV was created by combining the GTV and the edema structures and 3 mm margins were added to form the PTV according to the RTOG guidelines [35]. On the resulting PTV structures, we analyzed the distribution of outliers.

#### Dose effect from deep learning segmentations

From the total of 120 constructed PTVs from deep learning models (6 models × 20 test cases), 5 cases with an outlier of significant size and distance from the PTV were randomly selected for dosimetric analysis. For these cases a reference PTV was available, which is a manually drawn target verified by a radiation oncology expert. Based on the reference PTV, a plan was constructed according to the current clinical department’s protocol, which included constraints for all OARs. To show the impact of the outlier to the predicted target in particular, we made a copy of the predicted target with and without outlier. To ensure the geometrical similarity with respect to the reference PTV, the copy of the predicted PTV was manipulated to obtain the same Dice Similarity Coefficient (DSC) with respect to the reference PTV as the original predicted PTV has with the reference PTV. On both the predicted PTV containing the outlier (referred hereafter as “predicted outlier”) and the predicted PTV with the outlier removed (referred hereafter as “removed outlier”) the reference plan was re-optimized and recalculated. These two plans were compared to the reference plan. An analysis on the DVHs of the different OARs was then performed. Since the dose effect is highly dependent on the location of both the target and the outlier, the dose to the healthy tissue, defined as the brain minus the reference PTV, was also analyzed. As to perform a direct comparison of the total three-dimensional dose distribution, gamma analysis of the predicted outlier plan, and the removed outlier plan was performed with respect to the reference plan. For the gamma analysis we used the criterion of 3% of the prescribed dose and 3 mm and a dose cutoff at 20 Gy to remove the lower dose regions.

## Results

### Controlled experiments with synthetically generated outliers

#### Experiment 1 – outlier location

Planning on the PTV only without taking into account the OARs but with the outlier as part of the PTV at different locations did not have much effect on the target coverage. The maximum deviation in the 98% dose coverage (D98%) was 3.2% (i.e. 55.8 Gy instead of 57.6 Gy for the PTV-only reference plan).

Subsequently, we observed that the dose to healthy brain tissue (i.e., brain volume minus the reference PTV) is most fluctuating in the volume receiving a dose from 5 – 15 Gy. The volume receiving this dose range increases depending on relative location to the PTV or decrease with respect to the reference plan due to the fact that some of these outliers are located outside the brain tissue. The maximum dose to 1% of the healthy brain tissue remains stable just under 50 Gy for all locations.

**Fig. 6:**
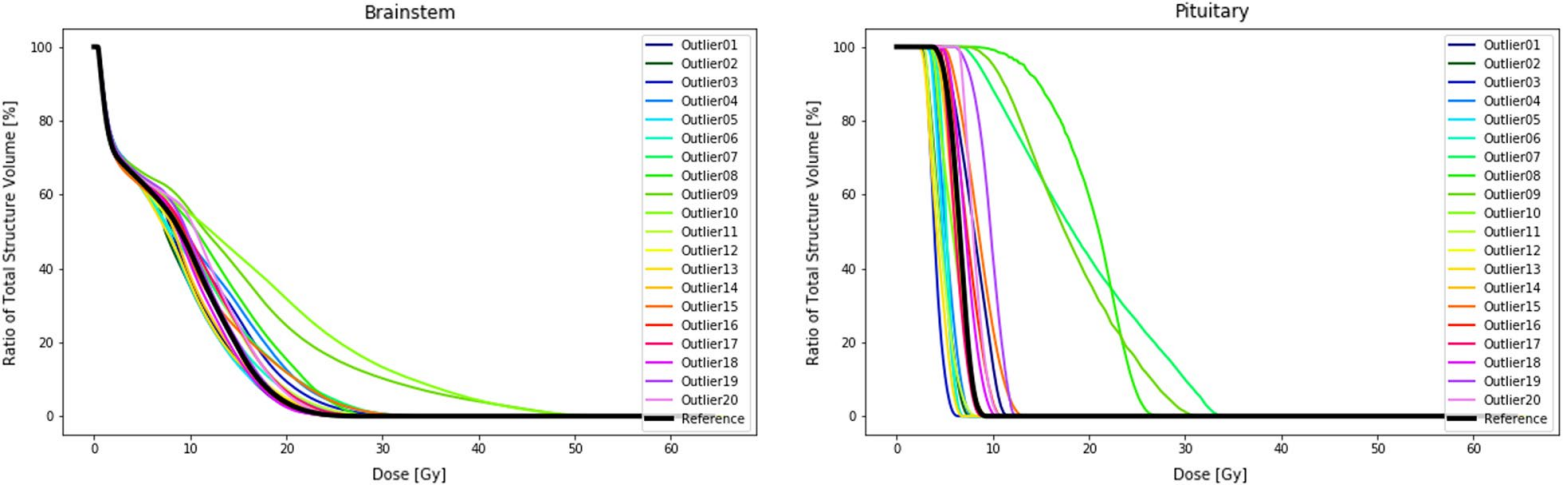
DVH curves brainstem (left) and the pituitary gland (right) of the reference plan and the 20 plans containing an outlier in the PTV at specific locations as displayed in Fig. 2. During the planning OARs have been taken into account

**Fig. 7:**
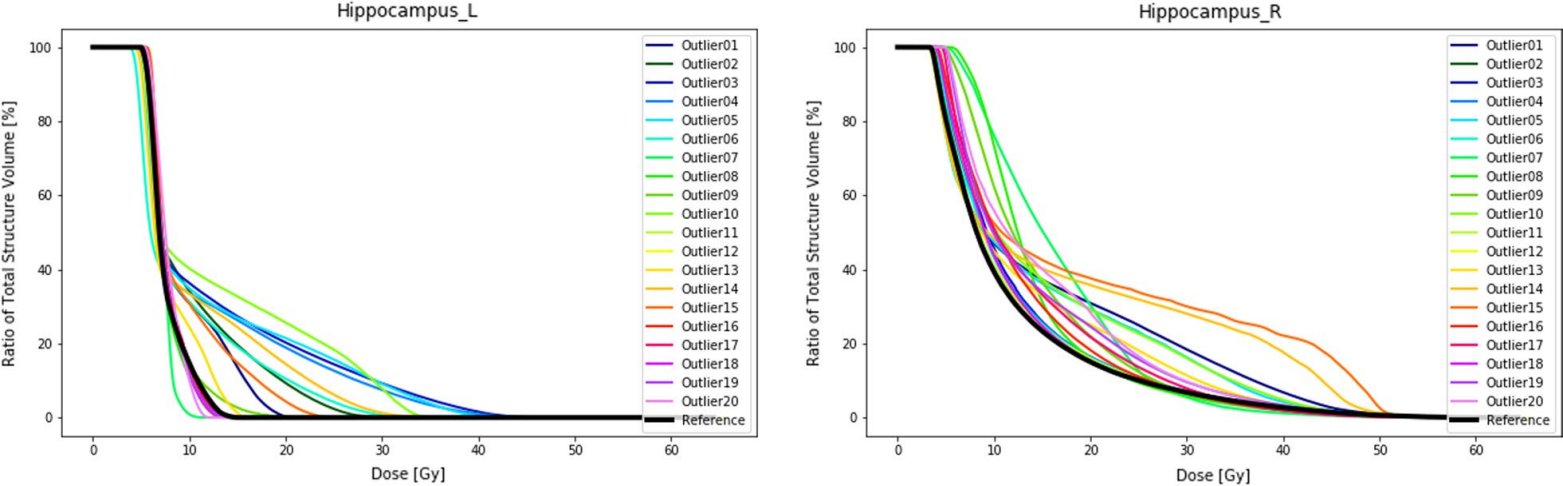
DVH curves of the left hippocampus (left) and the right hippocampus (right) for the reference plan and the 20 plans containing an outlier in the PTV at specific locations as displayed in Fig. 2. During the planning, OARs have been taken into account

When OARs are introduced during plan optimization we see some more pronounced effects. The target coverage compared to the PTV-only plans had a maximum deviation of the D98% of 4.1%. Doses to OARs are increasingly affected for outlier locations overlapping with or in close proximity to the specific OAR. Especially when an OAR is located between the reference PTV and the outlier, a typical increase to the dose received by the OAR is observed. This is shown in the DVH curves of the pituitary gland in locations 8, 9 and 10 (Fig. 6). The dosimetric effect is also highly influenced by the objectives set in the optimizer. This is well illustrated in Fig. 7. The left hippocampus, which is confined at 40% of the volume receiving 7.4 Gy, keeps doses well contained above the 40% volume but allows dose to increase freely outside the range of the constraints. For detailed overview of the results, we would like to refer to Additional file: 1.

#### Experiment 2 – shape and orientation

With regards to shape and orientation of the outlier, the four different shaped outliers do not affect target coverage when no OARs are involved. The maximum deviation of the D98% is less then 1%. The dose to the healthy brain tissue is however increased with respect to the reference plan and obviously more so at location B than at location A. Among the different shapes, we noticed that shape A2 and shape B2, corresponding to elongated shapes perpendicular to the more dominant radiation directions (see Additional file: 1), have the most influence on the dose.

**Fig. 8:**
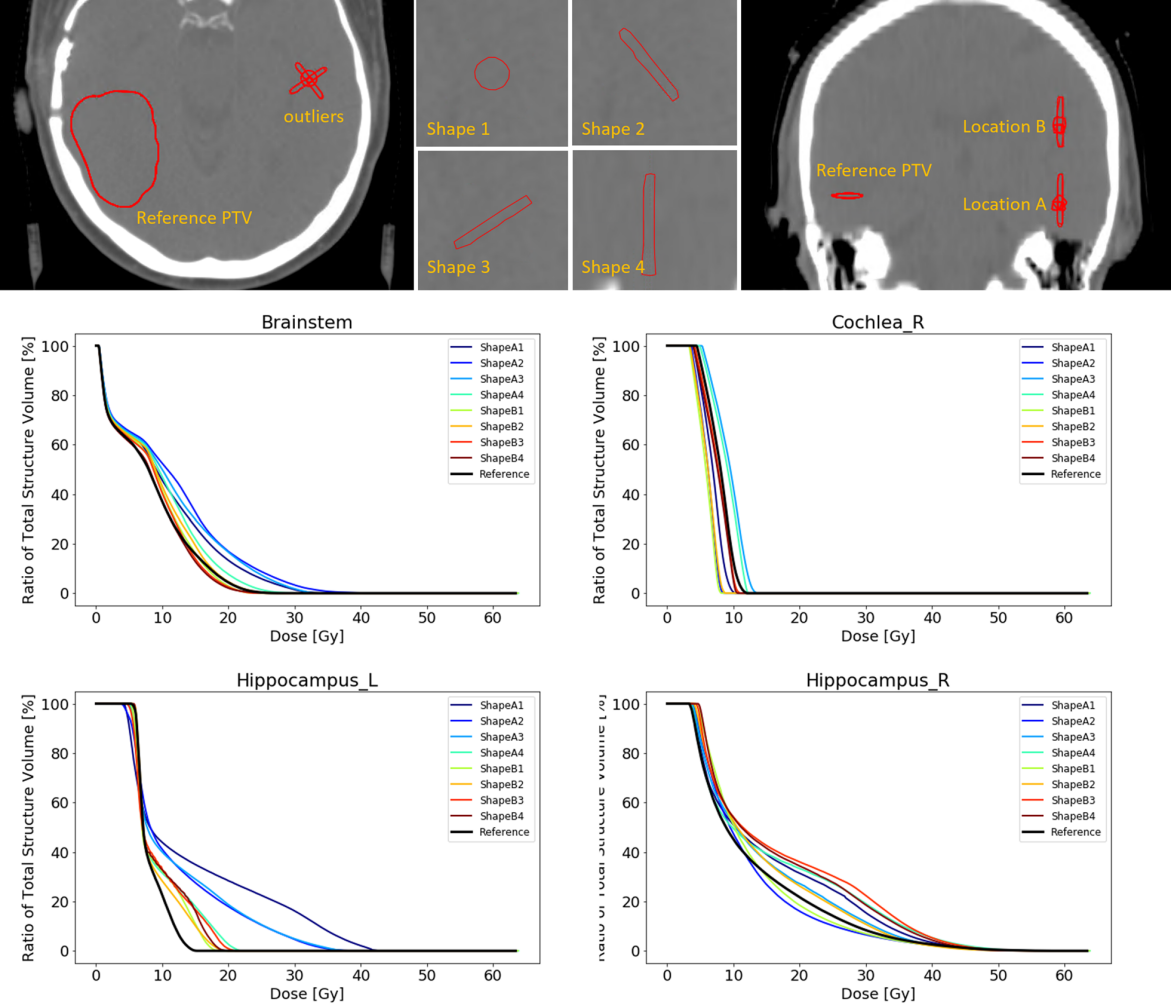
Above; schematic representation of the structures of experiment 2, equal to Fig. 3. Below; DVH curves of the brainstem (upper left), right cochlea (upper right) left hippocampus (lower left) and the right hippocampus (lower right) of the reference plan and the 8 plans containing an outlier in the PTV at specific shape and locations as displayed in the schematic above. During the planning OARs have been taken into account

Introducing OARs into the optimization process, the outliers will decrease dose coverage of the reference PTV with a maximum deviation of the D98% of 2.8%. In addition, the dose effects to the specific OARs in proximity of the target are more pronounced. The maximum dose to the brainstem is especially affected by the the outliers at location A, and most prominently so by shape 2. The left hippocampus however is most influenced by shape A1. At other locations such as the right cochlea, the dose decreases by the presence of the outliers (Fig. 8).

It becomes clear that at location B, further away from the OARs, less changes to the dose of these OARs occur. It has to be noted that elongated shape in the cranial caudal direction seems to have the least impact on the dosimetry (Fig. 8).

#### Experiment 3 – outlier size

**Fig. 9:**
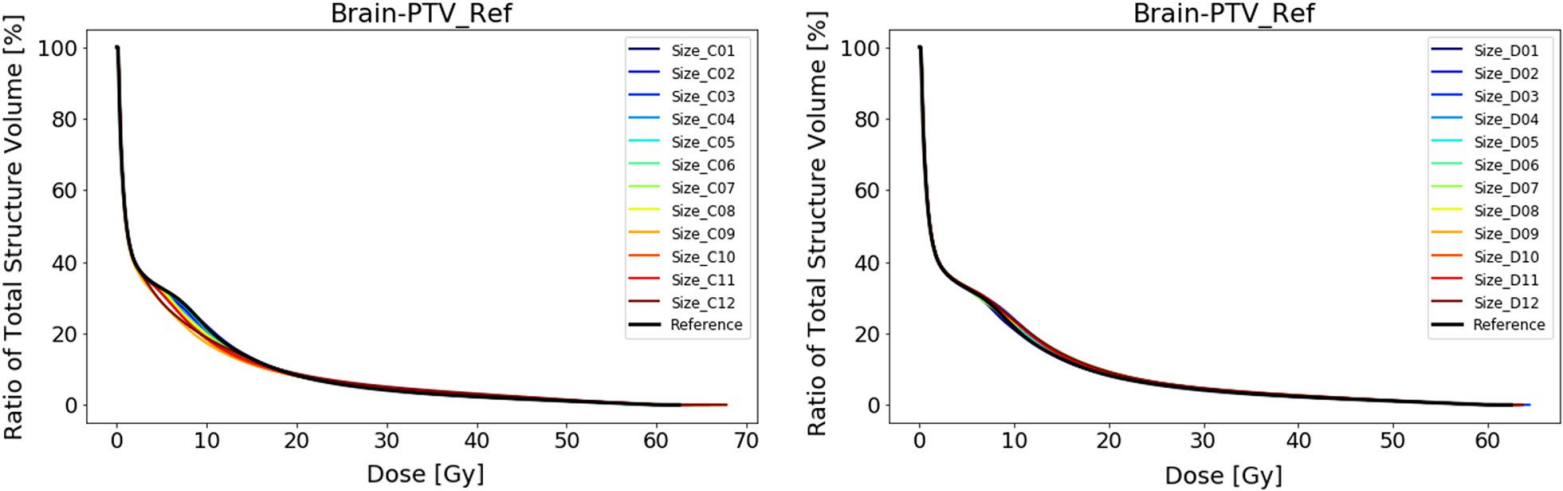
DVH curves of the healthy brain tissue (i.e. brain minus PTV) for the reference plan and the 12 plans containing an outlier in the PTV of a specific size as displayed in Fig. 2 at location A (left) and location C (right). During the planning OARs have not been taken into account

We looked at different sizes of outliers at two different locations. When no OARs are involved during optimization, the effect of the outliers on the target coverage is minimal. Maximum deviation of the D98% is less than 1%. The dose to the healthy brain tissue increases with increasing volume of the outlier. Interestingly, for location D with respect to the reference plan, we see a dose *increase* that is concentrated around the 10 to 20 Gy range, while for location C there is a clear *decrease* of the dose around the 5 to 15 Gy range (Fig. 9). This decrease is caused on the one hand because the outlier is situated outside the healthy brain tissue and hence does not contribute fully to the dose to the brain. More importantly, the location of the outlier in fact concentrates the dominant beam direction to the region between the outlier and the target. This results in a decrease in the dose bath in the regions outside this dominant beam direction. This effect is visible in the dose distribution animations provided in Additional file: 3.

Noteworthy to this experiment, when the outlier is small enough (C1 and C6, D1) the optimizer choses to ignore the outlier as part of the target altogether and no dose is directed to the outlier directly.

**Fig. 10:**
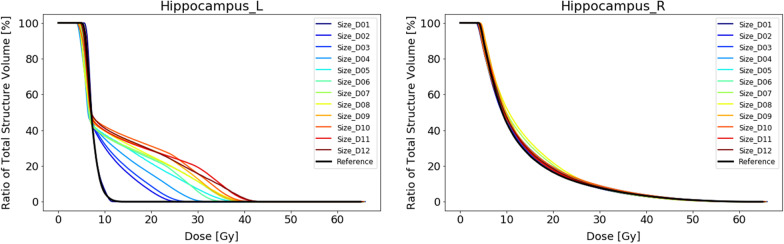
DVH curves of the left hippocampus (left) and the right hippocampus (right) for the reference plan and the 12 plans containing an outlier in the PTV at specific sizes as displayed in Fig. 4 at location D. During the planning OARs have been taken into account

Introducing OARs to the optimization does slightly affect the coverage. This is most pronounced at location C where the largest outlier shows a deviation for the D98% of 4.1% with respect to the reference plan. At location D, we see that the doses to specific OARs are affected significantly. The dose to the brainstem, where the outlier is located in, increases correlating to the size of the outlier. We see the same trend for the left hippocampus, which is located in the dominant beam direction. In contrast the right hippocampus, which is also in the vicinity of both the target and the outlier but remains outside the main beam direction, the dose effect is minimal (Fig. 10).

Similarly, when the outlier is small enough (C1, C6, D1) there is no explicit dose directed by the optimizer. However, we noticed that outliers that resided on multiple axial slices had a larger impact than outliers residing on a single slice, even when comparable in size.

**Fig. 11:**
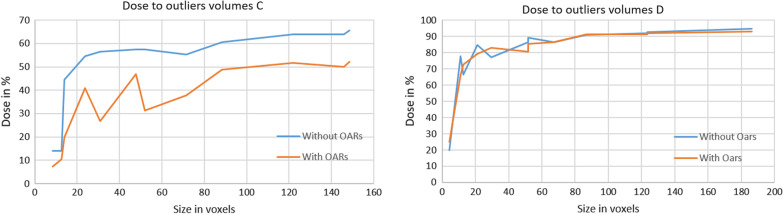
Plot graphs of the dose to the outlier volume against the size of the outlier volume in voxel size. On the left the results of the plans without OARs (blue) and the plans involving OARs (orange) are shown for location A. On the right, they are shown for location C

If we look at the amount of dose that is received by the isolated outlier regions, we see how the optimizer deposits dose to different sized outliers. In Fig. 11, we have plotted the relative dose to the outliers against the voxel sizes. Figure 11 shows that for the small sizes under 10 voxels (1 voxel = 1 × 1 × 1 mm^3^), the optimizer ignores the outliers since they are irrelevant in the cost function. There is a slight trend of increasing dose coverage with increasing volume. At location C, the outliers receive much less dose. Approximately only 60% of the prescribed dose as opposed to 80–90% of the prescribed dose for location D. The main reason is that the surrounding OARs at location C have lower constraints. The seesaw pattern can be explained by the difference between outliers covering a single axial slices (dips) and multiple axial slices (peaks). (Table 1).

**Table 1:** Volumes of outliers

Location	voxel volume (mm 3)	mesh volume (mm 3)	Difference (%)
C1	8.41	5.49	34.72
C2	14.02	10.05	28.32
C3	30.84	25.12	18.55
C4	51.87	45.09	13.07
C5	71.50	63.31	11.45
C6	12.62	9.11	27.81
C7	23.83	19.04	20.10
C8	47.66	41.65	12.61
C9	88.32	80.72	8.61
C10	121.97	113.20	7.19
C11	145.80	136.45	6.41
C12	148.60	139.02	6.45
D1	4.21	1.98	52.97
D2	12.62	8.65	31.46
D3	29.44	24.07	18.24
D4	51.87	45.09	13.07
D5	67.29	59.11	12.16
D6	11.22	7.94	29.23
D7	21.02	16.71	20.50
D8	51.87	46.15	11.03
D9	86.91	79.85	8.12
D10	123.36	114.61	7.09
D11	123.36	114.61	7.09
D12	186.45	176.17	5.51

Sizes of the outlier volumes of experiment 3. Determined by means of the voxel volume and mesh volume on the converted nifty file format. The mean difference over all locations is 17.2%

#### Experiment 4 – outliers relative size to PTV

In the last synthetic experiment, we looked at the influence of the relative size of the outlier with respect to the reference PTV by adjusting the size of the latter. We performed this with both the outlier D1 and outlier D2 from experiment 3. The sizes of the reference PTV increase incrementally with an isotropic margin of 1 mm from structure 1 to 9. The actual size ranges from 29.1 to 55.6 cm^3^. The middle sized PTV with number 5 refers to the the reference PTV from the previous experiments.

**Fig. 12:**
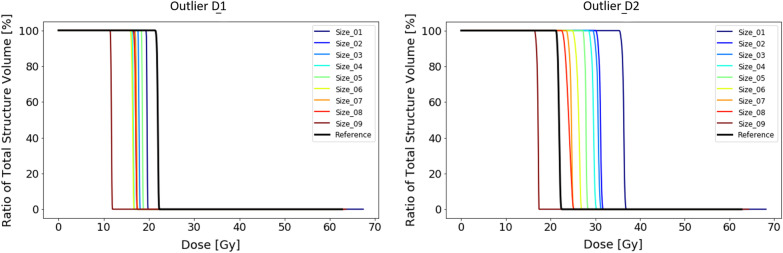
Results of the dose to the outlier volumes of experiment 4 without OARs. On the left the DVH of the reference plan and the 9 plans for the different PTV sizes are shown in combination with outlier D1 (4.2 mm^2^). On the right the DVHs for the plans in combination with outlier D2 (12.6 mm^2^) are shown

From plans made without OARs taken into account it shows that the dose received by the outlier volume D1 is in all instances smaller than the reference plan where no outlier was present (Fig. 12). This suggests that the outlier was not taken into account by the optimizer and no additional dose was directed to the outlier volume. It shows that there is a relationship between the size of the PTV and the dose received by the outlier (Fig. 12). The smallest PTV led to the highest dose in the outlier volume (19.1 Gy), while the plan on the largest PTV led to the smallest dose to the outlier volume (13.9 Gy). Overall, the dose bath changes from a more spherical shape around the main target for the smaller PTVs to a longitudinal shape for the largest PTV. This suggests that the increased size of the PTV changed the dominant beam direction. In this case it diverted the dominant beam direction away from the outlier. The changes to the dose distribution per increasing PTV size are represented by the animations in the Additional file 3.

The outlier volume D2, which was approximately 3 times the size of D1 (Table 1), was taken into account by the plan optimizer. The dose received by the outlier volume is larger for the experimental plans than for the reference plan (Fig. 12). Again, we see a relationship between the amount of dose received by the outlier volume and the size of the PTV. As expected, the larger the relative size of the outlier with respect to the total PTV the more dose will be directed to the outlier by the optimizer without changing any of the optimization criteria. At a specific size of the PTV (size 9) the optimizer chooses to ignore the outlier altogether. This is also influenced by the change in beam directions for this specific plan as seen on animations in Additional file 3.

**Fig. 13:**
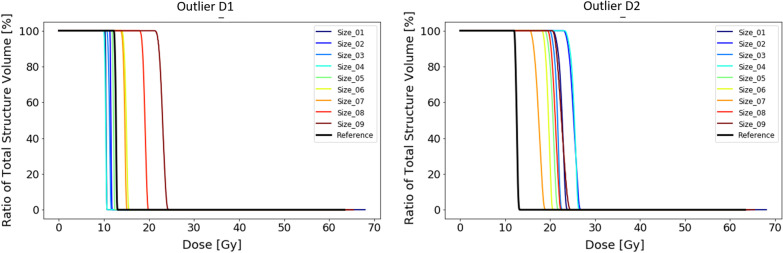
Results of the dose to the outlier volumes of experiment 4 involving OARs. On the left the DVH of the reference plan and the 9 plans for the different PTV sizes are shown in combination with outlier D1 (4.2 mm^2^). On the right the DVHs for the plans in combination with outlier D2 (12.6 mm^2^) are shown

Once we introduce OARs and their constraints to the optimization, the amount of freedom to the optimizer is more limited. Plans on the smaller sized PTVs led to less received dose by the outlier volume while for the larger PTV sizer the dose to the outlier volume is larger than for the reference plan (Fig. 13). From the animations of the dose distributions, it shows that outlier D1 is not receiving any dose.

For the slightly larger sized outlier D2, the dose received by the outlier for all the plans is larger than for the reference plan. The increasing sizes of PTV do not perfectly correspond to dose to the outlier volume, however there is a trend showing the smaller the PTV the higher the dose to the outlier (Fig. 13). The OARs and their constraints, especially the right hippocampus do have a direct influence on the way the outlier is accounted for by the optimizer.

### Outlier target segmentations from deep learning data

#### Amount of outliers

**Fig. 14:**
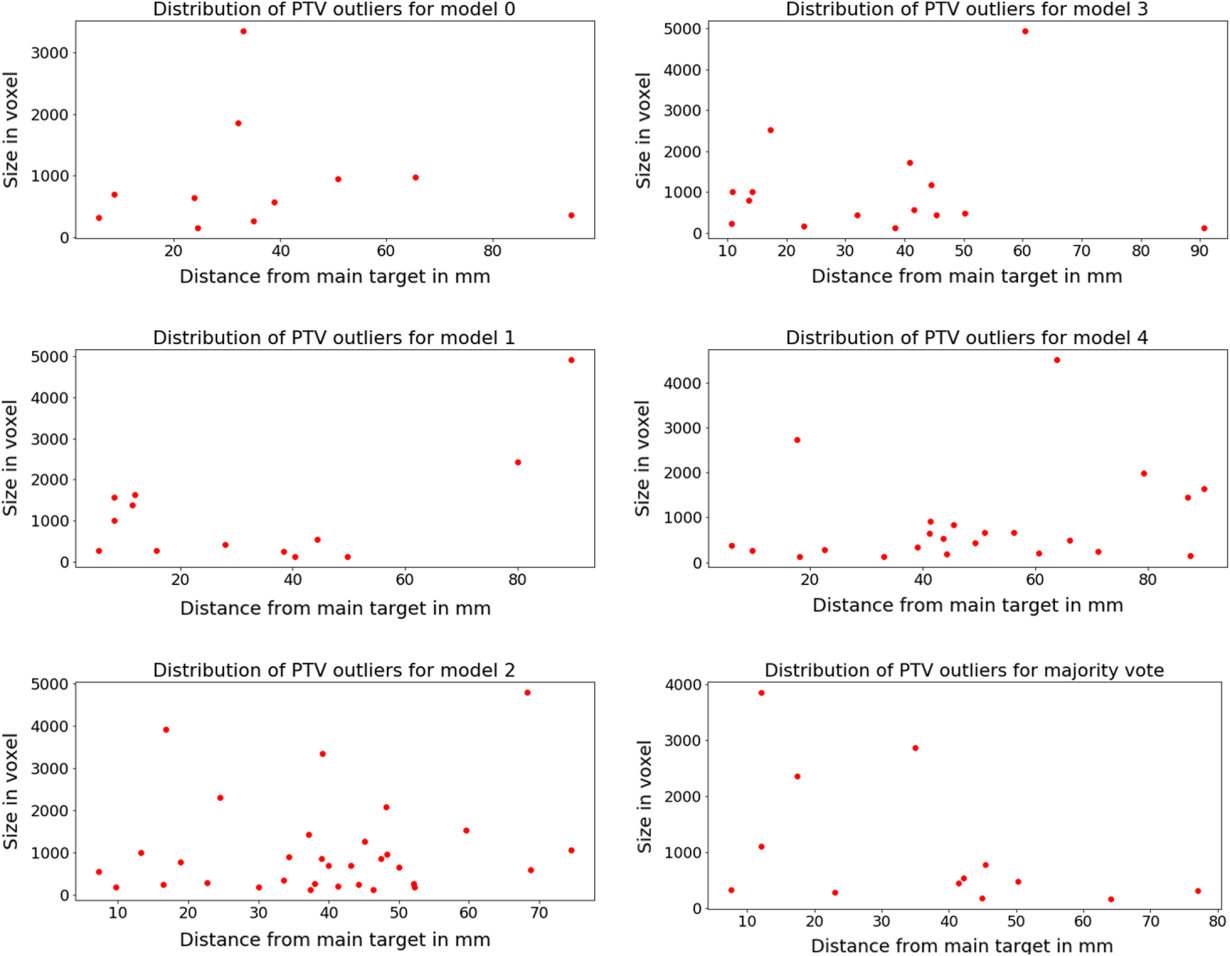
Distribution of size and distance from main target of the PTV predictions of the 20 cases. Each plot represents a different trained deep learning model. The bottom right shows the ensembling method by majority voting. The y-axis are scaled

For the 20 test cases, we have the GTV and the edema region predicted by 5 different trained models and additionally an ensemble method using a prediction based on majority voting. For the different nnUnet models, we found a combined amount of outliers, for all 20 cases and both the GTV and edema structures, that averaged 281.2 ± 10.5. Curiously, the majority voting prediction resulted in 481 outliers, evidently larger as for the other models. After construction of the PTV the average combined amount of outliers for the nnUnet models was 24.2 ± 5.5. The majority voting method resulted in a combined amount of 16 outliers, which is less than any of the separate models (Table 2). The distribution of the separate outliers in terms of size and closest distance from the main PTV structure is shown in Fig. 14 for each deep learning model and the ensemble method. For detailed overview of the results, we would like to refer to Additional file: 2.

**Table 2:** Auto-segmented outliers

		Model 1	Model 2	Model 3	Model 4	Model 5	Majority vote
GTV	56	61	47	53	64	209	209
Edema	243	201	259	208	214	272	272
PTV	17	18	36	22	28	16	16
	Mean distance (mm)	37.5	33.2	39	35.6	48.9	36.6
	Median dist, (mm)	33.0	27.9	39.6	38.4	45.4	41.5
	Mean size (mm^3^)	923.3	1150.1	1029.2	1053.1	856.4	1052.9
	Median size (mm^3^)	636	554	690	577	483	477

Number of outliers per structure per deep learning model and the ensembling method by majority vote method. Additionally for the outliers in the PTV structure the mean distance to the main target and the mean size is presented

#### Dose effect of outliers

**Fig. 15:**
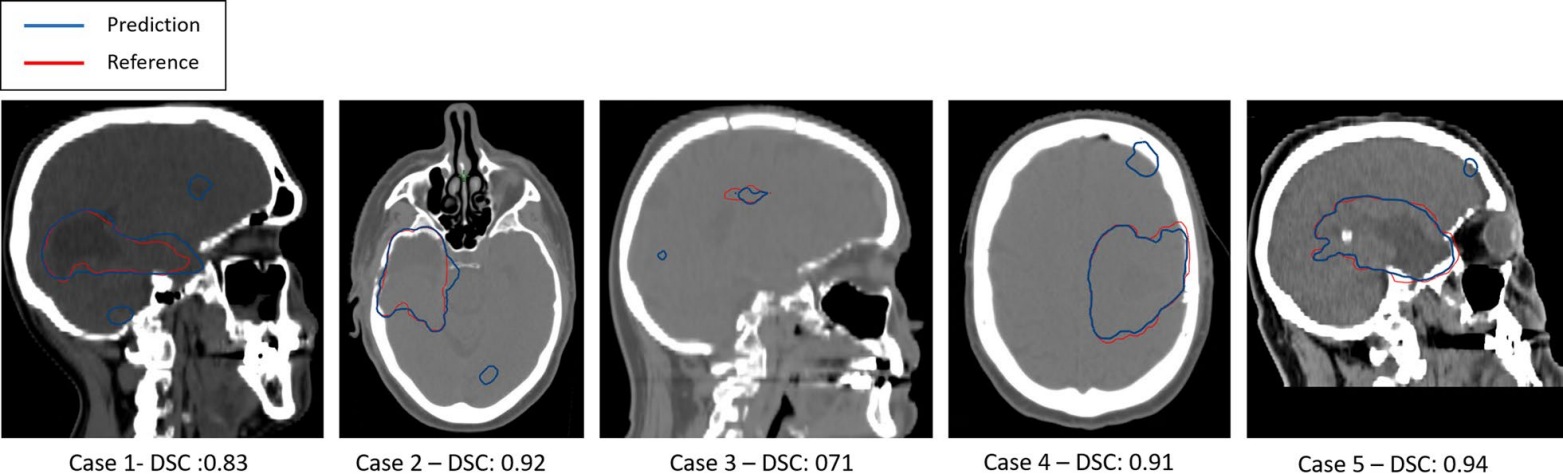
Contours of the predicted PTV (blue) and the reference PTV (red) of the five cases overlaid on an axial or sagittal slice of the planning CT to show the relation between the main target and the outlier. Additionally the dice similarity coefficient (DSC) of the predicted PTV versus the reference PTV is given

Out of the 120 constructed PTVs from the predicted GTV and edema structures test cases, 56 cases had one or more outliers. Five of these cases, from any of the deep learning models or the majority vote result, are selected for dosimetric analysis of the outlier (Fig. 15).

For each of the selected cases we compared the plan calculated on the predicted PTV containing the outlier (predicted outlier) and the plan calculated on the predicted PTV with the outlier removed (removed outlier) with the reference plan based on reference PTV drawn manually by an RT professional.

**Fig. 16:**
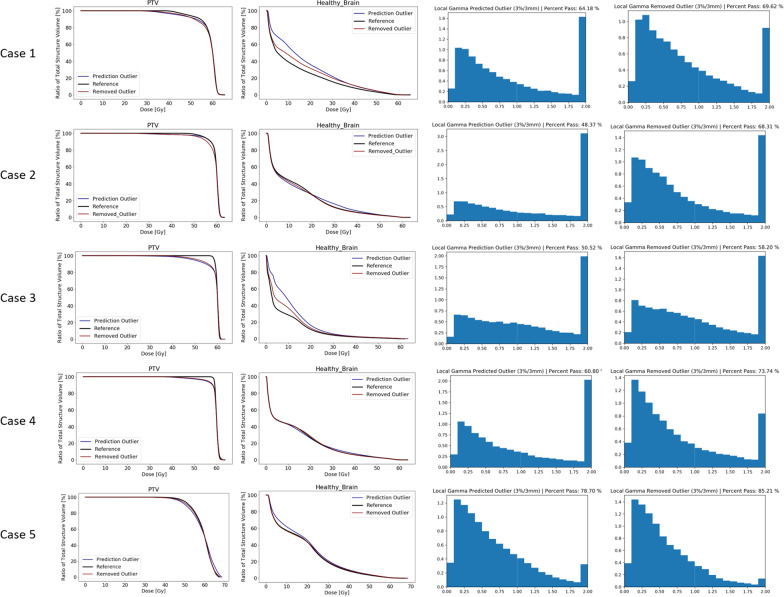
Dosimetric analysis of the predicted outlier and the removed outlier plan versus the reference plan. In the left column, the DVH of the PTV is displayed. The second column shows the DVH of the healthy brain tissue. The third and fourth column show the gamma analysis result of the predicted outlier and the removed outlier plans to the reference plan respectively

From the perspective of the reference plan, the dose coverage of the reference plan is superior to the plans based on the predicted targets. Depending on the similarity of the predicted PTVs to the reference, the dose coverage approached that of the reference plan (Fig. 16). The outlier did not show to have an effect on the dose coverage. In the DVH for healthy brain tissue, we see that in cases 1, 3 and 5, even though having similar DSC to the reference PTV, the predicted outlier plan shows increased doses. This relatively large difference with respect to case 2 and case 4 is most likely because the outlier lies above or below the axial range of the main PTV target.

The gamma pass rate for each case was better for the removed outlier plan than for the predicted outlier plan. On average, the gamma pass rate improved with 10.5%, ranging from 5.4% for case 1 to 19.9% for case 2. This means that for the predicted outlier plans the three-dimensional dose distribution deviated more from the reference plan then the removed outlier plan according to the 3% and 3 mm criterion (Fig. 16).

If we focus at the DVH of the specific OARs, we observe that in case 1 and case 3 the dose in many OARs was higher for the predicted outlier plan than for the removed outlier plans. In these particular cases the outlier is located inferior of the main target towards the base of skull, where the majority of OARs are located. In the other cases there is not much dose effect in the OARs. There are even some OARs receiving less dose in the predicted outlier plan than for the removed outlier plan. I.e. the right optic nerve in case 2 and the right hippocampus in case 5.

## Discussion

In this study, we investigated the effects of random outliers, here defined as isolated false positive segmentations, of the target volume in case of deep learning-based auto-segmentation. Besides how often outliers occur for a current DL model, we looked at the dosimetric influence of the location, size, shape and size relative to the actual PTV.

Based on the synthetically generated outliers, results showed that false positive segmentations can have an influence on the dose distribution. In general, we found that in the presence of outliers, the dose coverage of the target is not compromised, but additional dose is added to the healthy tissues. The extent of the dosimetric effects is dependent on the relative size, location, and the distance to the main target and involved OARs. Though in general, the larger the outlier volume and the larger the distance to the actual target, the higher the potential dosimetric effect is for the healthy tissues.

In specific cases where an outlier is small enough the optimizer of the Eclipse TPS ignored the outlier and no direct dose was applied to the area. In this case, the cost for covering the outlier probably exceeded the costs for leaving the outlier without any dose. In general, the cost to cover the outlier increases when (i) it is further away from the original target, (ii) when it is outside the range of the dominant beam directions and (iii) when it is close to an OAR which has a limiting constraint.

Additionally, the smaller the relative size of the outlier is to the actual target, the less attention it will receive from the optimizer to cover it with dose. Evidently, this is highly dependent on the goals, constraints and the weights that are set for the plan optimizer. If the desired target coverage is set very progressively, close to 100%, with a high relative weight, coverage of any false positive outlier will be more likely. As opposed to a more conservative approach where coverage of the target is less important, small outliers can be ignored because they fall outside the target volume that needs to be covered.

The deep learning results we used are predominantly a result of state-of-the-art networks and trained models that are currently available [38, 39], and based on current best practices in deep learning for medical image analysis. Nonetheless, outliers can occur in practice and their location and appearance is unpredictable. Although the models employed are not part of a commercially available solution, our goal with this study was to address the fact that random outliers are a real world problem when working on deep learning-based segmentation models. Our results yielded one or more of such outliers in about half of the tested cases.

Eventually, 5 cases containing an outlier were selected to show the possible dosimetric consequences of having a false positive in the target volume. We acknowledge that such limited number of cases cannot give a significant outcome on the average increased dose or the increased clinical risks. However, these results show that such outliers can occur, and have an evident and predominantly negative effect on the dose distribution with respect to a plan based on the ground truth. Compared to the removed outlier plan, we observed that outliers are dominant contributors of the negative dosimetric effect.

Noticeably, the dosimetric effect are mainly present locally in the path between the actual target and the outlier. If certain conditions are met, the dose to the healthy tissue in this area can increase with a few dozen gray. In addition, defined OARs in the vicinity will be exposed to higher mean and or max dose levels. In conclusion, isolated false positive segmentation of the target typically will have a detrimental effect on dose distribution and could lead to an increased chance of toxicity.

To the best of our knowledge, no comparable studies have been performed up to now. We could not find previous studies analyzing outliers or any other specific errors on auto-segmentation of targets. There is some literature available on the dosimetric effects of contouring variations on targets, but these mainly focus on differences or lack of compliance to protocol guidelines and inter-rater effects [24, 42, 43]. None of the studies actually mentions specific false positive outliers. There is also some literature available on quality assurance for auto-segmented target structures [44–46]. However, none of these articles focuses on intracranial tumors or mentions false positive outliers specifically.

This work particularly focuses on the outliers that could occur with deep learning-based auto-segmentation in the targets in the brain. This is motivated by the observation that deep learning models excel in accuracy but lack precision, leading to false positives, which is outlined by a summary of the results of the brain tumor segmentation challenge [33]. Furthermore, it focuses on the dosimetric impact of a VMAT treatment delivered by a Truebeam delivery system and optimized on the Eclipse TPS. Although this is a widely used system and VMAT is a common treatment modality in the developed world for GBMs, the results in this study are only true for this specific setup. The hardware, software, treatment prescription, optimizer settings and beam setup will all have an influence on the result. The exact results are therefore not generalizable but the main principles of how random false positive errors influence the dosimetry could be true for other systems or even other diseases and target locations.

One could question the relevance of determining the dosimetric impact of false positive target segmentations. As it is obvious that random outliers can be detrimental for treatment planning and can have negative effects on the dosimetry, the issue should be tackled earlier in the process. During manual contouring such errors are unlikely to occur. Besides, such errors can be detected during inspection, especially when considerate isotropic margins are used to define the CTV and the PTV, a random error would likely be more visible. If planning is performed manually, this provides an additional possibility where such errors would likely be detected, especially when they impact the dose.

Until now, there has been no need for this knowledge. However, in our experience with deep learning, in both OARs and target structures, we see random false positive segmentations occurring. Treatment planning will become more and more automated in the future. While deep learning technologies are being developed by companies in the field, errors stemming from deep learning systems need to be considered in the process. Obviously, the first priority is to make the deep learning methods more robust in their initial models, but also via post processing steps aiming at spotting and eliminating false positive errors. Nonetheless, further understanding the root cause of these errors is in our opinion crucial to ensure robustness and trustability of deep learning systems. In these regards, the results presented in this study also aim at promoting and raising the awareness of the deep learning research community towards a more balanced focus of accuracy and precision (robustness) training and evaluation metrics.

One possible strategy in improving robustness of automatic segmentations based on deep learning are ensemble methods [47]. In particular when combining results of models that are derived from distinct network architectures and therefor focus on different features, random false positives might be avoided. In this work, we had the opportunity to test a simple version of ensemble learning by taking the majority vote of one specific network that was trained in a fivefold split. Our results suggest that ensembling does not reduce the total number of outliers in the GTV and edema structures however, we noticed a beneficial reduction of outliers after construction of the PTV (average reduction of 33.8%), which further contributes to an improved dosimetry when using such strategy. An important issue in improving robustness is the interpretability of AI models. Interpretability is mentioned a lot recently as a requirement for clinical implementation [48, 49], but it could also be a key in understanding why a deep learning model makes a mistake. Once you know the underlying flaws of a model, you can focus on improving them.

We think it is unlikely that automatic target segmentations will be used without proper inspection of a trained radiation oncologists in the foreseen future. We do think it is valuable to know the dosimetric effects and what possible clinical impact such errors might bring about. Understanding why the deep learning models make these typical errors is complicated. Improving the models in terms of robustness is therefore challenging. Post-processing of target definition is not a straightforward process either. There is little prior knowledge on tumor in the brain to discriminate false positives from true positives. This step is currently only possible with the interaction of a trained radiation oncologists. A QA system can help in recognizing possible errors and request the input of the physician. The knowledge from this study could make such a process more efficient by helping us define when outliers should be addressed and which ones could be ignored.

## Conclusion

In this study, we show that there is a severe risk of false positive outliers in modern DL predictions of target structures. These errors will have an evident detrimental impact on the dose and therefore could affect treatment outcome. Additionally, we showed that ensembling different models by majority vote is a strategy that can reduce outliers.

## Supplementary Information

**Additional file 1.** Results synthetic experiments.

**Additional file 2. ** Results deep learning segmented outliers.

**Additional file 3.** Gifs of dosimetric results segmented outliers.

## Data Availability

The imaging data generated and/or analyzed during the study are not publicly available due to privacy and confidentiality. All results of the analysis are made available as supplementary material. Additional data on the DL method or methods are available from the corresponding author on reasonable request.

## Abbreviations

AI: Artificial intelligence
cm: Centimeter
CT: Computed tomography
CTV: Clinical target volume
D95%: Dose received by 95 procent of the volume
D98%: Dose received by 98 procent of the volume
DL: Deep learning
DVH: Dose volume histogram
GBM: Glioblastoma
GTV: Gross tumor volume
Gy: Gray
mm: Milimeter
MRI: Magnetic resonance imaging
OAR: Organ at risk
PTV: Planning tumor volume
QA: Quality assurance
RT: Radiation therapy
RTOG: Radiation therapy oncology group
TPS: Treatment planning system
VMAT: Volumetric modulated arc therapy
