# Supplementary material for: Impact of random outliers in auto-segmented targets on radiotherapy treatment plans for glioblastoma
Source: Radiat Oncol. 2022 Oct 22;17:170. doi: 10.1186/s13014-022-02137-9 (PMC9587574; doi:10.1186/s13014-022-02137-9)
Supplement: Supplementary file 1 — Additional file 1. Results synthetic experiments. [file 13014_2022_2137_MOESM1_ESM.pdf]

Additional File A:

Results Synthetic Experiments

Experiment 1: Outlier location, no OARs involved:

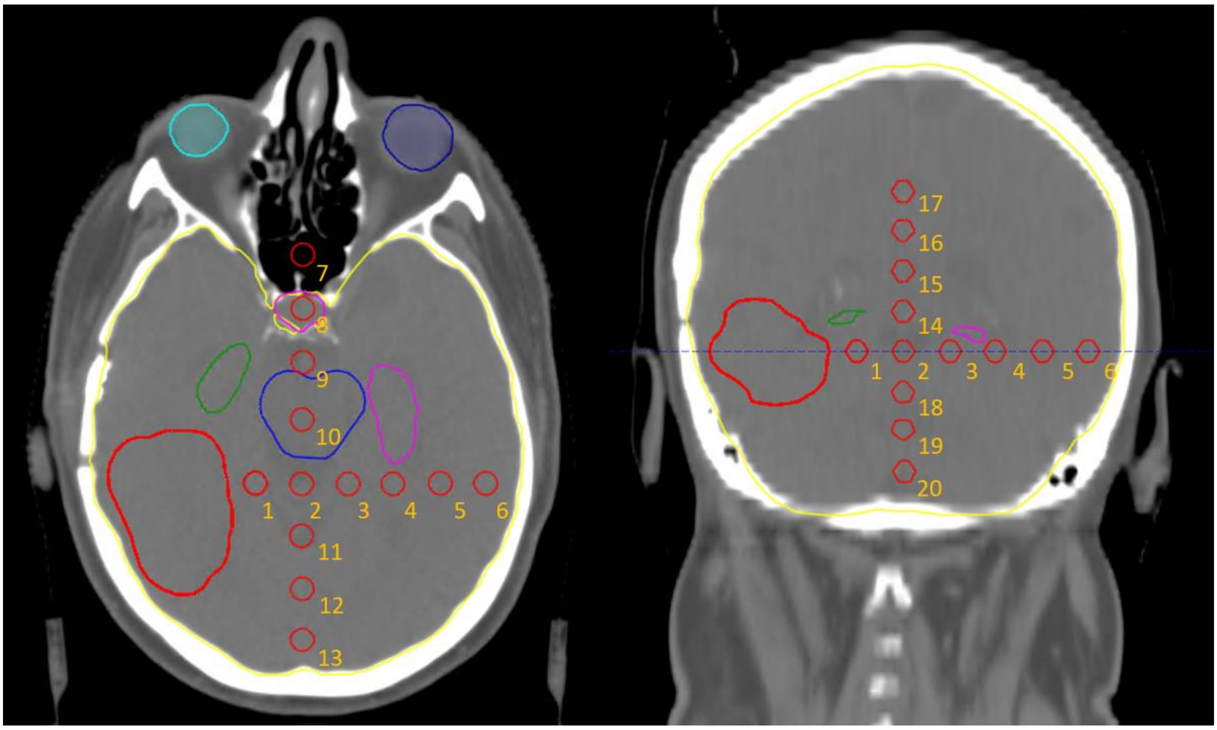

Figure 1: Overview of the synthetic setup of experiment 1. The large red volume is the reference PTV. Along three axes 20 outlier volumes, each of size 0.13cm², were generated and labelled 1 to 20.

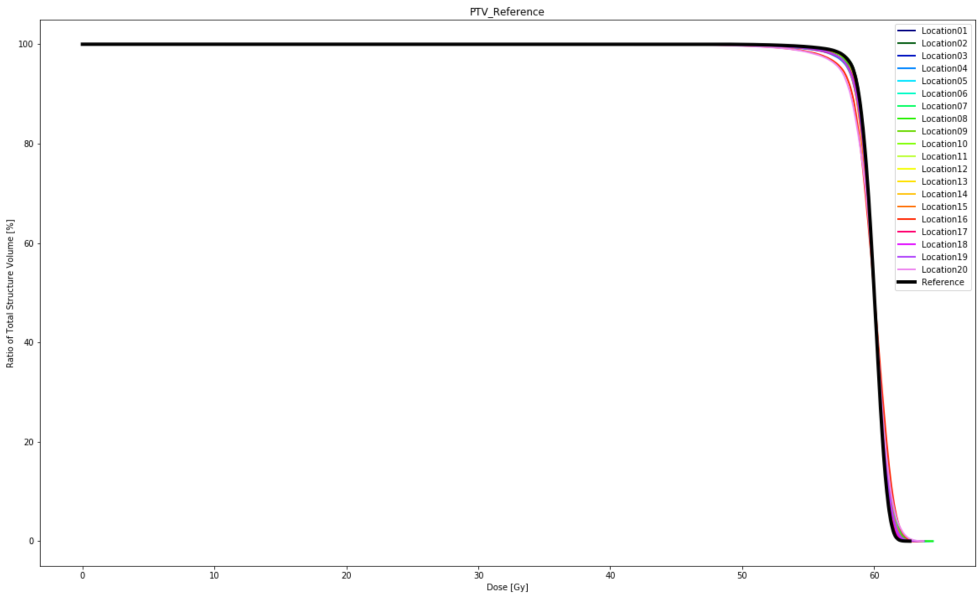

Figure 2: DVH curves of PTV structure of the reference plan and the 20 plans including an outlier to the target at the specific location displayed in Figure 1.

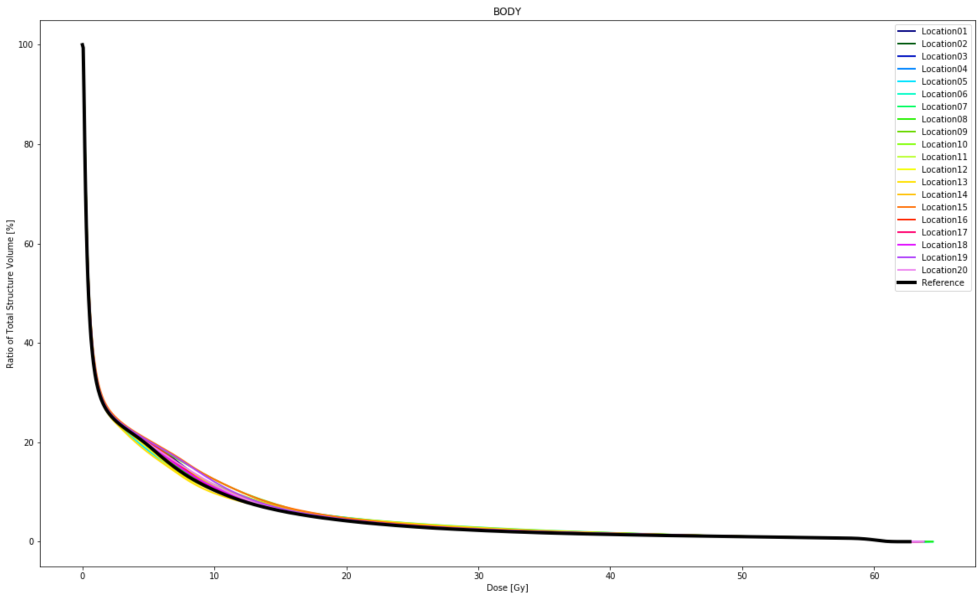

Figure 3: DVH curves of body structure of the reference plan and the 20 plans including an outlier to the target at the specific location displayed in Figure 1.

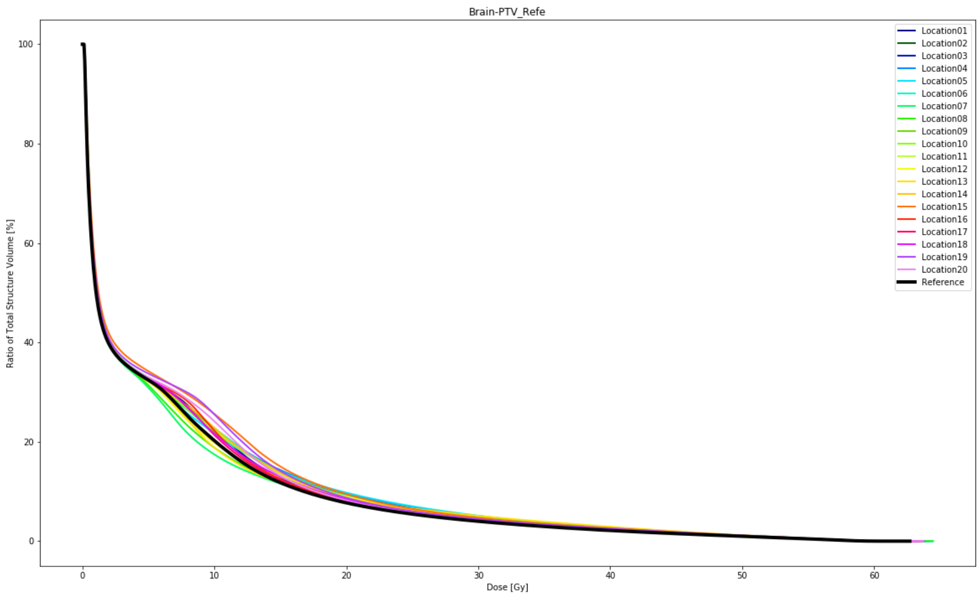

Figure 4: DVH curves of healthy brain, i.e. brain tissue minus the reference PTV of the reference plan and the 20 plans including an outlier to the target at the specific location displayed in Figure 1.

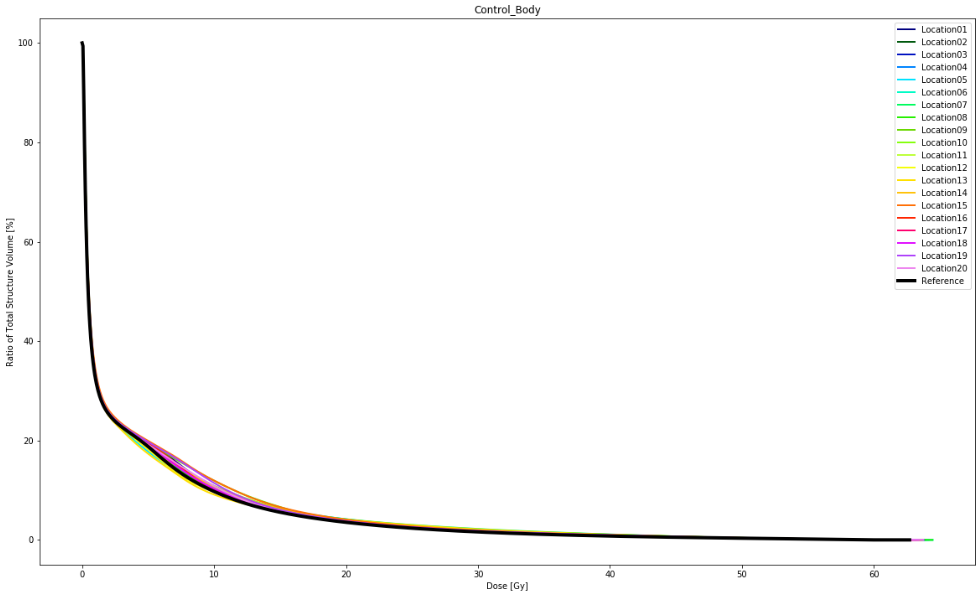

Figure 5: DVH curves of control body structure, i.e. the body minus the reference PTV, of the reference plan and the 20 plans including an outlier to the target at the specific location displayed in Figure 1.

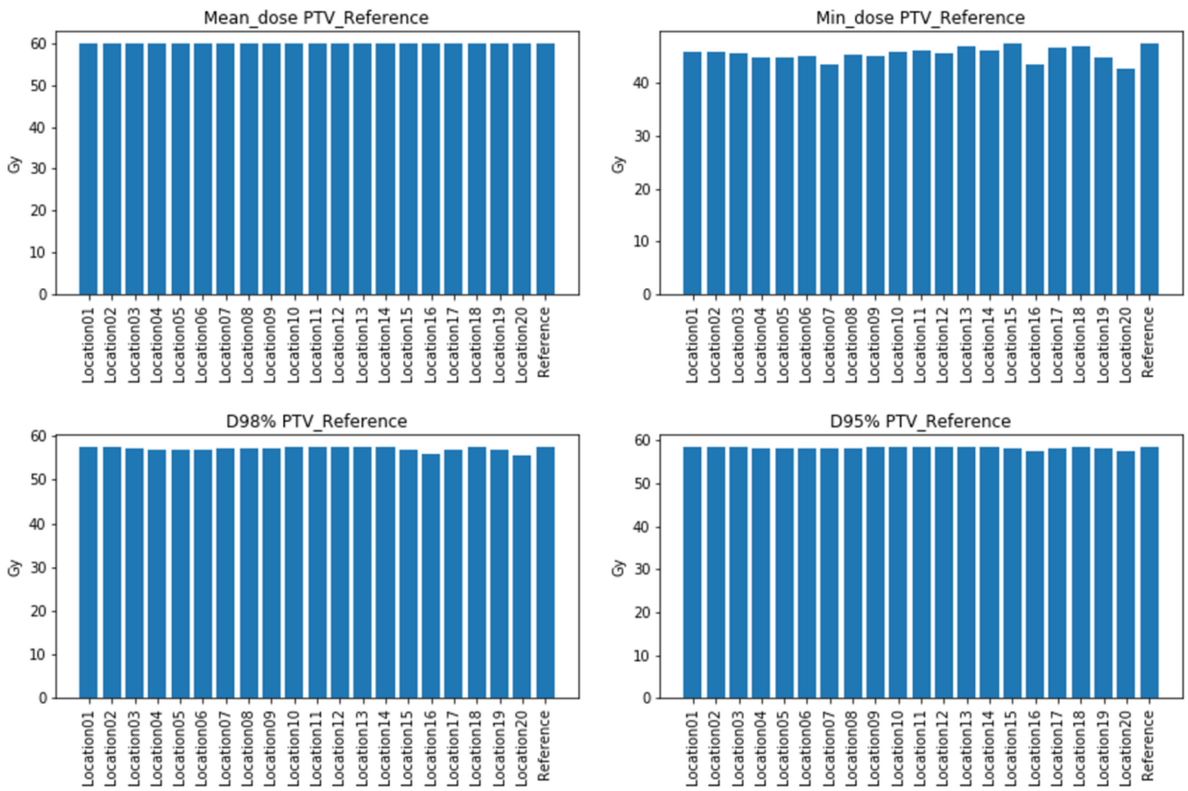

Figure 6: Bar plots of the mean dose, min dose and the 98% and 95% coverage of the PTV for the reference plan and the 20 plans including an outlier to the target at the specific location displayed in Figure 1.

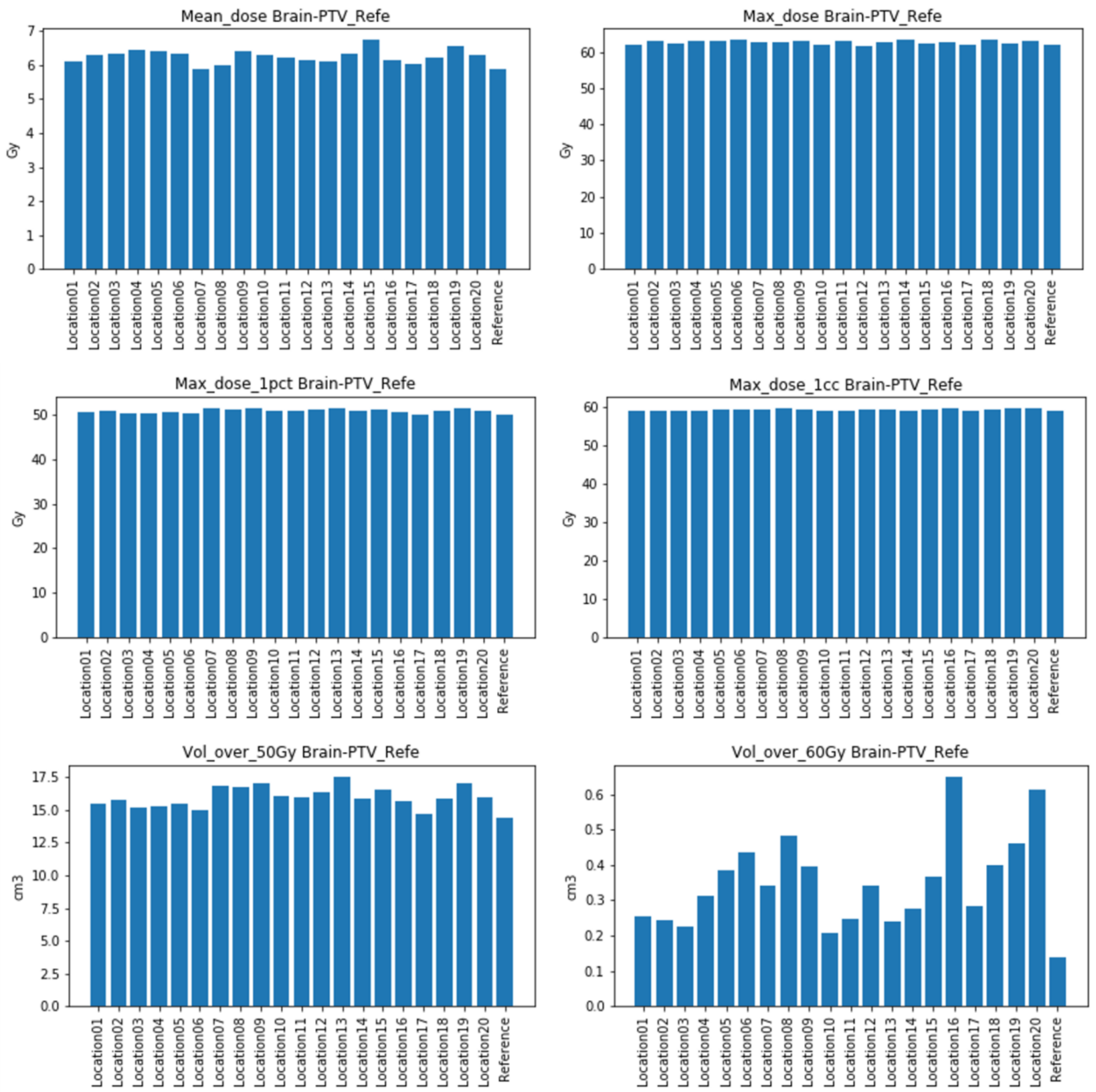

Figure 7: Bar plots of the mean dose, max dose, max dose to 1% of the volume, max dose to 1 cc of the volume, the volume receiving over 50Gy and the volume receiving over 60 Gy of the Healthy brain structure (Brain minus PTV), for the reference plan and the 20 plans including an outlier to the target at the specific location displayed in Figure 1.

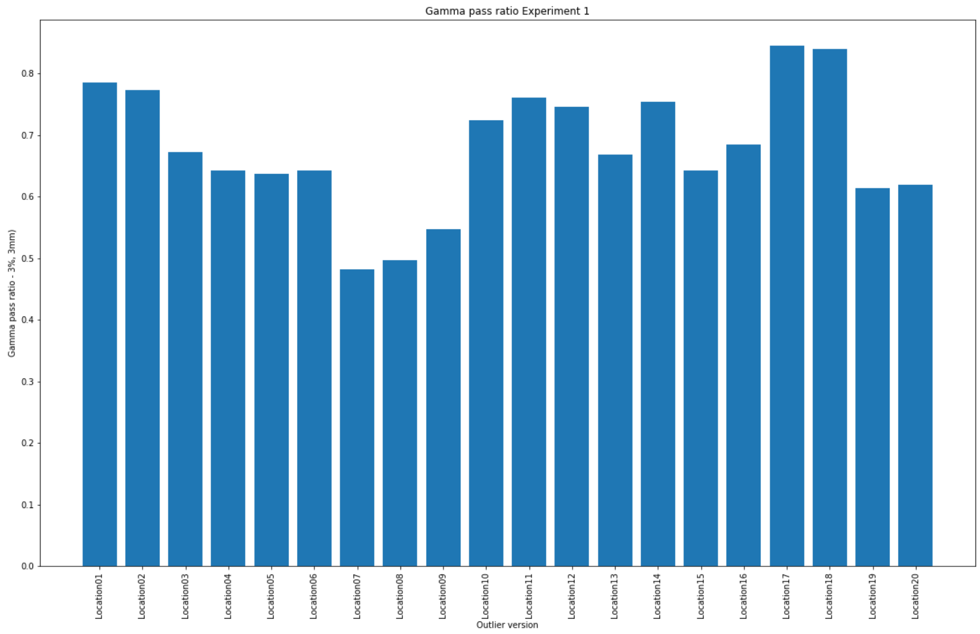

Figure 8: Bar plot of the Gamma pass ratio of each experimental plan containing an outlier at a specific location with respect to the reference plan. The criteria for the gamma pass rate were set to 3% and 3 mm.

Experiment 1: Outlier locations with OARs involved:

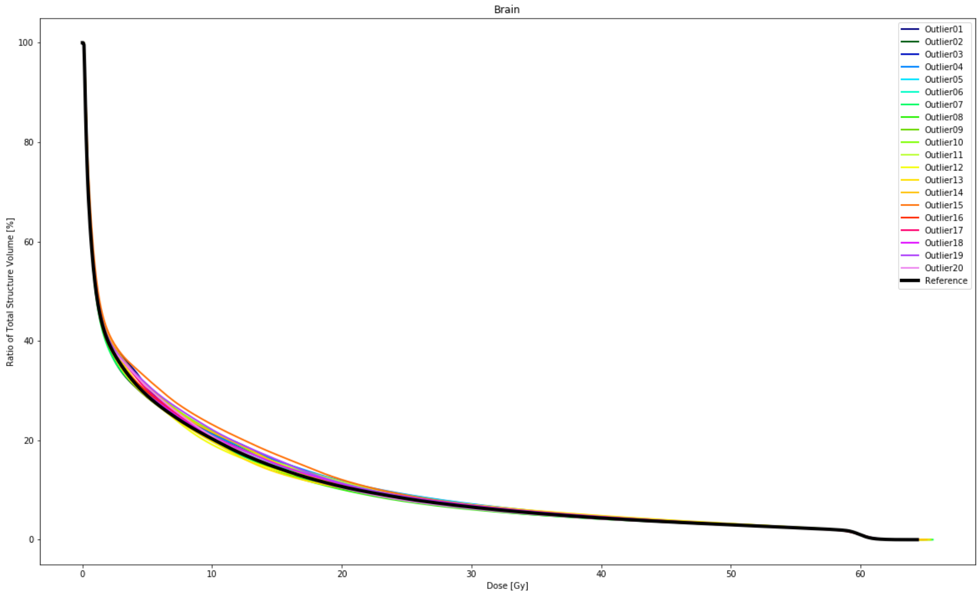

Figure 9: DVH curves of the brain of the reference plan and the 20 plans including an outlier to the target at the specific location displayed in Figure 1.

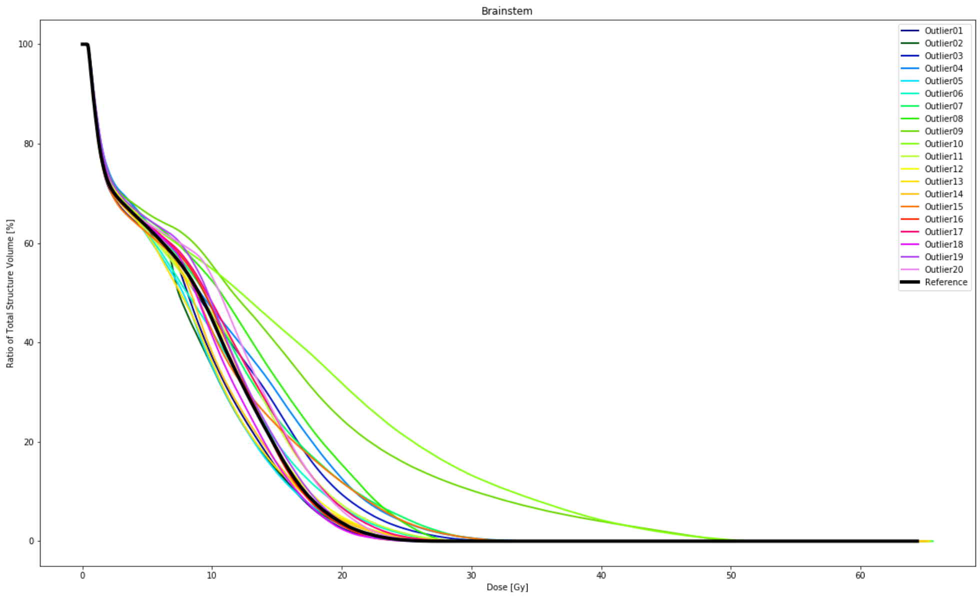

Figure 10: DVH curves of the brainstem of the reference plan and the 20 plans including an outlier to the target at the specific location displayed in Figure 1.

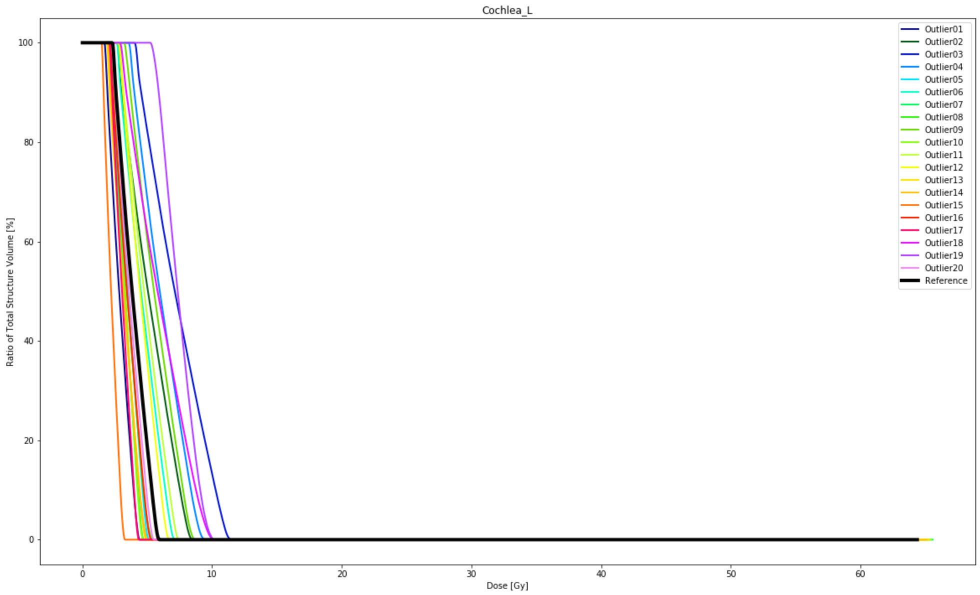

Figure 11: DVH curves of the left cochlea of the reference plan and the 20 plans including an outlier to the target at the specific location displayed in figure 1.

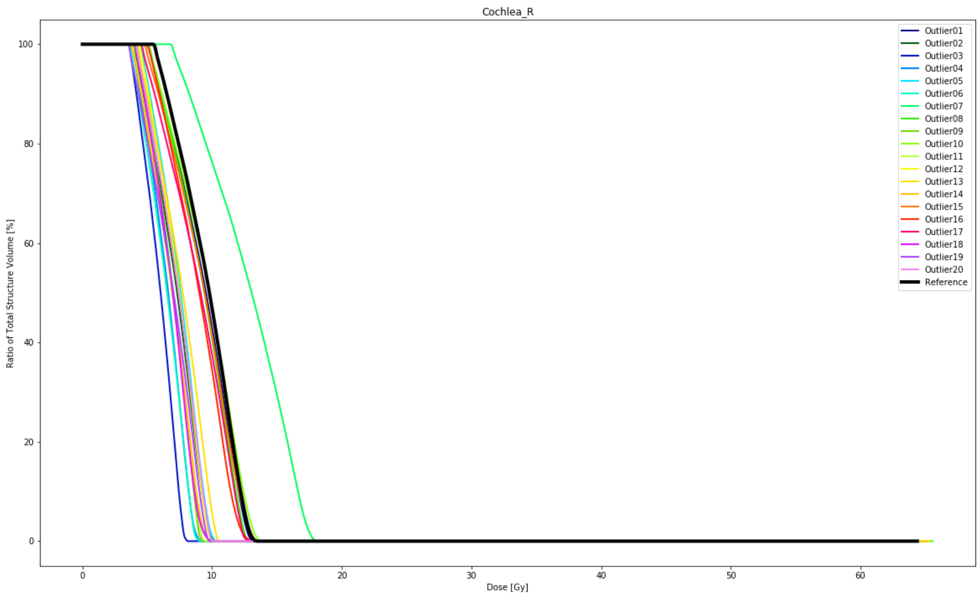

Figure 12: DVH curves of the right cochlea of the reference plan and the 20 plans including an outlier to the target at the specific location displayed in Figure 1.

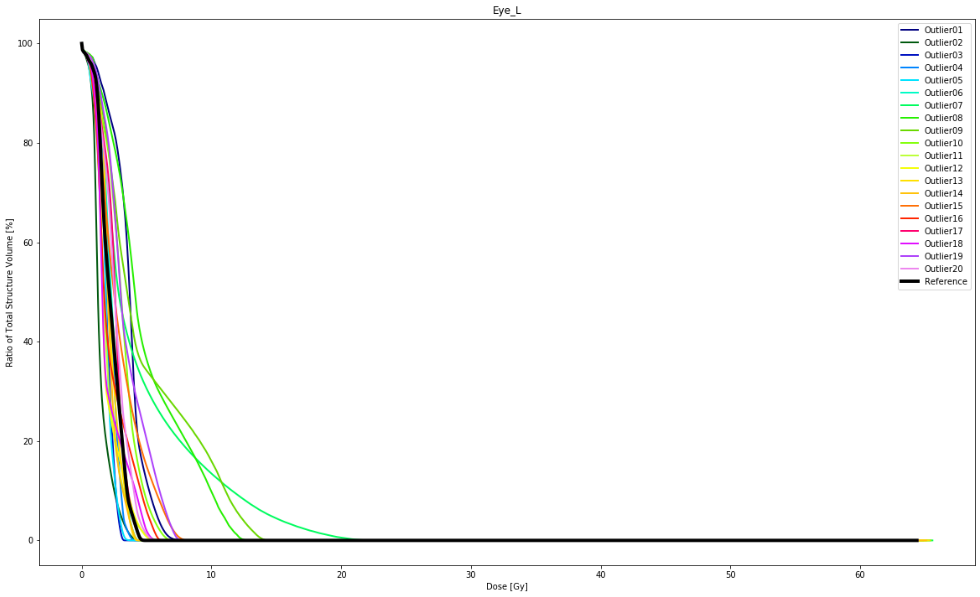

Figure 13: DVH curves of the left eye of the reference plan and the 20 plans including an outlier to the target at the specific location displayed in Figure 1.

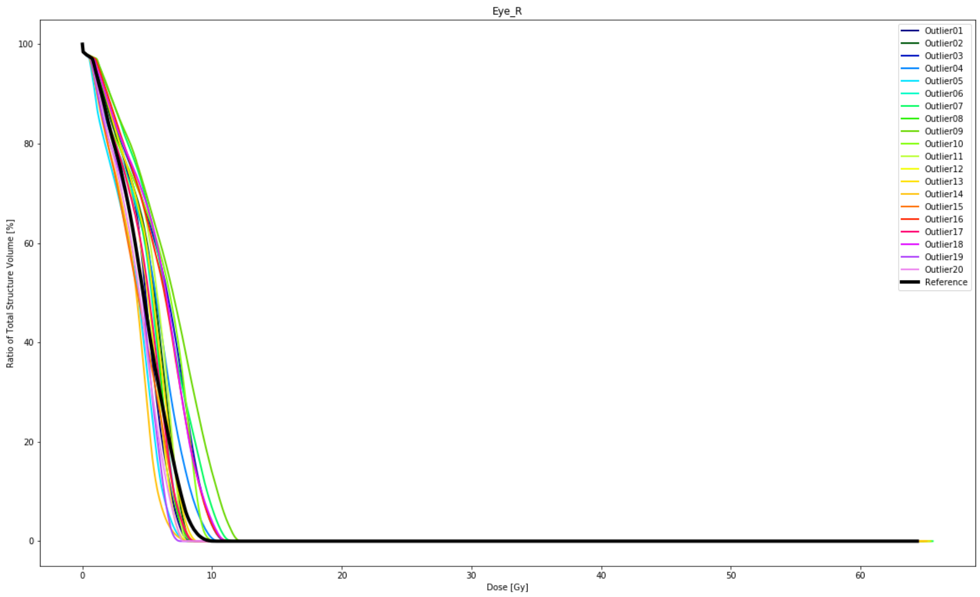

Figure 14: DVH curves of the right eye of the reference plan and the 20 plans including an outlier to the target at the specific location displayed in Figure 1.

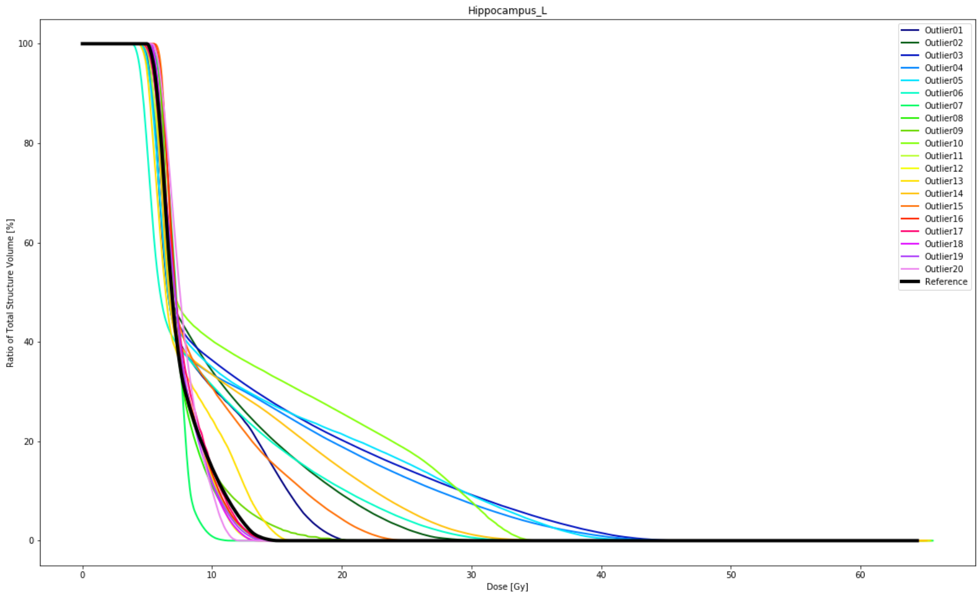

Figure 15: : DVH curves of the left hippocampus of the reference plan and the 20 plans including an outlier to the target at the specific location displayed in Figure 1.

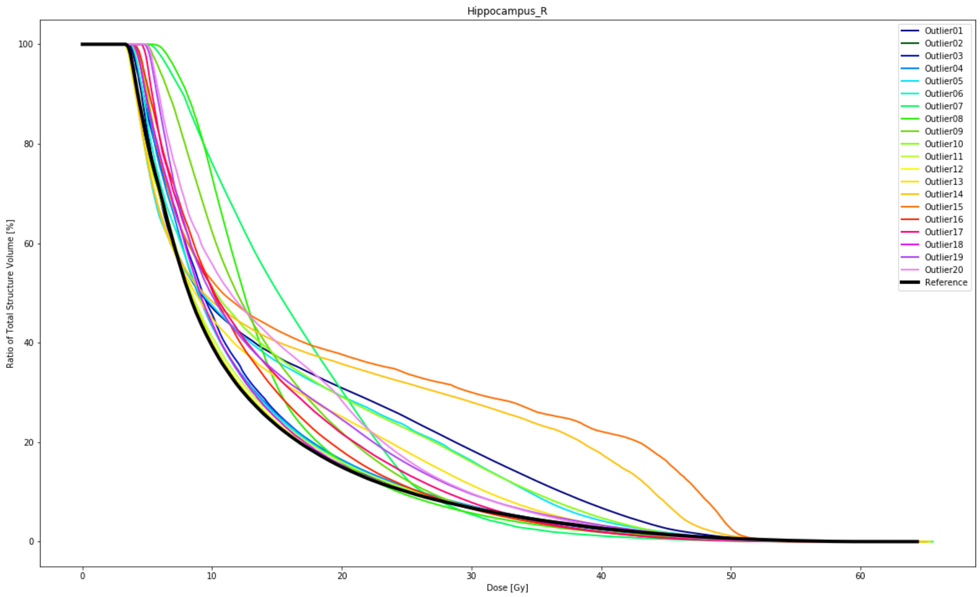

Figure 16: : DVH curves of the right hippocampus of the reference plan and the 20 plans including an outlier to the target at the specific location displayed in Figure 1.

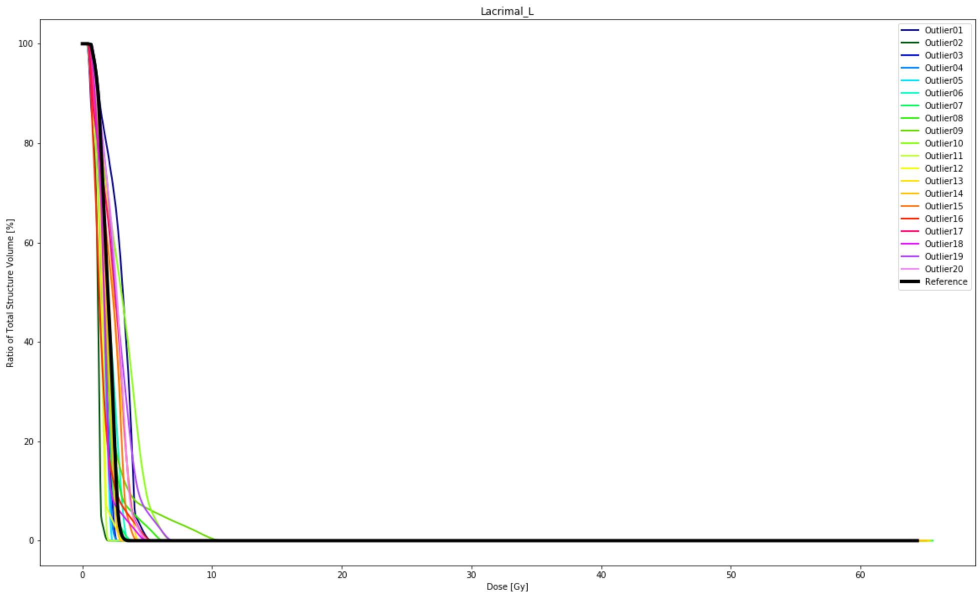

Figure 17: DVH curves of the left lacrimal gland of the reference plan and the 20 plans including an outlier to the target at the specific location displayed in Figure 1.

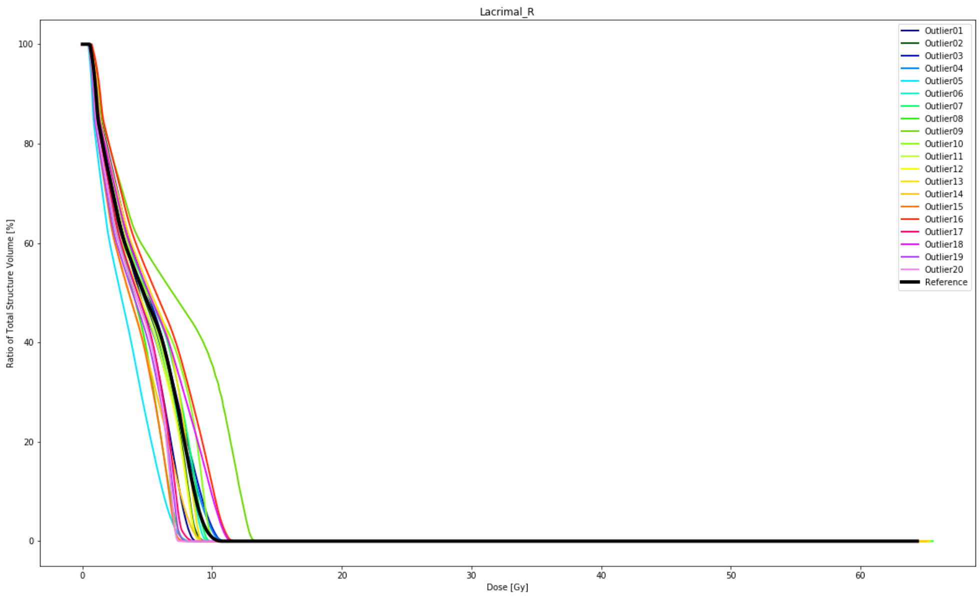

Figure 18: DVH curves of the right lacrimal gland of the reference plan and the 20 plans including an outlier to the target at the specific location displayed in Figure 1.

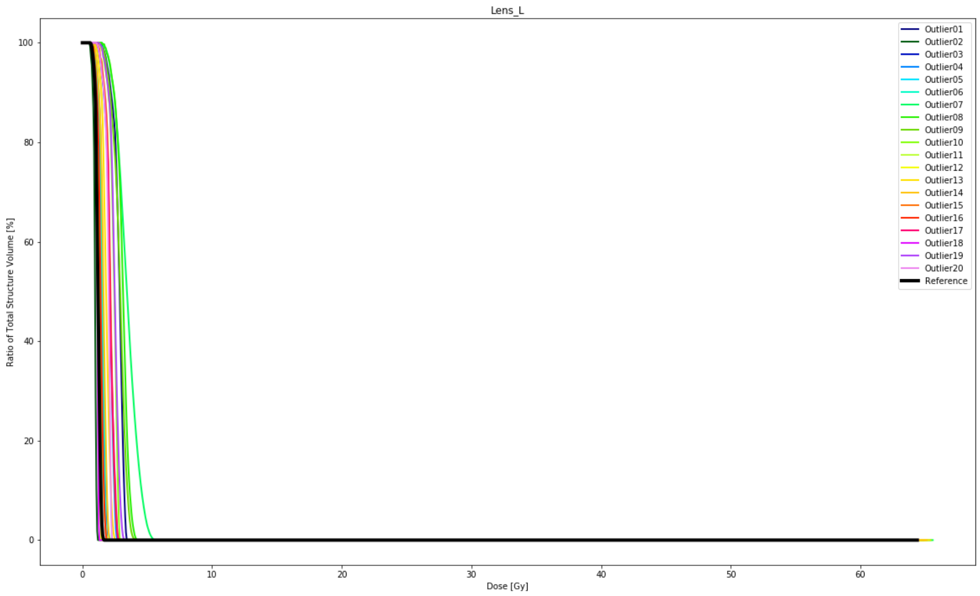

Figure 19: : DVH curves of the left lens of the reference plan and the 20 plans including an outlier to the target at the specific location displayed in Figure 1.

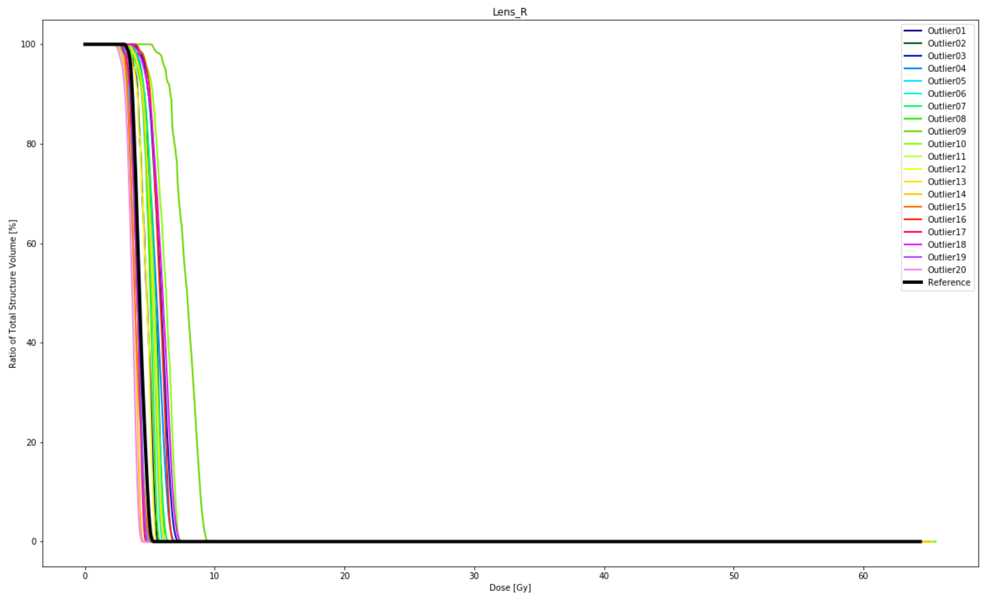

Figure 20: : DVH curves of the right lens of the reference plan and the 20 plans including an outlier to the target at the specific location displayed in Figure 1.

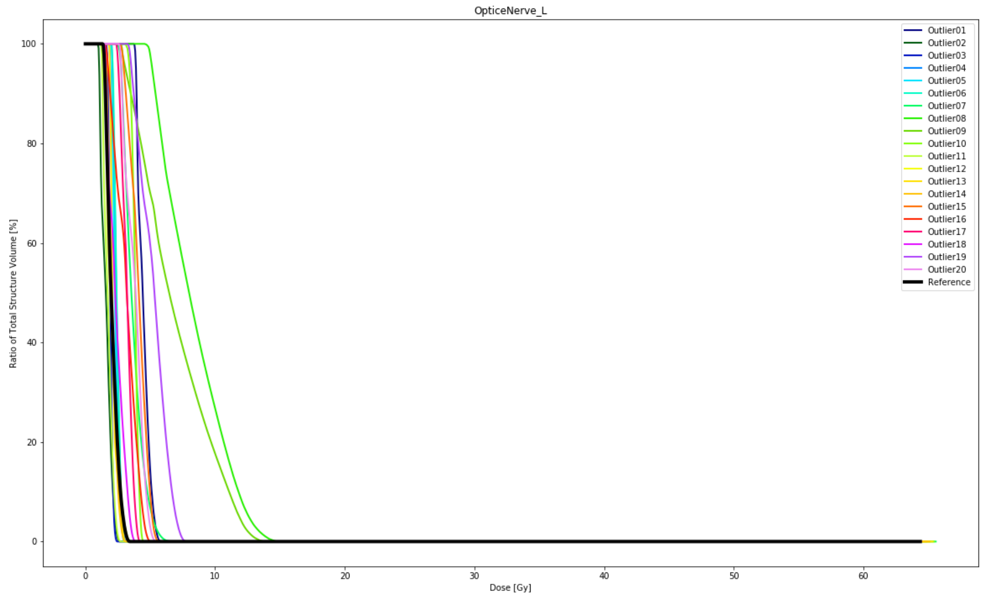

Figure 21: DVH curves of the left optic nerve of the reference plan and the 20 plans including an outlier to the target at the specific location displayed in Figure 1.

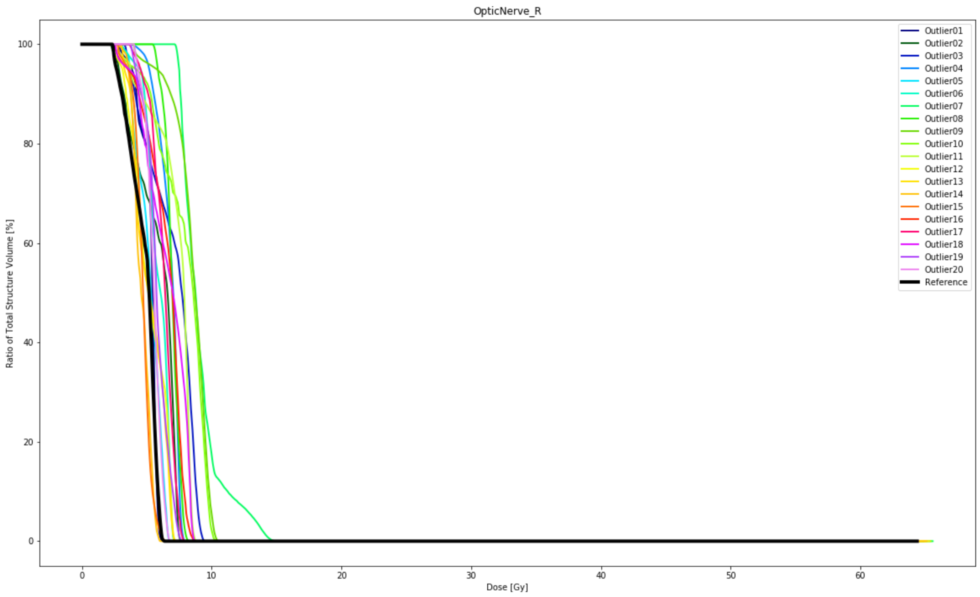

Figure 22: DVH curves of the right optic nerve of the reference plan and the 20 plans including an outlier to the target at the specific location displayed in Figure 1.

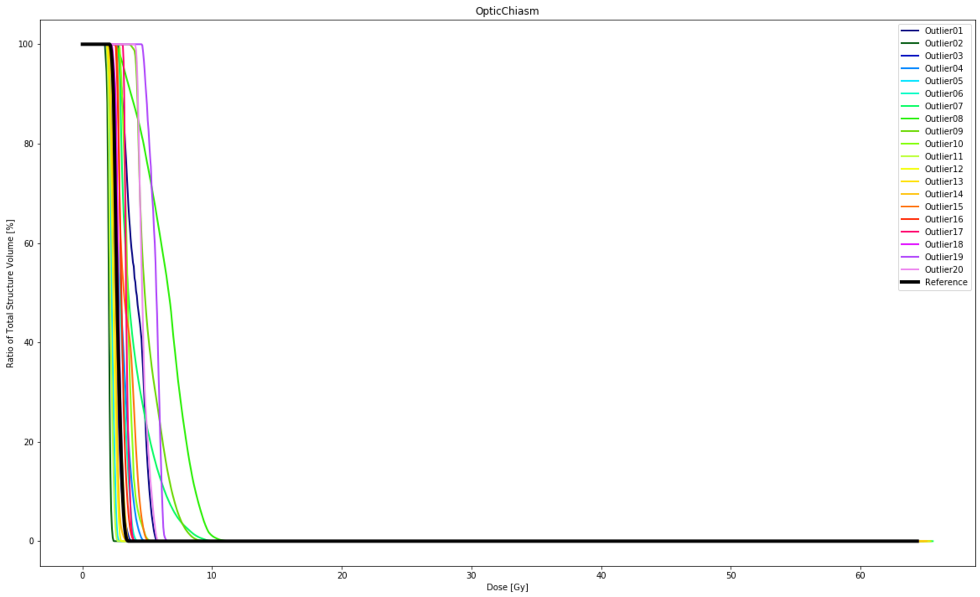

Figure 23: DVH curves of the optic chiasm of the reference plan and the 20 plans including an outlier to the target at the specific location displayed in Figure 1.

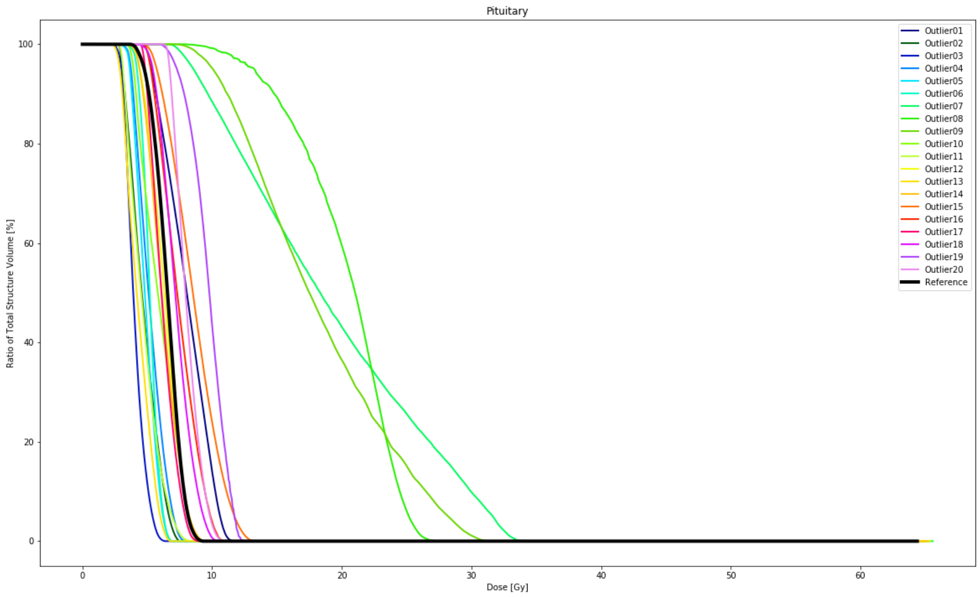

Figure 24: DVH curves of the pituitary gland of the reference plan and the 20 plans including an outlier to the target at the specific location displayed in Figure 1.

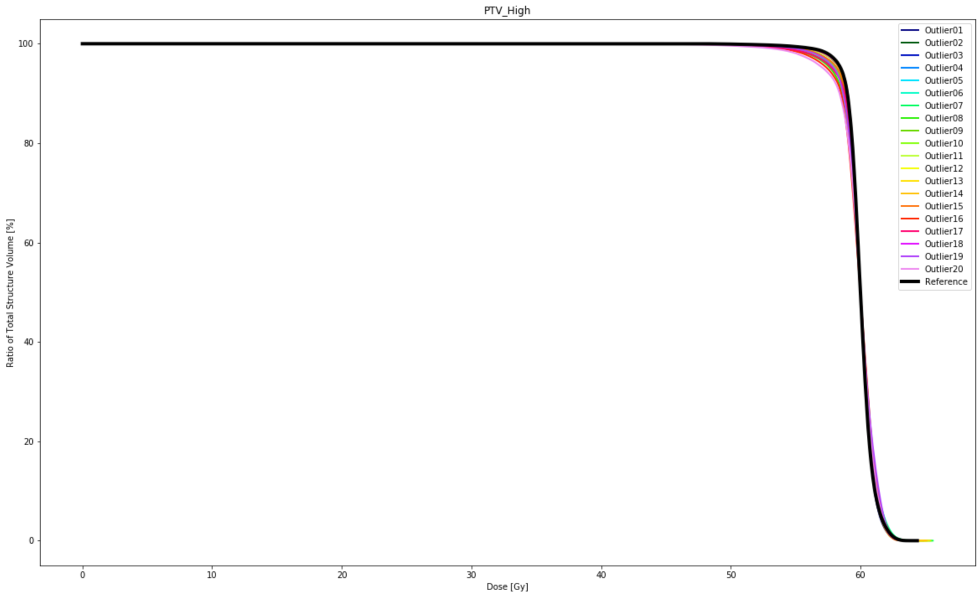

Figure 25: DVH curves of the PTV of the reference plan and the 20 plans including an outlier to the target at the specific location displayed in Figure 1.

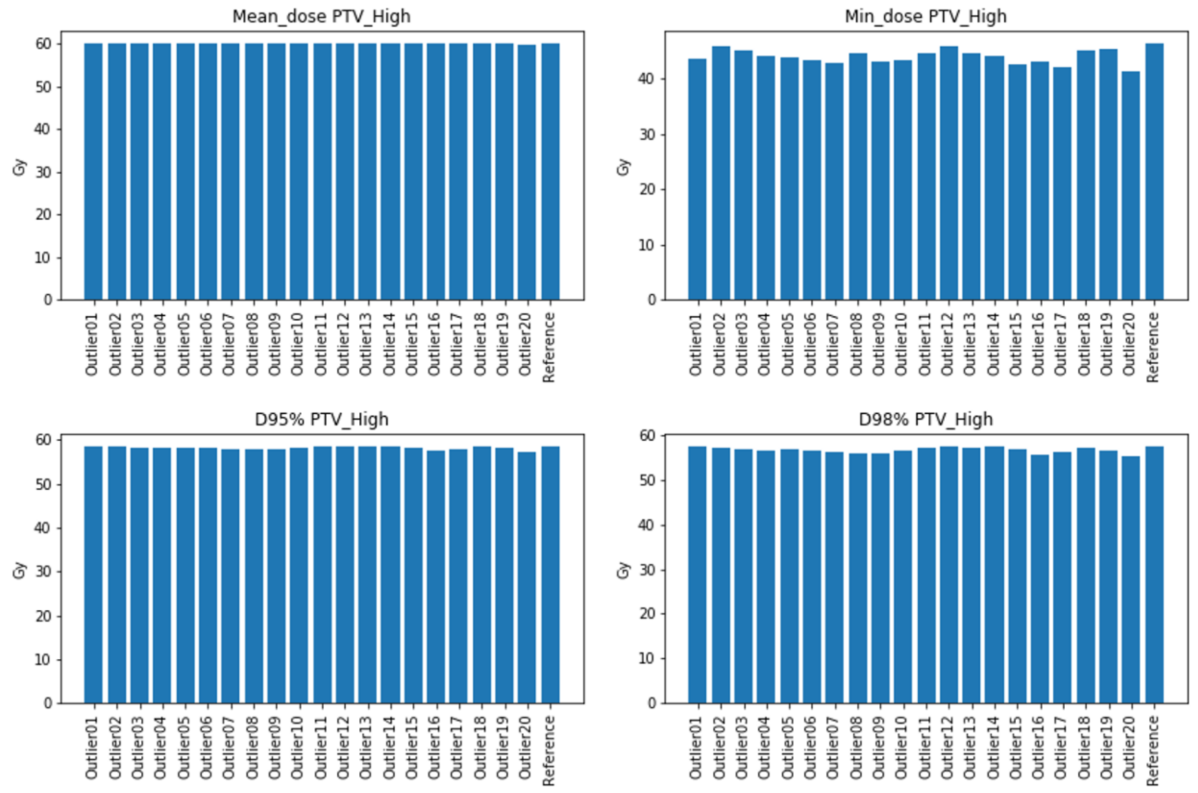

Figure 26: Bar plots of the mean dose, min dose and the 98% and 95% coverage of the PTV for the reference plan and the 20 plans including an outlier to the target at the specific location displayed in Figure 1.

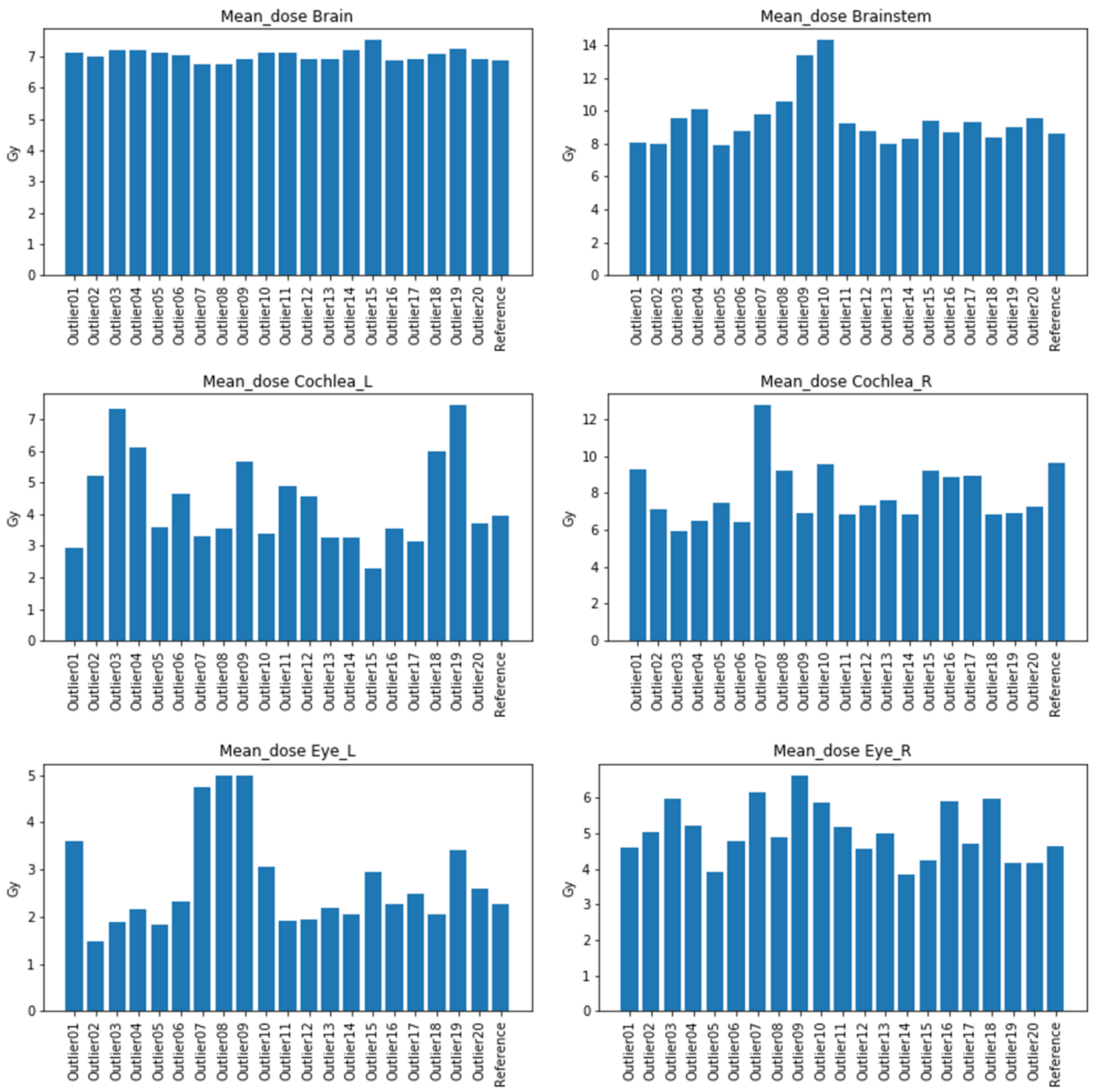

Figure 27: Bar plots of the mean dose of the brain, brainstem, cochlea and eyes, for the reference plan and the 20 plans including an outlier to the target at the specific location displayed in Figure 1.

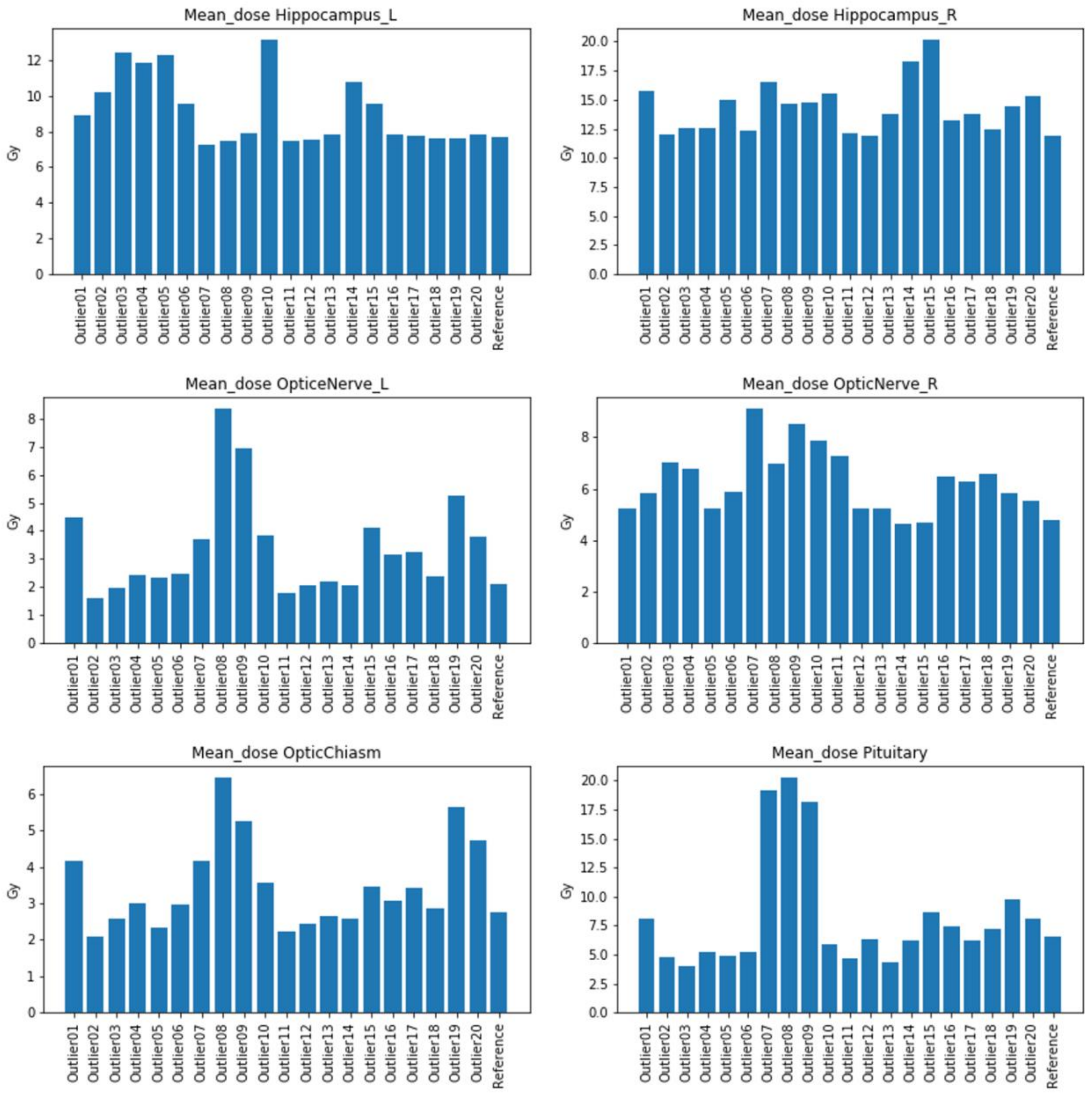

Figure 28: Bar plots of the mean dose of the hippocampi, optic nerves, optic chiasm and pituitary gland, for the reference plan and the 20 plans including an outlier to the target at the specific location displayed in Figure 1.

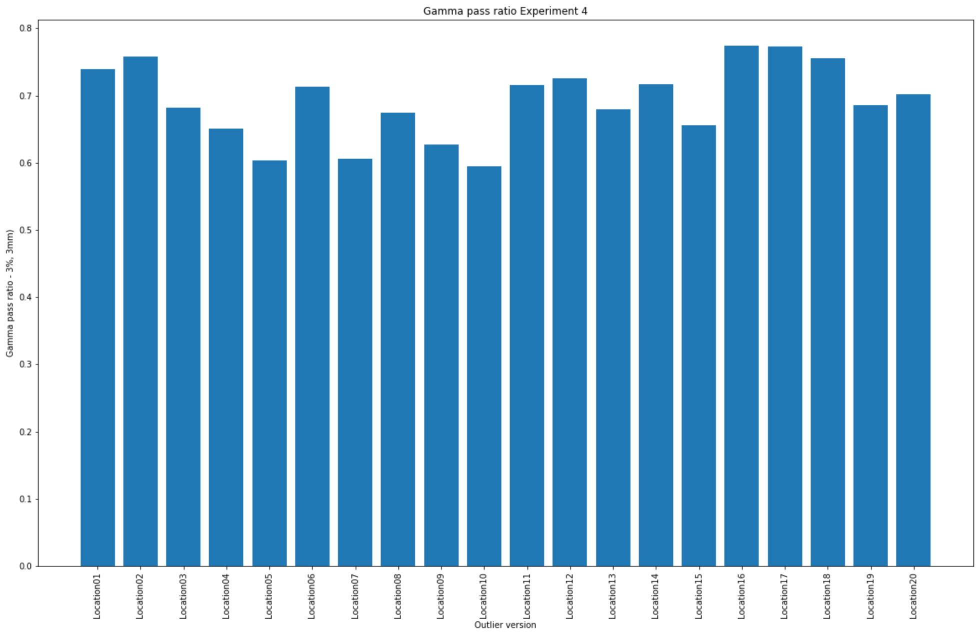

Figure 29: Bar plot of the Gamma pass ratio of each experimental plan containing an outlier at a specific location according to Figure 1 with respect to the reference plan. The criteria for the gamma pass rate were set to 3% and 3 mm.

Experiment 2: Outlier shape, no OARs involved:

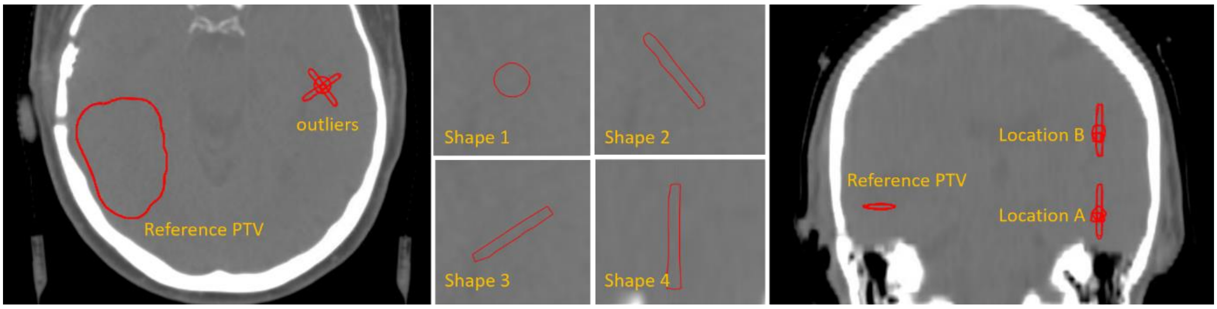

Figure 30: Overview of the synthetic setup of experiment 2. Left: Axial slice through the reference PTV and the lower location (A) of the 4 different shapes of outliers. Middle: Details of the outlier shapes and orientation. It should be noted that shape 4 is positioned in the cranial-caudal direction. Right: The difference of the axial range of locations A and B can be seen.

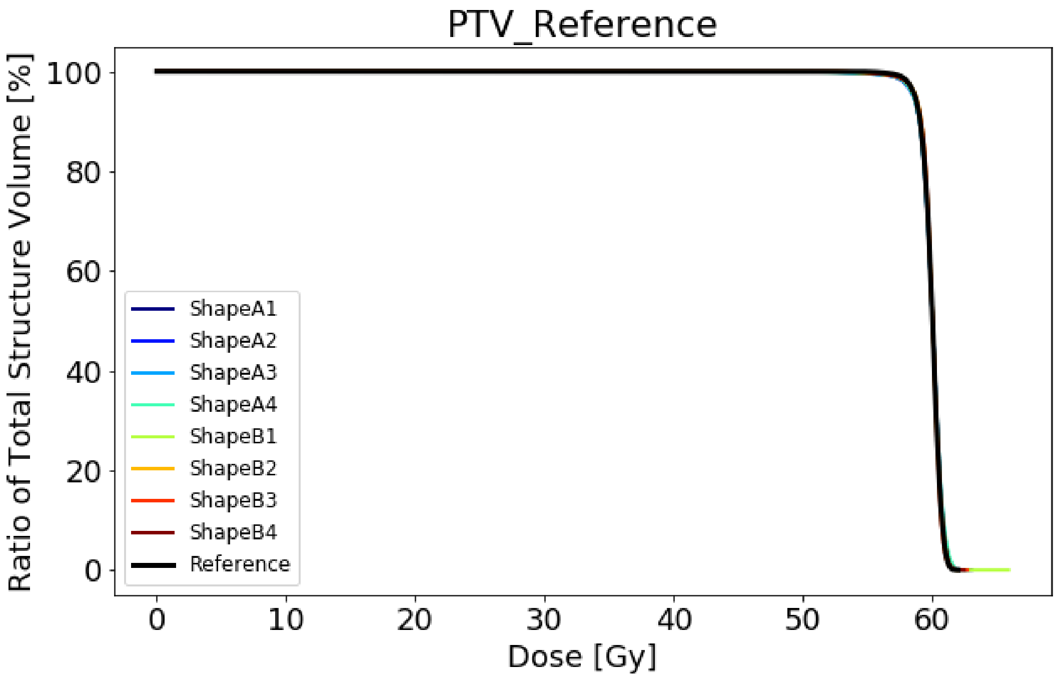

Figure 31: DVH curves of PTV structure of the reference plan and the 8 plans including an outlier to the target at the specific location ans shapes as displayed in Figure 30.

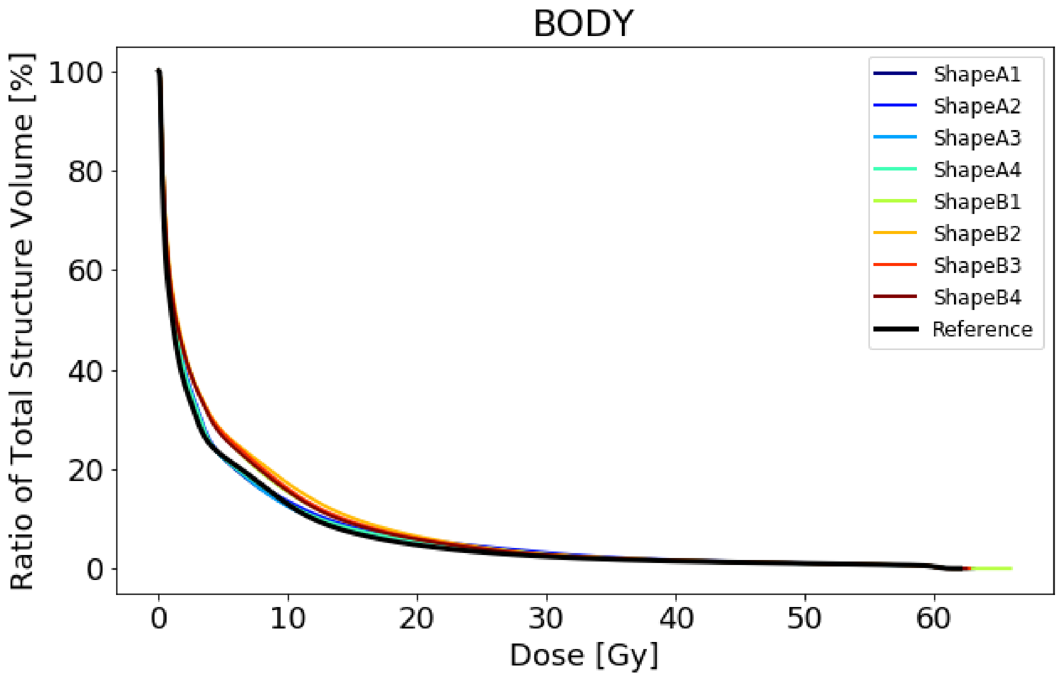

Figure 32: DVH curves of Bod structure of the reference plan and the 8 plans including an outlier to the target at the specific location ans shapes as displayed in Figure 30.

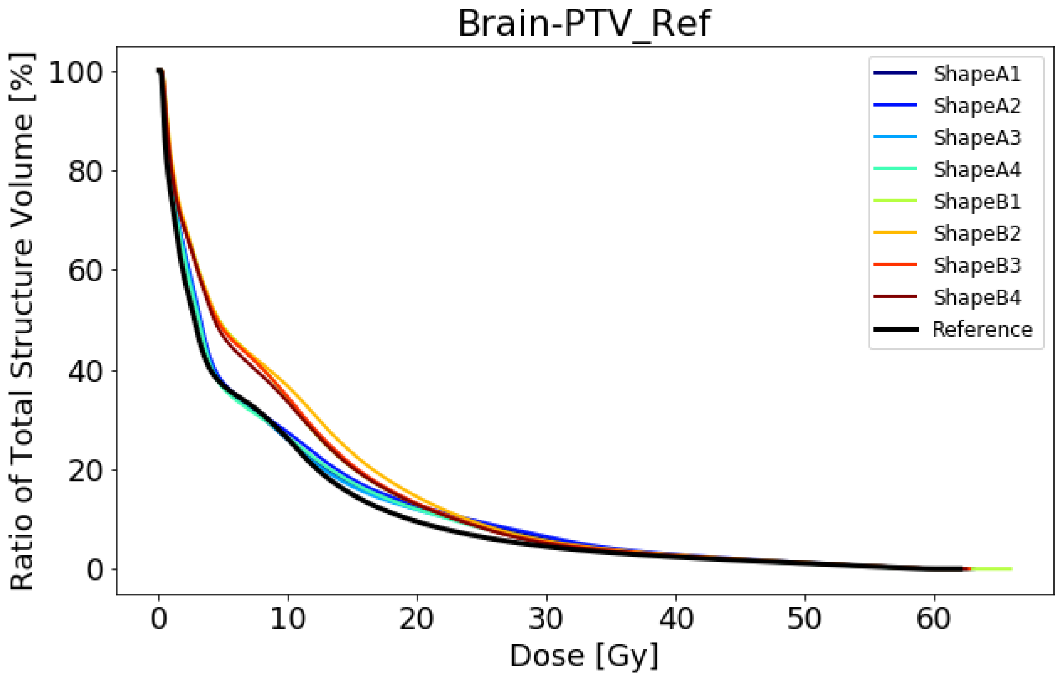

Figure 33: DVH curves of healthy brain structure (i.e. brain minus PTV) of the reference plan and the 8 plans including an outlier to the target at the specific location and shapes as displayed in Figure 30.

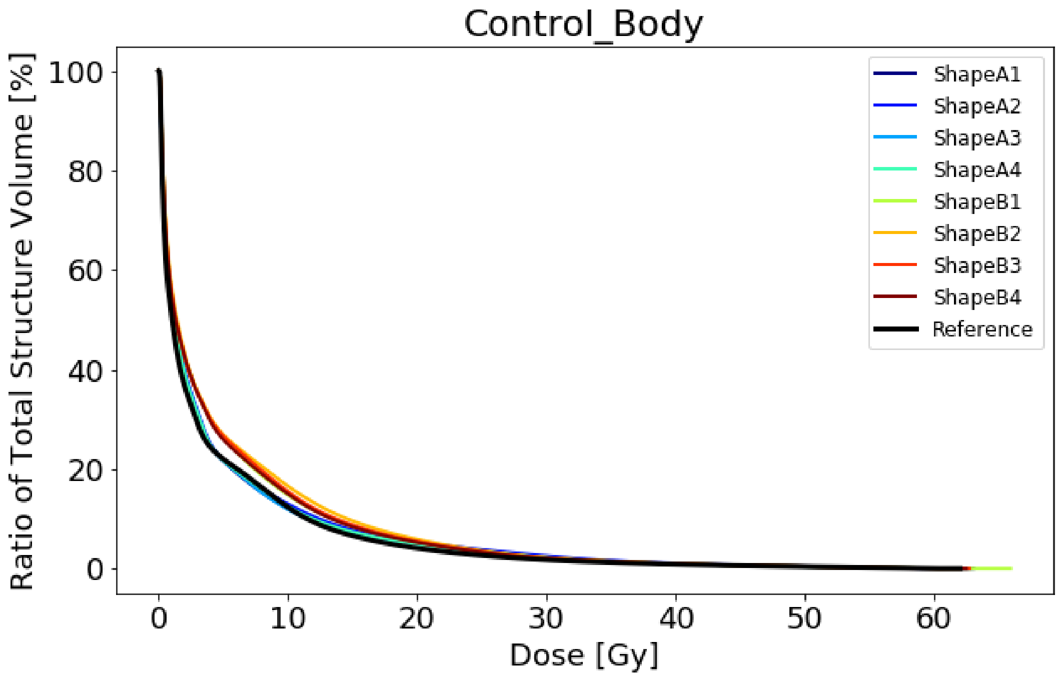

Figure 34: DVH curves of Control Body structure (i.e. body minus PTV) of the reference plan and the 8 plans including an outlier to the target at the specific location and shapes as displayed in Figure 30.

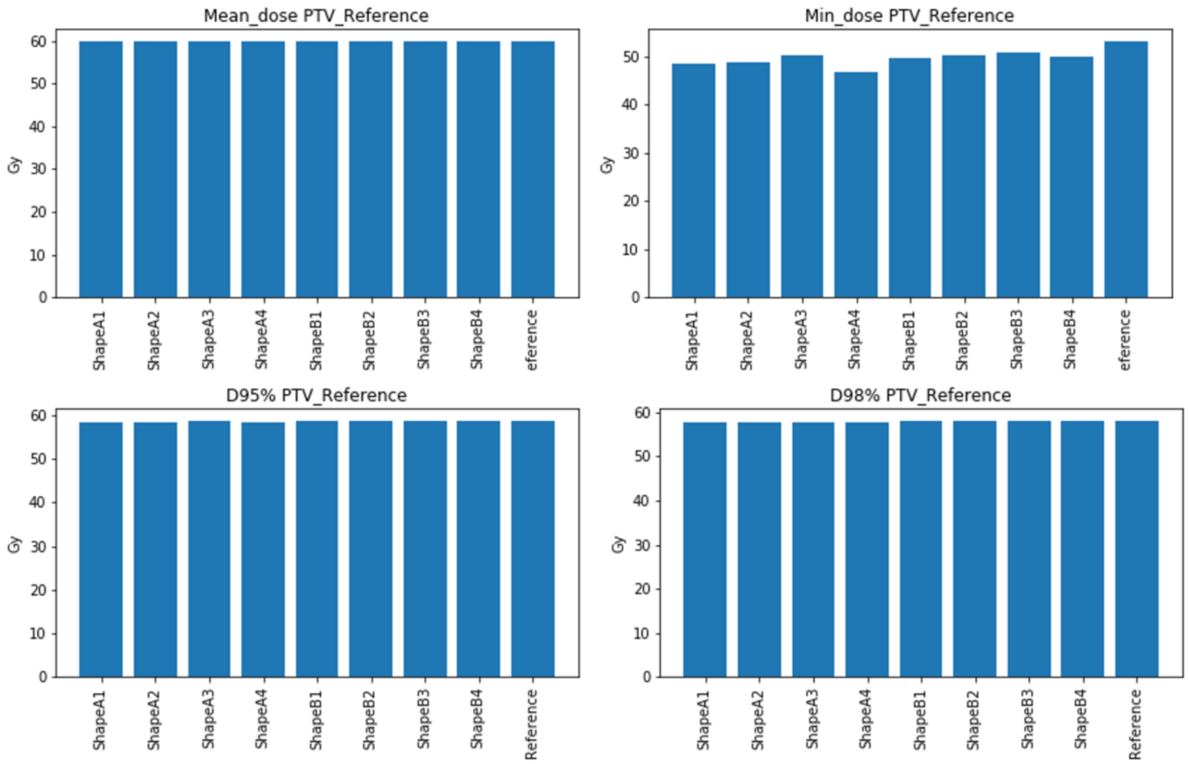

Figure 35: Bar plots of the mean dose, min dose and the 98% and 95% coverage of the PTV for the reference plan and the 8 plans including an outlier to the target at the specific location and shapes as displayed in Figure 30.

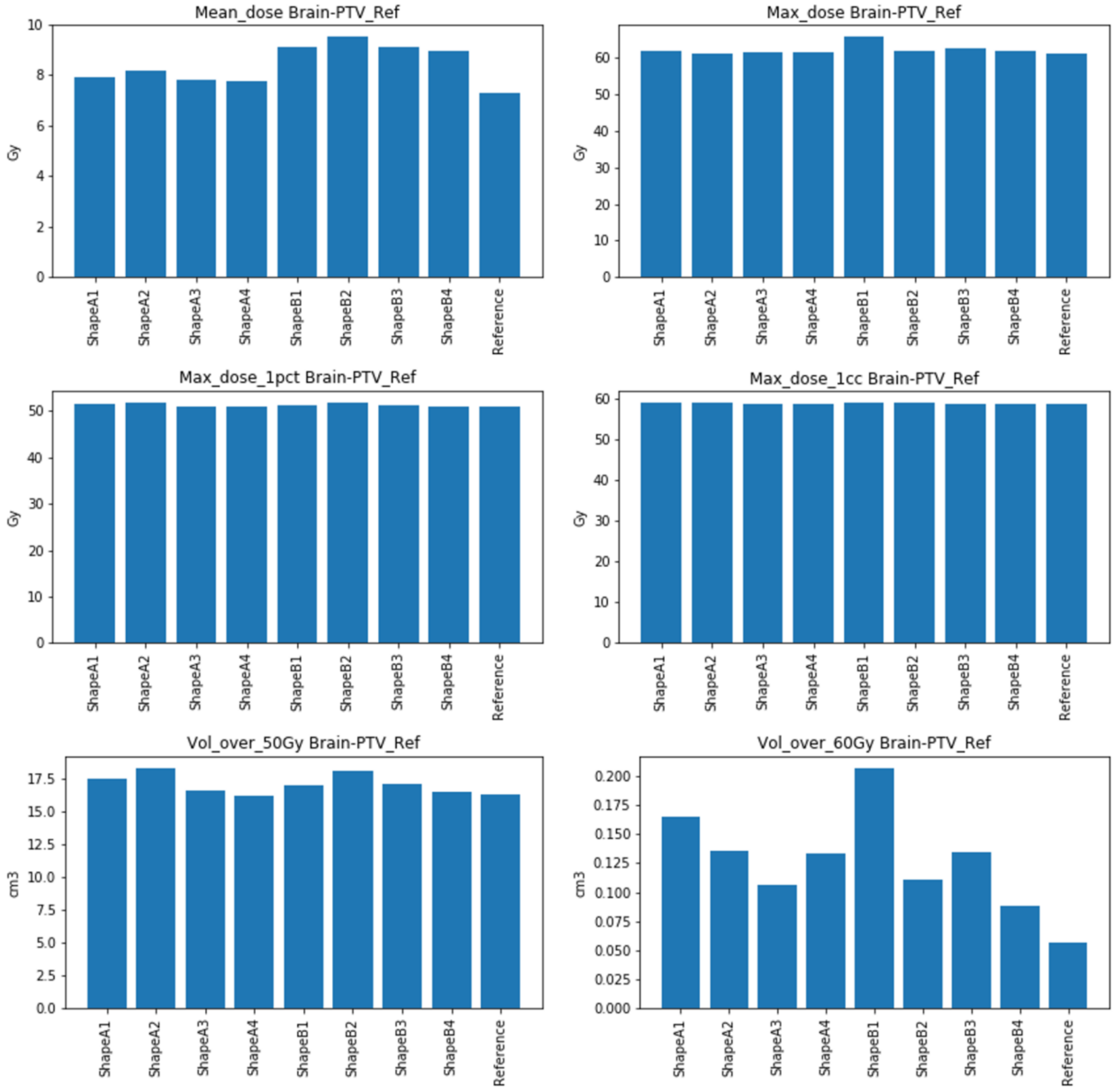

Figure 36: Bar plots of the mean dose, max dose, max dose to 1% of the volume, max dose to 1 cc of the volume, the volume receiving over 50Gy and the volume receiving over 60 Gy of the Healthy brain structure (Brain minus PTV), for the reference plan and the 8 plans including an outlier to the target at the specific location and shapes as displayed in Figure 30.

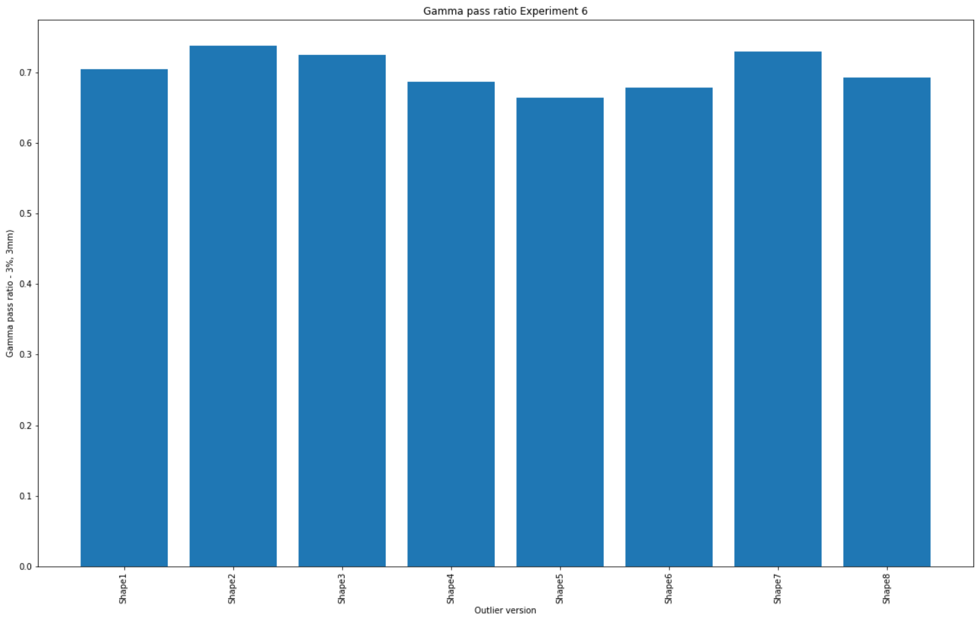

Figure 37: Bar plot of the Gamma pass ratio of each experimental plan containing an outlier at a specific location and shape according to Figure 30 with respect to the reference plan. The criteria for the gamma pass rate were set to 3% and 3 mm.

Experiment 2: Outlier shape, with OARs involved:

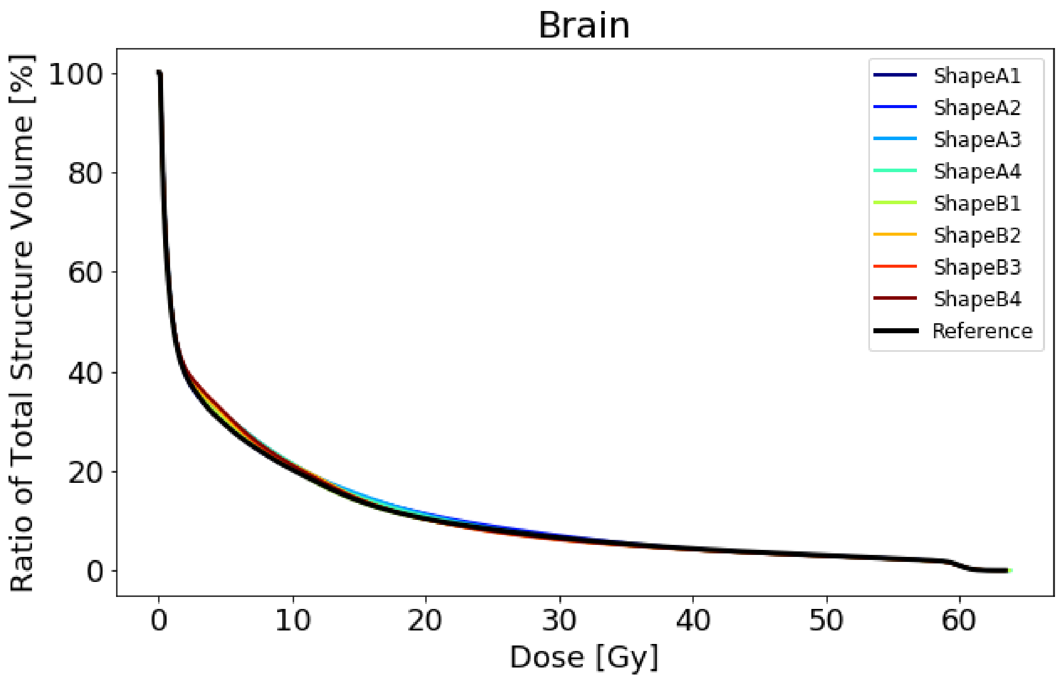

Figure 38: DVH curves of the brain of the reference plan and the 8 plans including an outlier to the target at the specific location and shape as displayed in Figure 30.

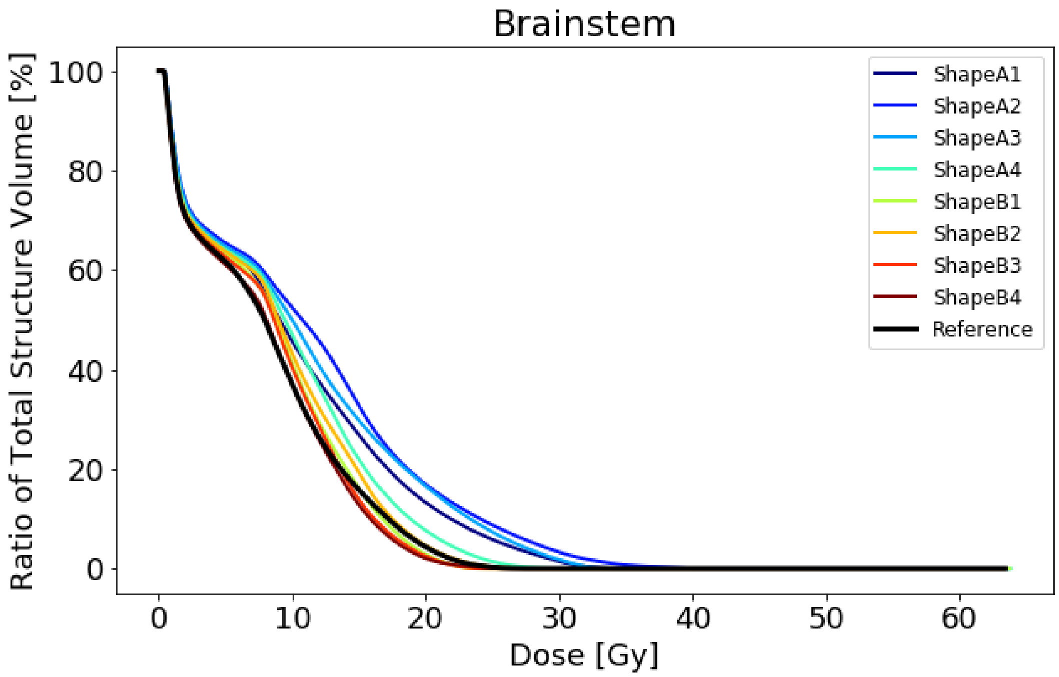

Figure 39: DVH curves of the brainstem of the reference plan and the 8 plans including an outlier to the target at the specific location and shape as displayed in Figure 30.

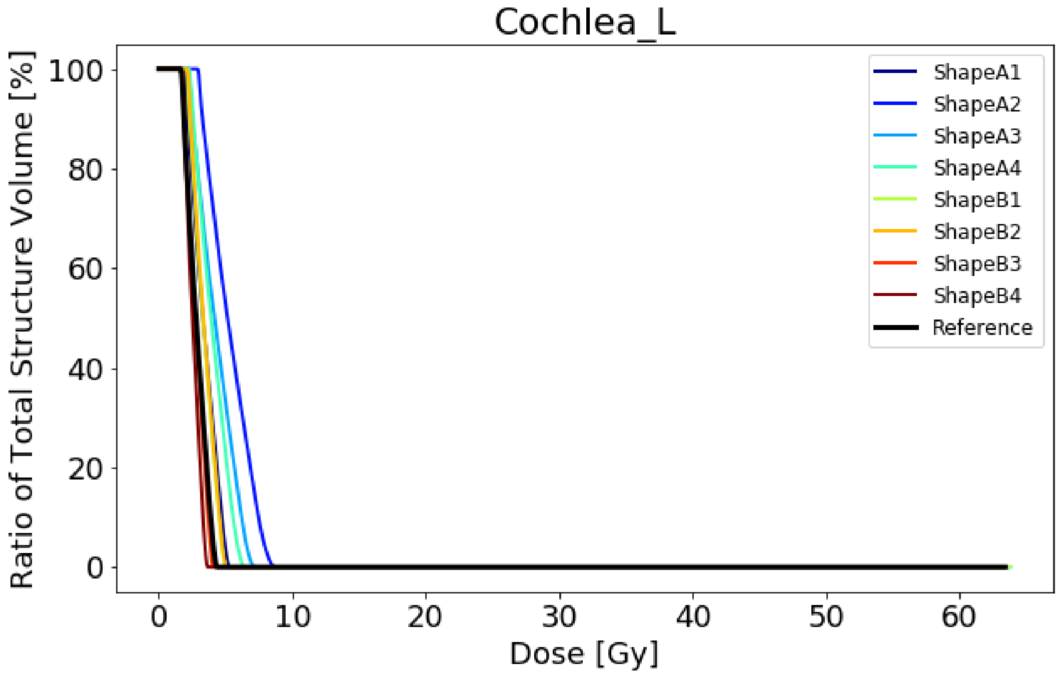

Figure 40: DVH curves of the left cochlea of the reference plan and the 8 plans including an outlier to the target at the specific location and shape as displayed in Figure 30.

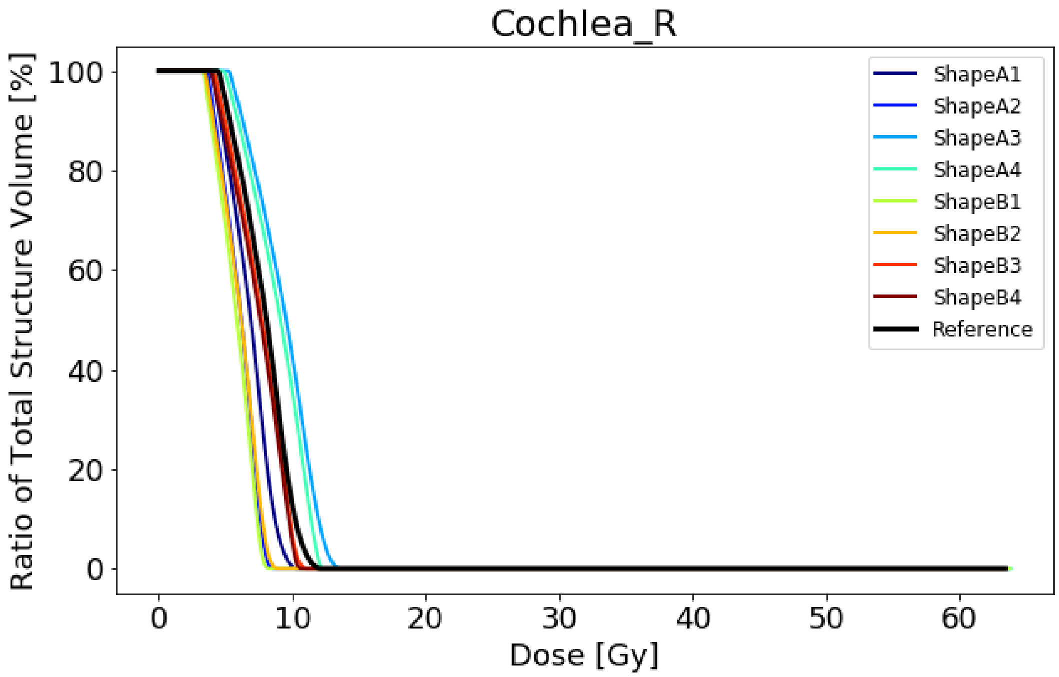

Figure 41: DVH curves of the right cochlea of the reference plan and the 8 plans including an outlier to the target at the specific location and shape as displayed in Figure 30.

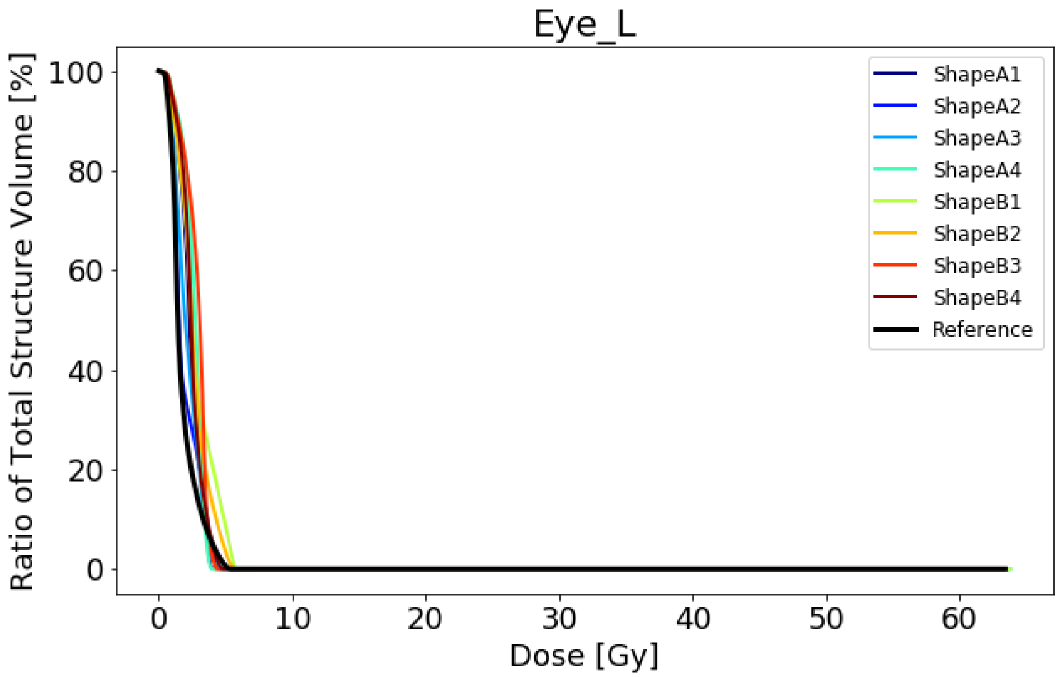

Figure 42: DVH curves of the left eye of the reference plan and the 8 plans including an outlier to the target at the specific location and shape as displayed in Figure 30.

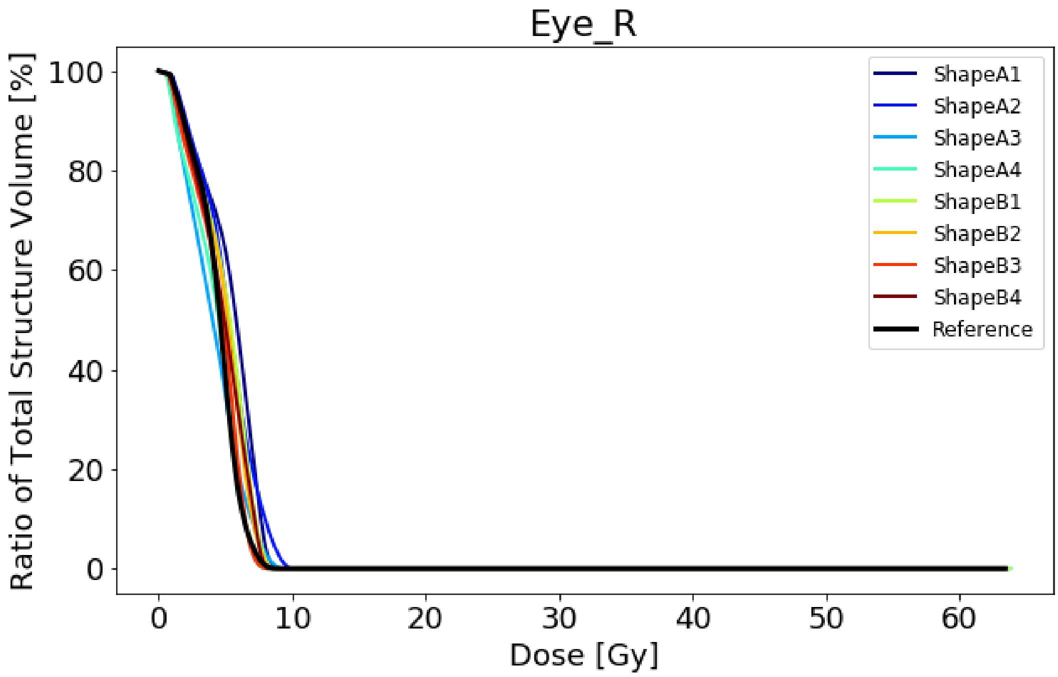

Figure 43: DVH curves of the right eye of the reference plan and the 8 plans including an outlier to the target at the specific location and shape as displayed in Figure 30.

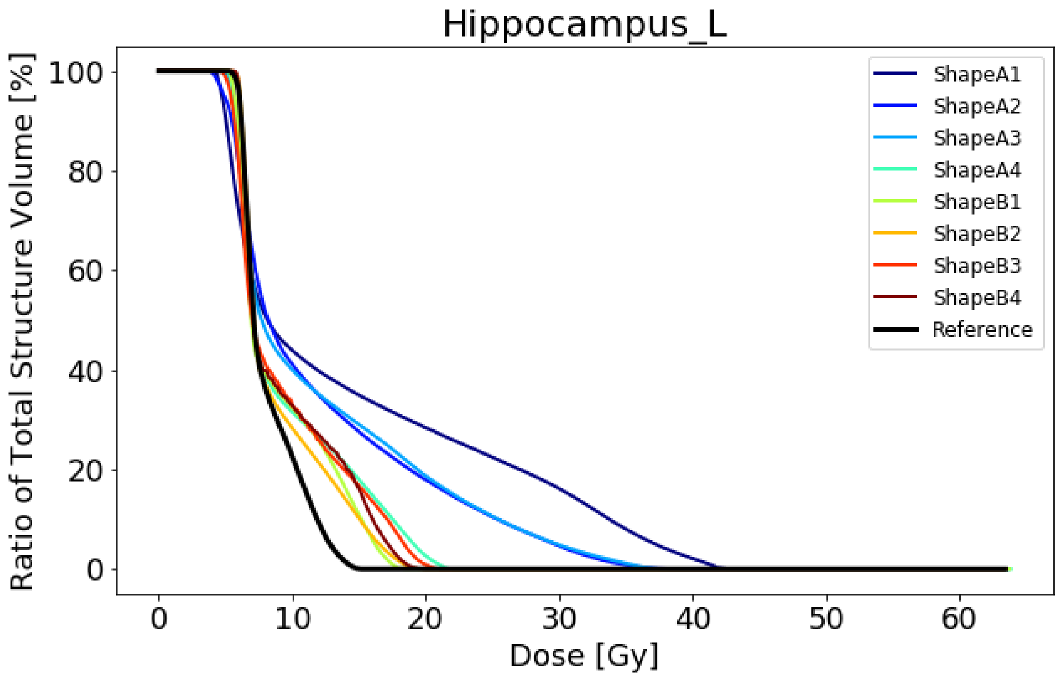

Figure 44: DVH curves of the left hippocampus of the reference plan and the 8 plans including an outlier to the target at the specific location and shape as displayed in Figure 30.

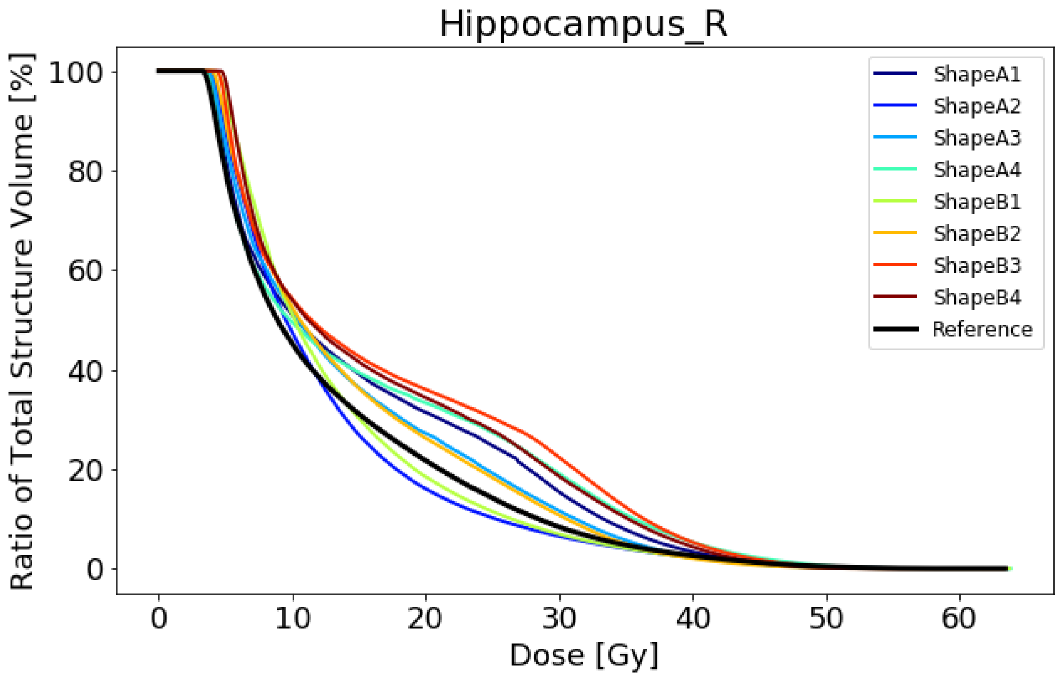

Figure 45: DVH curves of the right hippocampus of the reference plan and the 8 plans including an outlier to the target at the specific location and shape as displayed in Figure 30.

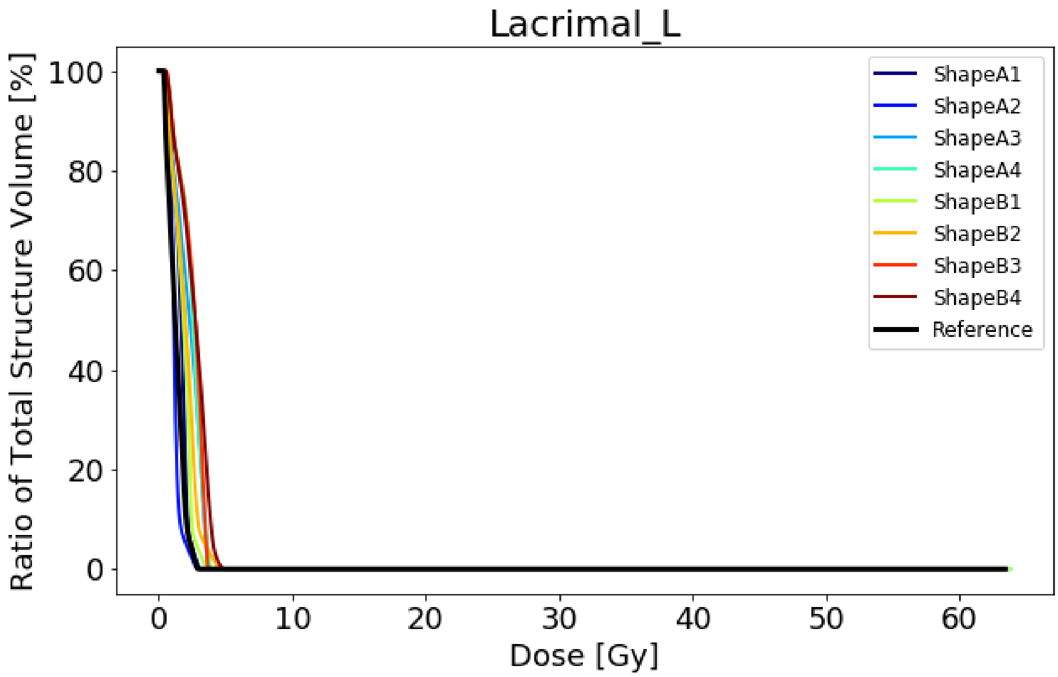

Figure 46: DVH curves of the left lacrimal gland of the reference plan and the 8 plans including an outlier to the target at the specific location and shape as displayed in Figure 30.

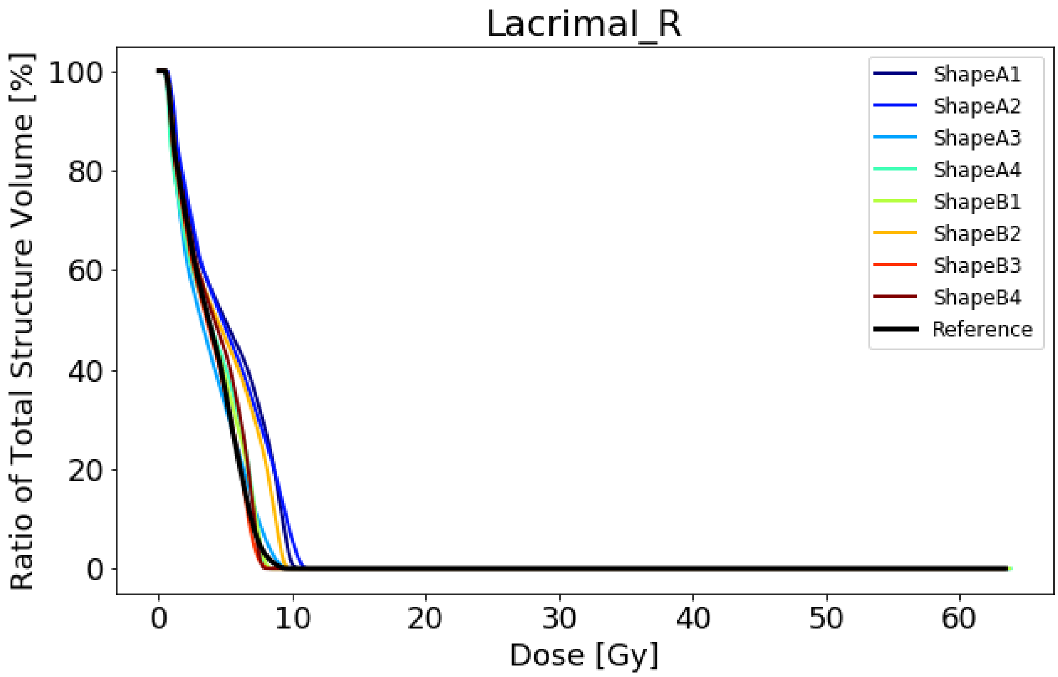

Figure 47: DVH curves of the right lacrimal gland of the reference plan and the 8 plans including an outlier to the target at the specific location and shape as displayed in Figure 30.

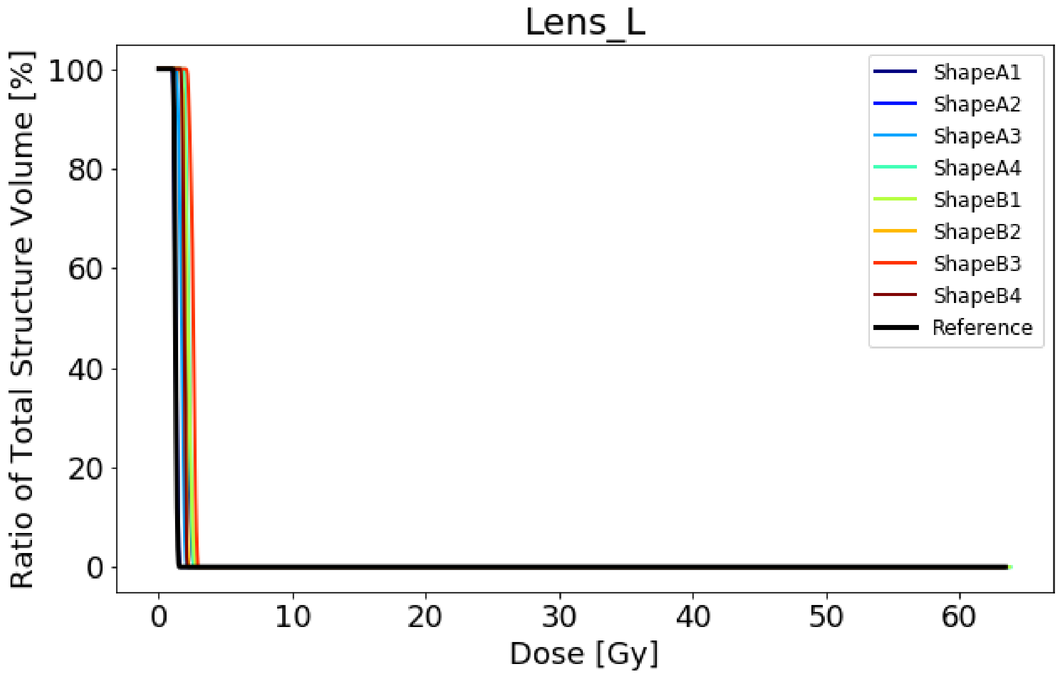

Figure 48: DVH curves of the left lens of the reference plan and the 8 plans including an outlier to the target at the specific location and shape as displayed in Figure 30.

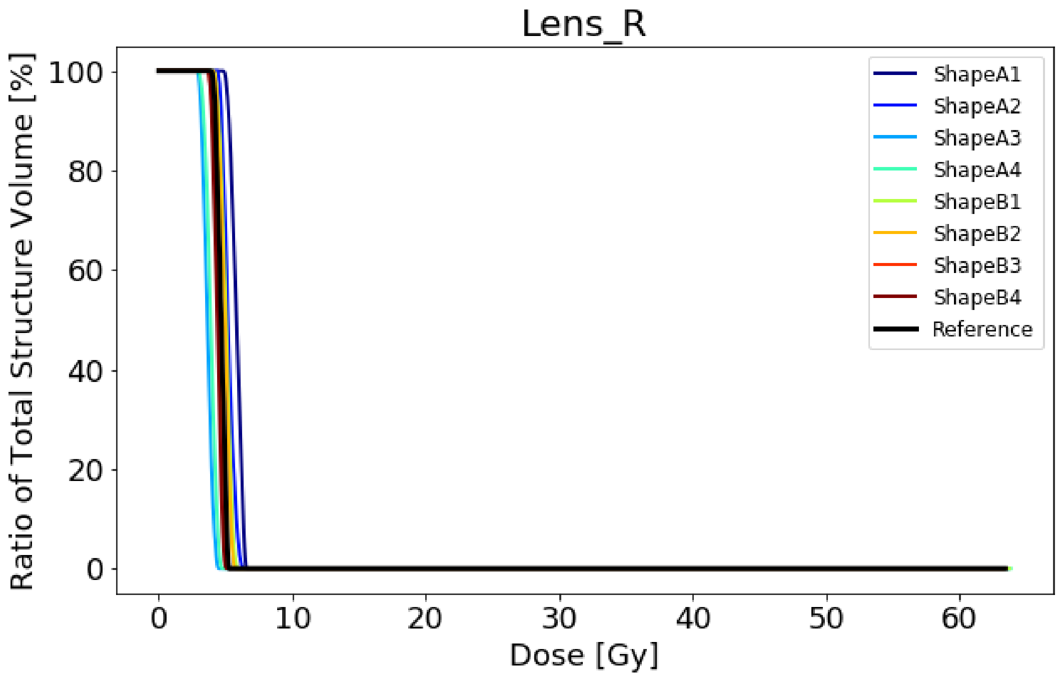

Figure 49: DVH curves of the right lens of the reference plan and the 8 plans including an outlier to the target at the specific location and shape as displayed in Figure 30.

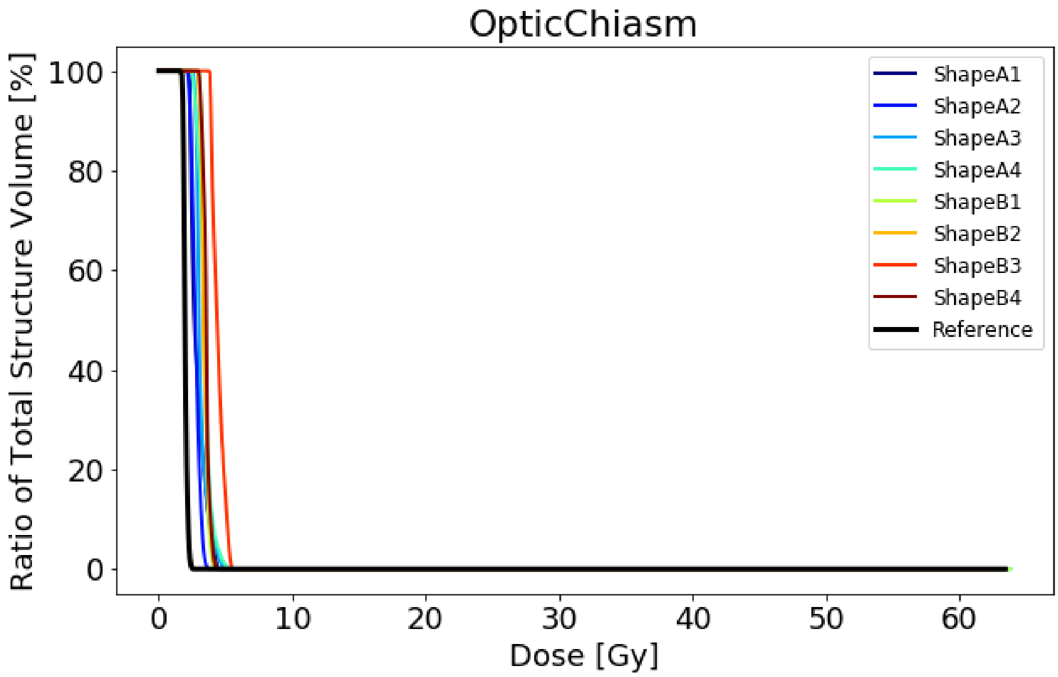

Figure 50: DVH curves of the optic chiasm of the reference plan and the 8 plans including an outlier to the target at the specific location and shape as displayed in Figure 30.

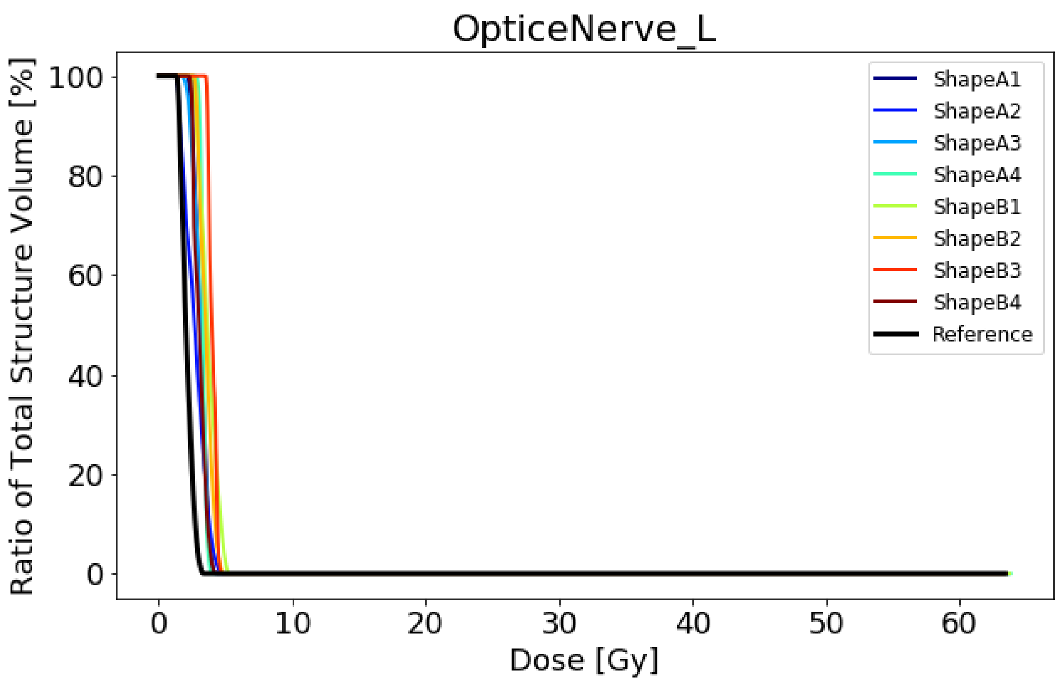

Figure 51: DVH curves of the left optic nerve of the reference plan and the 8 plans including an outlier to the target at the specific location and shape as displayed in Figure 30.

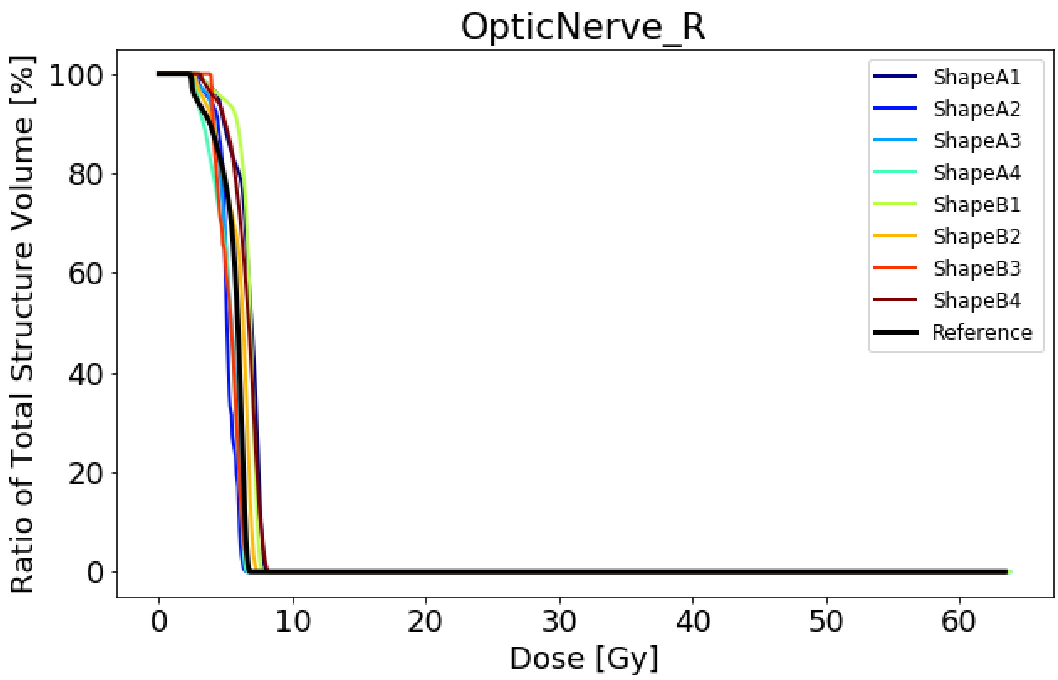

Figure 52: DVH curves of the right optic nerve of the reference plan and the 8 plans including an outlier to the target at the specific location and shape as displayed in Figure 30.

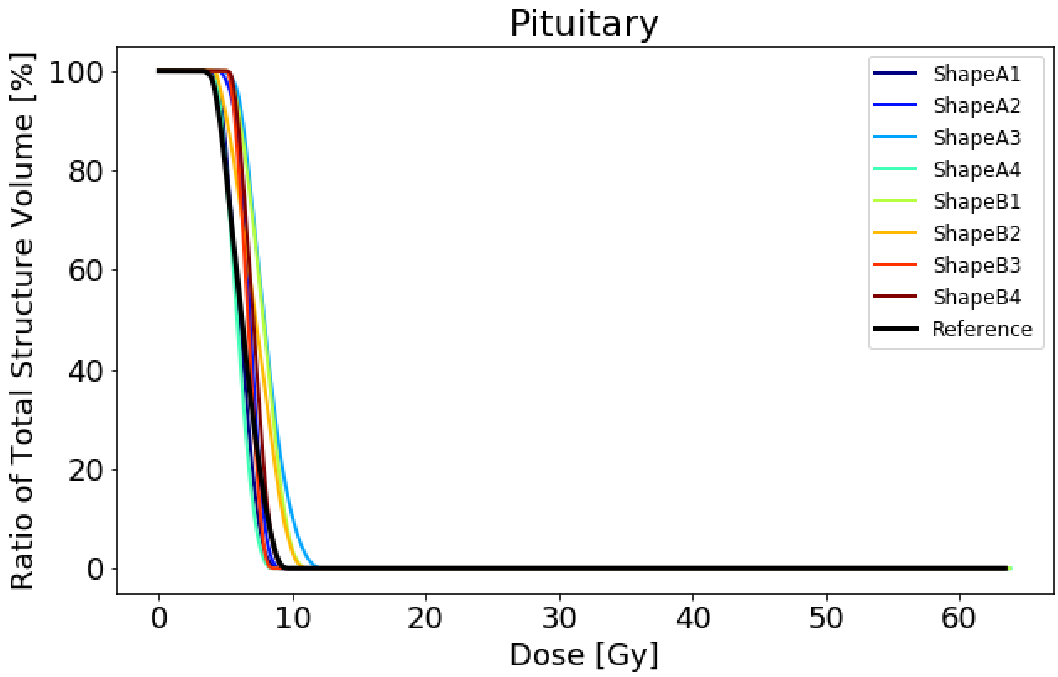

Figure 53: DVH curves of the pituitary gland of the reference plan and the 8 plans including an outlier to the target at the specific location and shape as displayed in Figure 30.

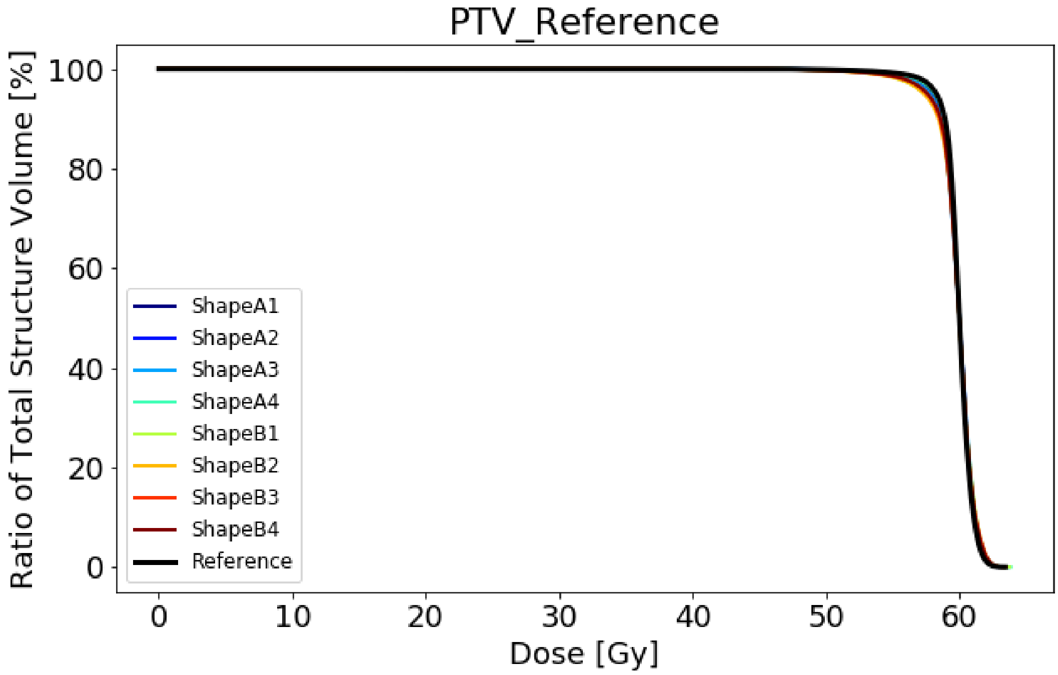

Figure 54: DVH curves of the PTV of the reference plan and the 8 plans including an outlier to the target at the specific location and shape as displayed in Figure 30.

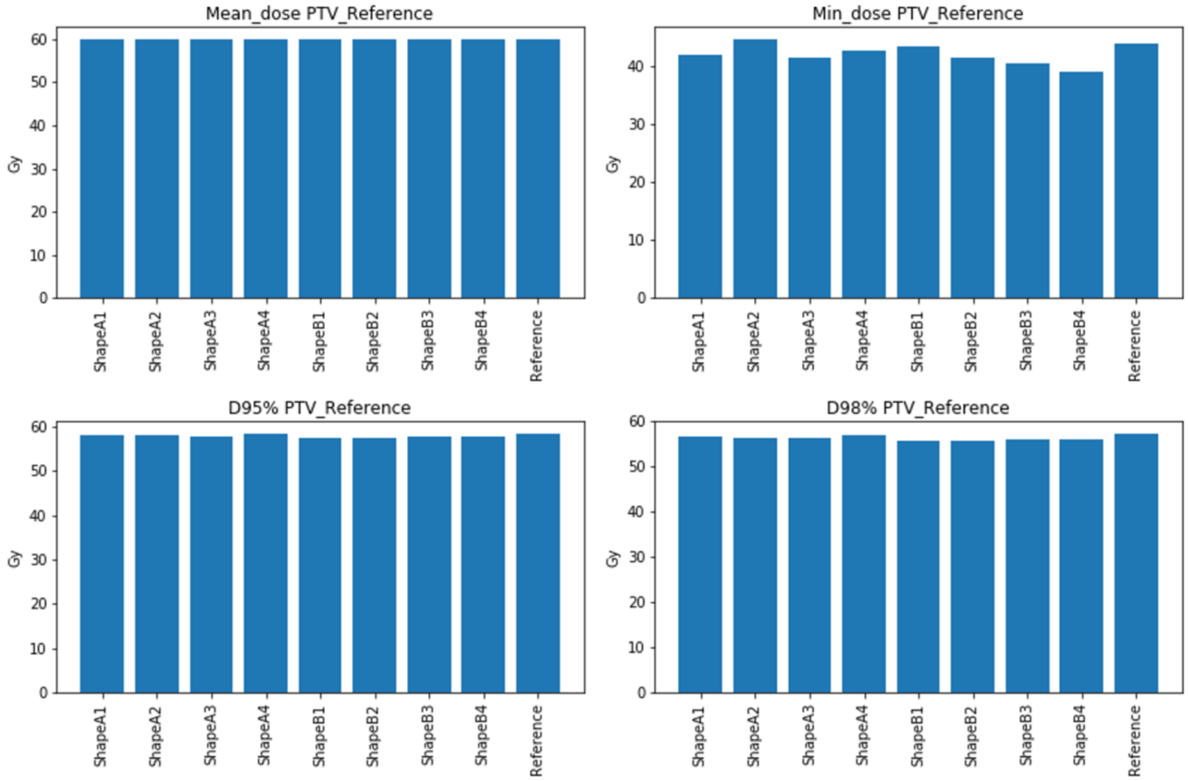

Figure 55: Bar plots of the mean dose, min dose and the 98% and 95% coverage of the PTV for the reference plan and the 8 plans including an outlier to the target at the specific location and shape displayed in Figure 30.

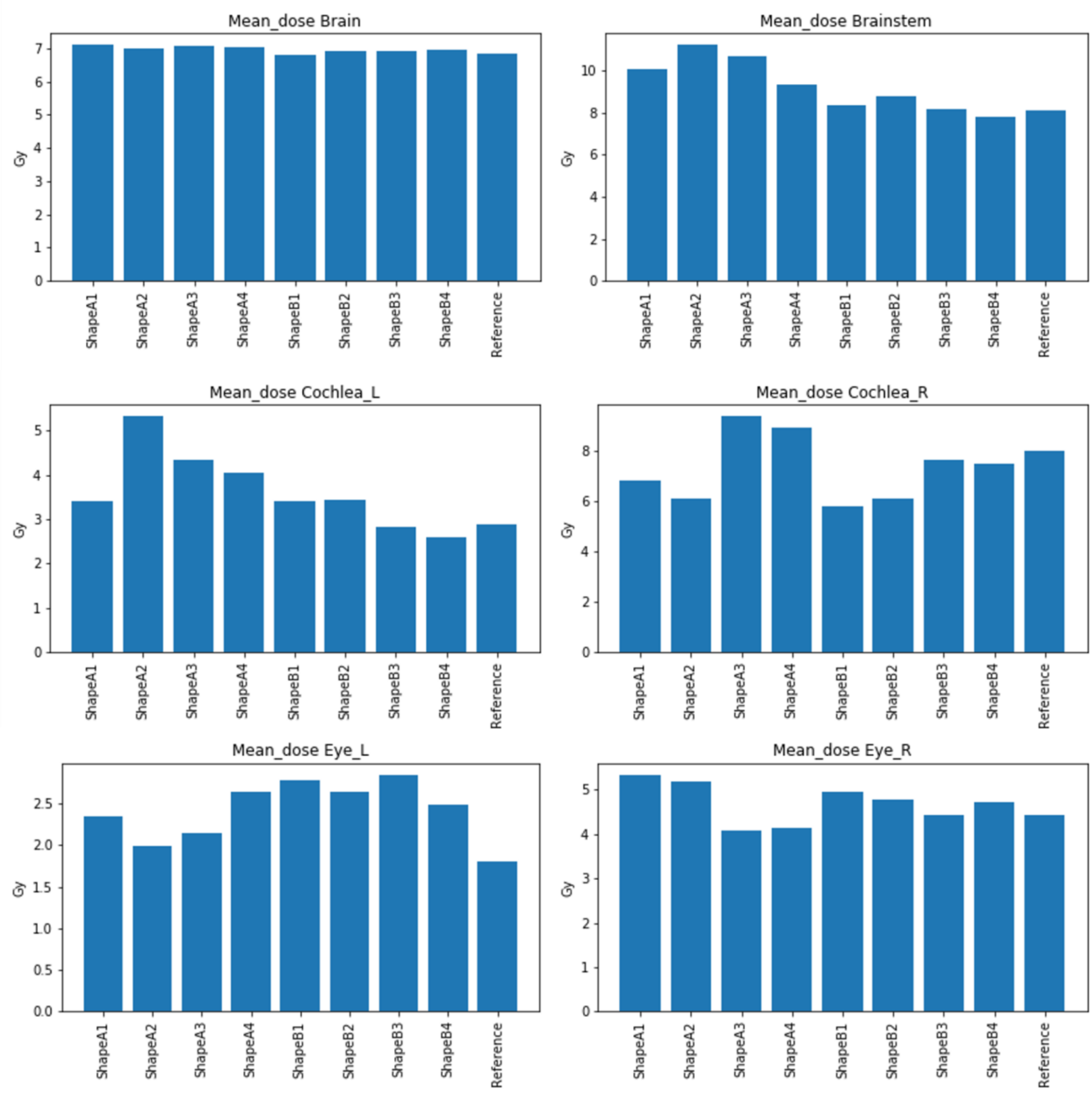

Figure 56: Bar plots of the mean dose of the brain, brainstem, cochlea and eyes, for the reference plan and the 8 plans including an outlier to the target at the specific location and shape displayed in Figure 30.

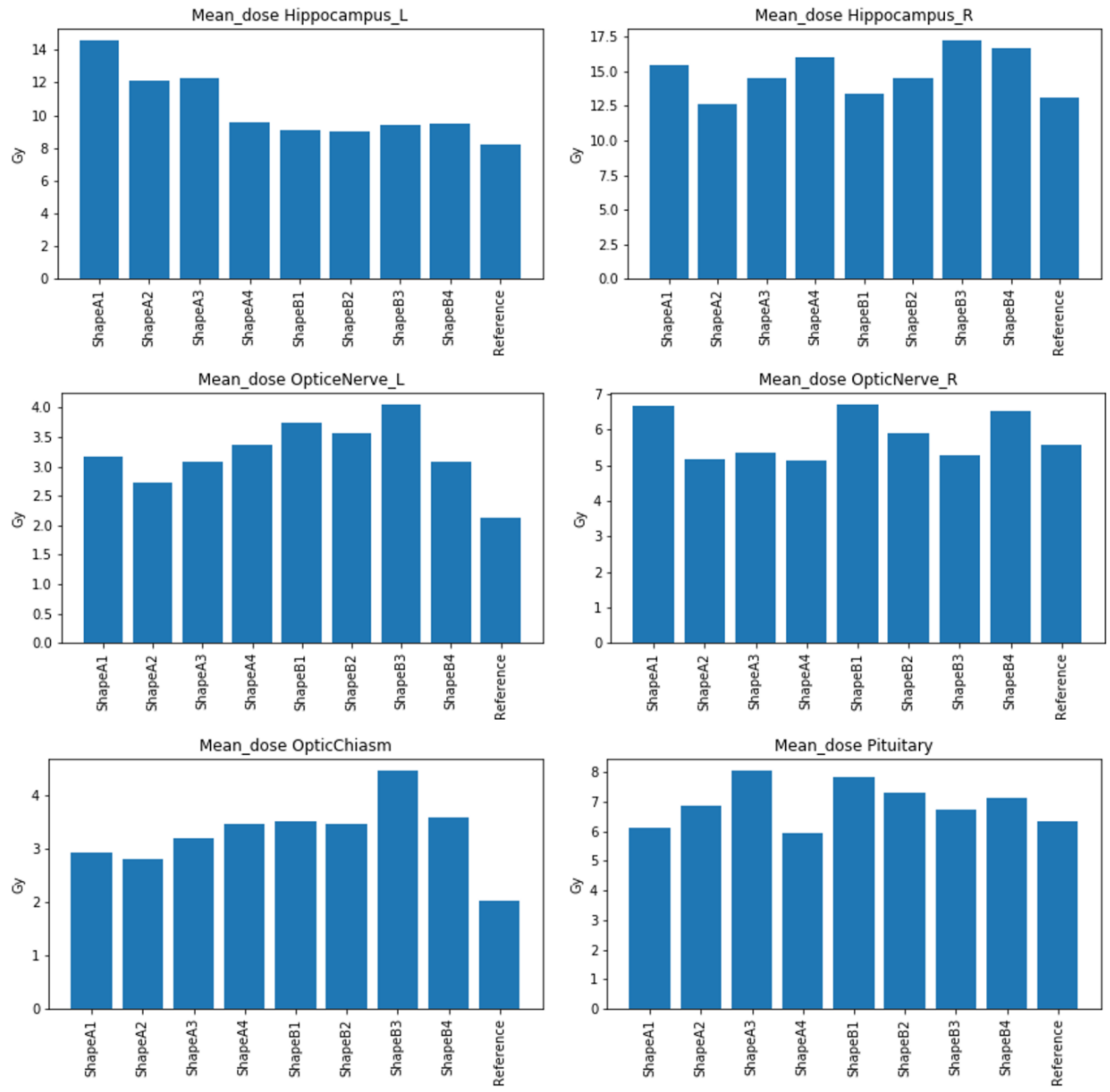

Figure 57: Bar plots of the mean dose of the hippocampi, optic nerves, optic chiasm and pituitary, for the reference plan and the 8 plans including an outlier to the target at the specific location and shape displayed in Figure 30.

Experiment 3: Outlier size, no OARs involved:

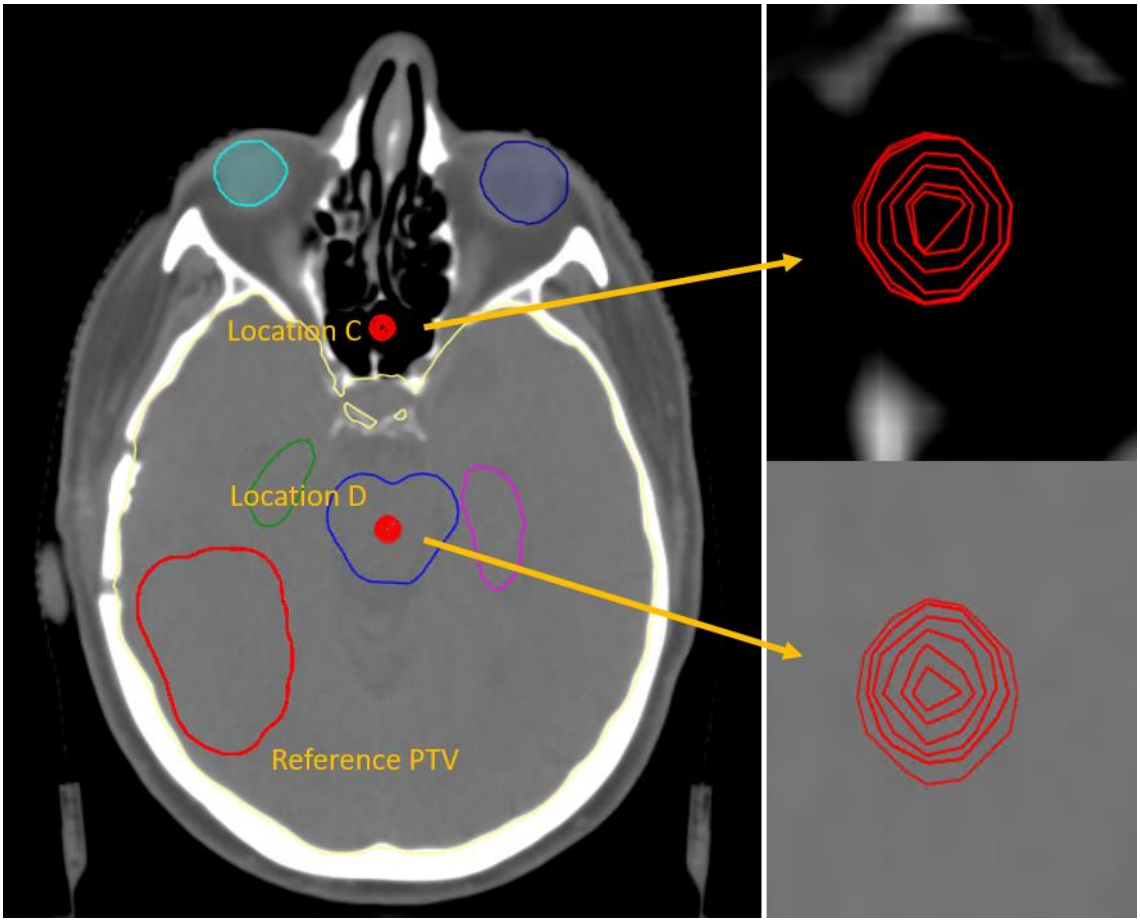

Figure 58: Overview of synthetic setup of experiment 3 to determine the effect of outlier size on dosimetry. In red the reference PTV and two locations, C and D of outlier volumes are represented. In both locations 12 outliers of gradually increasing size were generated. Right: Close up view of the outlier volumes.

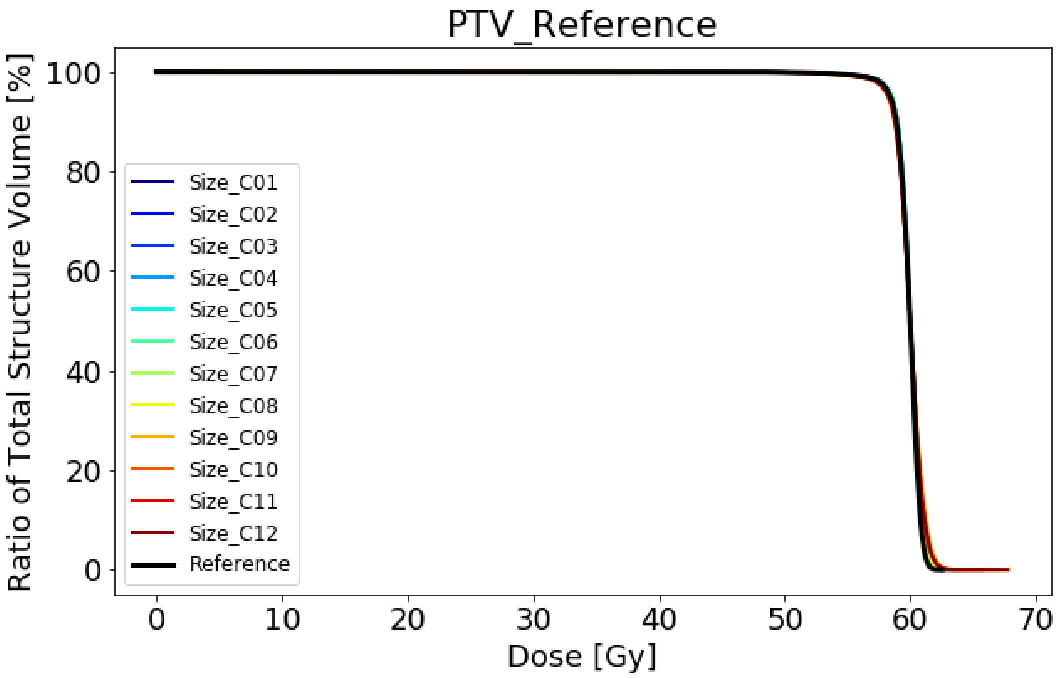

Figure 59: DVH curves of PTV structure of the reference plan and the 12 plans including an outlier to the target at of specific size at location C as displayed in Figure 58.

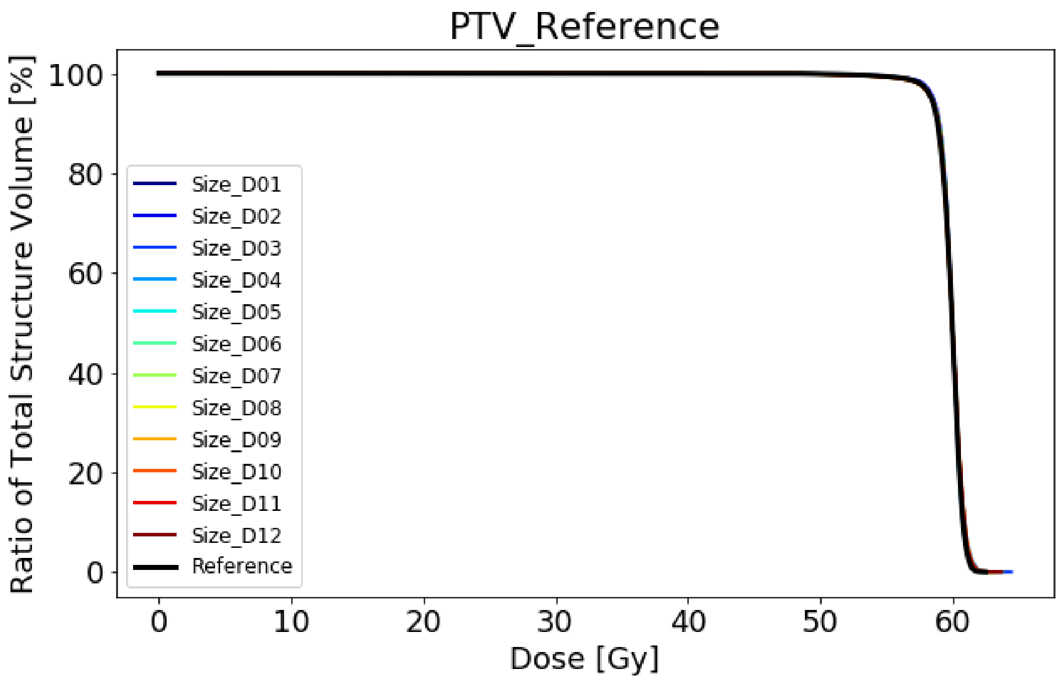

Figure 60: DVH curves of PTV structure of the reference plan and the 12 plans including an outlier to the target at of specific size at location D as displayed in Figure 58

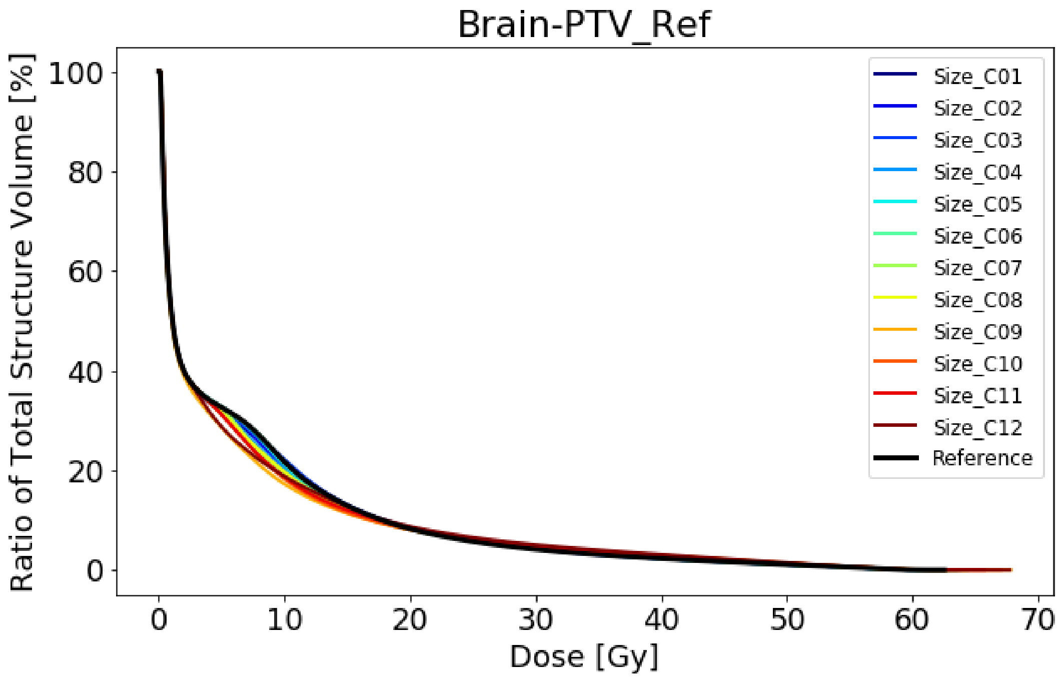

Figure 61: DVH curves of healthy brain structure (i.e. brain minus PTV) of the reference plan and the 12 plans including an outlier to the target at of specific size at location C as displayed in Figure 58

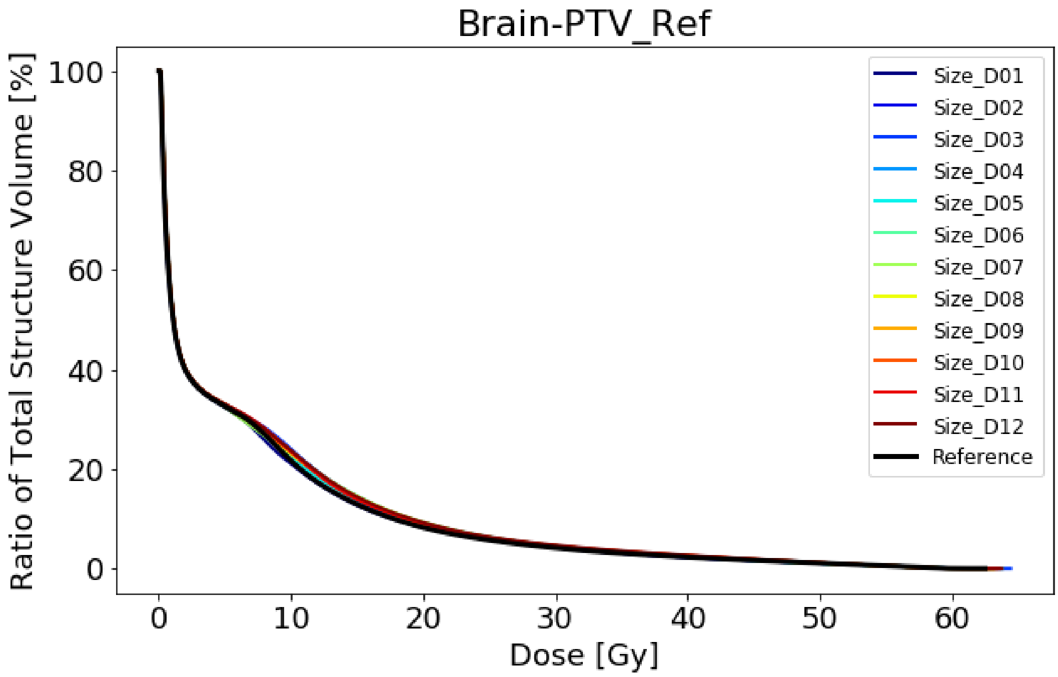

Figure 62: DVH curves of healthy brain structure (i.e. brain minus PTV) of the reference plan and the 12 plans including an outlier to the target at of specific size at location D as displayed in Figure 58

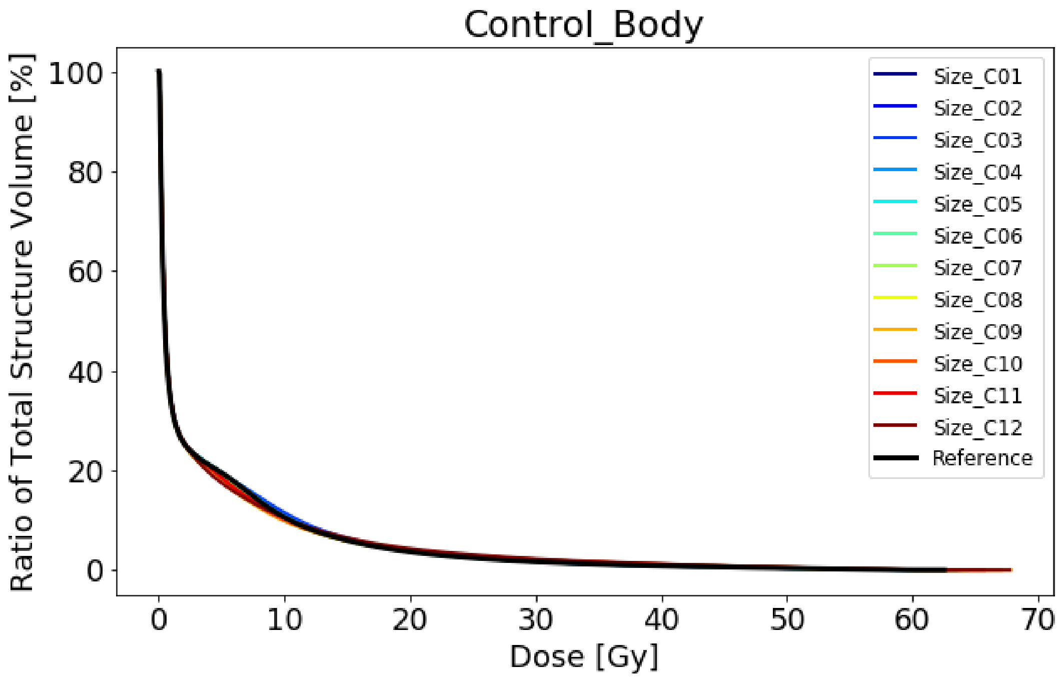

Figure 63: DVH curves of the control body structure (i.e. body minus PTV) of the reference plan and the 12 plans including an outlier to the target at of specific size at location C as displayed in Figure 58.

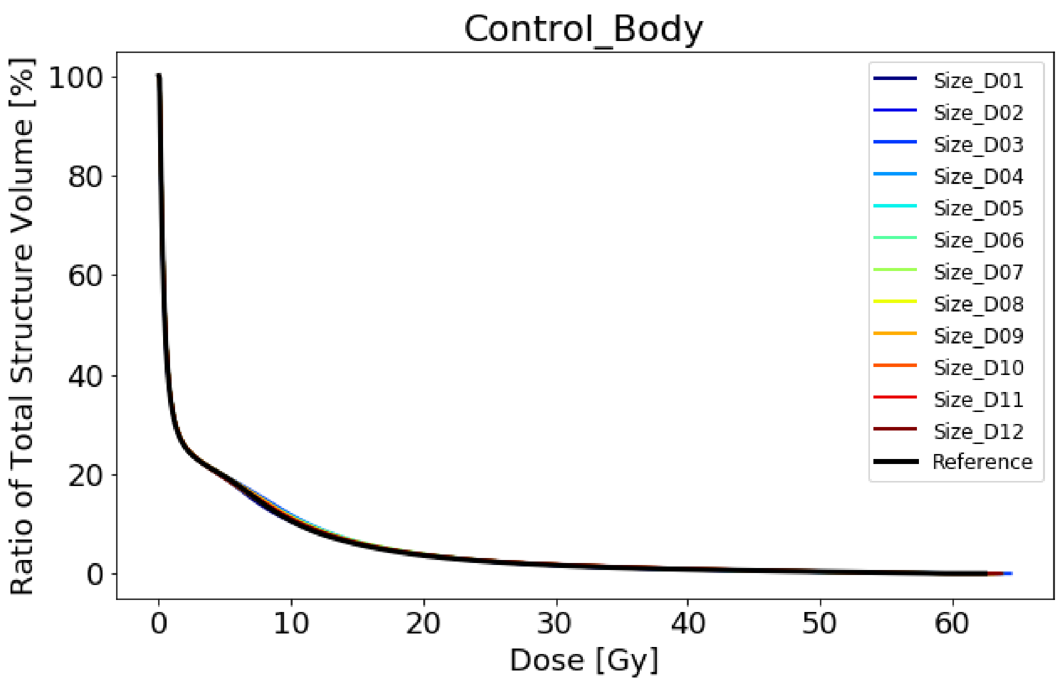

Figure 64: DVH curves of the control body structure (i.e. body minus PTV) of the reference plan and the 12 plans including an outlier to the target at of specific size at location D as displayed in Figure 58.

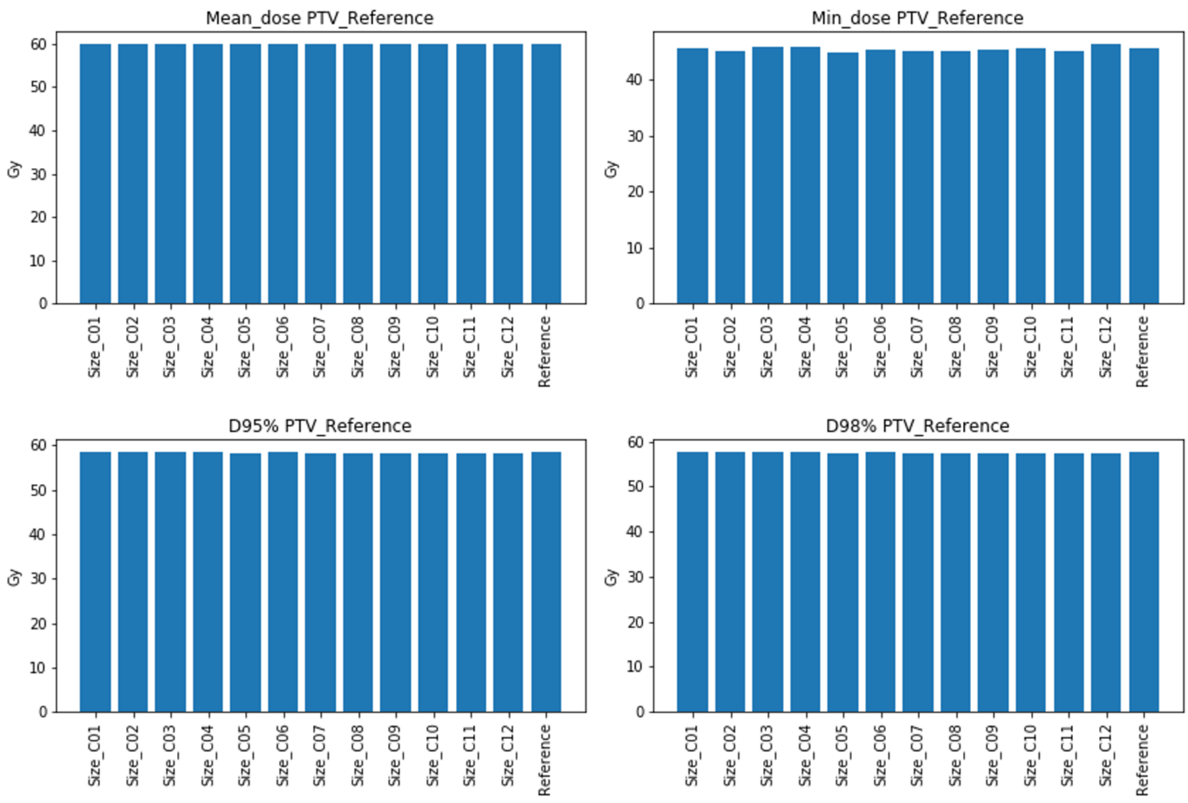

Figure 65: Bar plots of the mean dose, min dose and the 95% and 98% coverage of the PTV for the reference plan and the 12 plans including an outlier to the target at the specific sizes at location C as displayed in Figure 58.

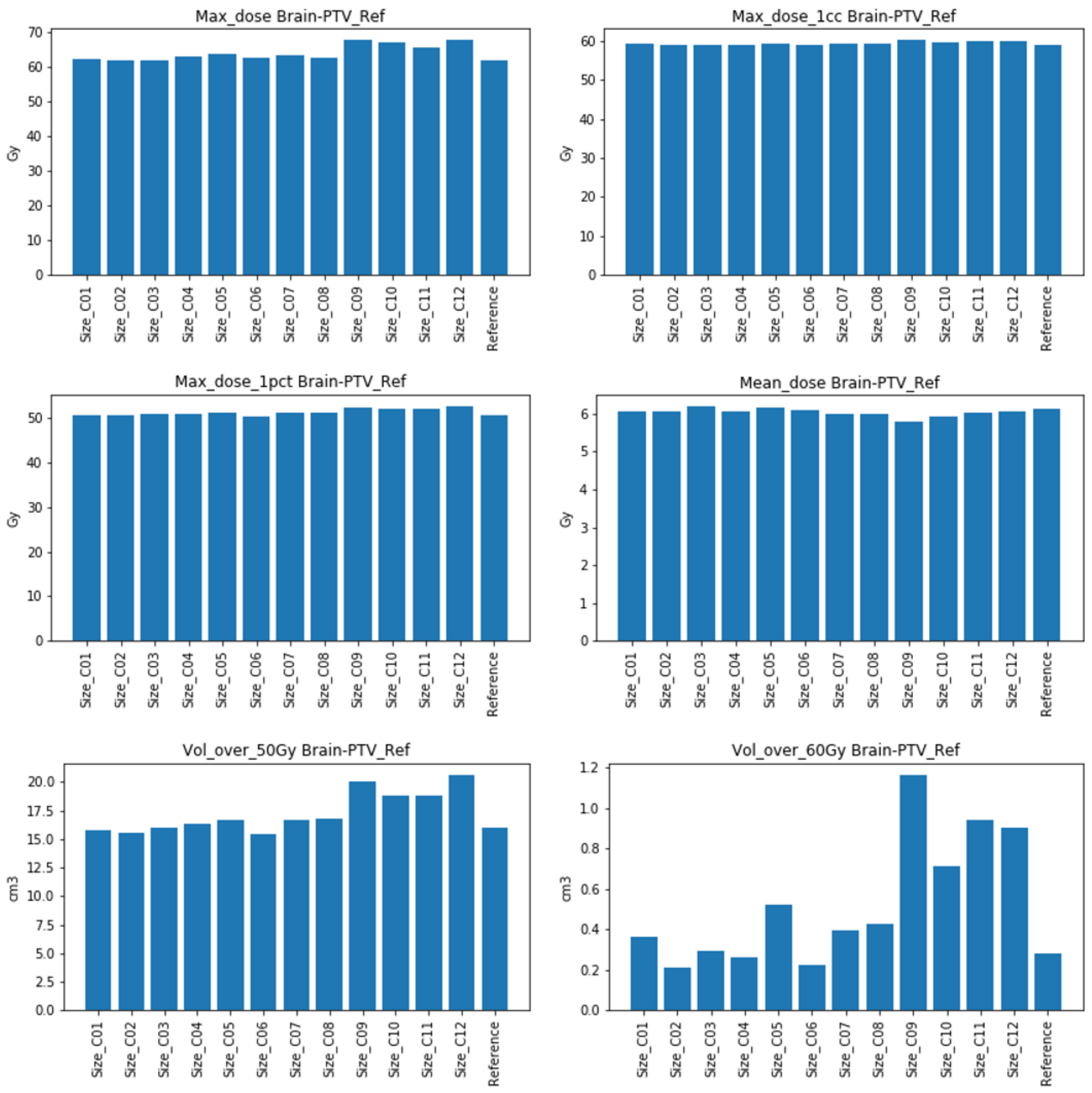

Figure 66: Bar plots of the max dose, mean dose, maximum dose to 1% of the volume, maximum dose to 1cc of the volume, the volume receiving over 50Gy and the volume receiving over 60 Gy to the healthy brain for the reference plan and the 12 plans including an outlier to the target at the specific sizes at location C as displayed in Figure 58.

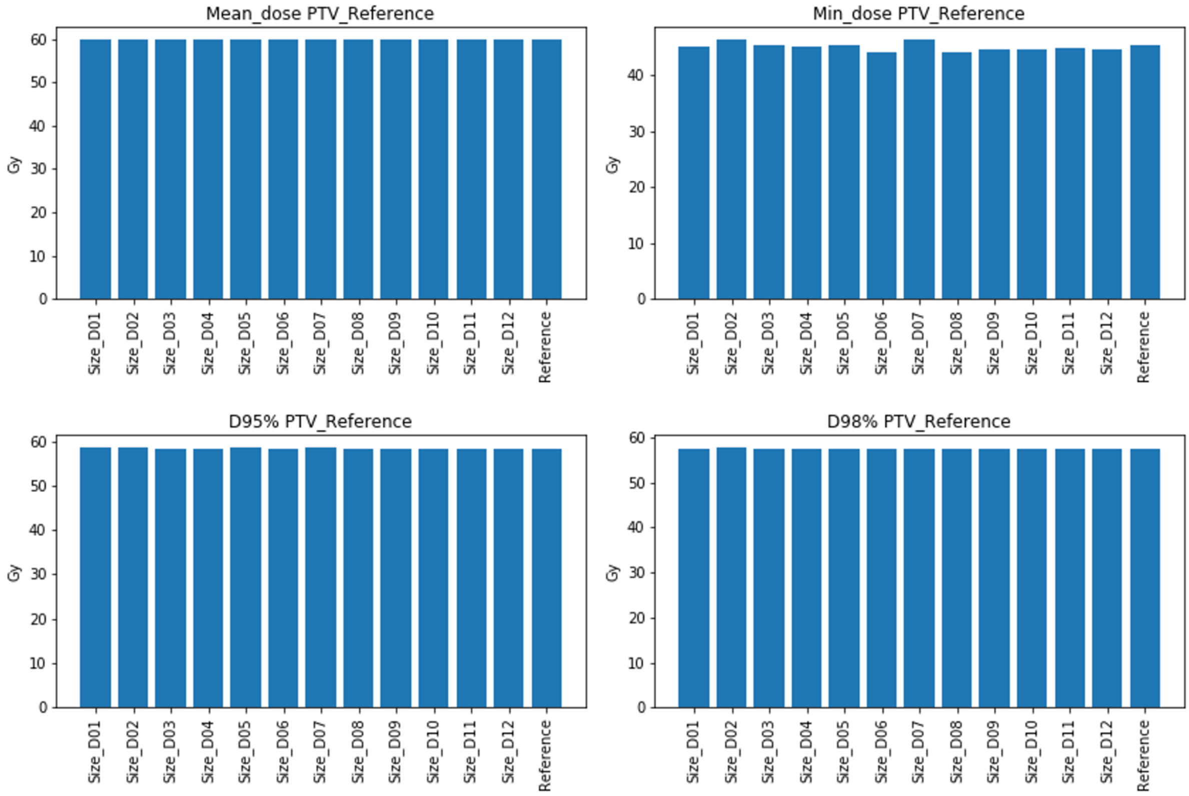

Figure 67: Bar plots of the mean dose, min dose and the 98% and 95% coverage of the PTV for the reference plan and the 12 plans including an outlier to the target at the specific sizes at location D as displayed in Figure 58.

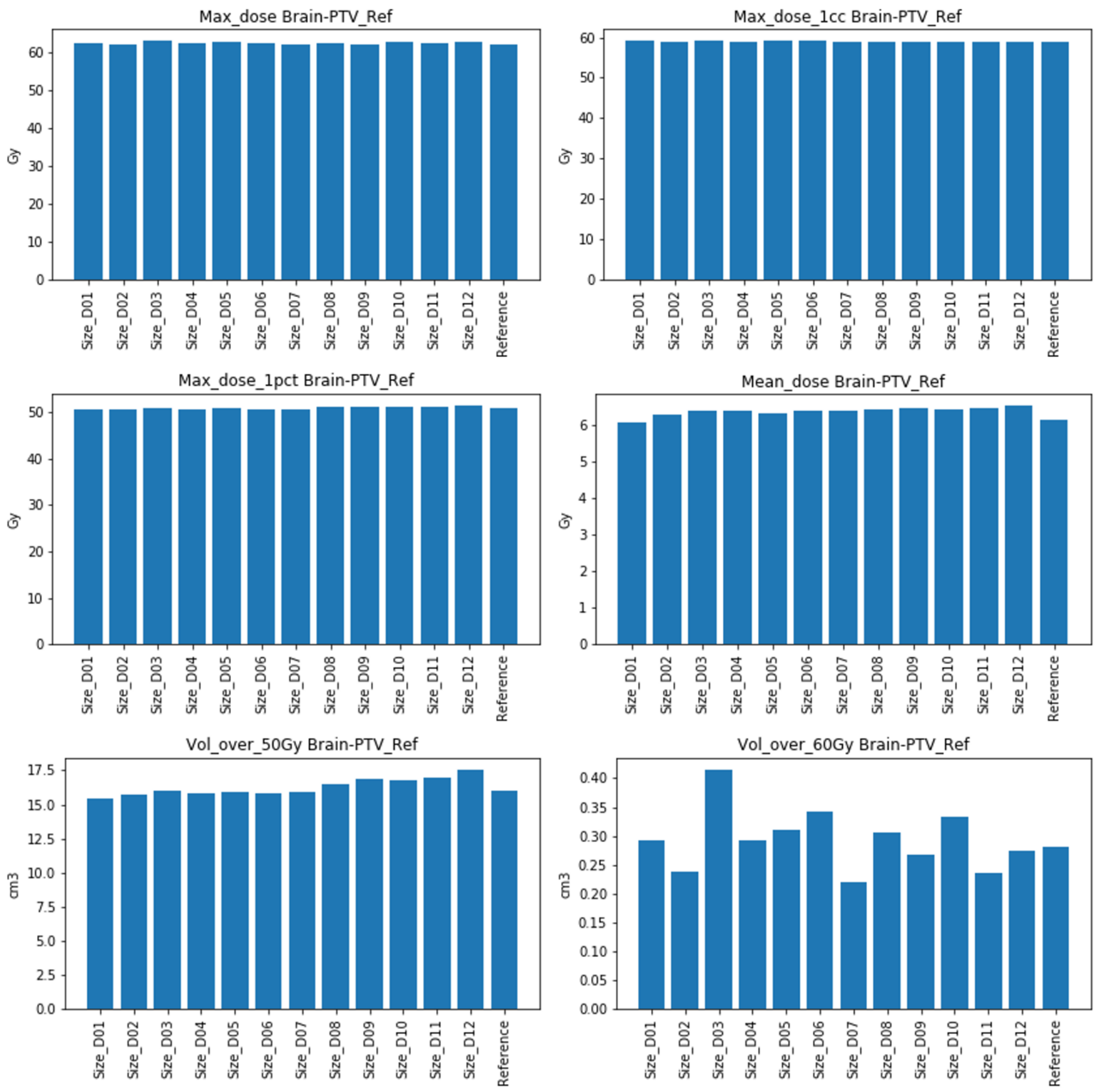

Figure 68: Bar plots of the max dose, mean dose, maximum dose to 1% of the volume, maximum dose to 1cc of the volume, the volume receiving over 50Gy and the volume receiving over 60 Gy to the healthy brain for the reference plan and the 12 plans including an outlier to the target at the specific sizes at location D as displayed in Figure 58.

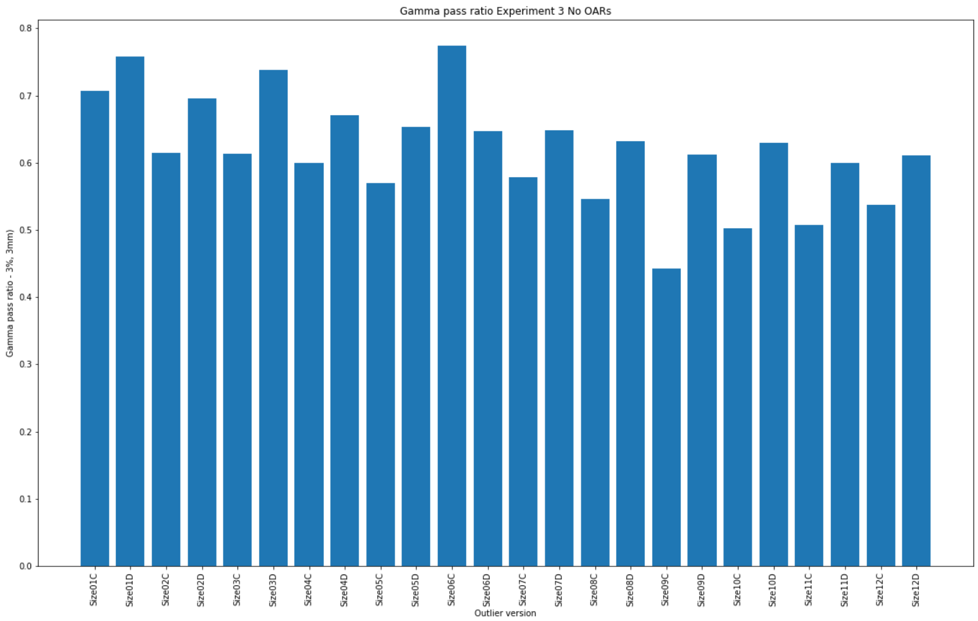

Figure 69: Bar plot of the Gamma pass ratio of each experimental plan containing an outlier at a specific size and location according to Figure 58. The plans did not involve OARs and the pass rate is determined with respect to the reference plan. The criteria for the gamma pass rate were set to 3% and 3 mm.

Experiment 3: Outlier size, OARs involved location C:

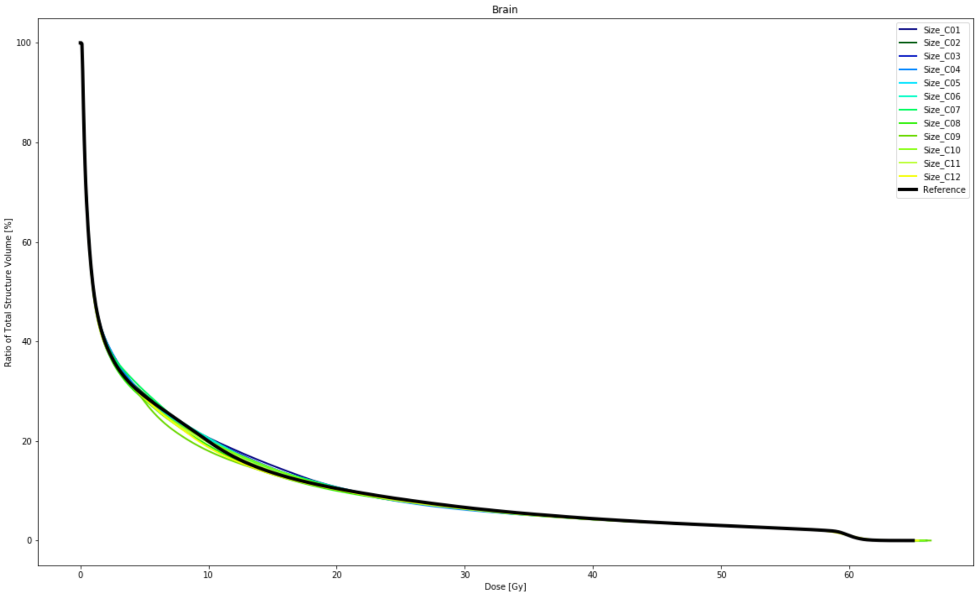

Figure 70: DVH curves of the brain of the reference plan and the 12 plans including an outlier to the target at the specific sizes at location C as displayed in Figure 58.

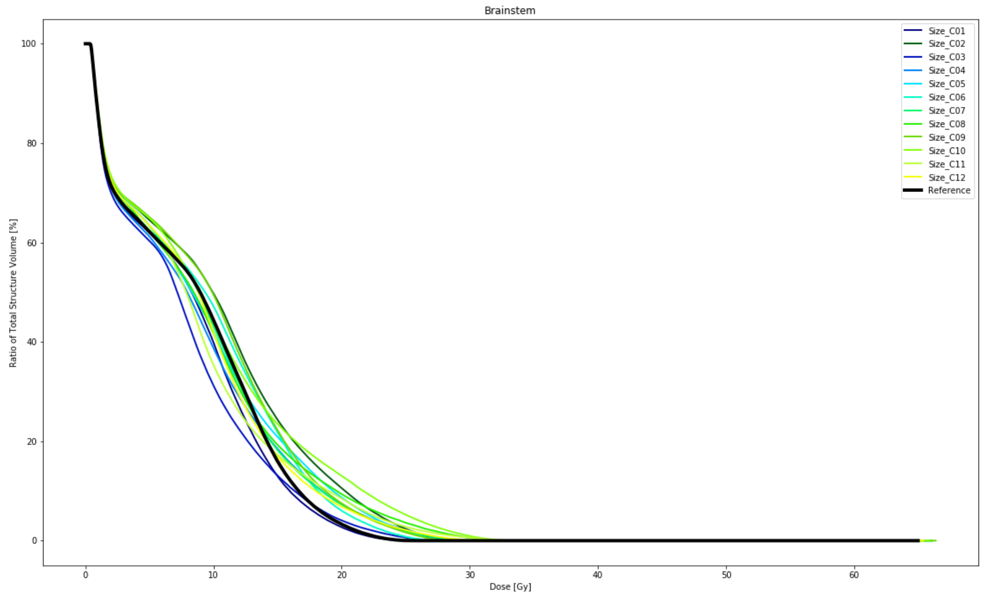

Figure 71: DVH curves of the brainstem of the reference plan and the 12 plans including an outlier to the target at the specific sizes at location C as displayed in Figure 58.

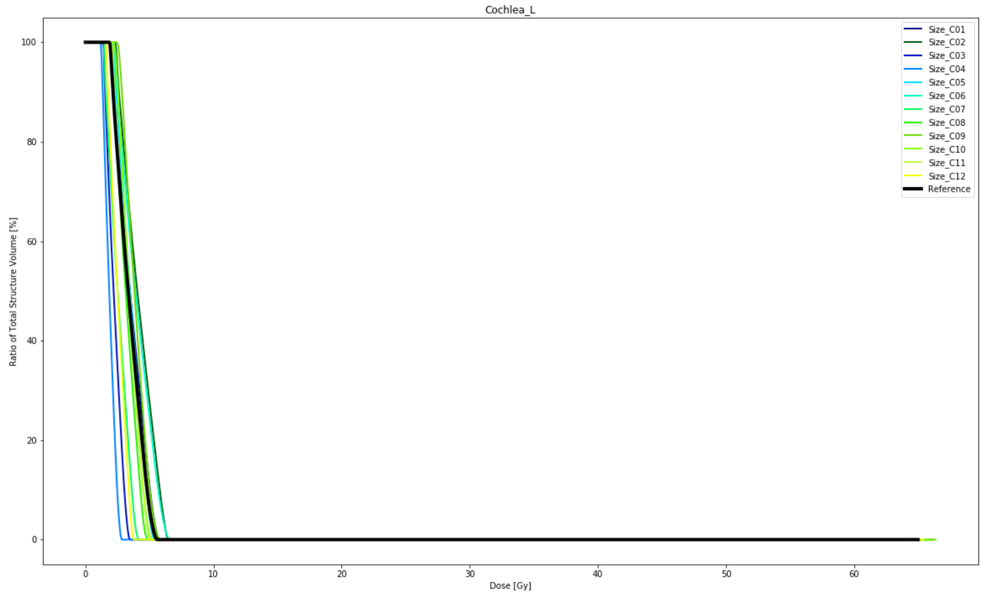

Figure 72: DVH curves of the left cochlea of the reference plan and the 12 plans including an outlier to the target at the specific sizes at location C as displayed in Figure 58.

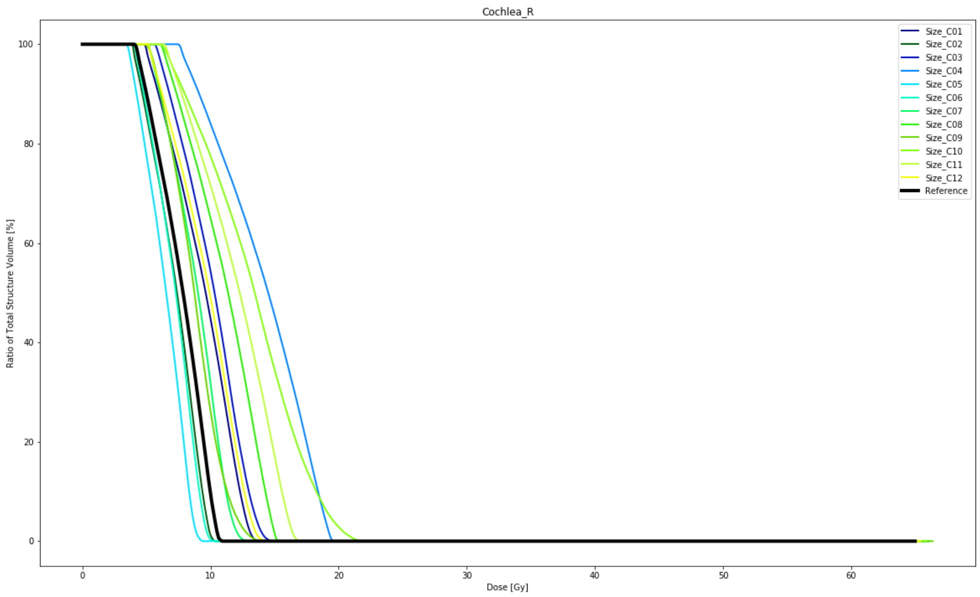

Figure 73: DVH curves of the right cochlea of the reference plan and the 12 plans including an outlier to the target at the specific sizes at location C as displayed in Figure 58.

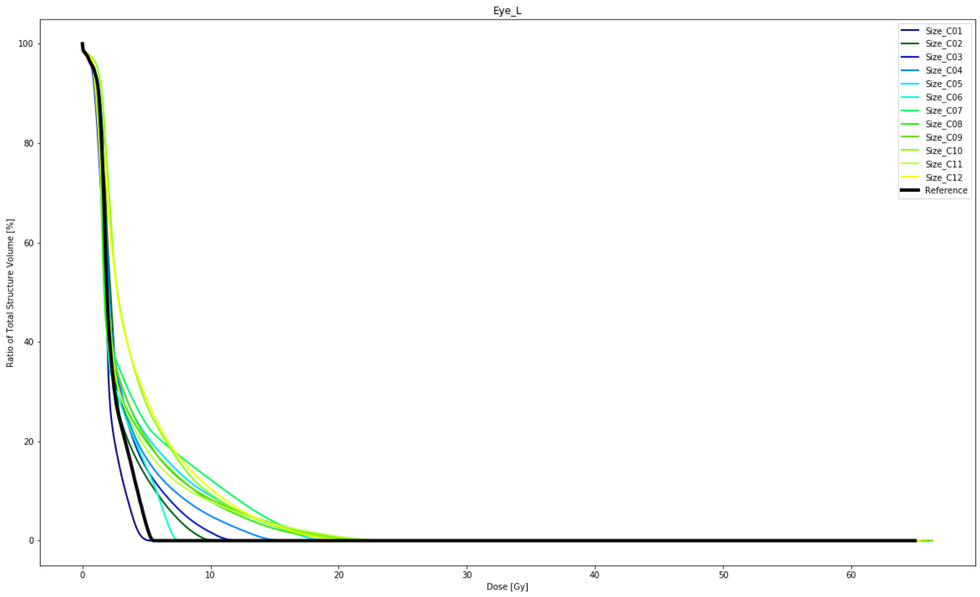

Figure 74: DVH curves of the left eye of the reference plan and the 12 plans including an outlier to the target at the specific sizes at location C as displayed in Figure 58.

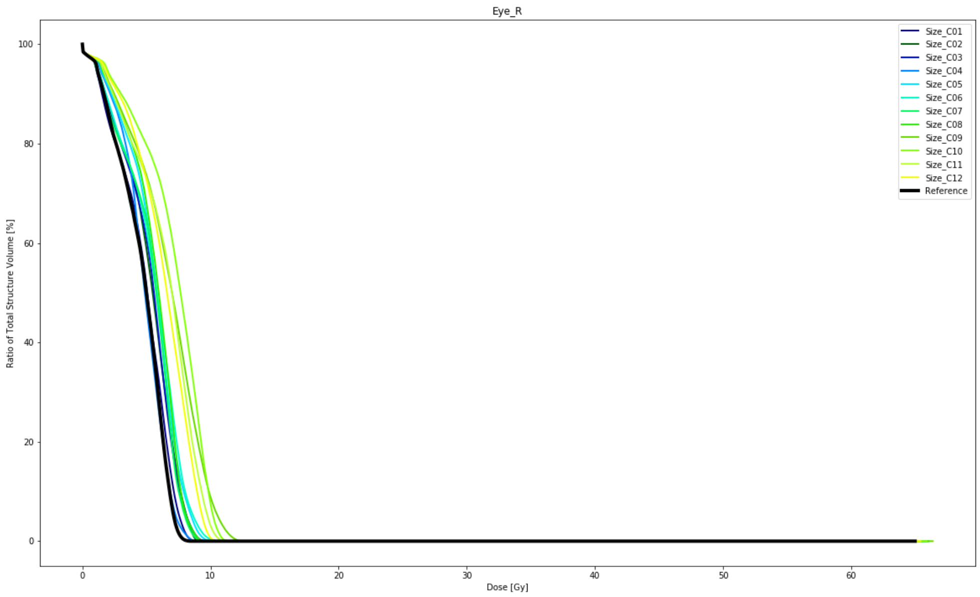

Figure 75: DVH curves of the right eye of the reference plan and the 12 plans including an outlier to the target at the specific sizes at location C as displayed in Figure 58.

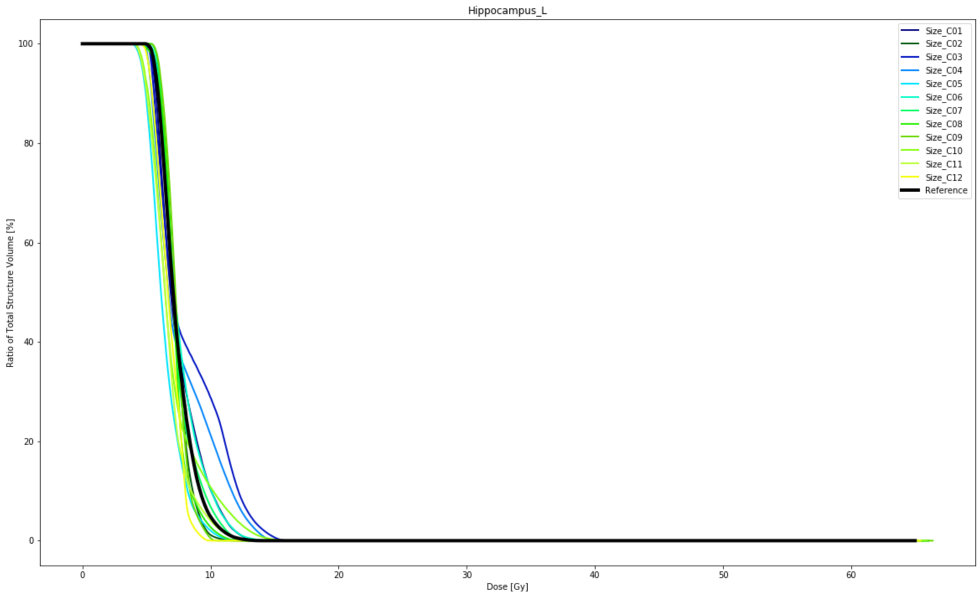

Figure 76: DVH curves of the left hippocampus of the reference plan and the 12 plans including an outlier to the target at the specific sizes at location C as displayed in Figure 58.

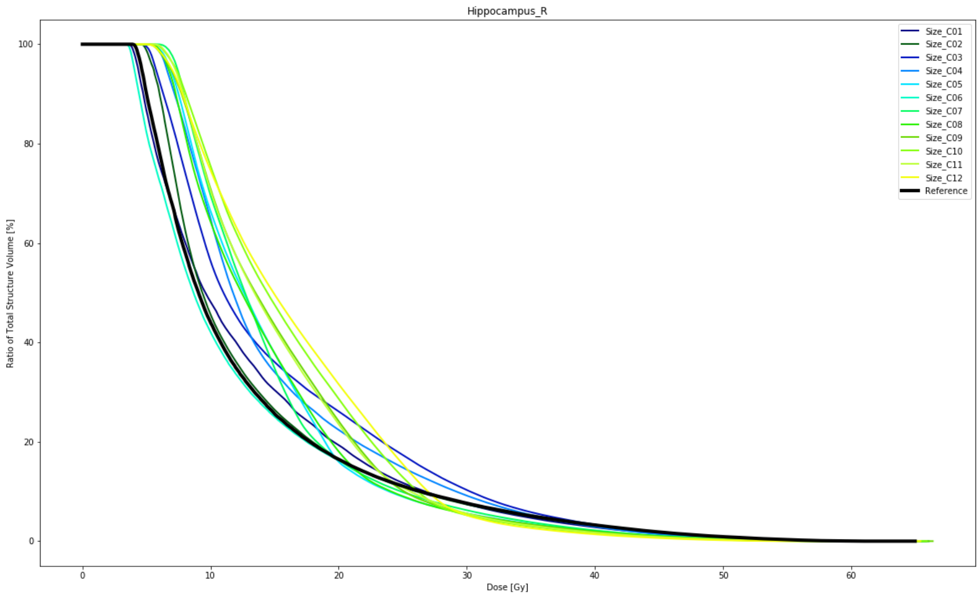

Figure 77: DVH curves of the right hippocampus of the reference plan and the 12 plans including an outlier to the target at the specific sizes at location C as displayed in Figure 58.

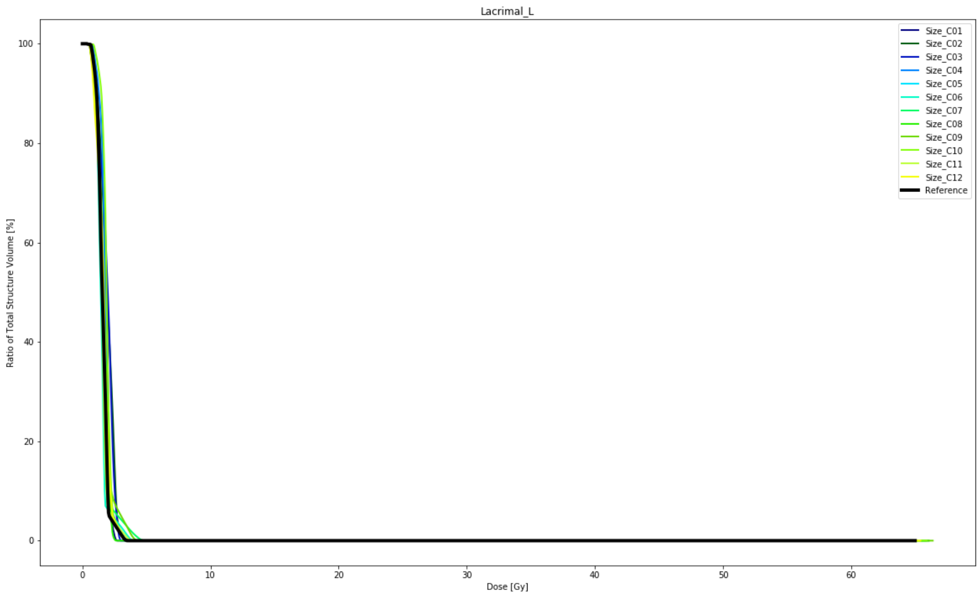

Figure 78: DVH curves of the left lacrimal gland of the reference plan and the 12 plans including an outlier to the target at the specific sizes at location C as displayed in Figure 58.

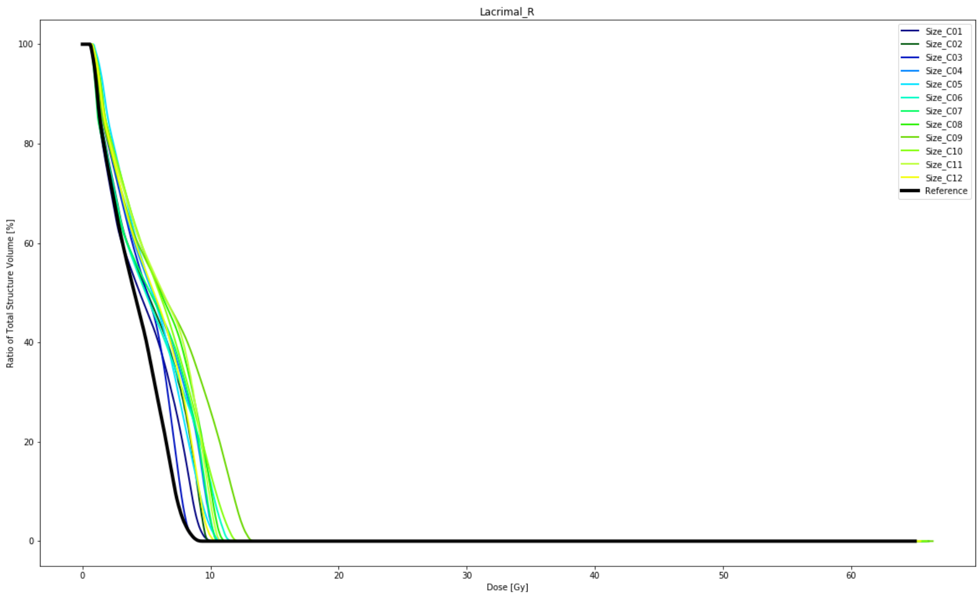

Figure 79: DVH curves of the right lacrimal gland of the reference plan and the 12 plans including an outlier to the target at the specific sizes at location C as displayed in Figure 58.

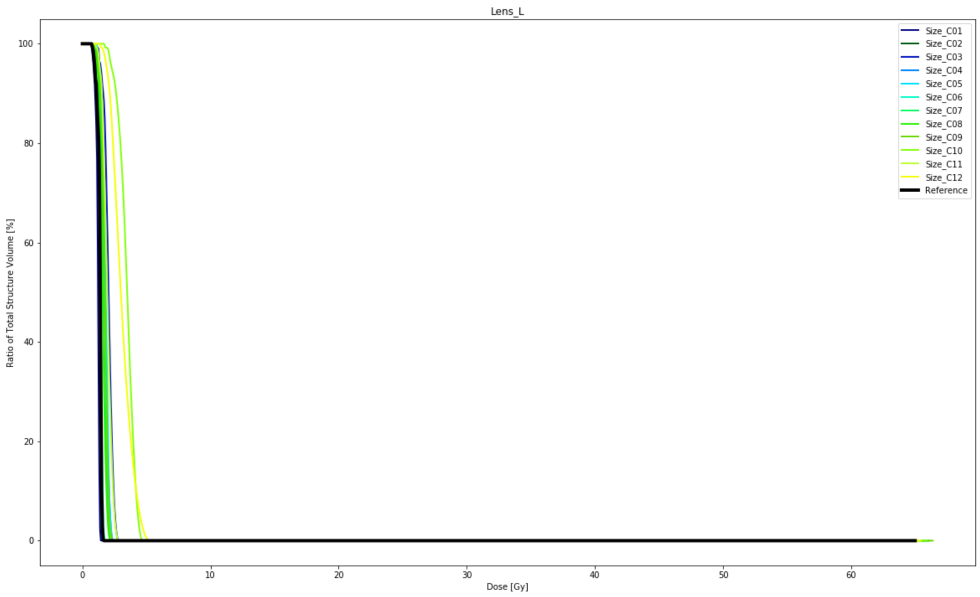

Figure 80: DVH curves of the left lens of the reference plan and the 12 plans including an outlier to the target at the specific sizes at location C as displayed in Figure 58.

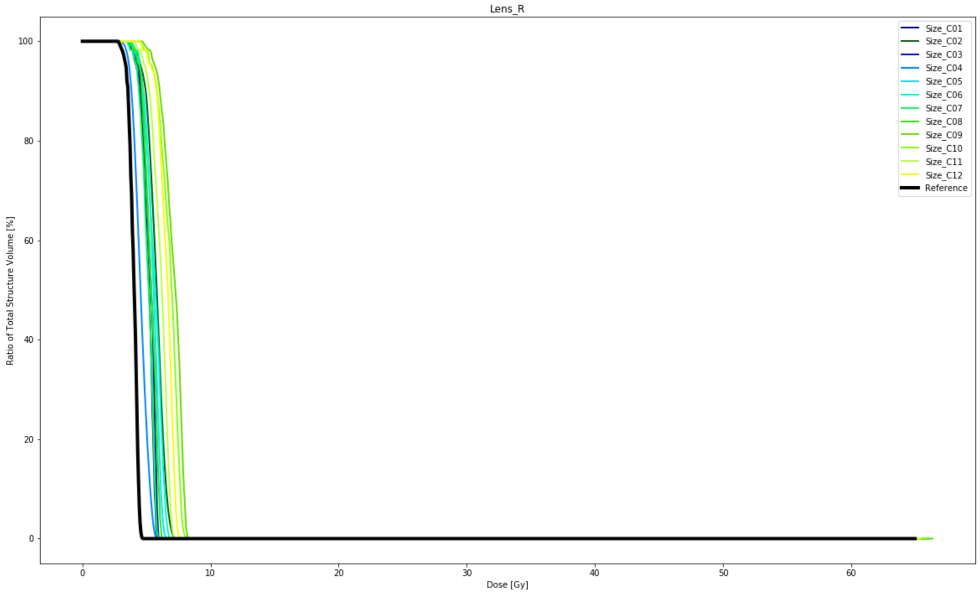

Figure 81: DVH curves of the right lens of the reference plan and the 12 plans including an outlier to the target at the specific sizes at location C as displayed in Figure 58.

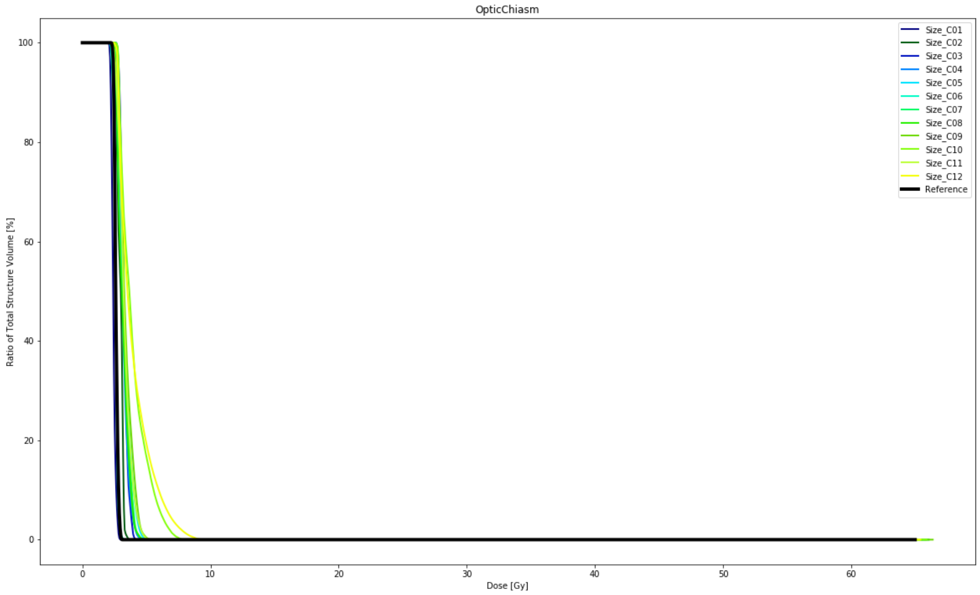

Figure 82: DVH curves of the optic chiasm of the reference plan and the 12 plans including an outlier to the target at the specific sizes at location C as displayed in Figure 58.

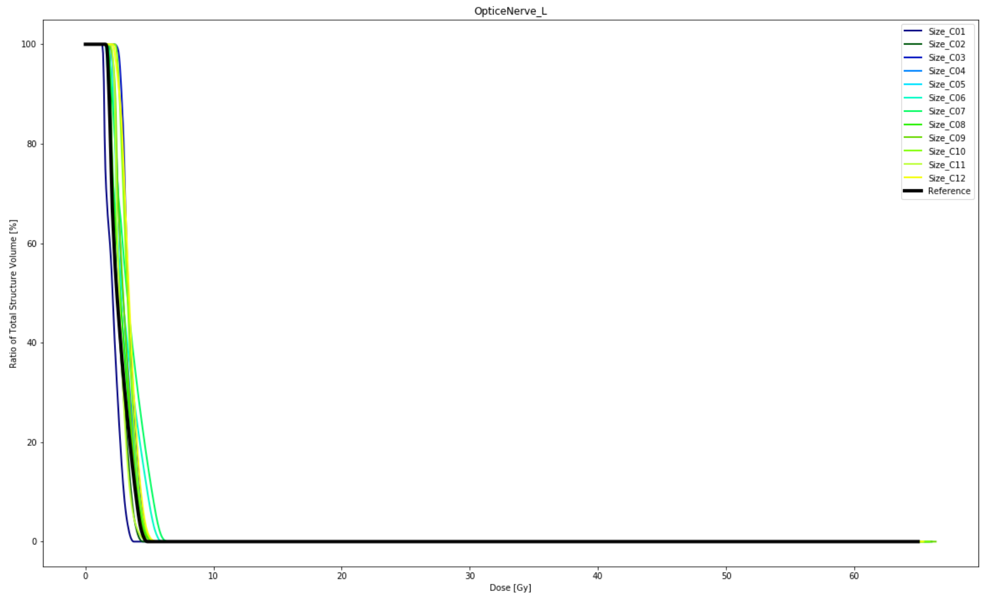

Figure 83: DVH curves of the left optic nerve of the reference plan and the 12 plans including an outlier to the target at the specific sizes at location C as displayed in Figure 58.

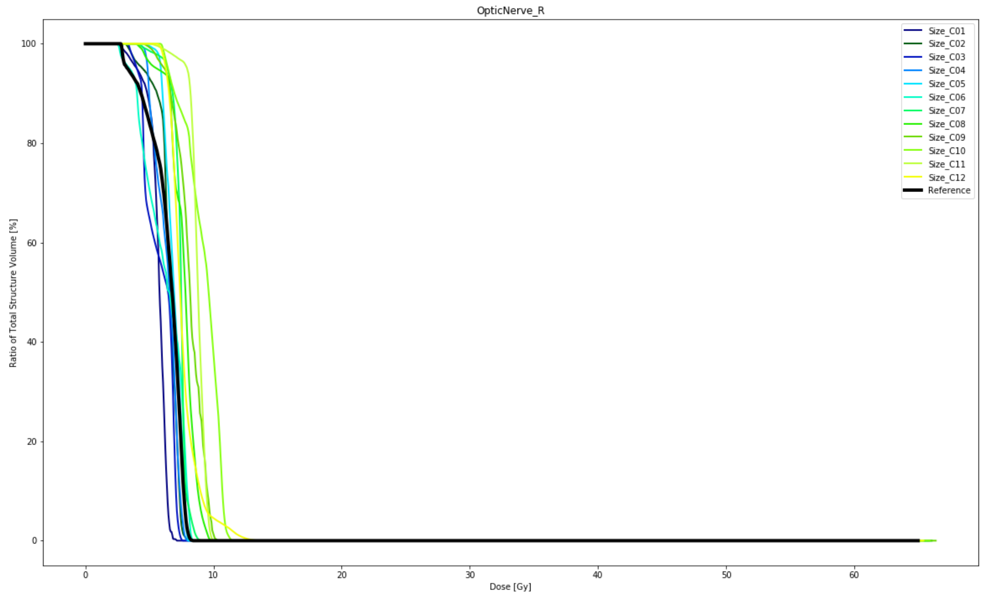

Figure 84: DVH curves of the right optic nerve of the reference plan and the 12 plans including an outlier to the target at the specific sizes at location C as displayed in Figure 58.

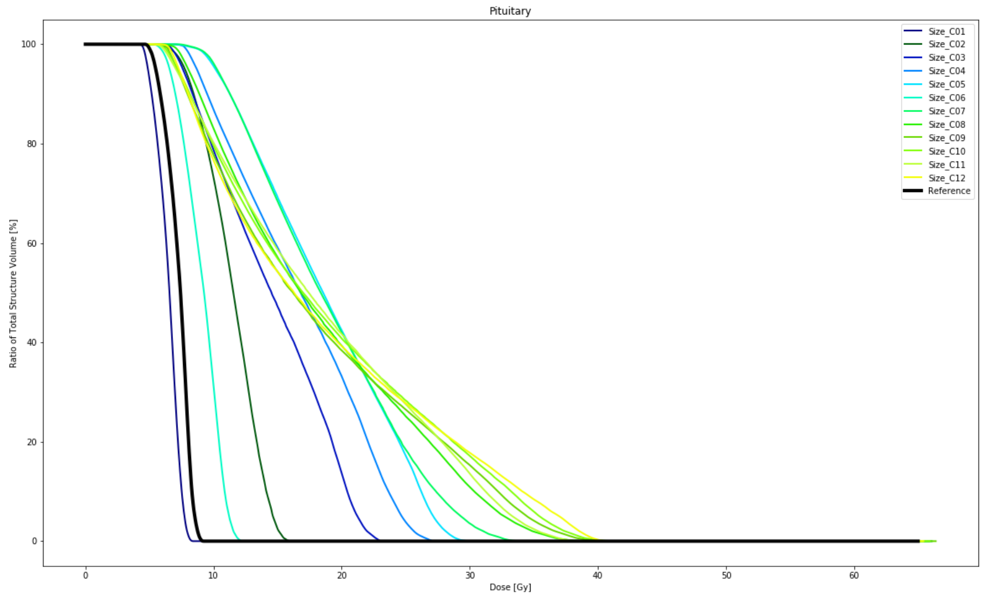

Figure 85: DVH curves of the pituitary gland of the reference plan and the 12 plans including an outlier to the target at the specific sizes at location C as displayed in Figure 58.

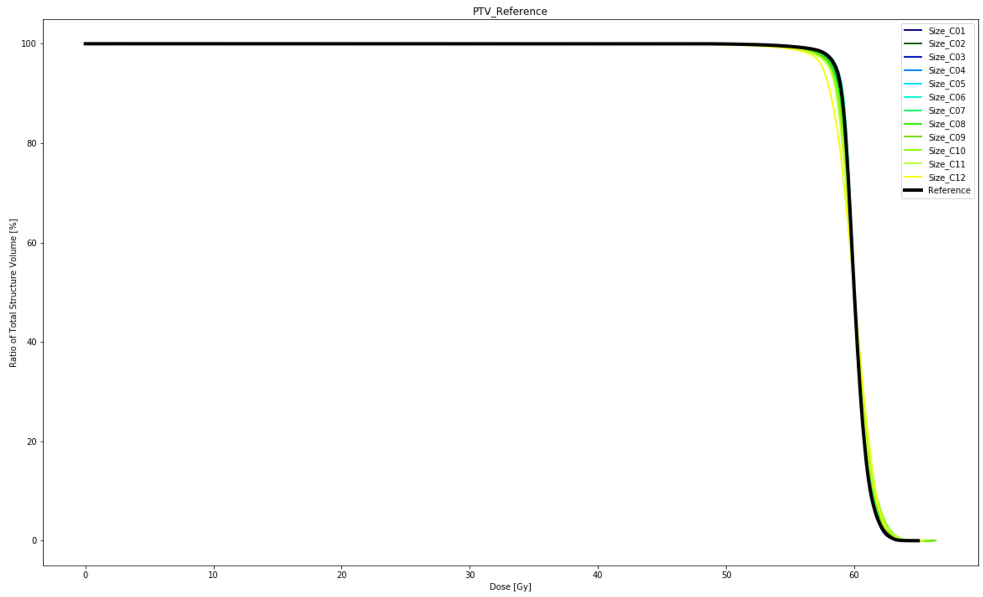

Figure 86: DVH curves of the PTV of the reference plan and the 12 plans including an outlier to the target at the specific sizes at location C as displayed in Figure 58.

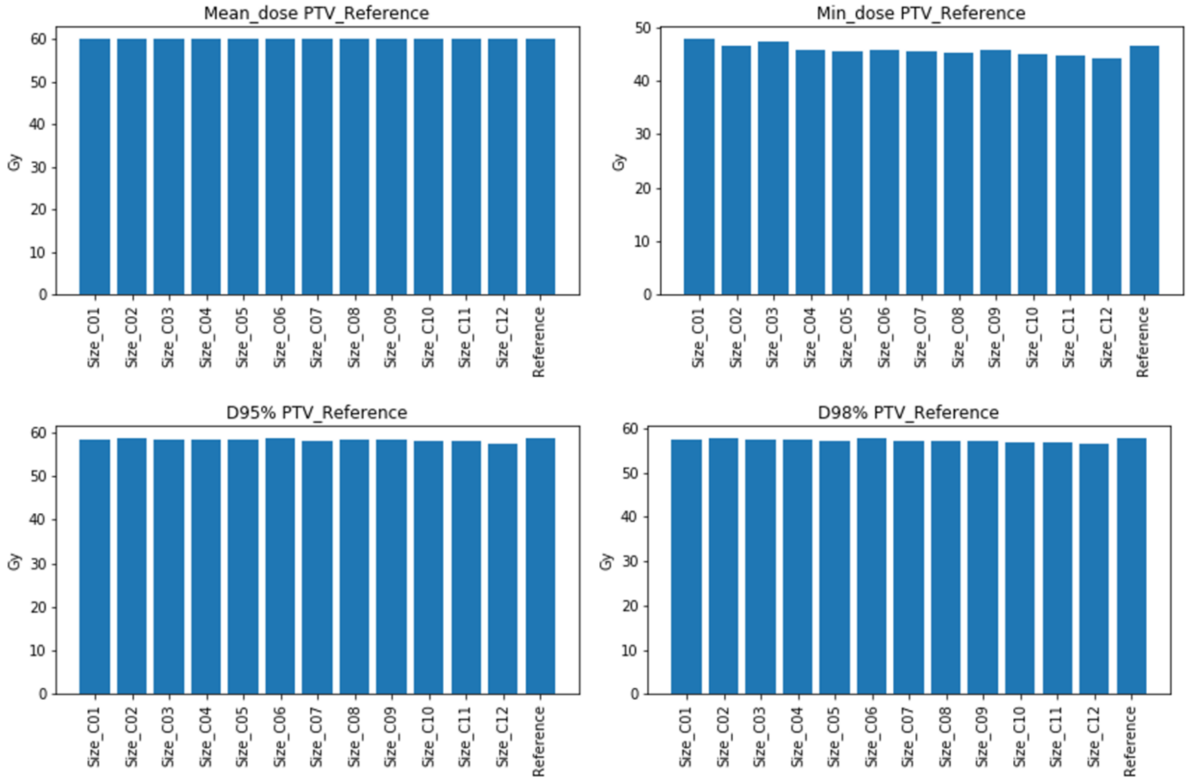

Figure 87: Bar plots of the mean dose, min dose and the 98% and 95% coverage of the PTV for the reference plan and 12 plans including an outlier to the target at the specific sizes at location C as displayed in Figure 58.

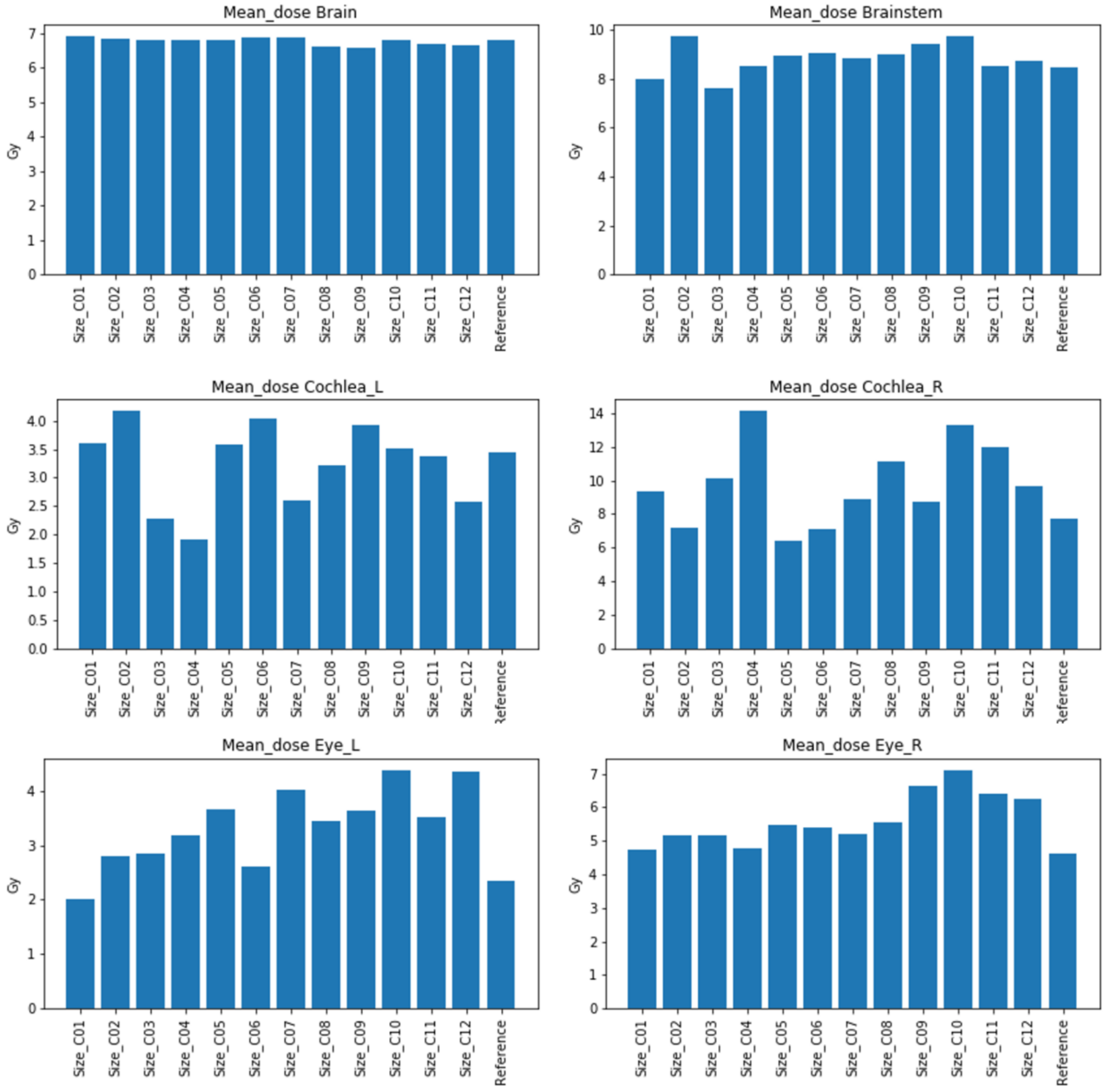

Figure 88: Bar plots of the mean dose of the brain, brainstem, cochlea and eyes, for the reference plan and the 12 plans including an outlier to the target at the specific sizes at location C as displayed in Figure 58.

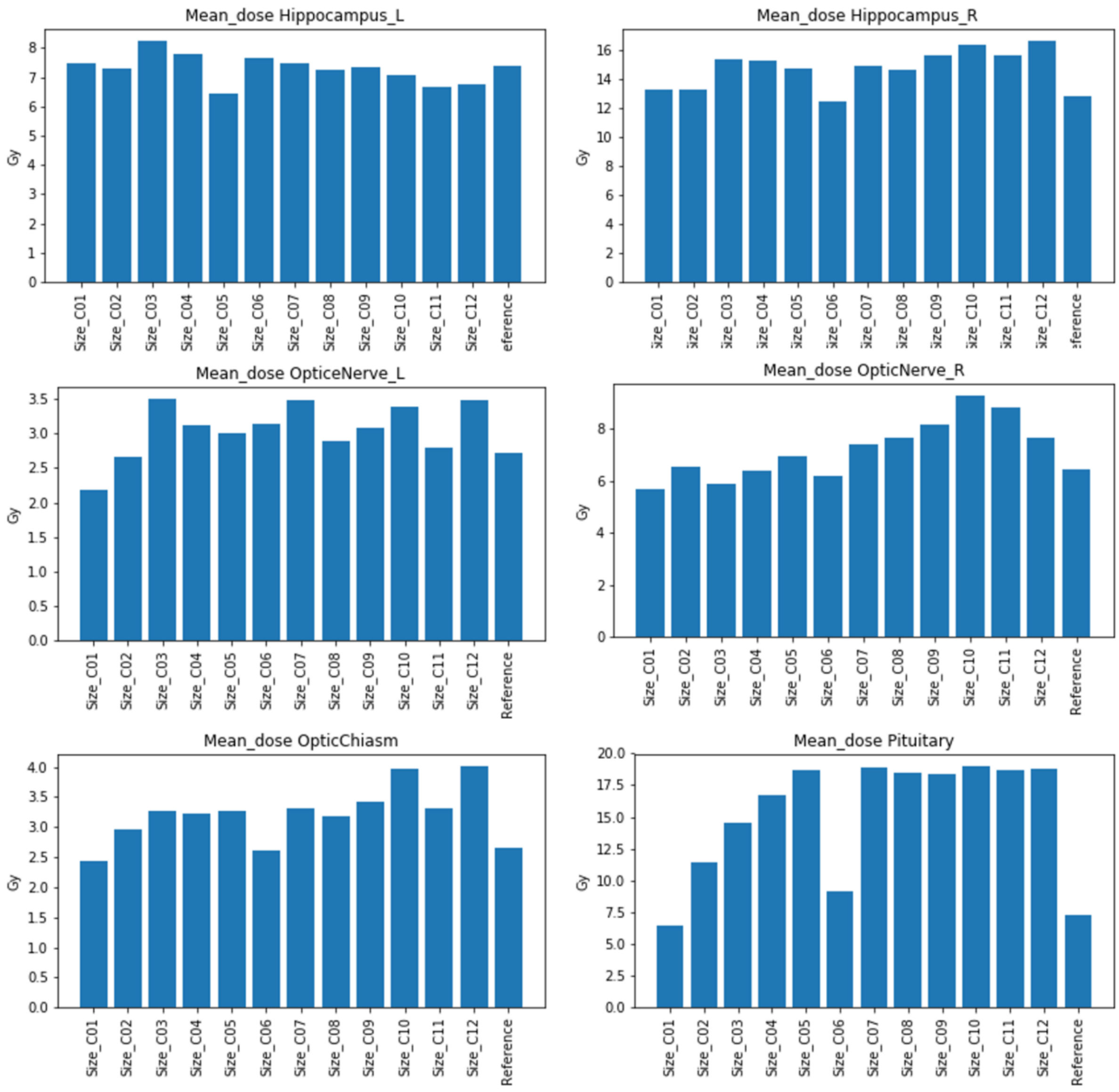

Figure 89: Bar plots of the mean dose of the hippocampi, optic nerves, optic chiasm and pituitary, for the reference plan and the 12 plans including an outlier to the target at the specific sizes at location C as displayed in Figure 58

Experiment 3: Outlier size, no OARs involved at location D:

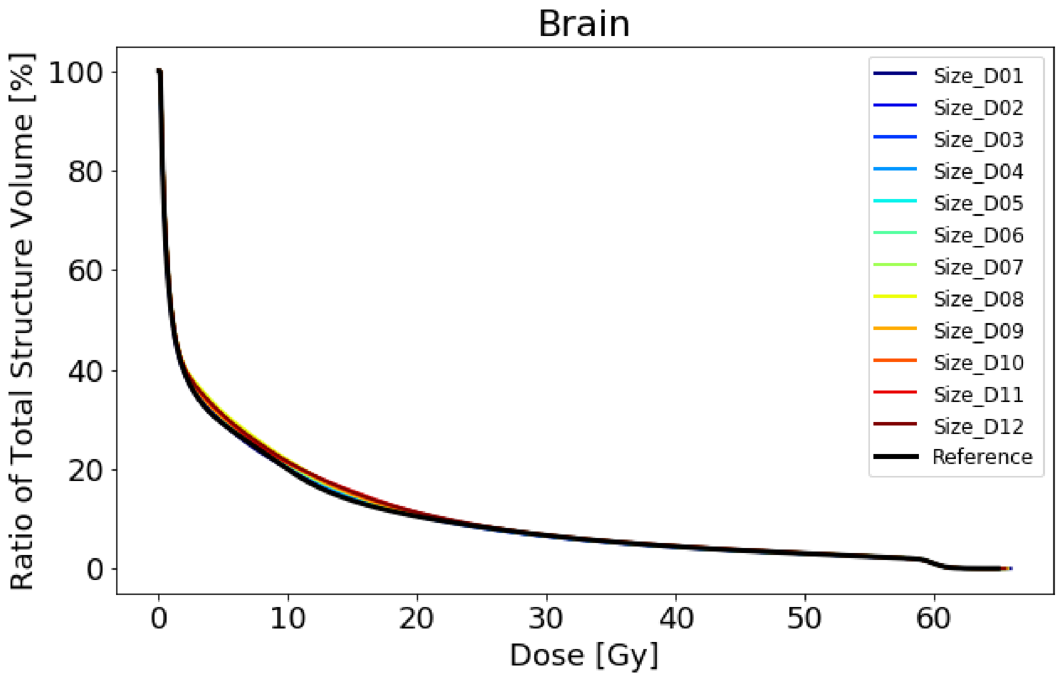

Figure 90: DVH curves of the brain of the reference plan and the 12 plans including an outlier to the target at the specific sizes at location D as displayed in Figure 58.

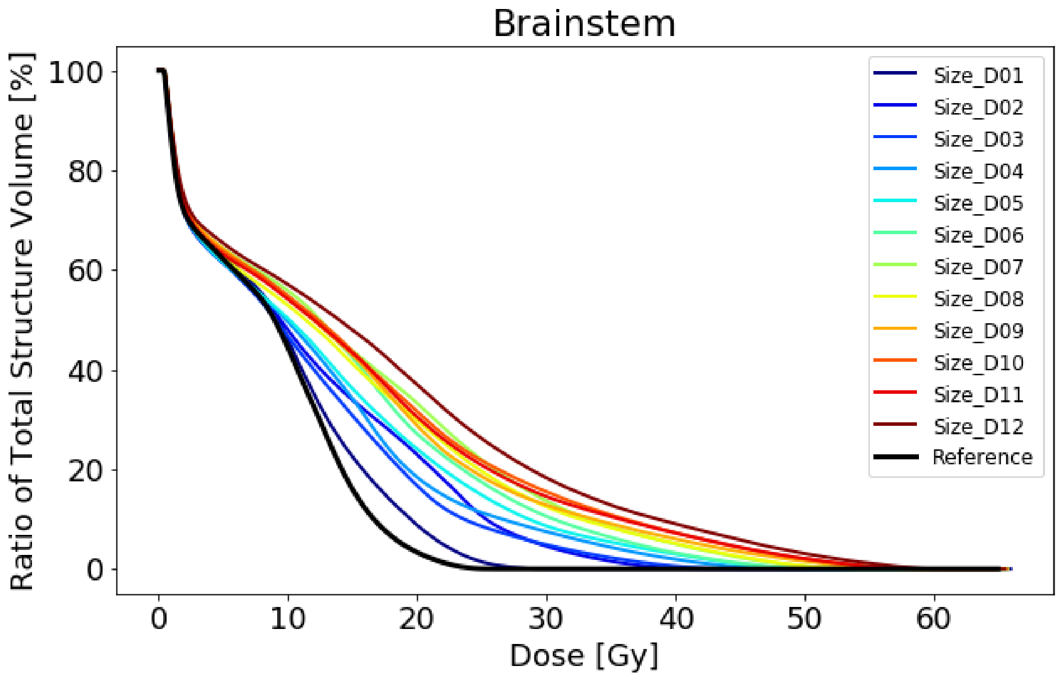

Figure 91: DVH curves of the brainstem of the reference plan and the 12 plans including an outlier to the target at the specific sizes at location D as displayed in Figure 58.

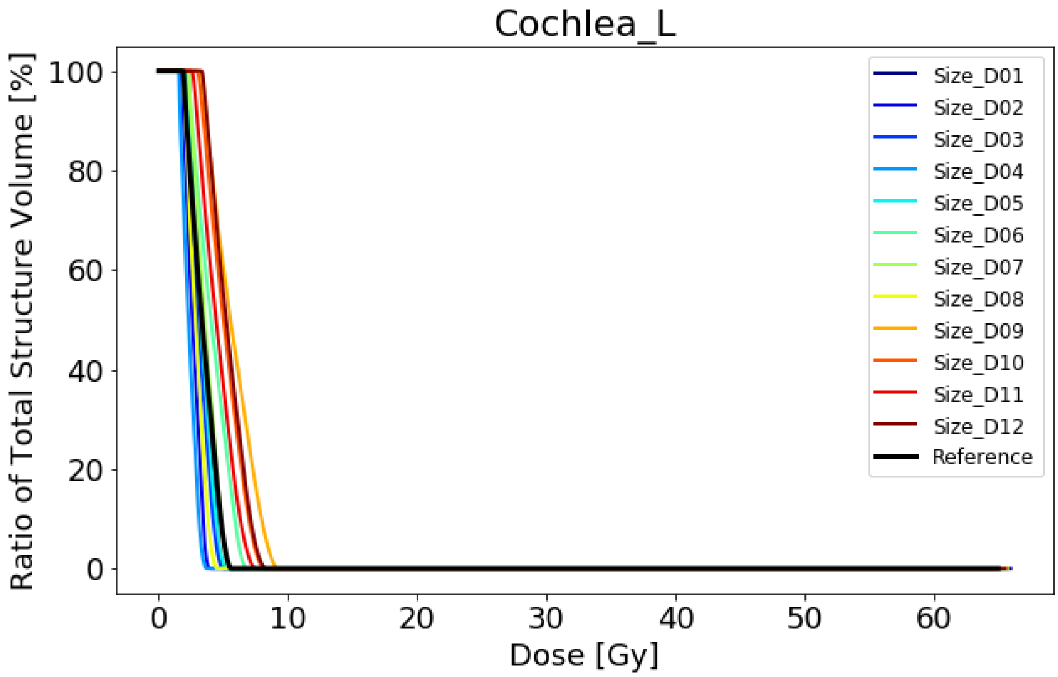

Figure 92: DVH curves of the left cochlea of the reference plan and the 12 plans including an outlier to the target at the specific sizes at location D as displayed in Figure 58.

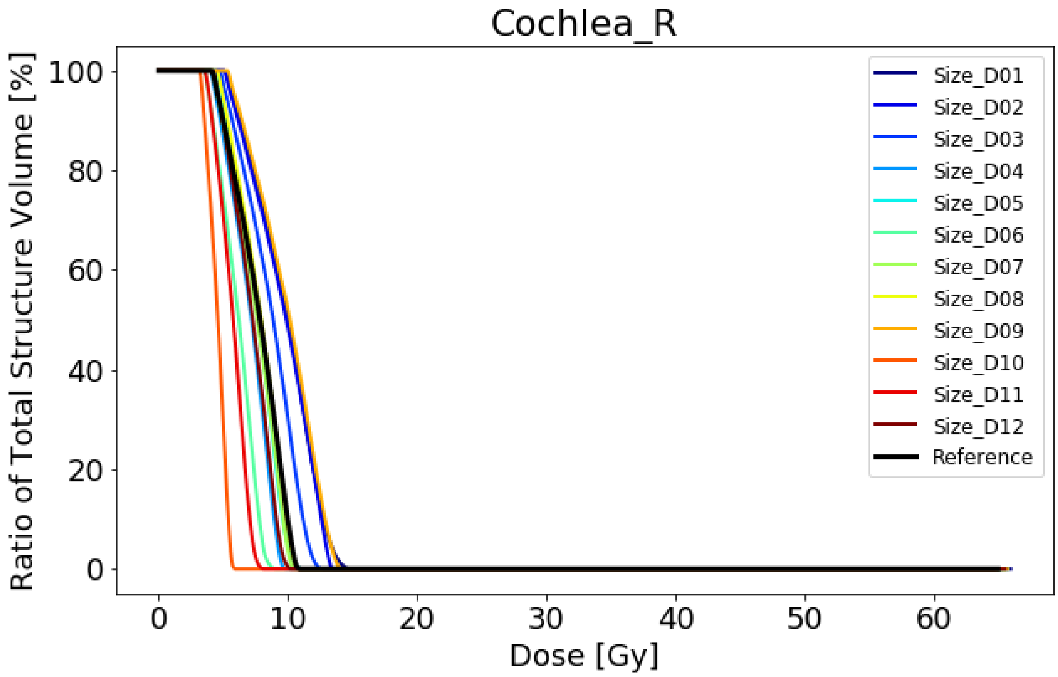

Figure 93: DVH curves of the right cochlea of the reference plan and the 12 plans including an outlier to the target at the specific sizes at location D as displayed in Figure 58.

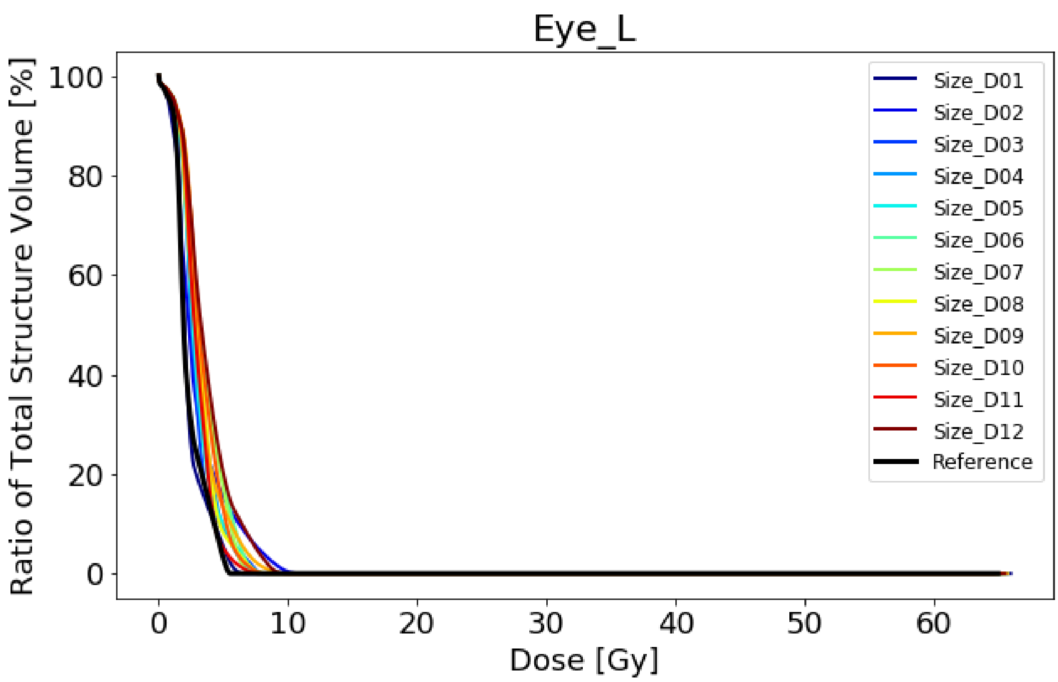

Figure 94: DVH curves of the left eye of the reference plan and the 12 plans including an outlier to the target at the specific sizes at location D as displayed in Figure 58.

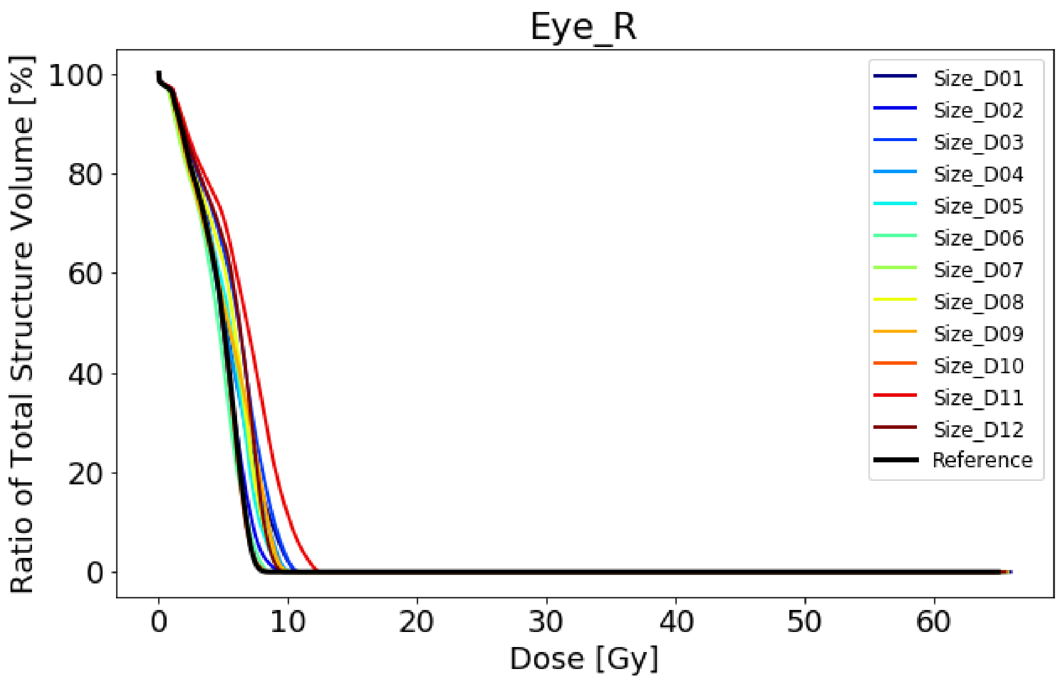

Figure 95: DVH curves of the right eye of the reference plan and the 12 plans including an outlier to the target at the specific sizes at location D as displayed in Figure 58.

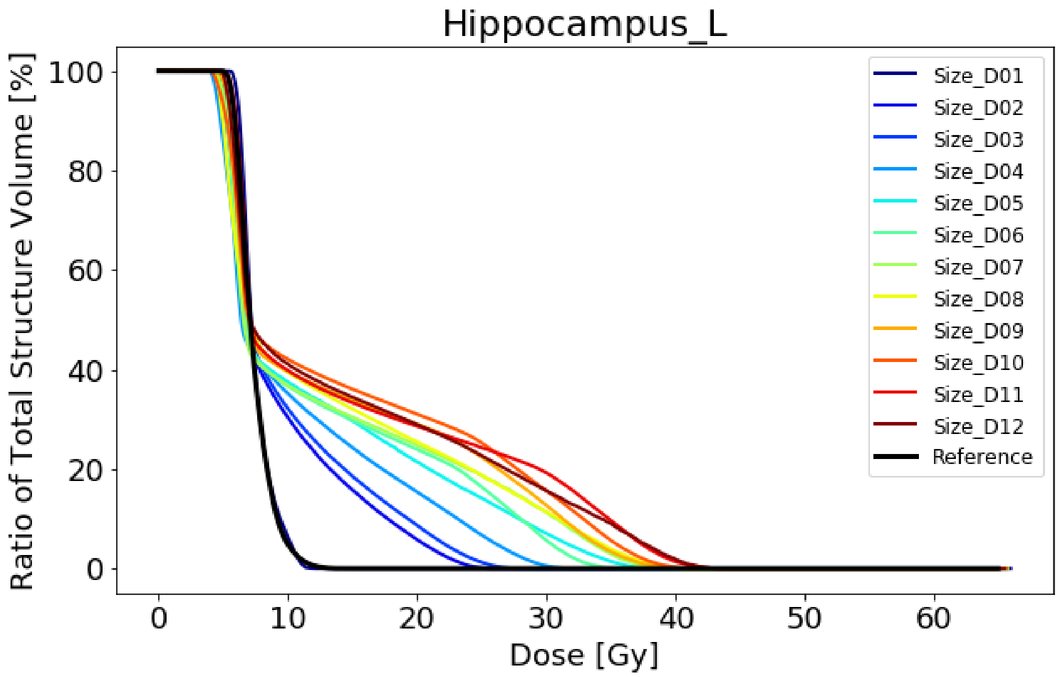

Figure 96: DVH curves of the left hippocampus of the reference plan and the 12 plans including an outlier to the target at the specific sizes at location D as displayed in Figure 58.

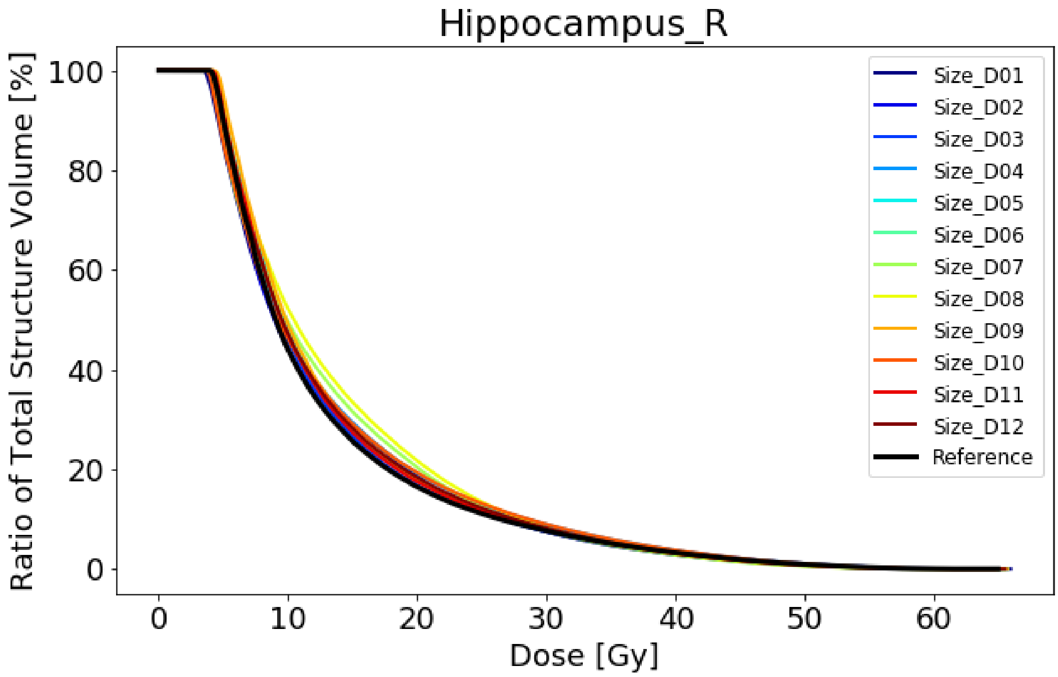

Figure 97: DVH curves of the right hippocampus of the reference plan and the 12 plans including an outlier to the target at the specific sizes at location D as displayed in Figure 58.

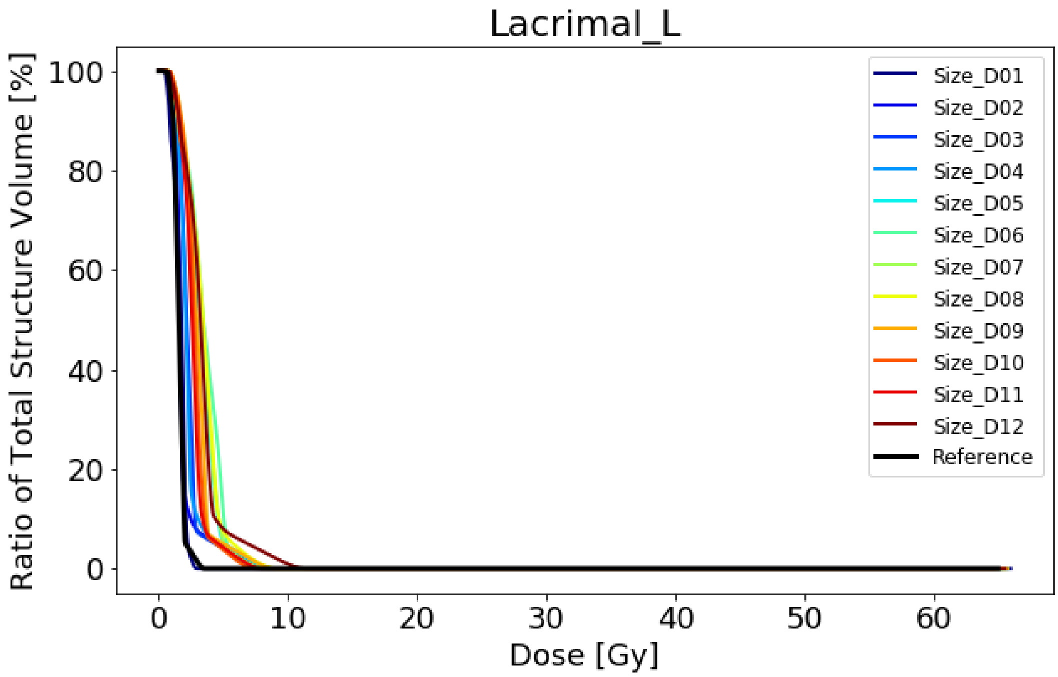

Figure 98: DVH curves of the left lacrimal gland of the reference plan and the 12 plans including an outlier to the target at the specific sizes at location D as displayed in Figure 58.

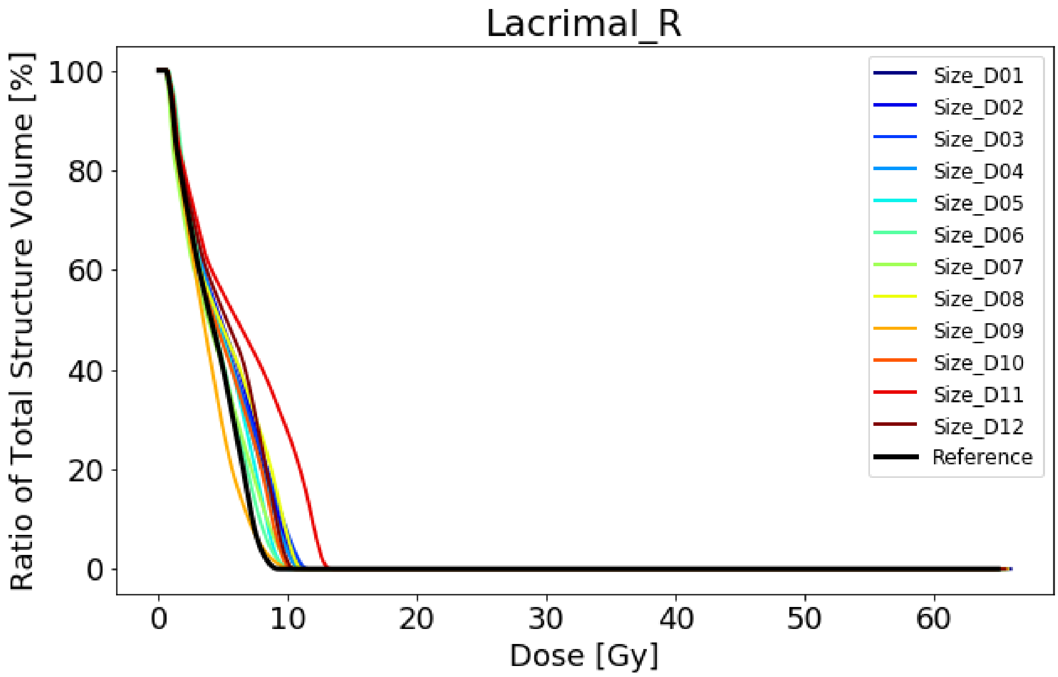

Figure 99: DVH curves of the right lacrimal gland of the reference plan and the 12 plans including an outlier to the target at the specific sizes at location D as displayed in Figure 58.

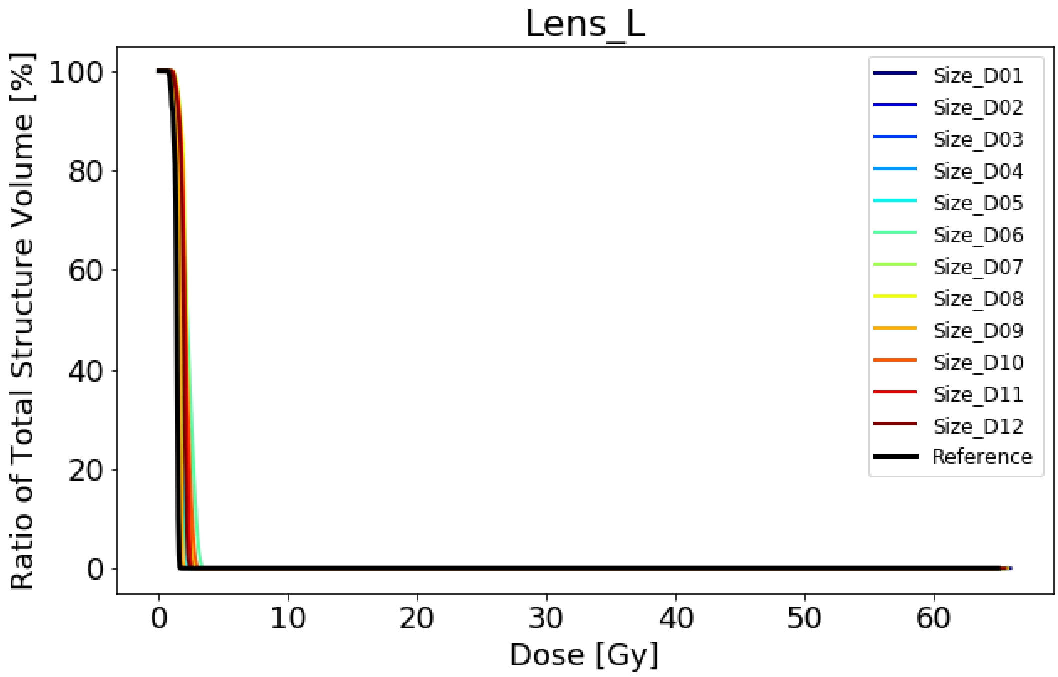

Figure 100: DVH curves of the left lens of the reference plan and the 12 plans including an outlier to the target at the specific sizes at location D as displayed in Figure 58.

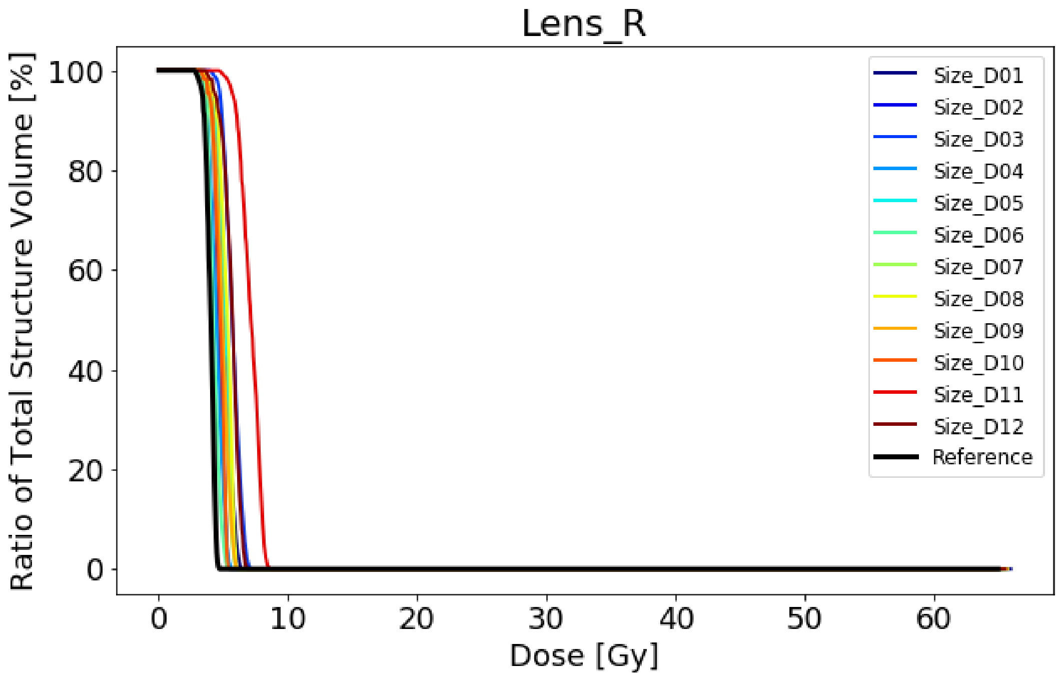

Figure 101: DVH curves of the right lens of the reference plan and the 12 plans including an outlier to the target at the specific sizes at location D as displayed in Figure 58.

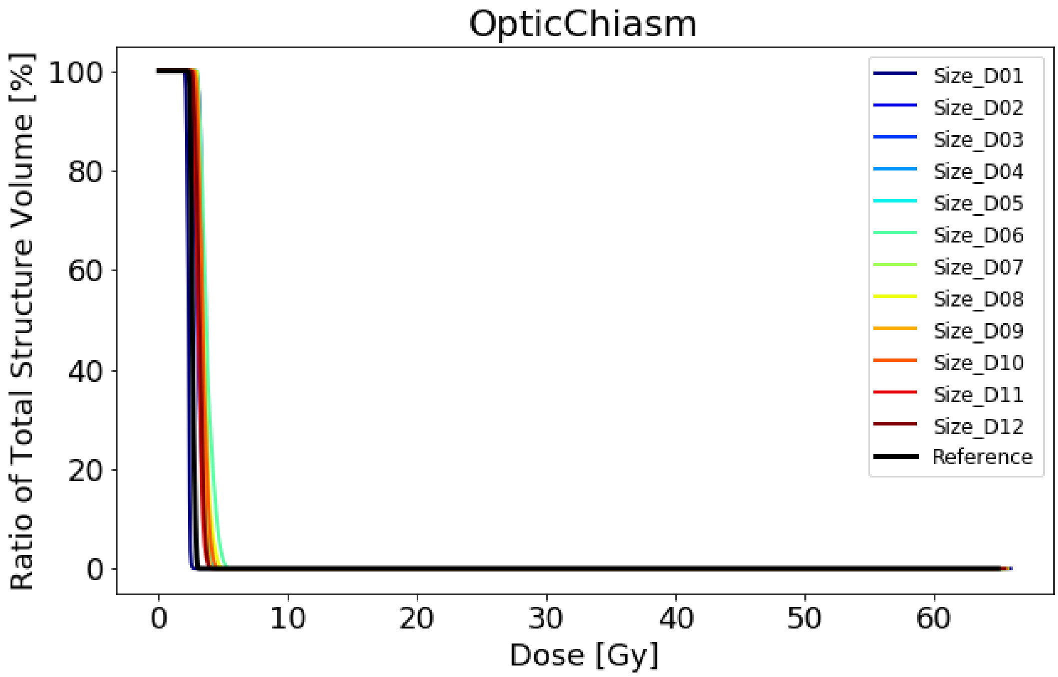

Figure 102. DVH curves of the optic chiasm of the reference plan and the 12 plans including an outlier to the target at the specific sizes at location D as displayed in Figure 58.

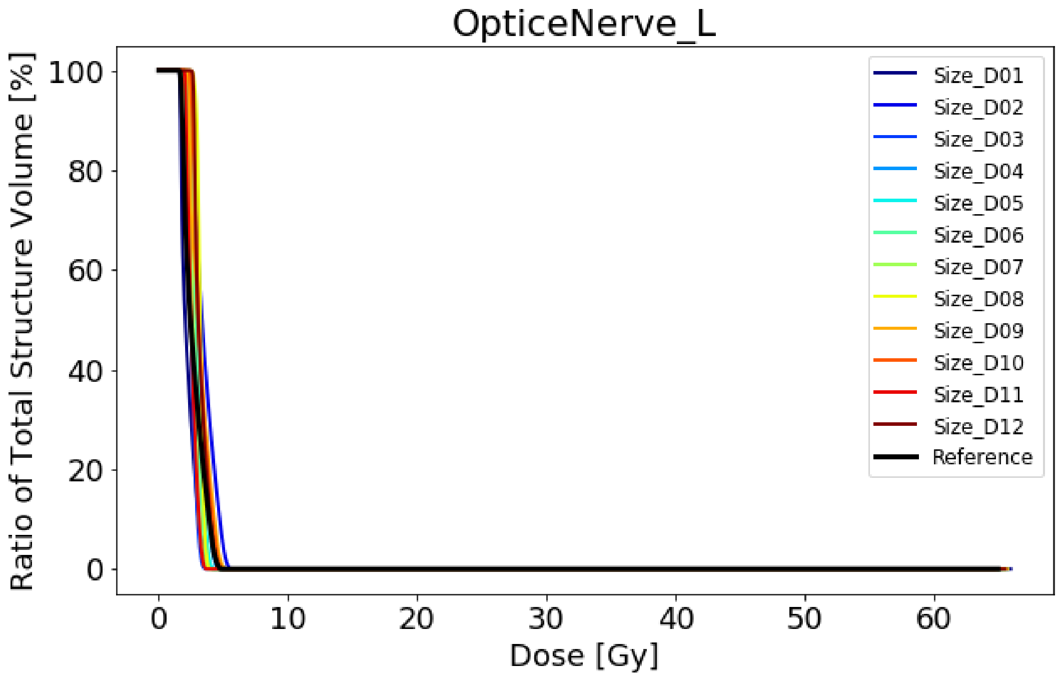

Figure 103: DVH curves of the left optic nerve of the reference plan and the 12 plans including an outlier to the target at the specific sizes at location D as displayed in Figure 58.

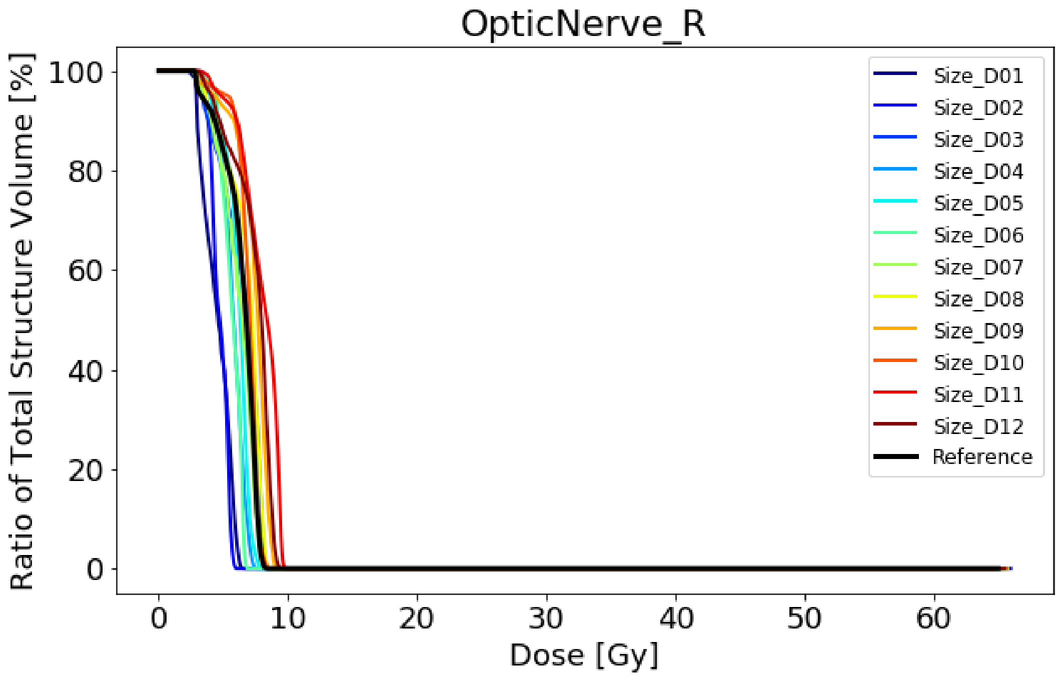

Figure 104: DVH curves of the right optic nerve of the reference plan and the 12 plans including an outlier to the target at the specific sizes at location D as displayed in Figure 58.

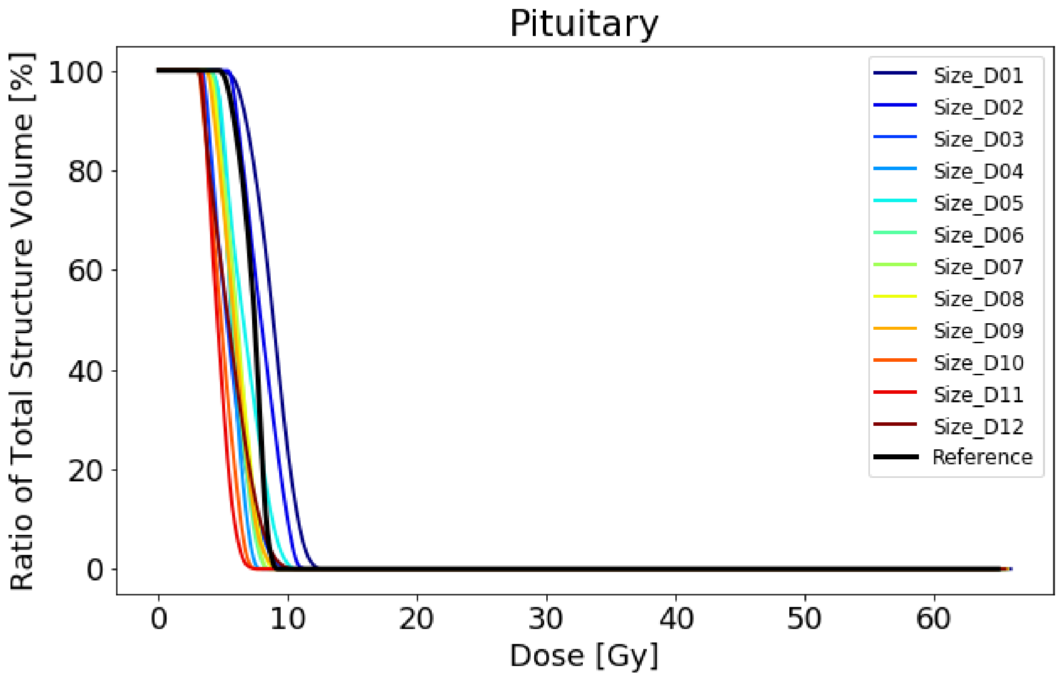

Figure 105: DVH curves of the pituitary gland of the reference plan and the 12 plans including an outlier to the target at the specific sizes at location D as displayed in Figure 58.

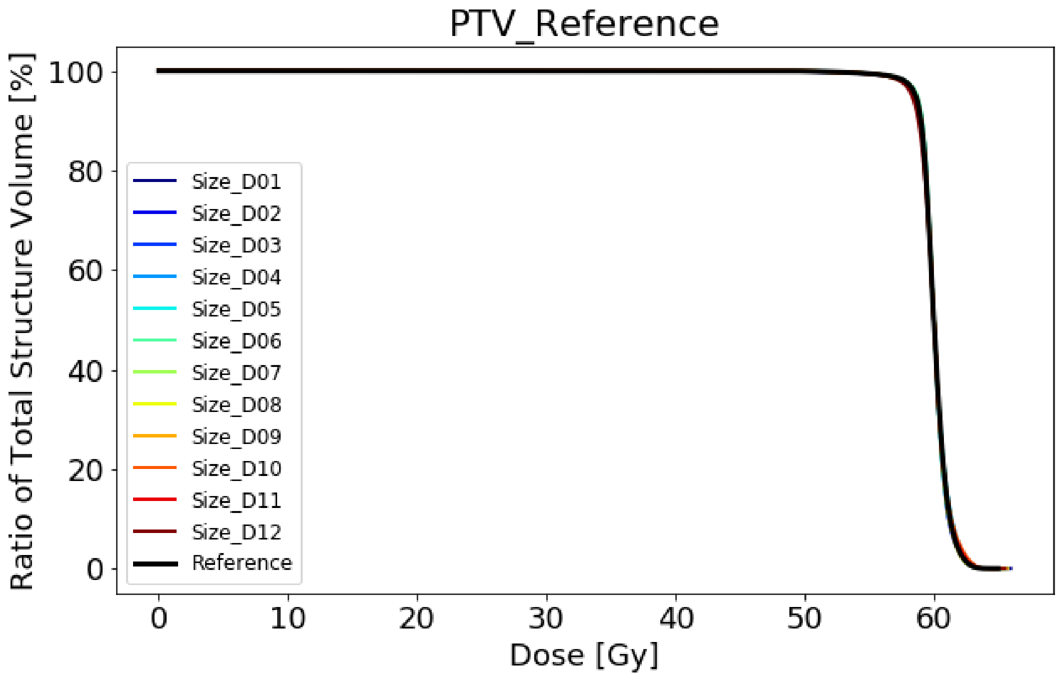

Figure 106: DVH curves of the PTV of the reference plan and the 12 plans including an outlier to the target at the specific sizes at location D as displayed in Figure 58.

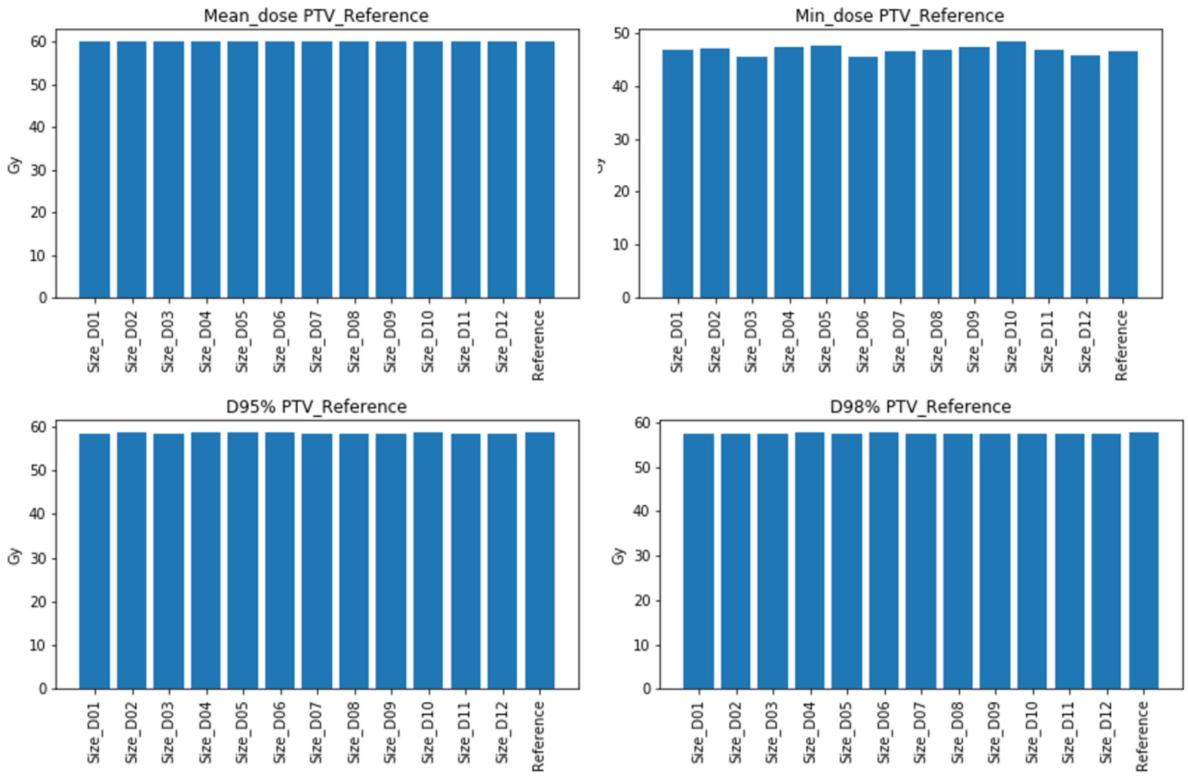

Figure 107: Bar plots of the mean dose, min dose and the 95% and 98% coverage of the PTV for the reference plan and 12 plans including an outlier to the target at the specific sizes at location D as displayed in Figure 58.

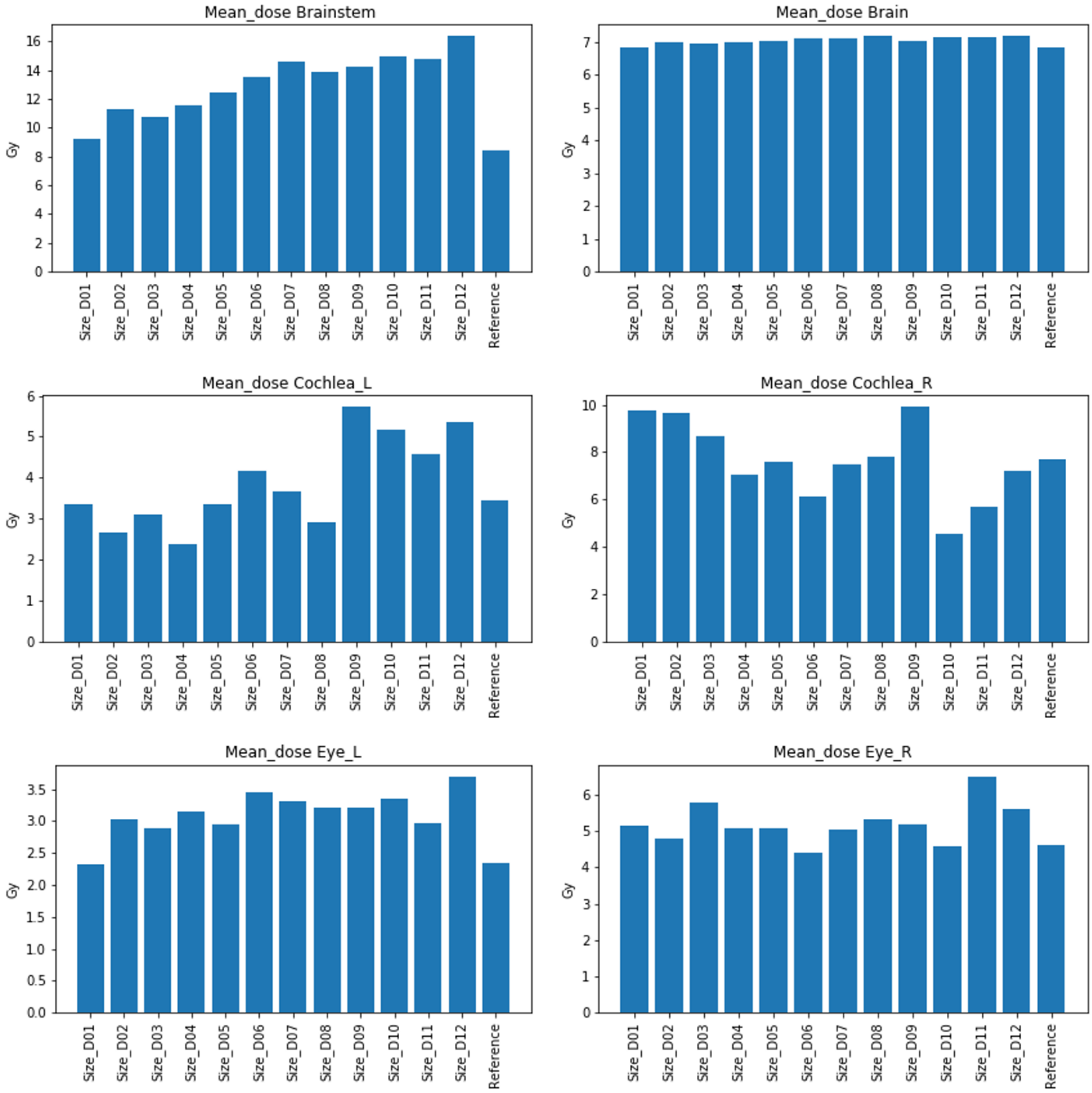

Figure 108: Bar plots of the mean dose of the brain, brainstem, cochlea and eyes, for the reference plan and the 12 plans including an outlier to the target at the specific sizes at location D as displayed in Figure 58.

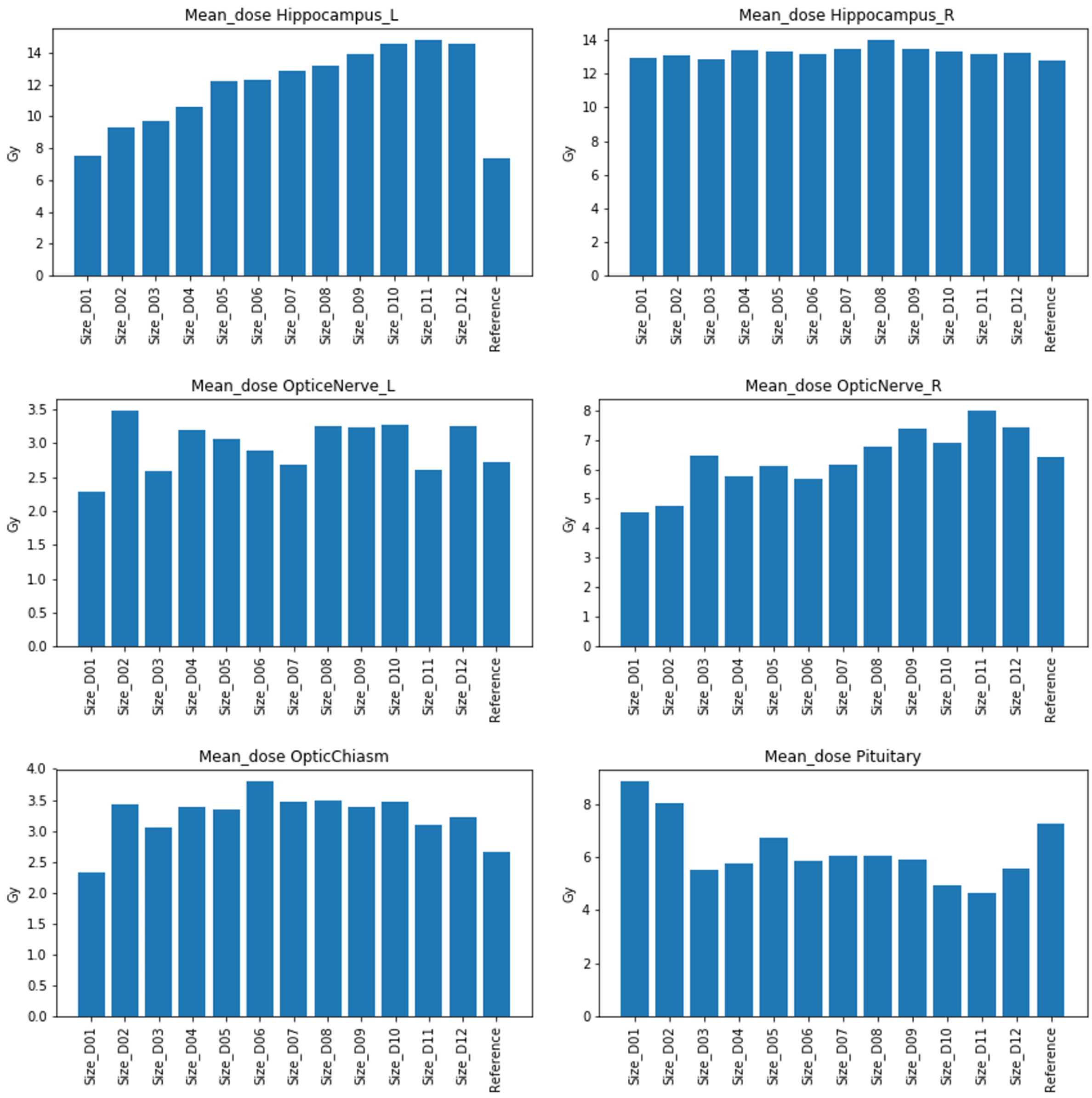

Figure 109: Bar plots of the mean dose of the hippocampi, optic nerves, optic chiasm and pituitary, for the reference plan and the 12 plans including an outlier to the target at the specific sizes at location D as displayed in Figure 58.

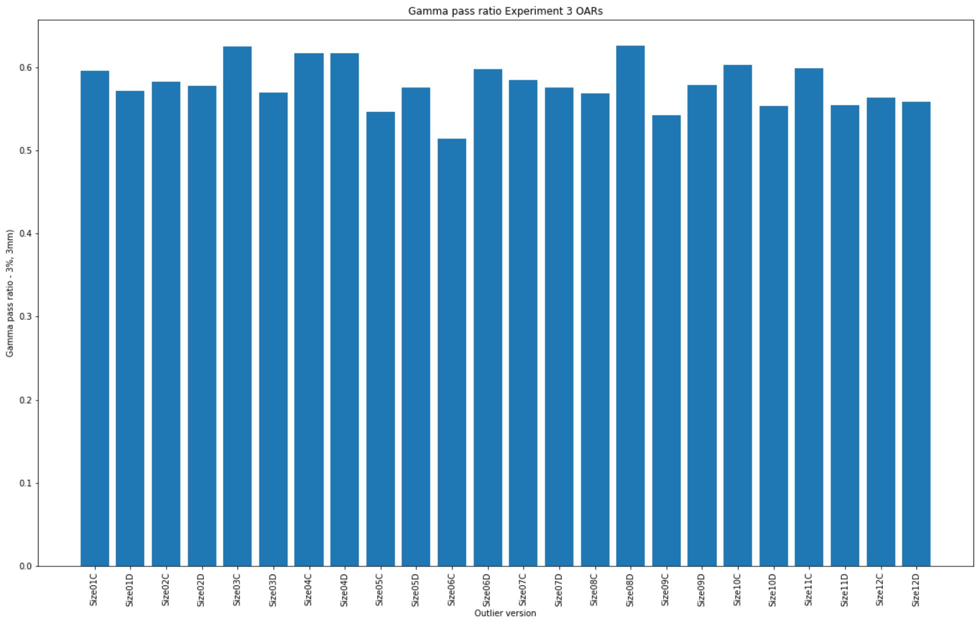

Figure 110: Bar plot of the Gamma pass ratio of each experimental plan containing an outlier at a specific size and location according to Figure 58 . The plans considered the OARs and the pass ratio is determined with respect to the reference plan. The criteria for the gamma pass rate were set to 3% and 3 mm.

Experiment 4: Outliers relative size to PTV, no OARs involved:

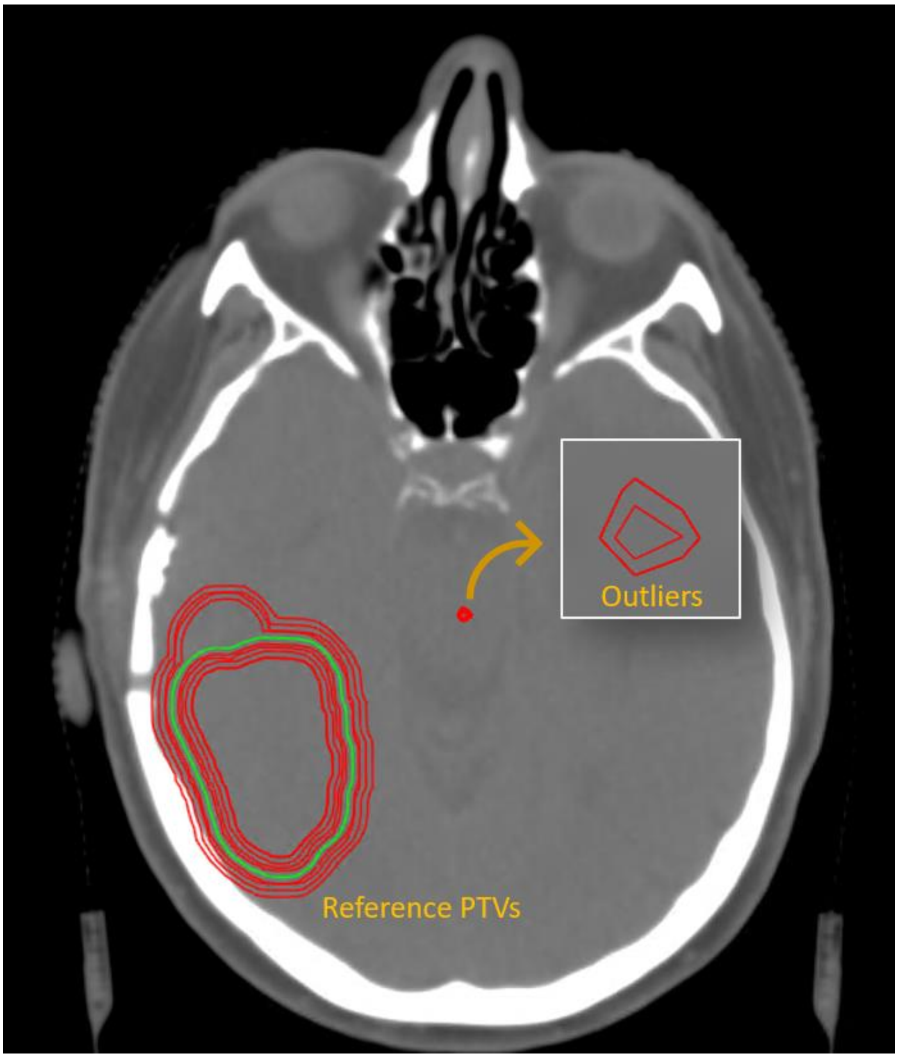

Figure 111: Overview of the synthetic setup of experiment 4 to determine the effect of outlier size relative to the PTV size. The smallest two outliers from location A from experiment 3 were used as outlier volumes. In this case the reference PTV (depicted in green) was increased and decreased incrementally with 1 mm margins. The yellow arrow indicates the location of the zoomed area.

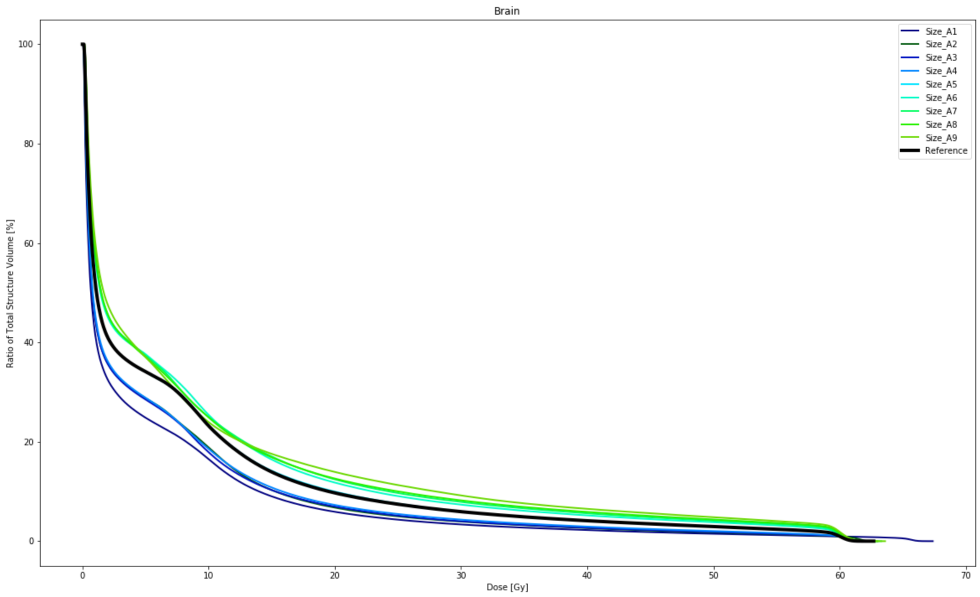

Figure 112: DVH curves of the brain structure of the reference plan and the 9 plans including different size reference target including the outlier A1 the specific location as displayed in Figure 111.

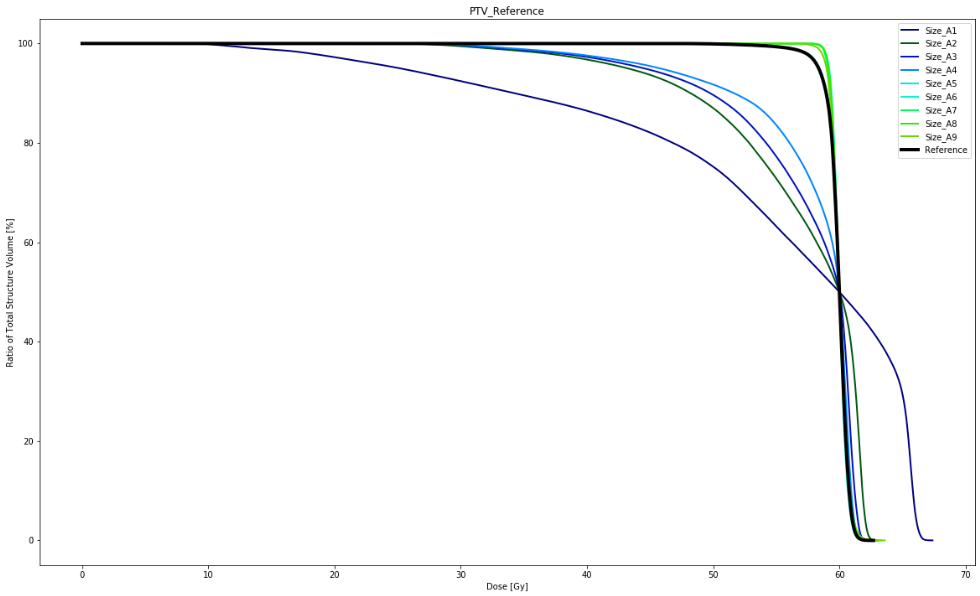

Figure 113: DVH curves of the reference PTV structure of the reference plan and the 9 plans including different size reference target including the outlier A1 the specific location as displayed in Figure 111. It has to be noted that the plans are defined on smaller and larger PTVs then the reference PTV where the DVH curves are shown in this graph.

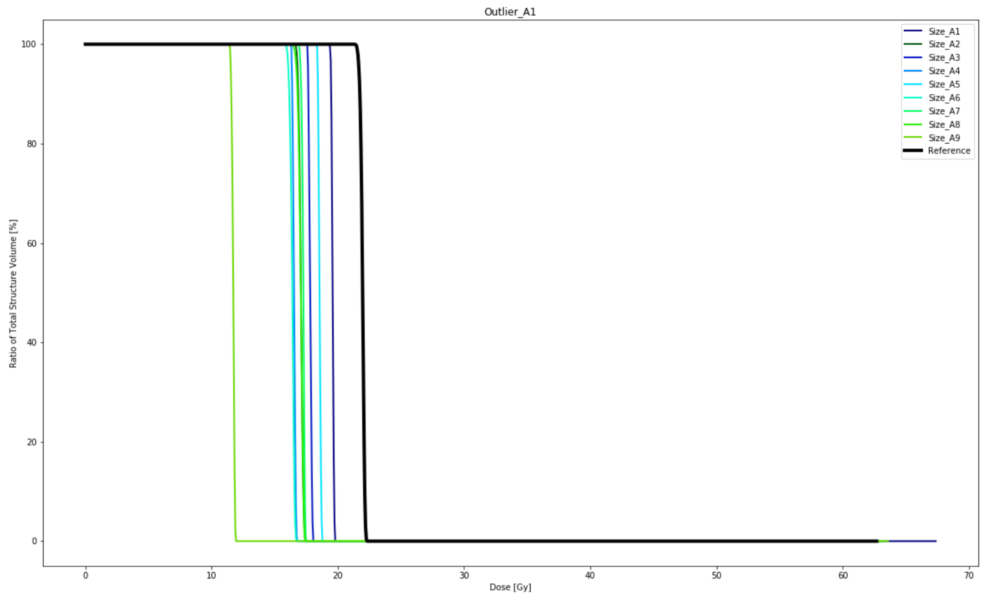

Figure 114: DVH curves of the outlier volume A1 for the reference plan and the 9 plans including different size reference target including the outlier A1 the specific location as displayed in Figure 111.

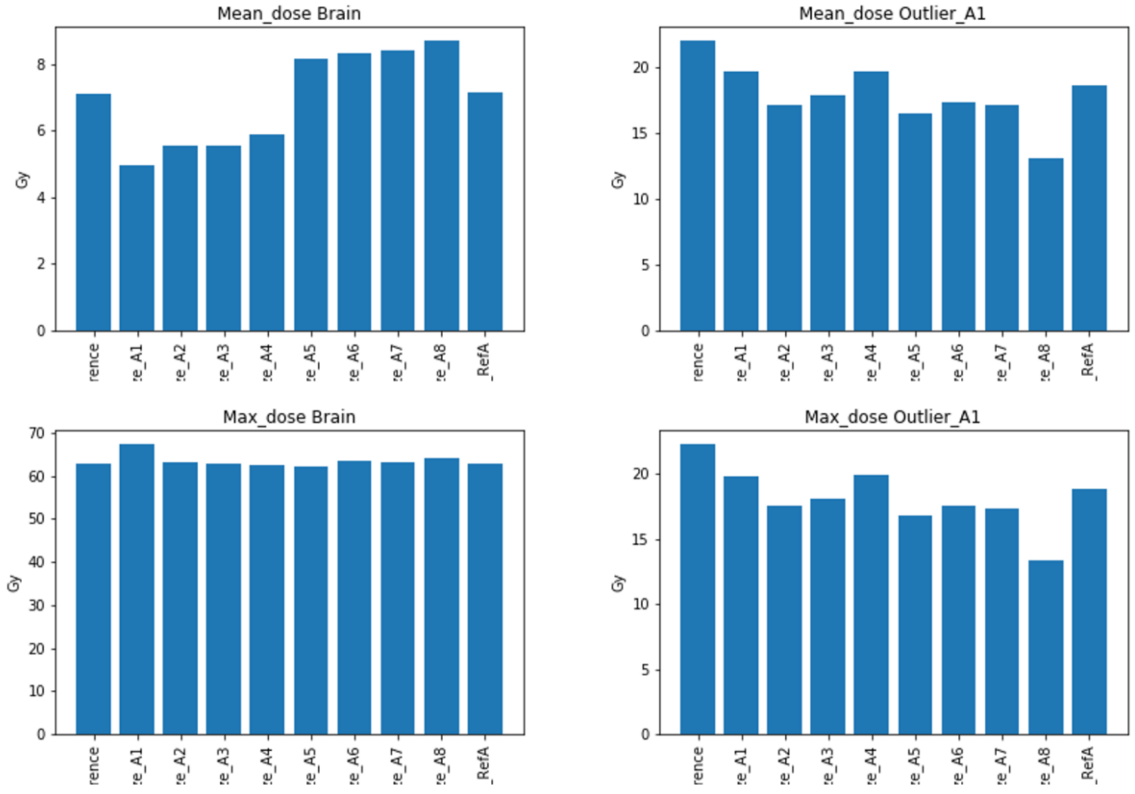

Figure 115: Box plots oft he mean and maximum dose of the brain structure (left) and the Outlier volume A1 (right) for the reference plan and the 9 plans including different size reference target including the outlier A1 the specific location as displayed in Figure 111.

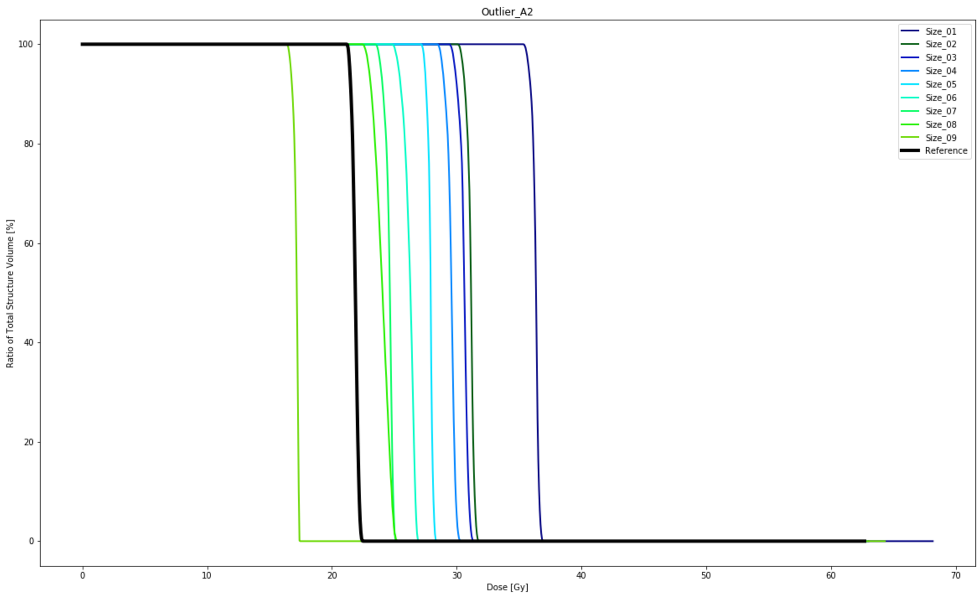

Figure 116: DVH curves of the outlier volume A2 for the reference plan and the 9 plans including different size reference target including the outlier A2 the specific location as displayed in Figure 111.

Experiment 4: Outliers relative size to PTV, with OARs involved, outlier A1:

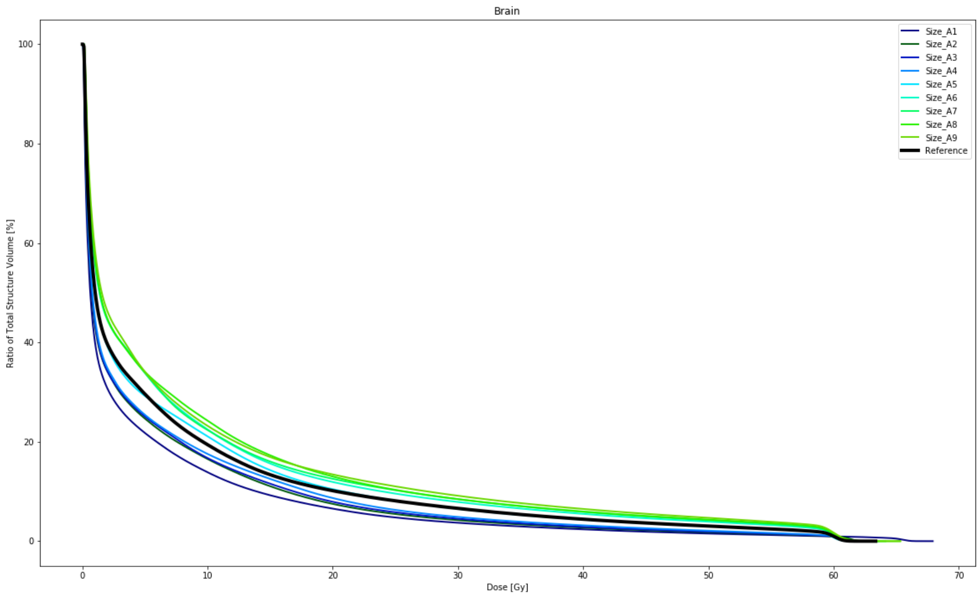

Figure 117: DVH curves of the brain structure of the reference plan and the 9 plans including different size reference target including the outlier A1 the specific location as displayed in Figure 111.

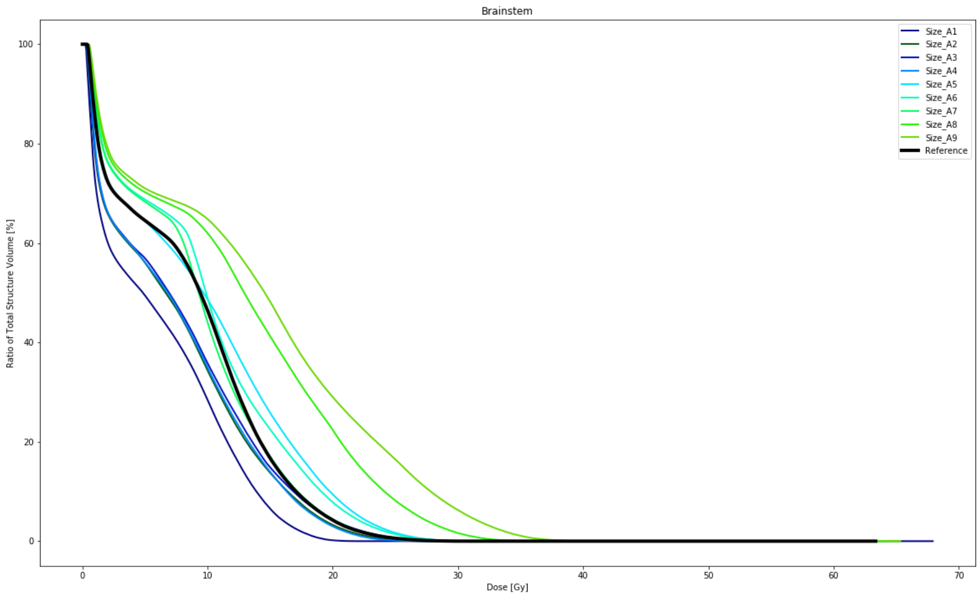

Figure 118: DVH curves of the brainstem structure of the reference plan and the 9 plans including different size reference target including the outlier A1 the specific location as displayed in Figure 111.

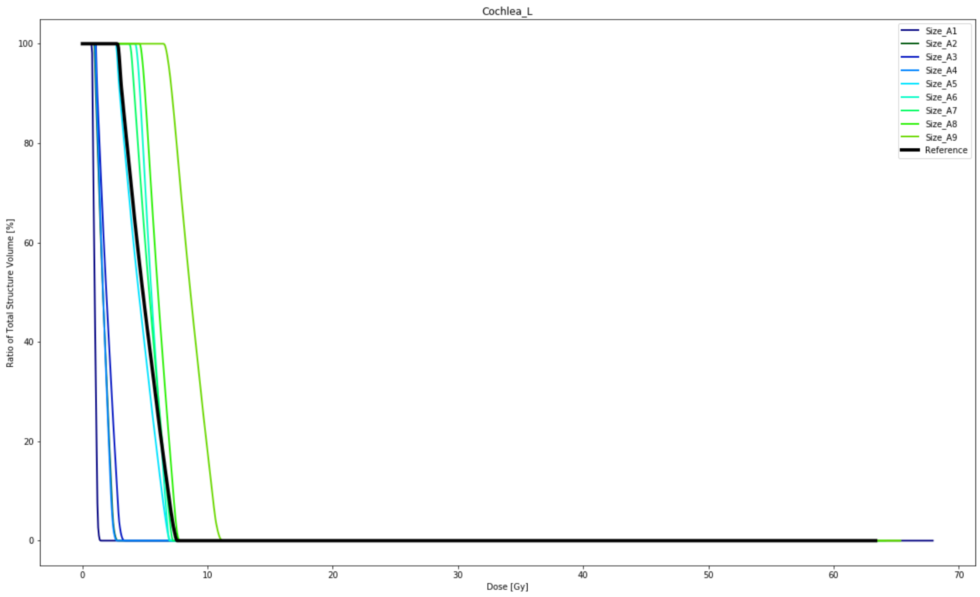

Figure 119: DVH curves of the left cochlea structure of the reference plan and the 9 plans including different size reference target including the outlier A1 the specific location as displayed in Figure 111.

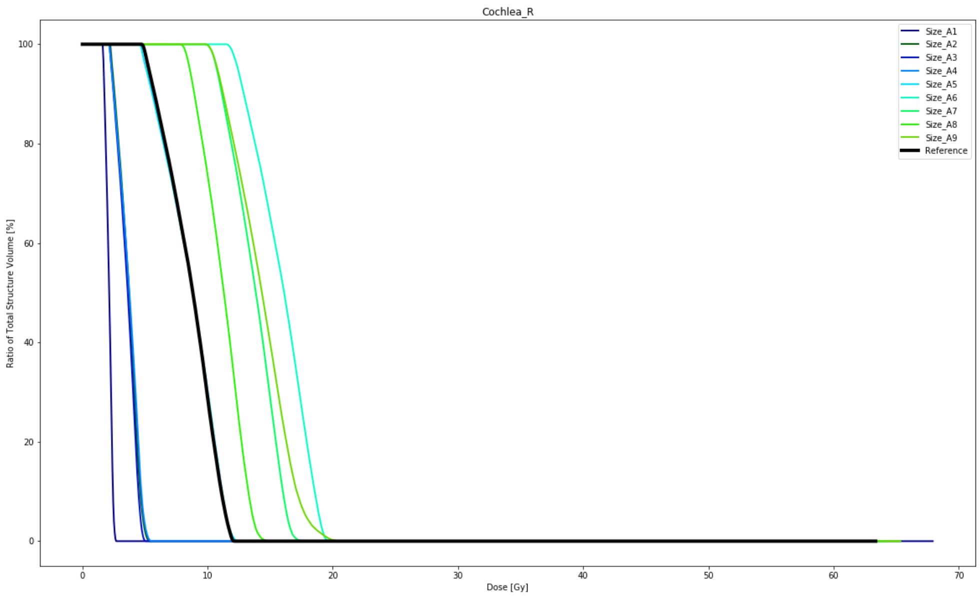

Figure 120: DVH curves of the right cochlea structure of the reference plan and the 9 plans including different size reference target including the outlier A1 the specific location as displayed in Figure 111.

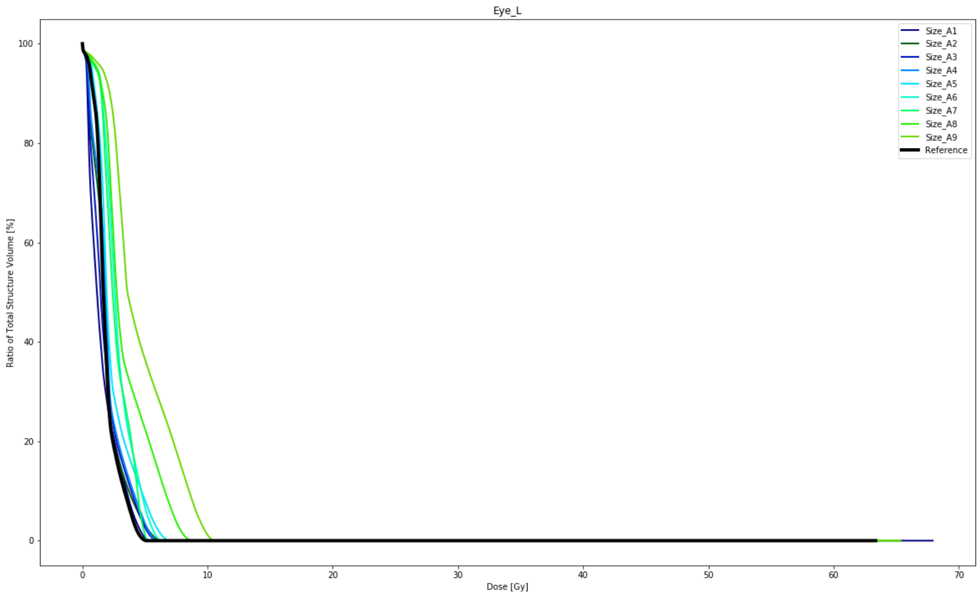

Figure 121: DVH curves of the left eye structure of the reference plan and the 9 plans including different size reference target including the outlier A1 the specific location as displayed in Figure 111.

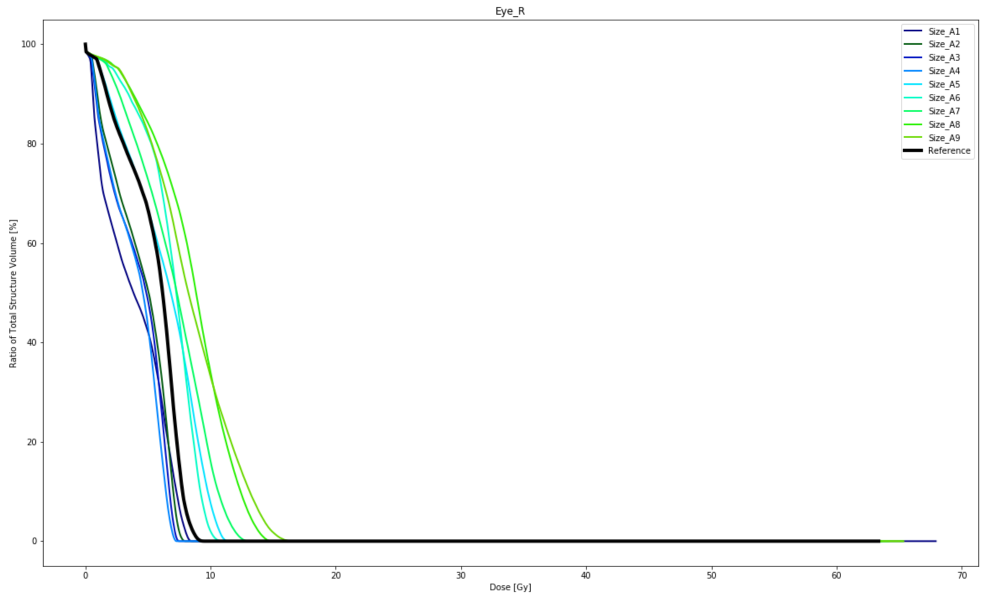

Figure 122: DVH curves of the right eye structure of the reference plan and the 9 plans including different size reference target including the outlier A1 the specific location as displayed in Figure 111.

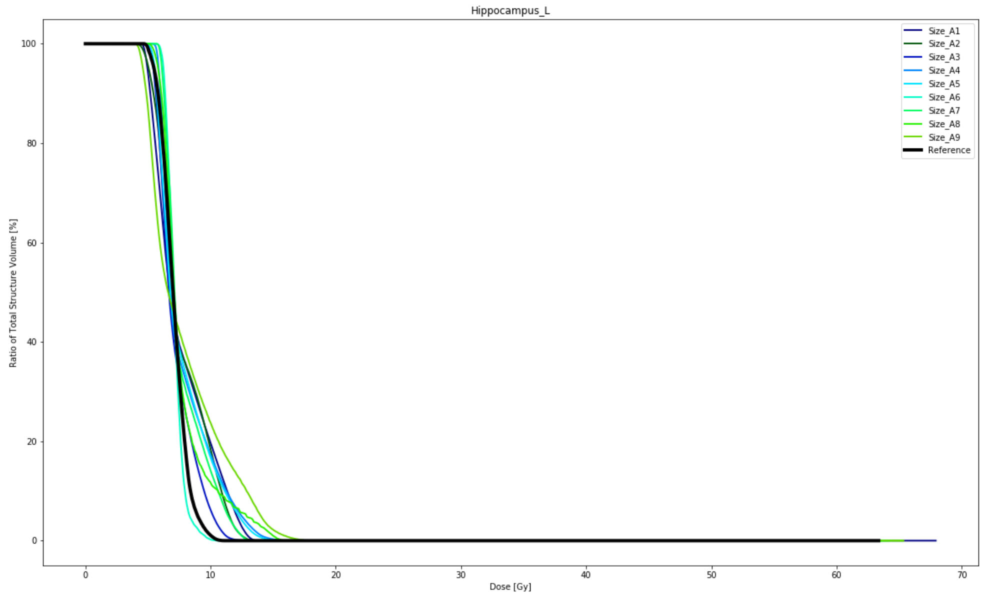

Figure 123: DVH curves of the left hippocampus structure of the reference plan and the 9 plans including different size reference target including the outlier A1 the specific location as displayed in Figure 111.

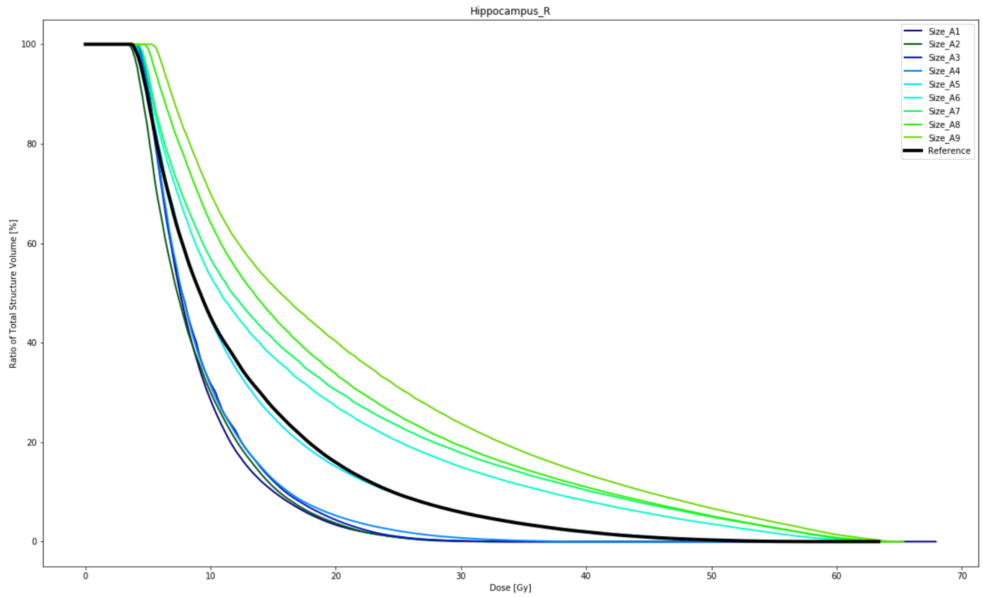

Figure 124: DVH curves of the right hippocampus structure of the reference plan and the 9 plans including different size reference target including the outlier A1 the specific location as displayed in Figure 111.

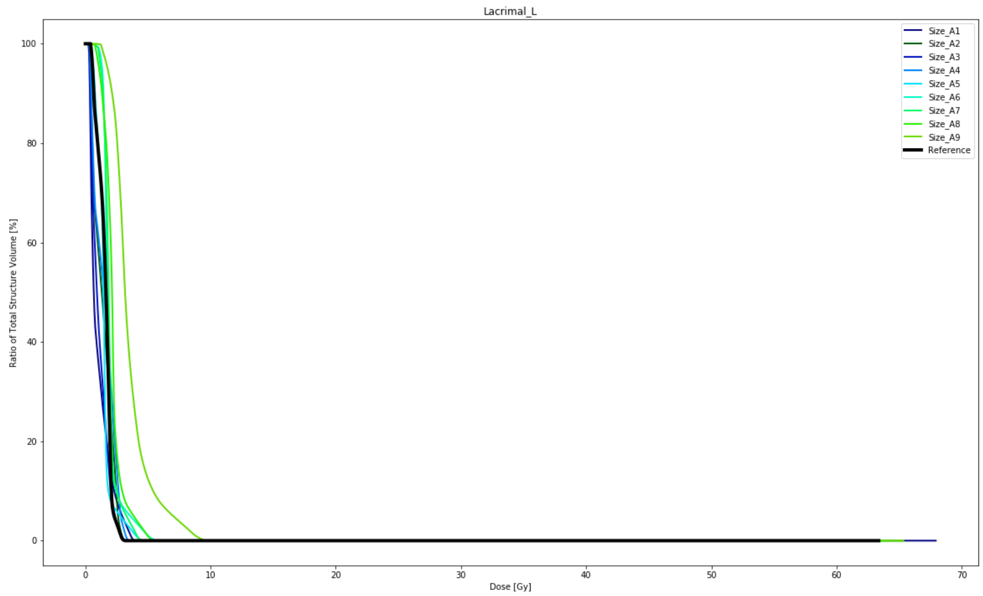

Figure 125: DVH curves of the left lacrimal gland structure of the reference plan and the 9 plans including different size reference target including the outlier A1 the specific location as displayed in Figure 111.

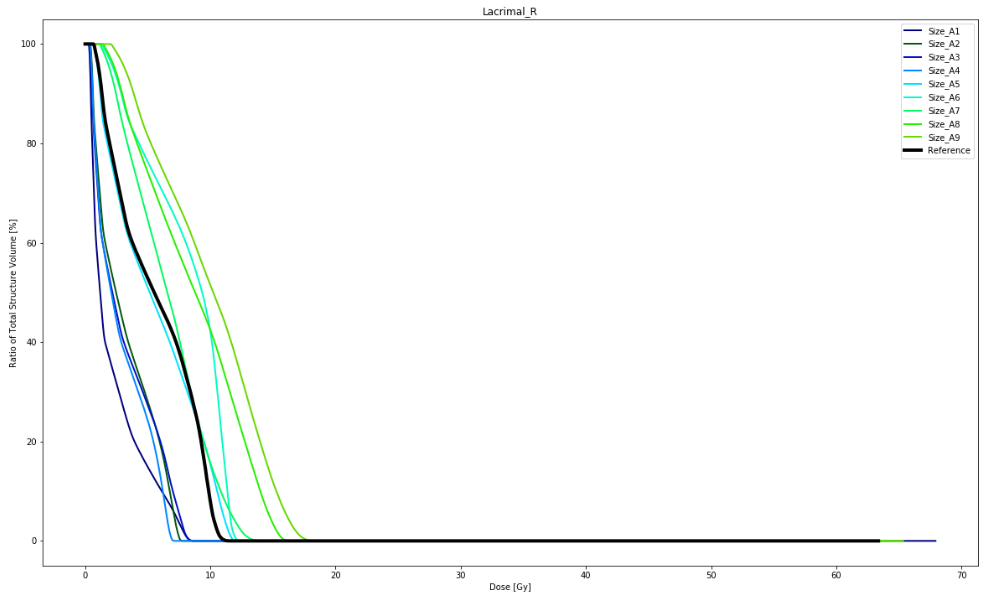

Figure 126: DVH curves of the right lacrimal gland structure of the reference plan and the 9 plans including different size reference target including the outlier A1 the specific location as displayed in Figure 111.

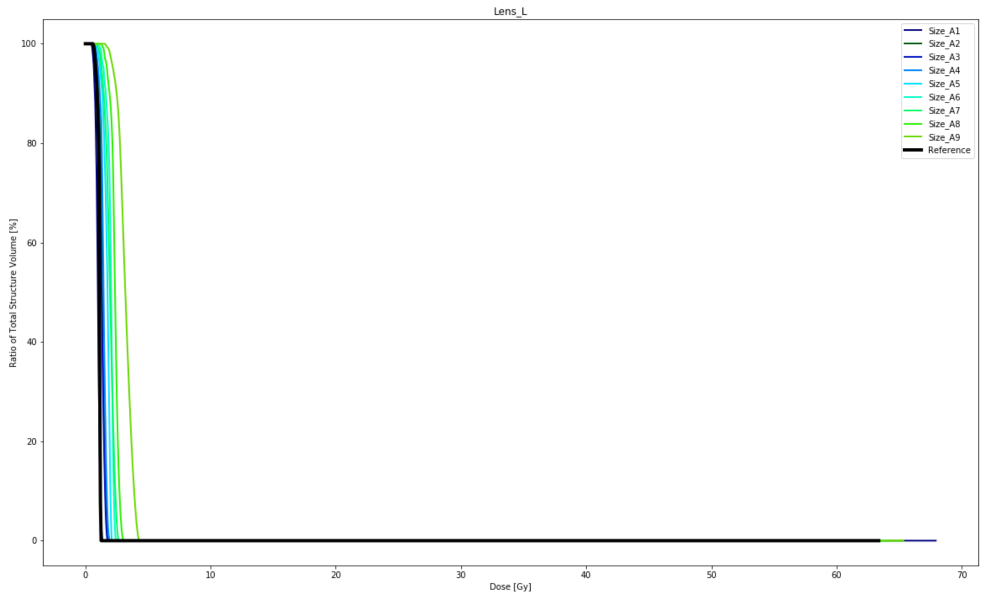

Figure 127: DVH curves of the left lens structure of the reference plan and the 9 plans including different size reference target including the outlier A1 the specific location as displayed in Figure 111.

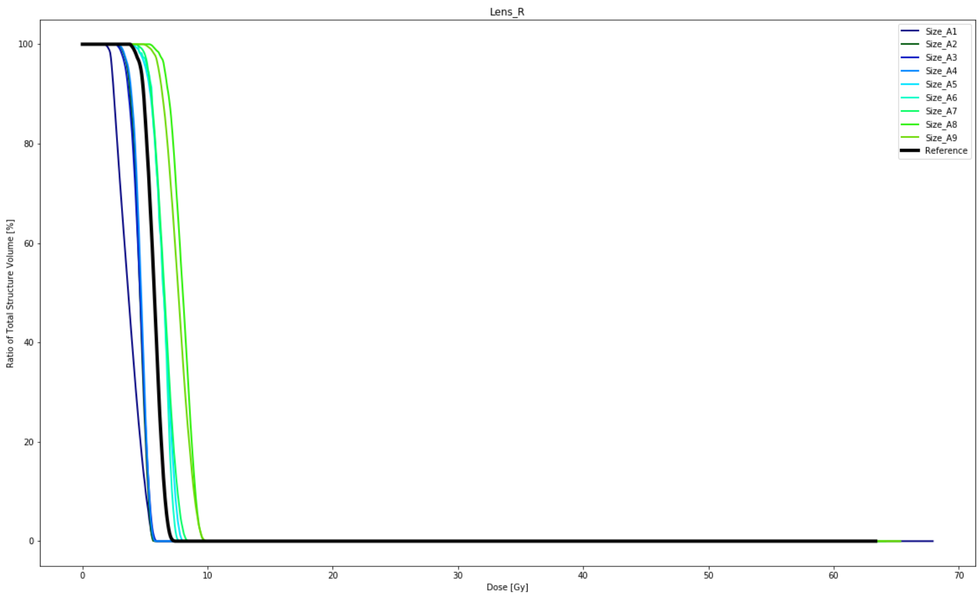

Figure 128: DVH curves of the right lens structure of the reference plan and the 9 plans including different size reference target including the outlier A1 the specific location as displayed in Figure 111.

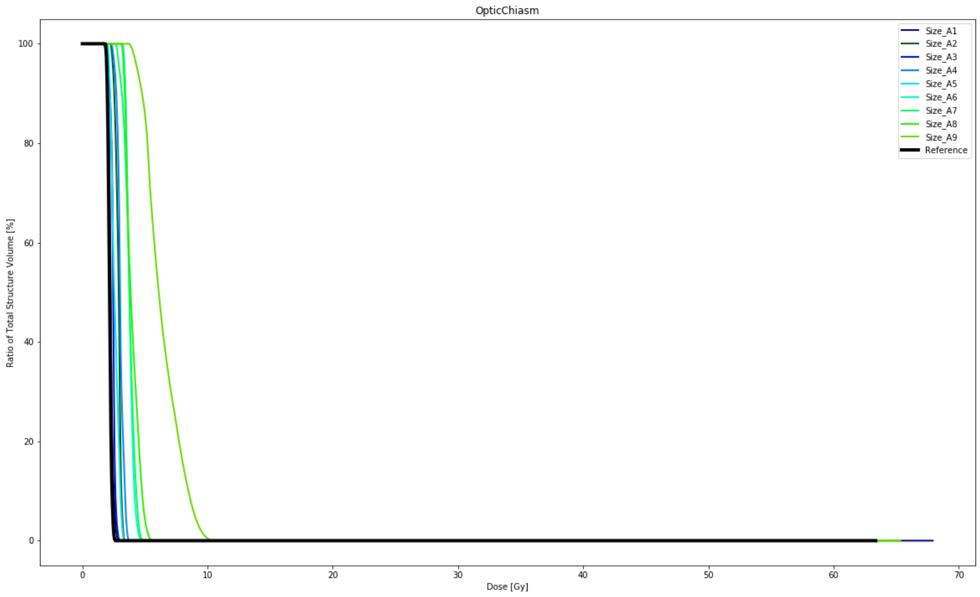

Figure 129: DVH curves of the optic chiasm structure of the reference plan and the 9 plans including different size reference target including the outlier A1 the specific location as displayed in Figure 111.

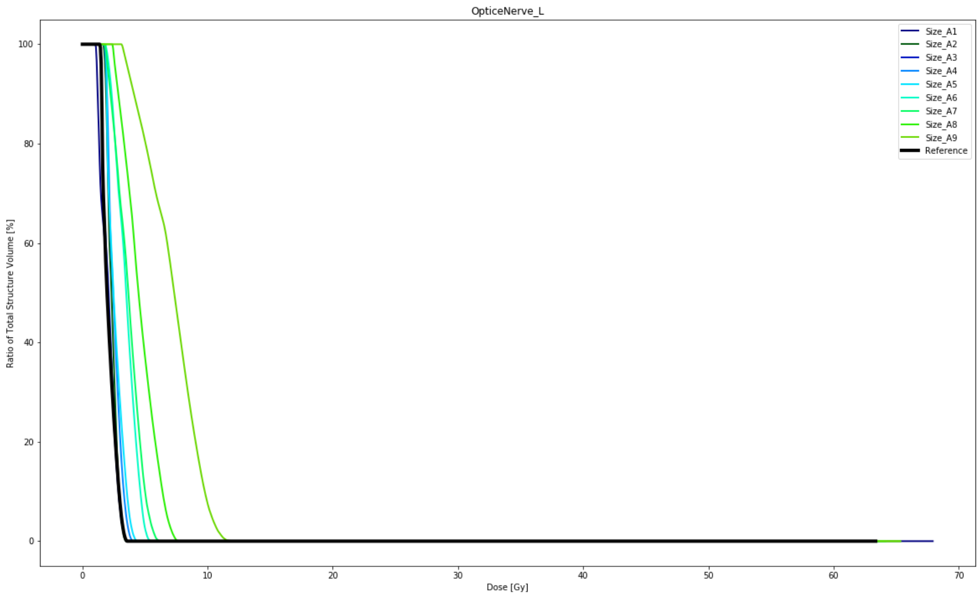

Figure 130: DVH curves of the left optic nerve structure of the reference plan and the 9 plans including different size reference target including the outlier A1 the specific location as displayed in Figure 111.

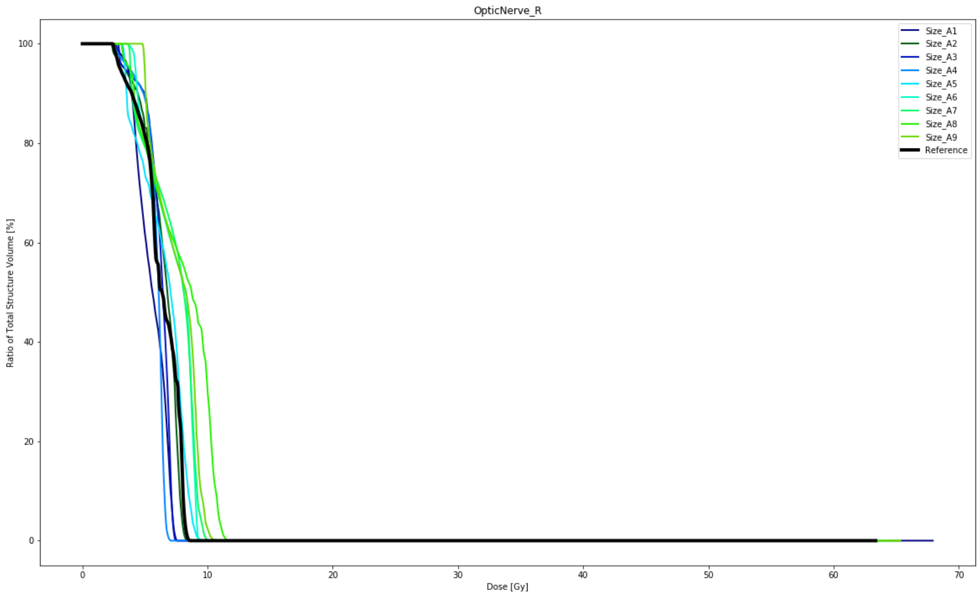

Figure 131: DVH curves of the right optic nerve structure of the reference plan and the 9 plans including different size reference target including the outlier A1 the specific location as displayed in Figure 111.

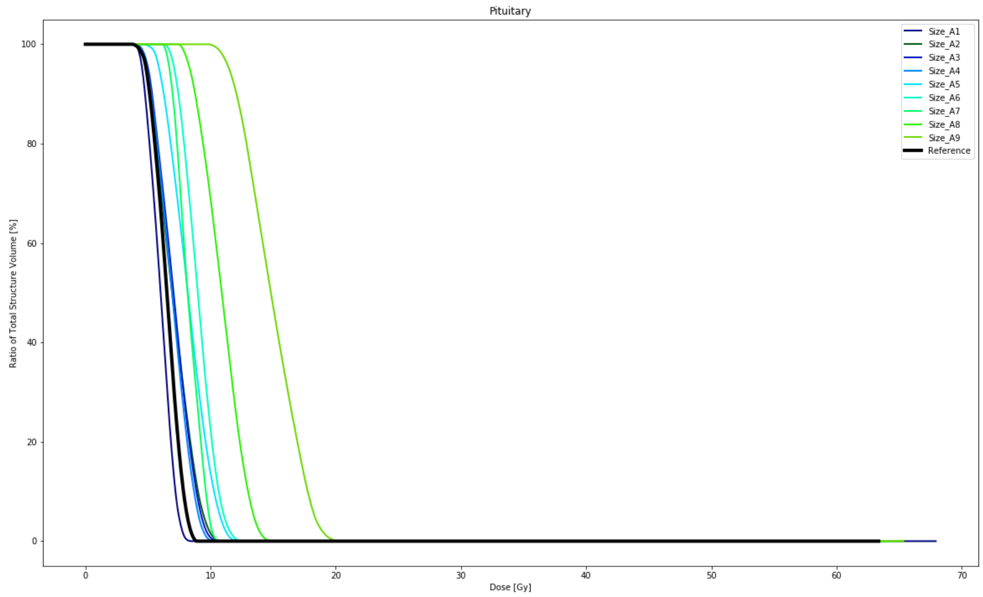

Figure 132: DVH curves of the pituitary gland structure of the reference plan and the 9 plans including different size reference target including the outlier A1 the specific location as displayed in Figure 111.

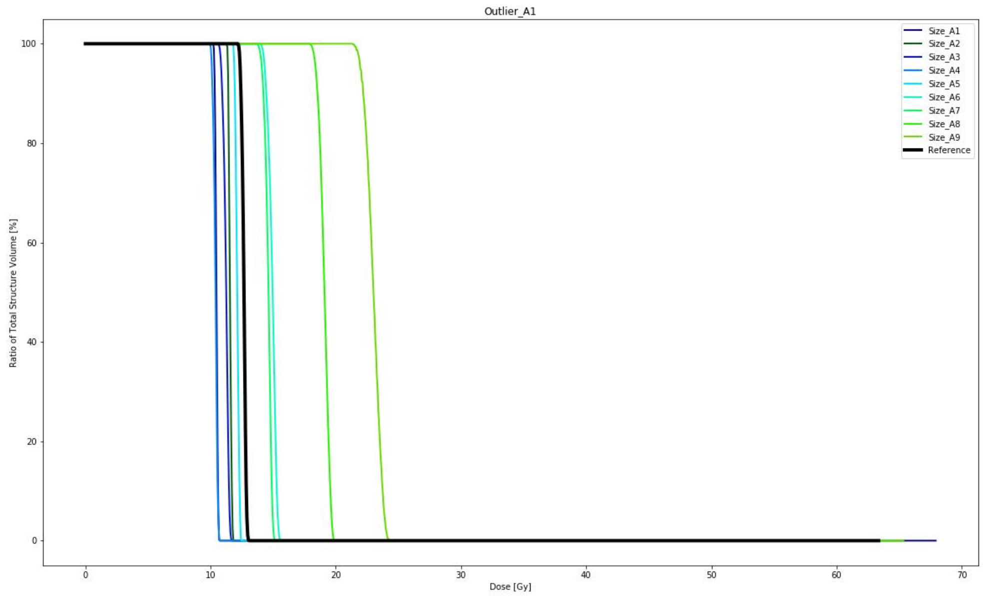

Figure 133: DVH curves of the Outlier volume A1 structure of the reference plan and the 9 plans including different size reference target including the outlier A1 the specific location as displayed in Figure 111.

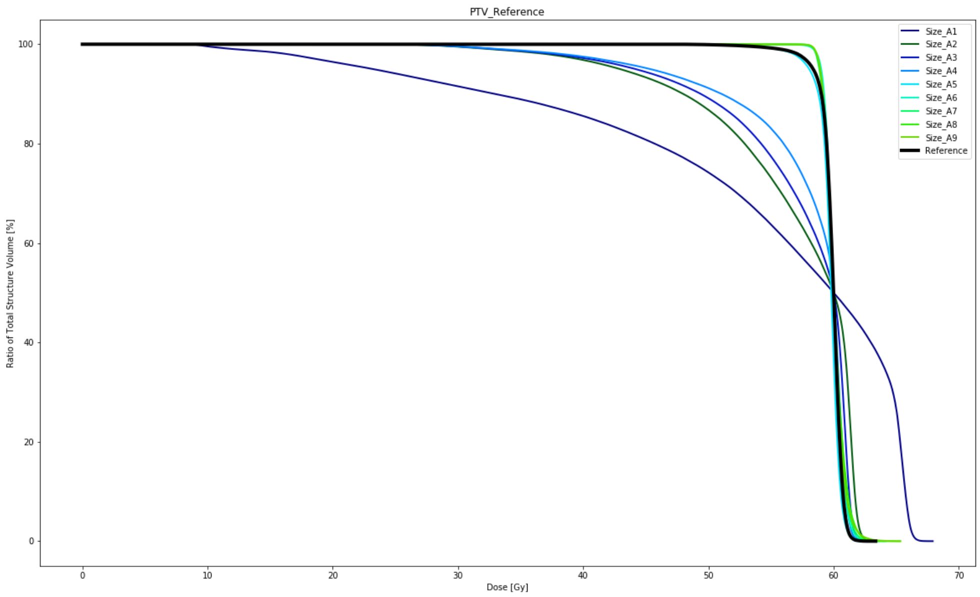

Figure 134: DVH curves of the reference PTV structure of the reference plan and the 9 plans including different size reference target including the outlier A1 the specific location as displayed in Figure 111. It has to be noted that the plans are defined on smaller and larger PTVs then the reference PTV where the DVH curves are shown in this graph.

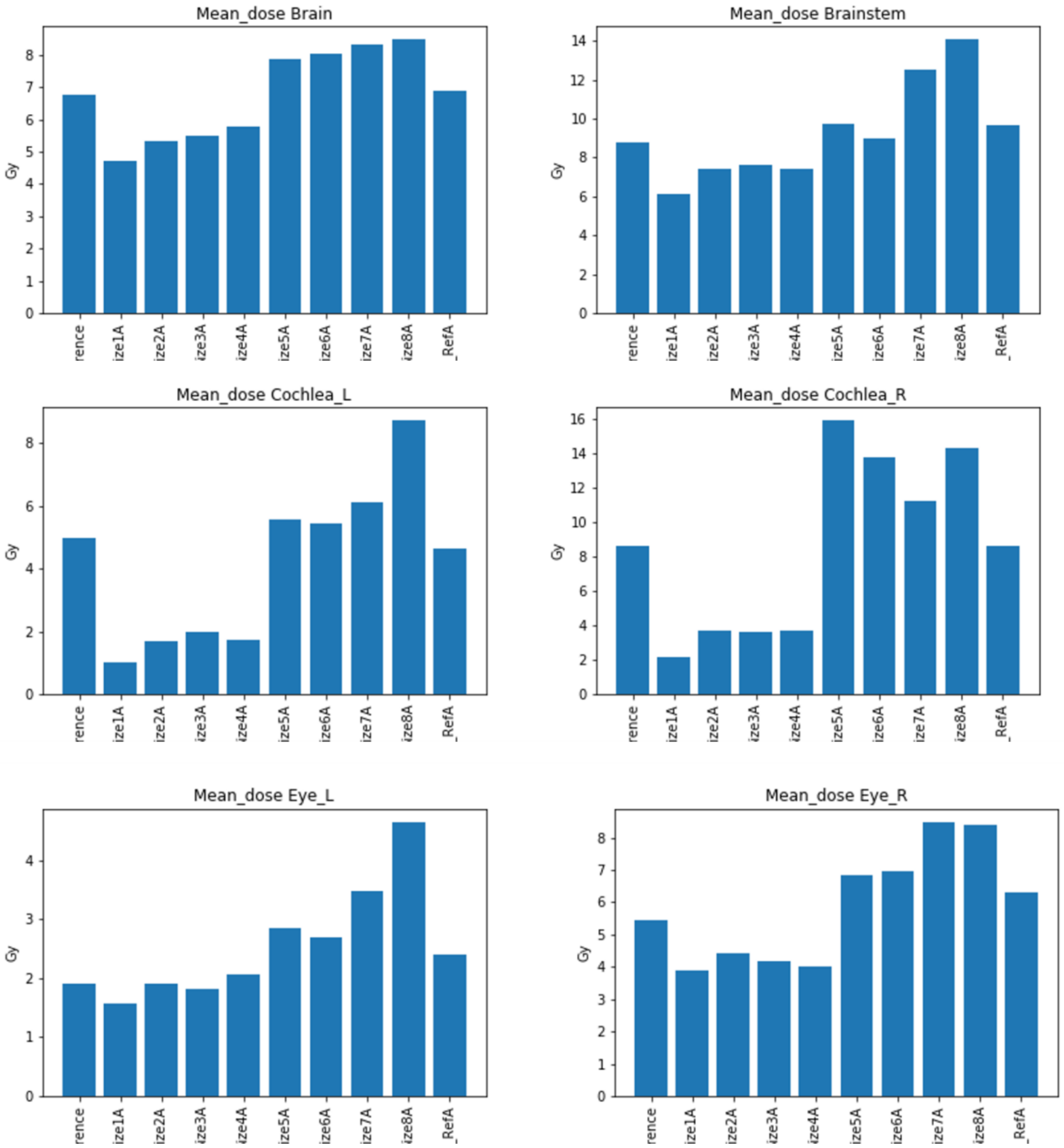

Figure 135: Bar plots of the mean dose of the brain, brainstem, cochlea and eyes, for the reference plan and the 9 plans including different size reference target including the outlier A1 the specific location as displayed in Figure 111

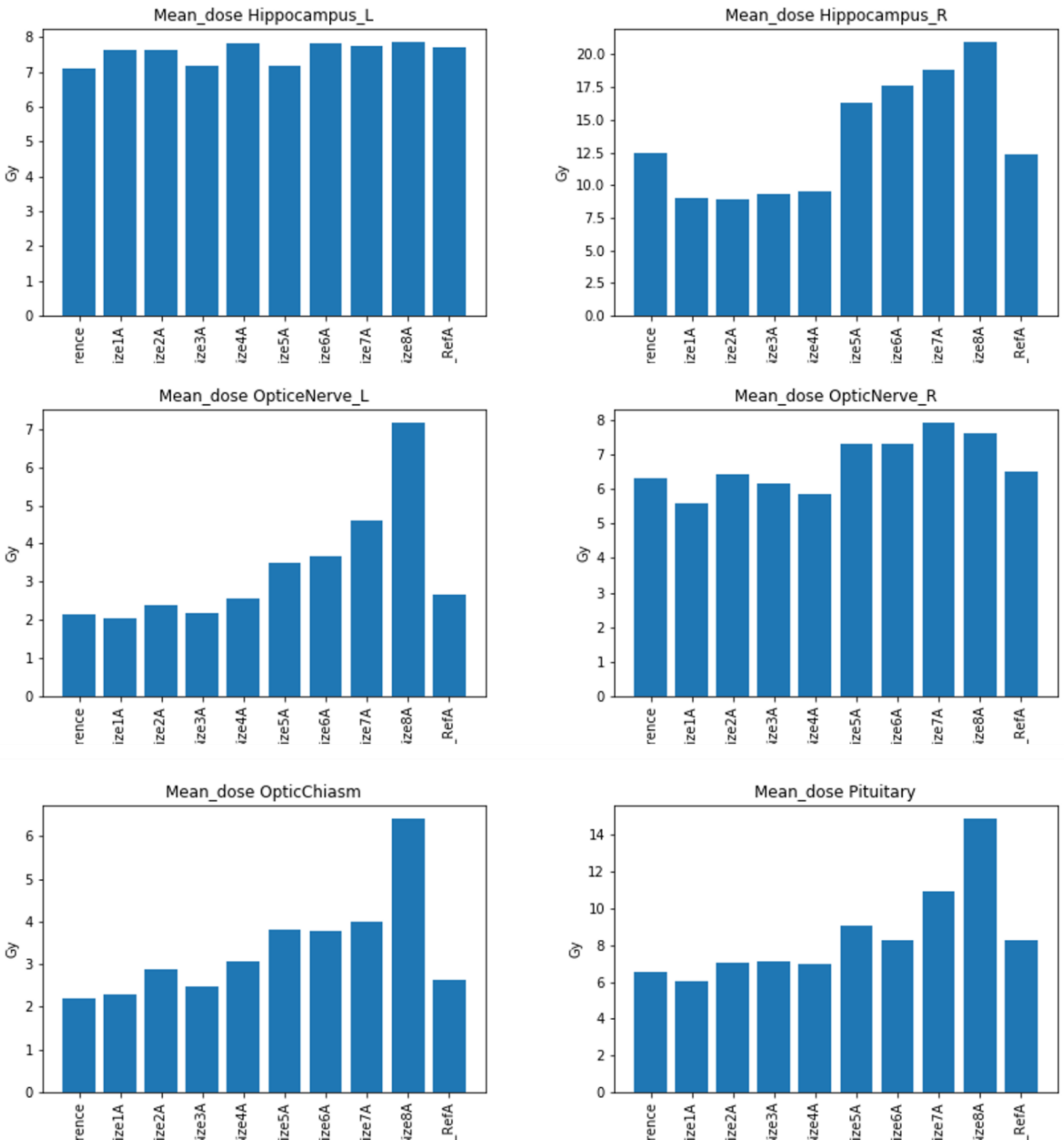

Figure 136: Bar plots of the mean dose of the hippocampi, optic nerves, optic chiasm and pituitary gland, for the reference plan and the 9 plans including different size reference target including the outlier A1 the specific location as displayed in Figure 111.

Experiment 4: Outliers relative size to PTV, with OARs involved, outlier A2:

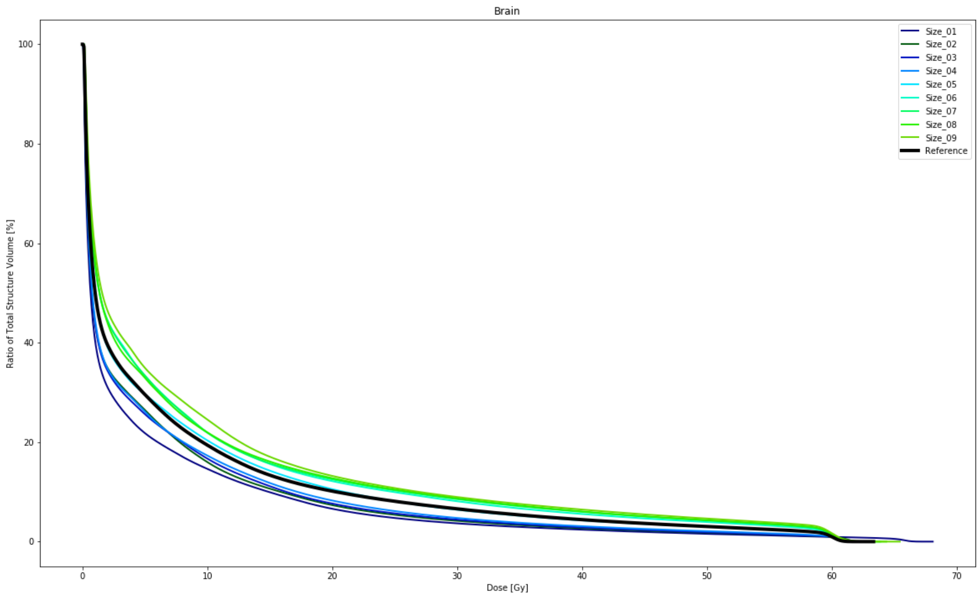

Figure 137: DVH curves of the brain structure of the reference plan and the 9 plans including different size reference target including the outlier A2 the specific location as displayed in Figure 111.

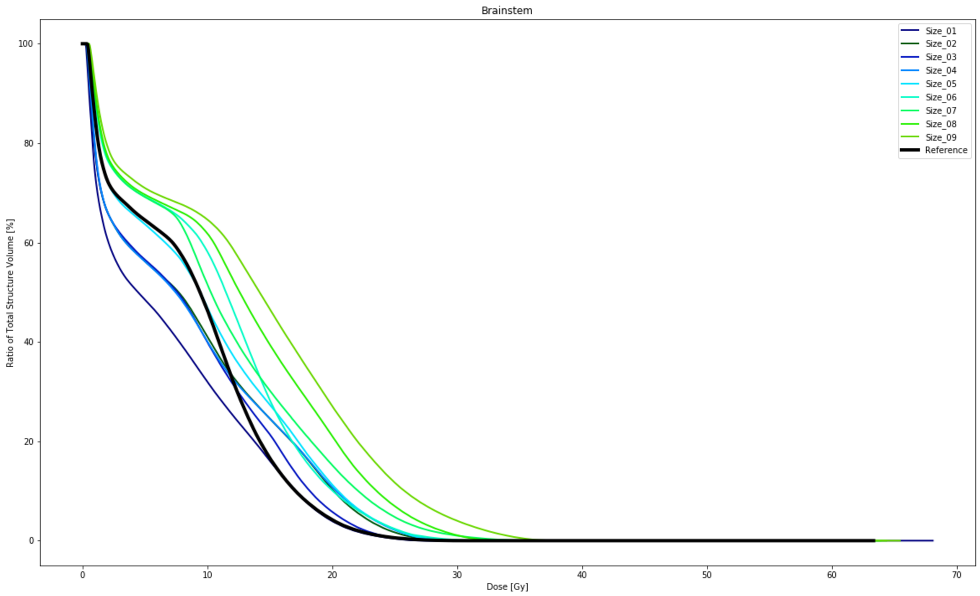

Figure 138: DVH curves of the brainstem structure of the reference plan and the 9 plans including different size reference target including the outlier A2 the specific location as displayed in Figure 111.

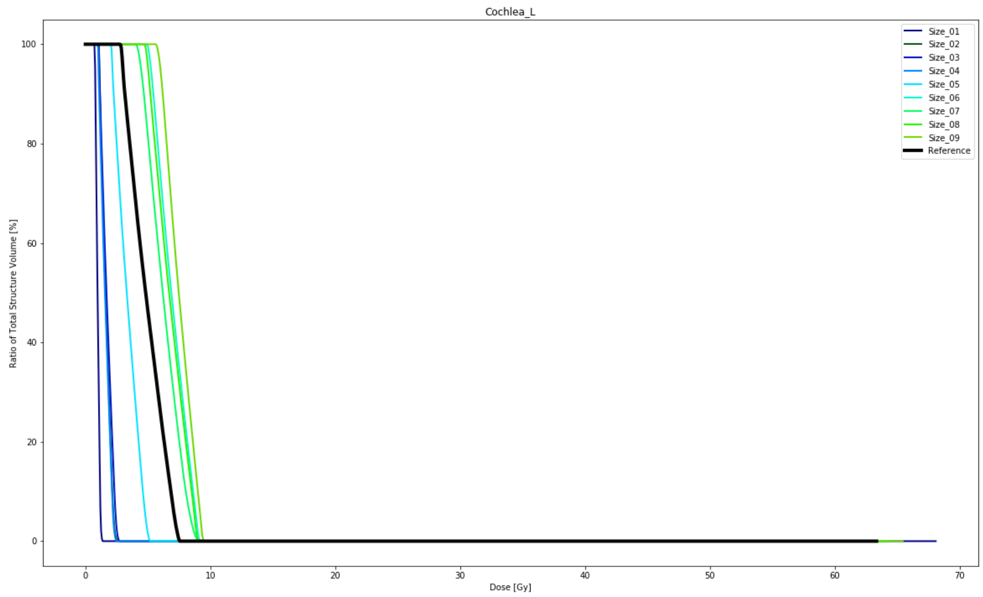

Figure 139: DVH curves of the left cochlea structure of the reference plan and the 9 plans including different size reference target including the outlier A2 the specific location as displayed in Figure 111.

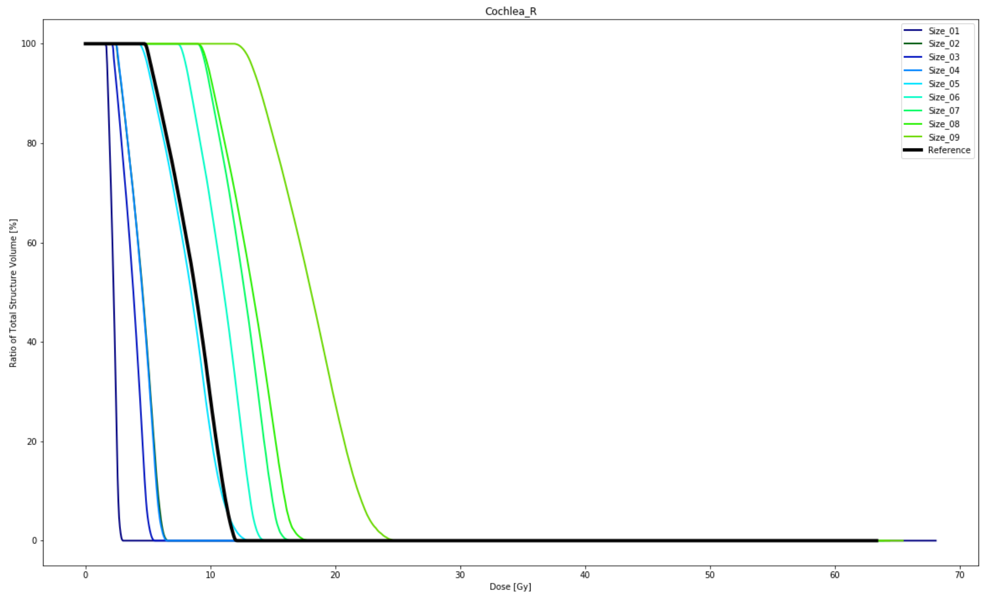

Figure 140: DVH curves of the right cochlea structure of the reference plan and the 9 plans including different size reference target including the outlier A2 the specific location as displayed in Figure 111.

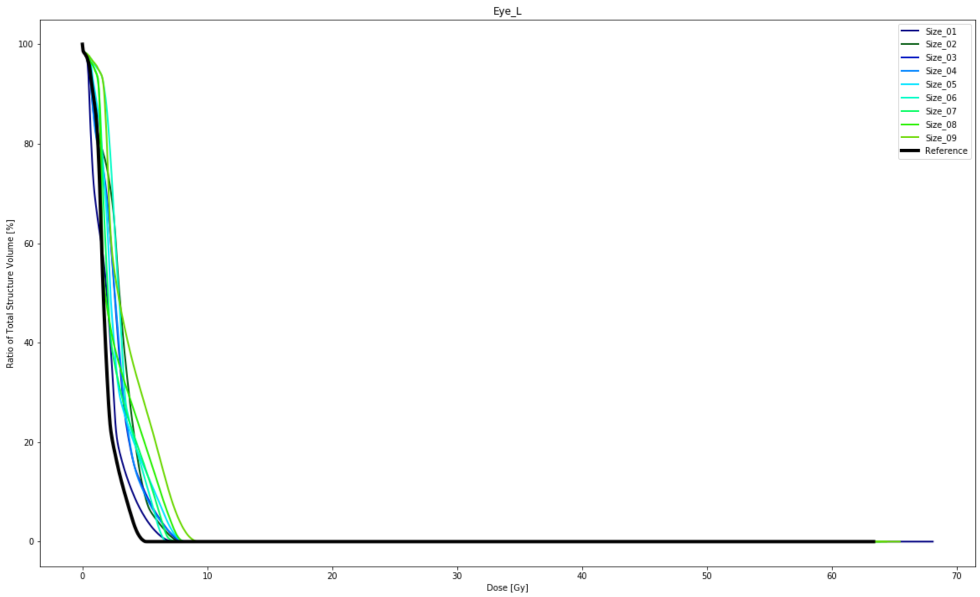

Figure 141: DVH curves of the left eye structure of the reference plan and the 9 plans including different size reference target including the outlier A2 the specific location as displayed in Figure 111.

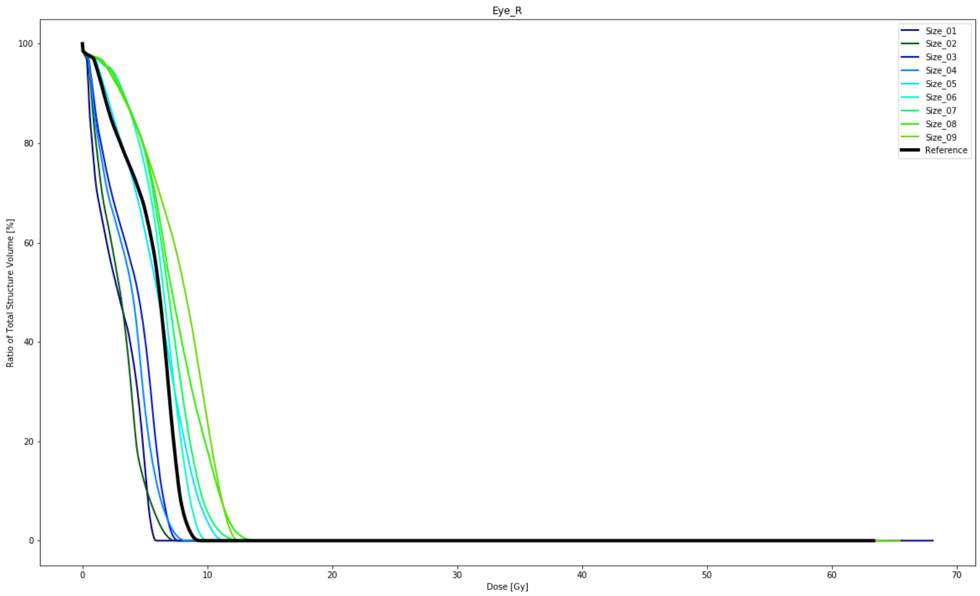

Figure 142: DVH curves of the right eye structure of the reference plan and the 9 plans including different size reference target including the outlier A2 the specific location as displayed in Figure 111.

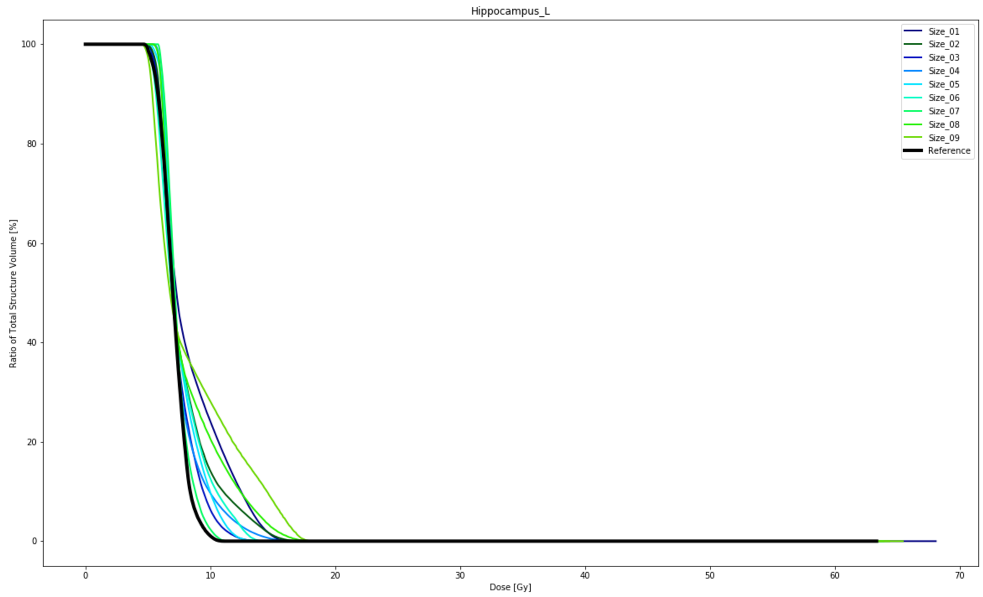

Figure 143: DVH curves of the left hippocampus structure of the reference plan and the 9 plans including different size reference target including the outlier A2 the specific location as displayed in Figure 111.

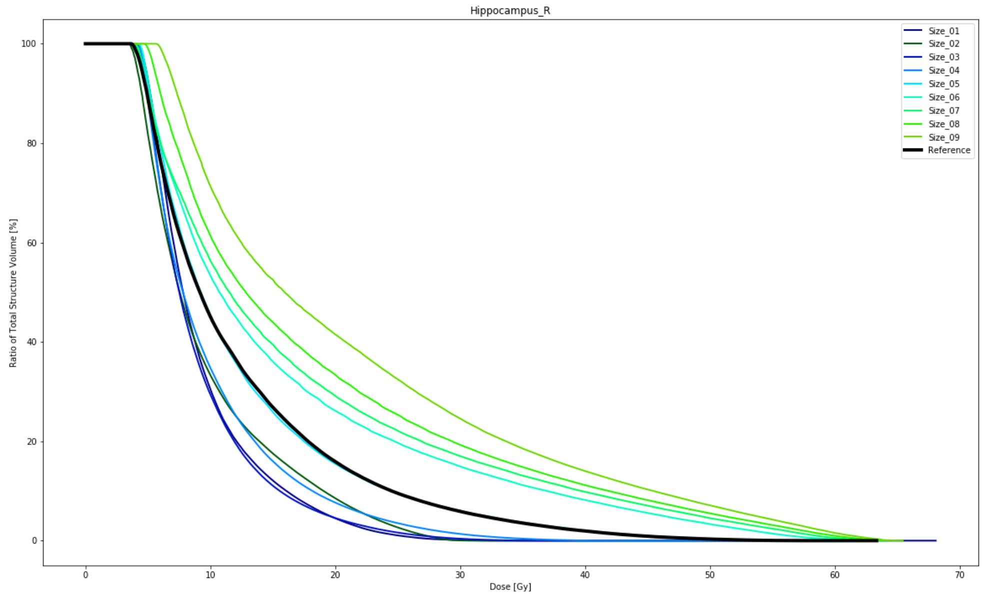

Figure 144: DVH curves of the right hippocampus structure of the reference plan and the 9 plans including different size reference target including the outlier A2 the specific location as displayed in Figure 111.

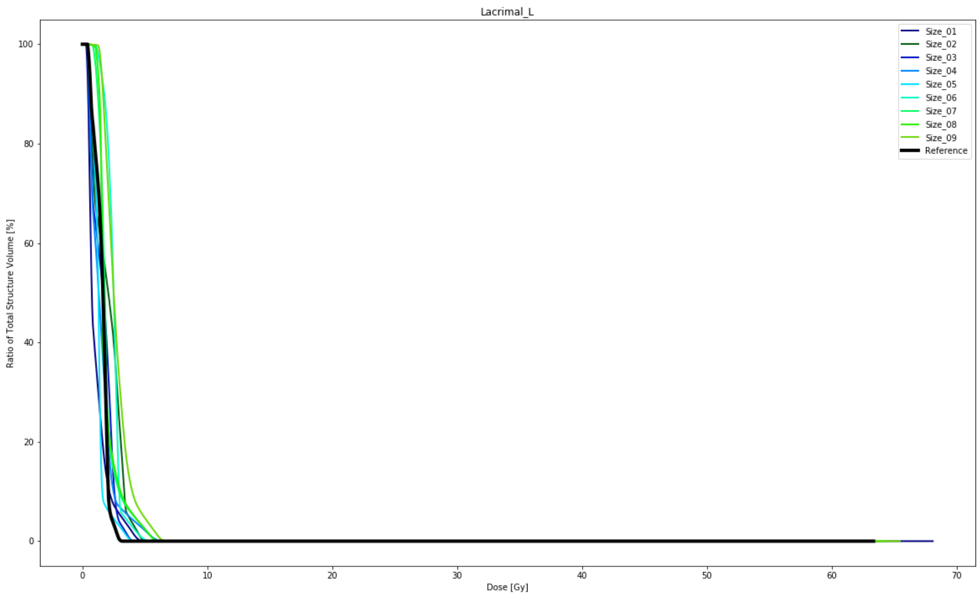

Figure 145: DVH curves of the left lacrimal gland structure of the reference plan and the 9 plans including different size reference target including the outlier A2 the specific location as displayed in Figure 111.

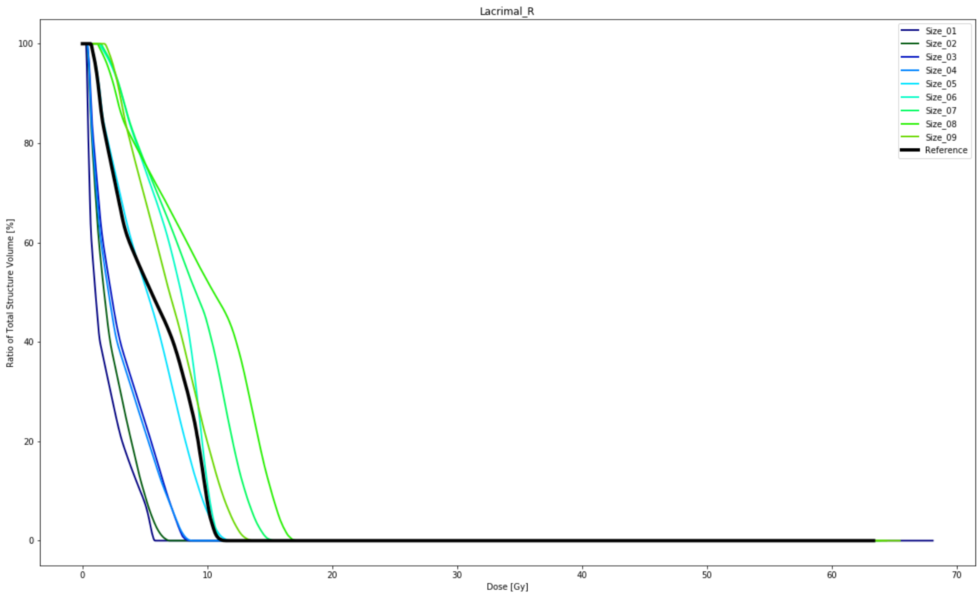

Figure 146: DVH curves of the right lacrimal gland structure of the reference plan and the 9 plans including different size reference target including the outlier A2 the specific location as displayed in Figure 111.

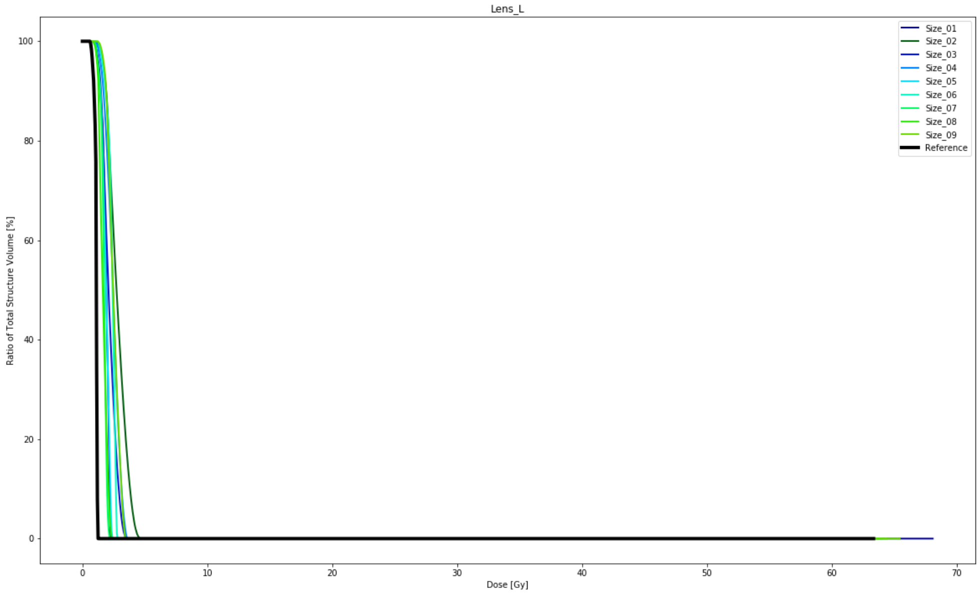

Figure 147: DVH curves of the left lens structure of the reference plan and the 9 plans including different size reference target including the outlier A2 the specific location as displayed in Figure 111.

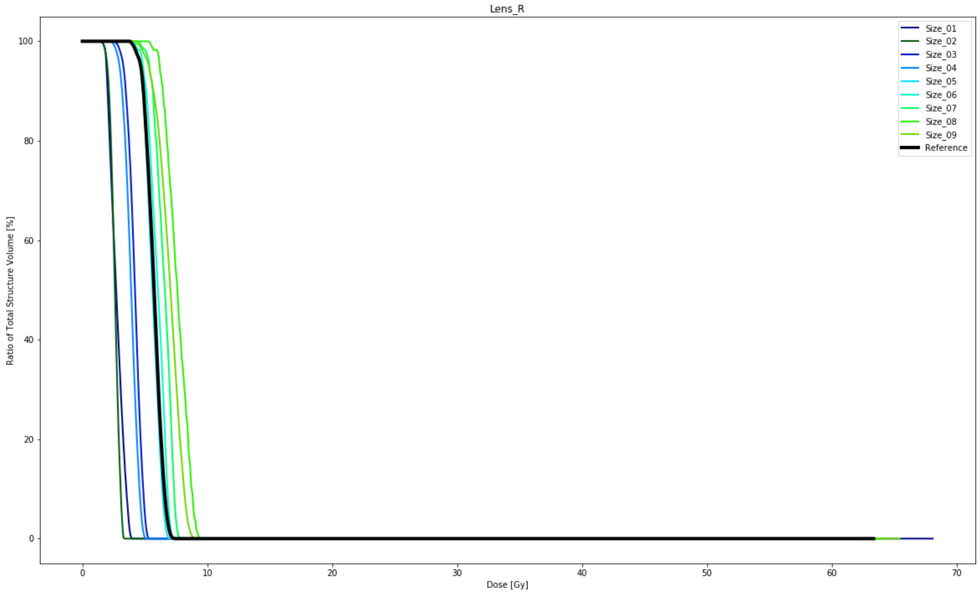

Figure 148: DVH curves of the right lens structure of the reference plan and the 9 plans including different size reference target including the outlier A2 the specific location as displayed in Figure 111.

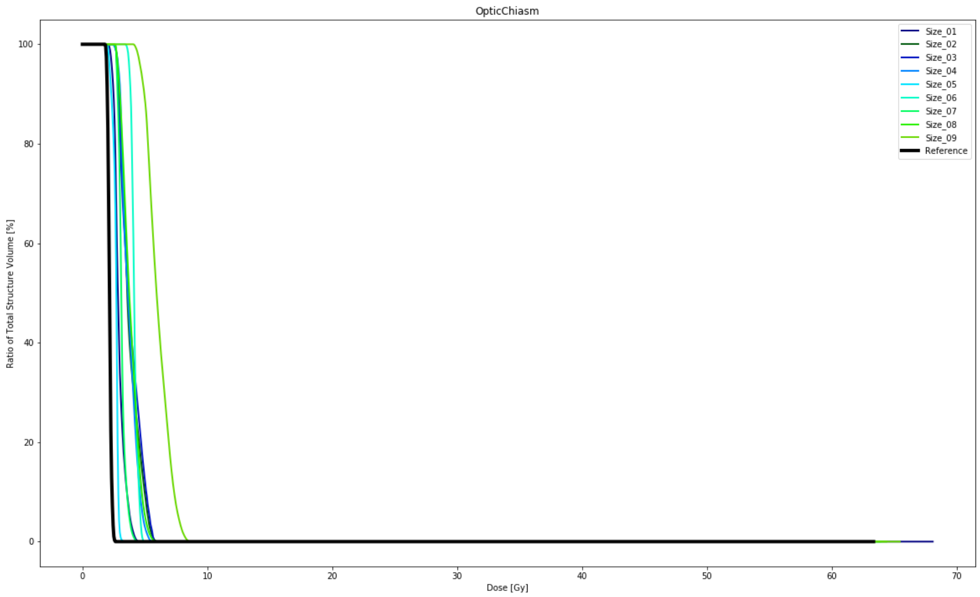

Figure 149: DVH curves of the optic chiasm structure of the reference plan and the 9 plans including different size reference target including the outlier A2 the specific location as displayed in Figure 111.

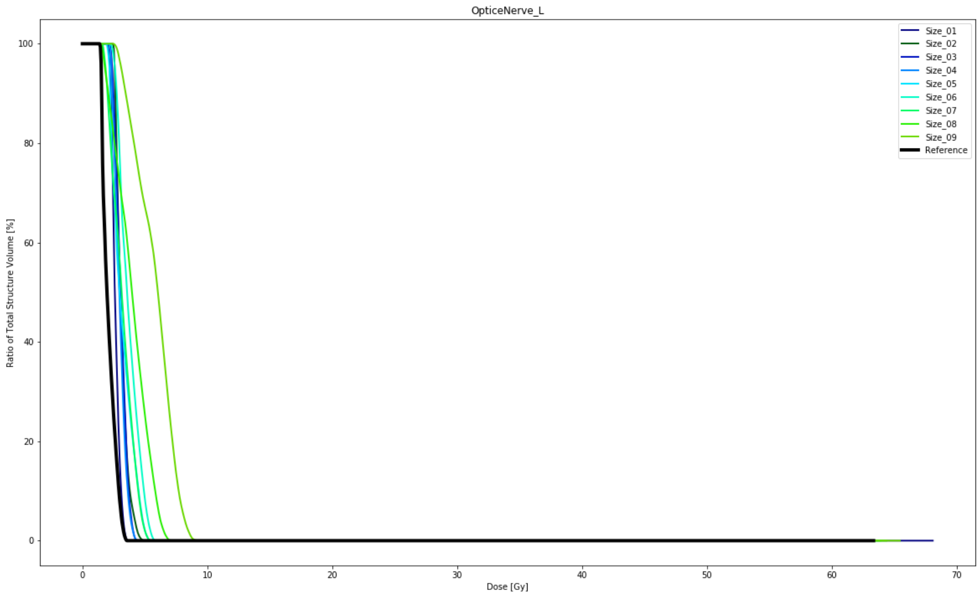

Figure 150: DVH curves of the left optic nerve structure of the reference plan and the 9 plans including different size reference target including the outlier A2 the specific location as displayed in Figure 111.

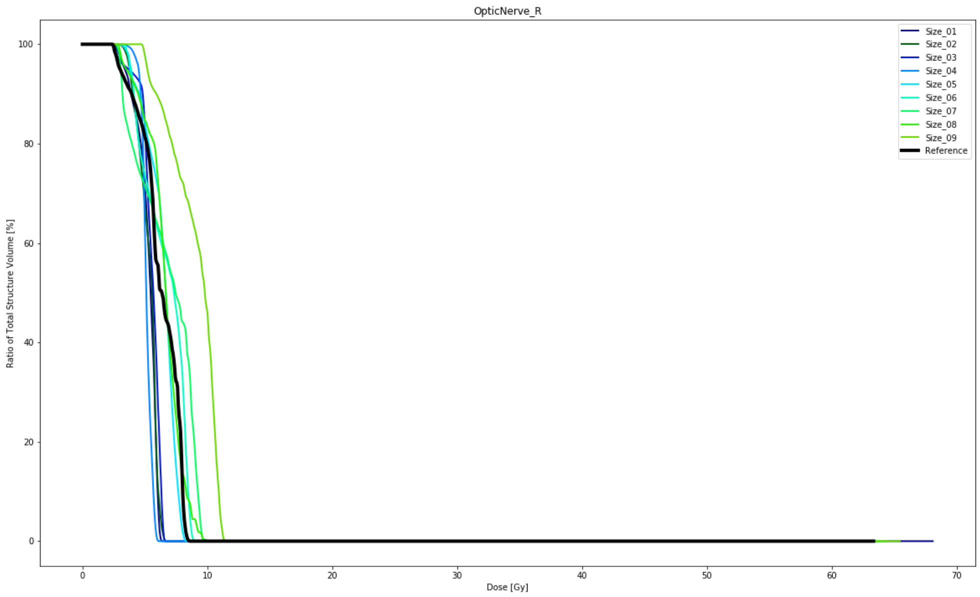

Figure 151: DVH curves of the right optic nerve structure of the reference plan and the 9 plans including different size reference target including the outlier A2 the specific location as displayed in Figure 111.

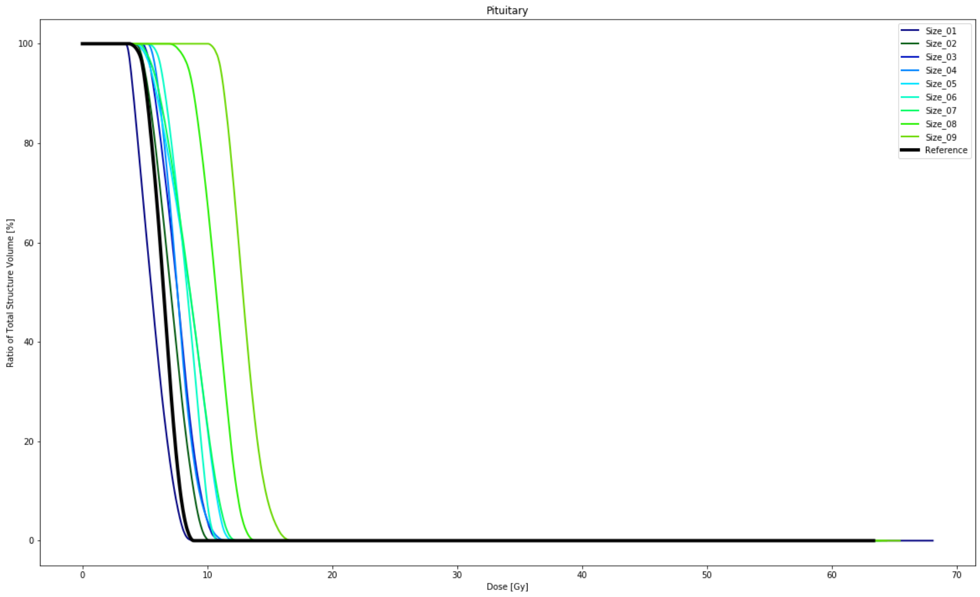

Figure 152: DVH curves of the pituitary gland structure of the reference plan and the 9 plans including different size reference target including the outlier A2 the specific location as displayed in Figure 111.

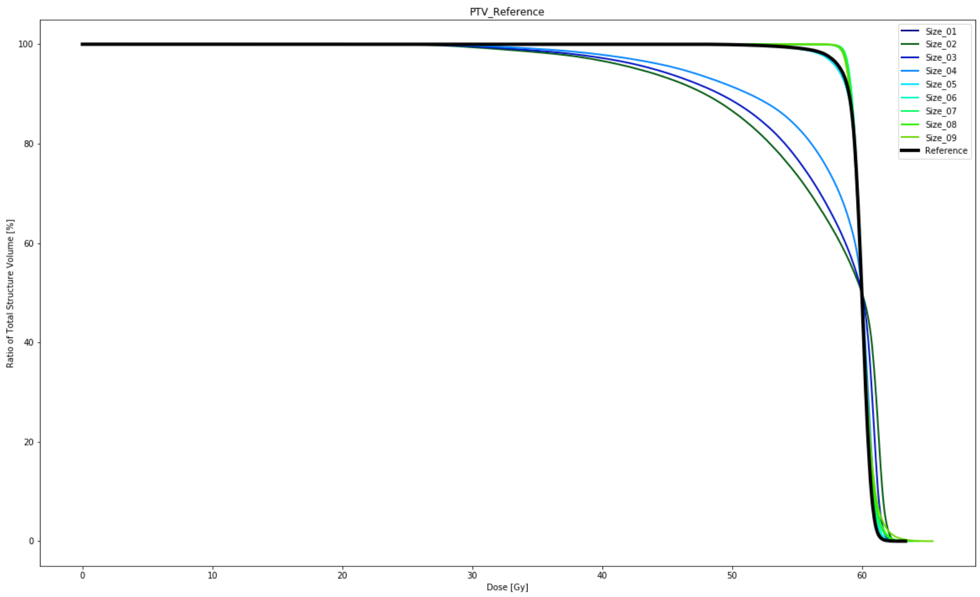

Figure 153: DVH curves of the reference PTV structure of the reference plan and the 9 plans including different size reference target including the outlier A2 the specific location as displayed in Figure 111. It has to be noted that the plans are defined on smaller and larger PTVs then the reference PTV where the DVH curves are shown in this graph.

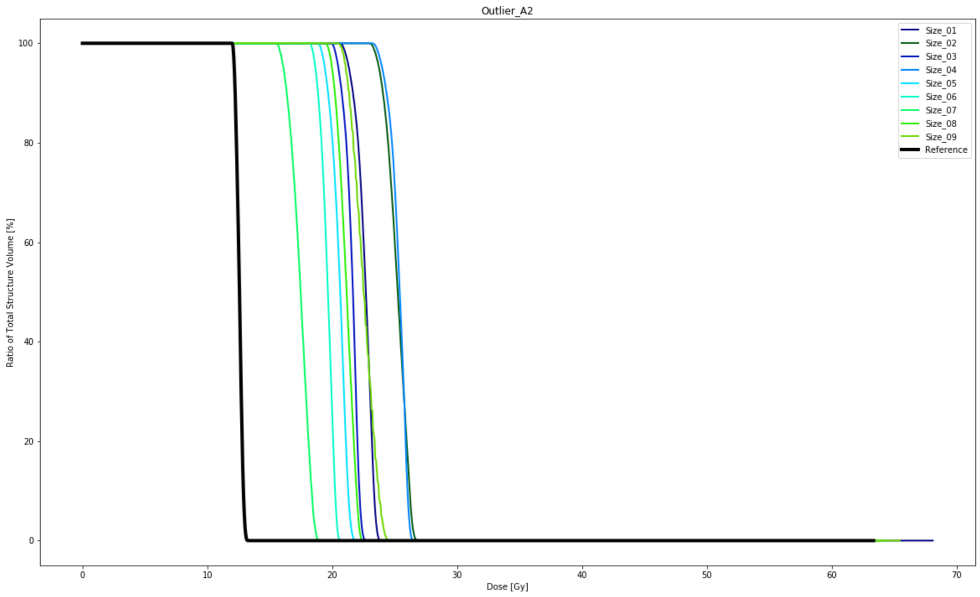

Figure 154: DVH curves of the Outlier volume A2 structure of the reference plan and the 9 plans including different size reference target including the outlier A2 the specific location as displayed in Figure 111.

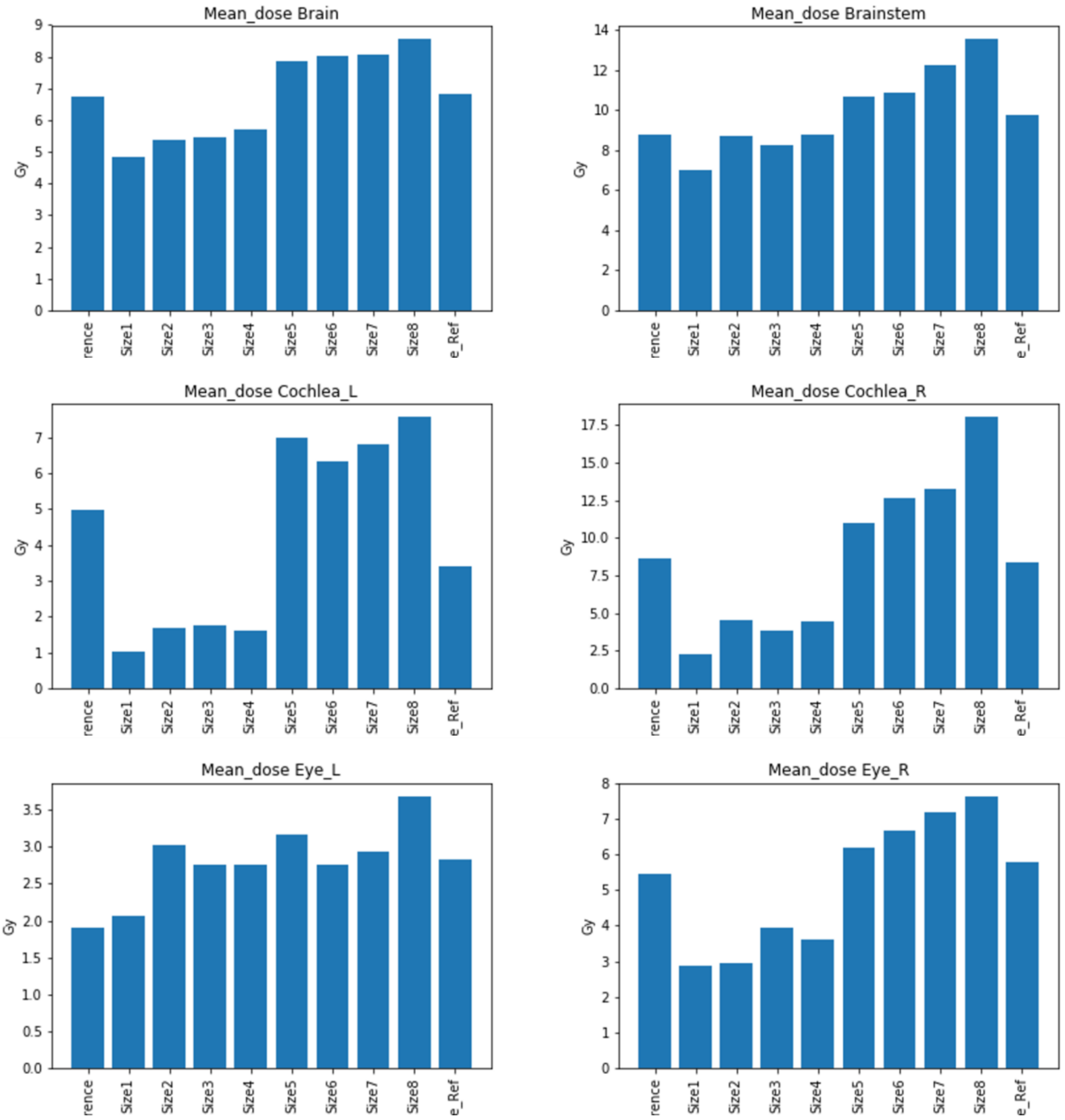

Figure 155: Bar plots of the mean dose of the brain, brainstem, cochlea and eyes, for the reference plan and the 9 plans including different size reference target including the outlier A2 the specific location as displayed in Figure 111.

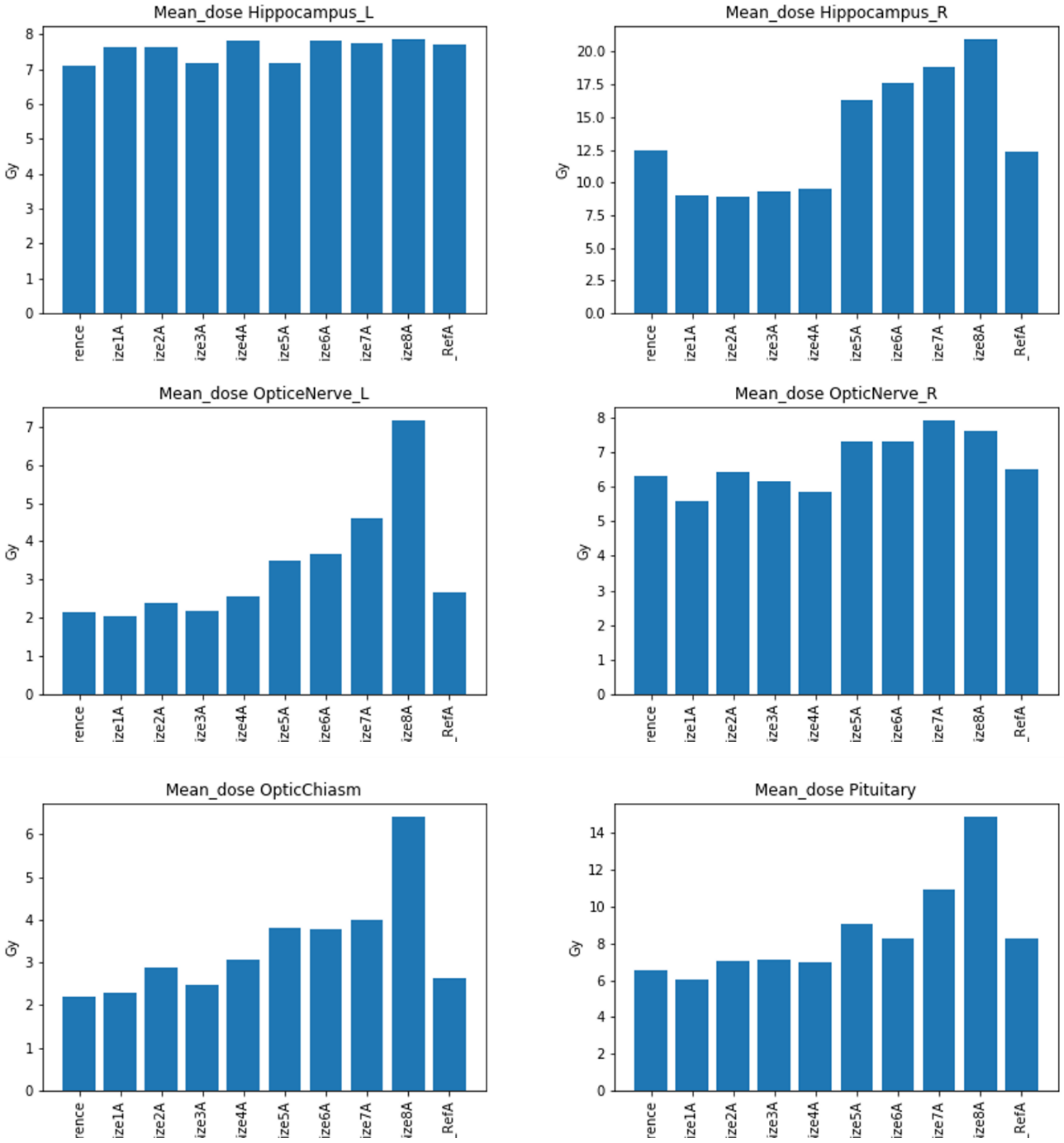

Figure 156: Bar plots of the mean dose of the hippocampi, optic nerves, optic chiasm and pituitary gland, for the reference plan and the 9 plans including different size reference target including the outlier A1 the specific location as displayed in Figure 111.

Dominant Beam Direction

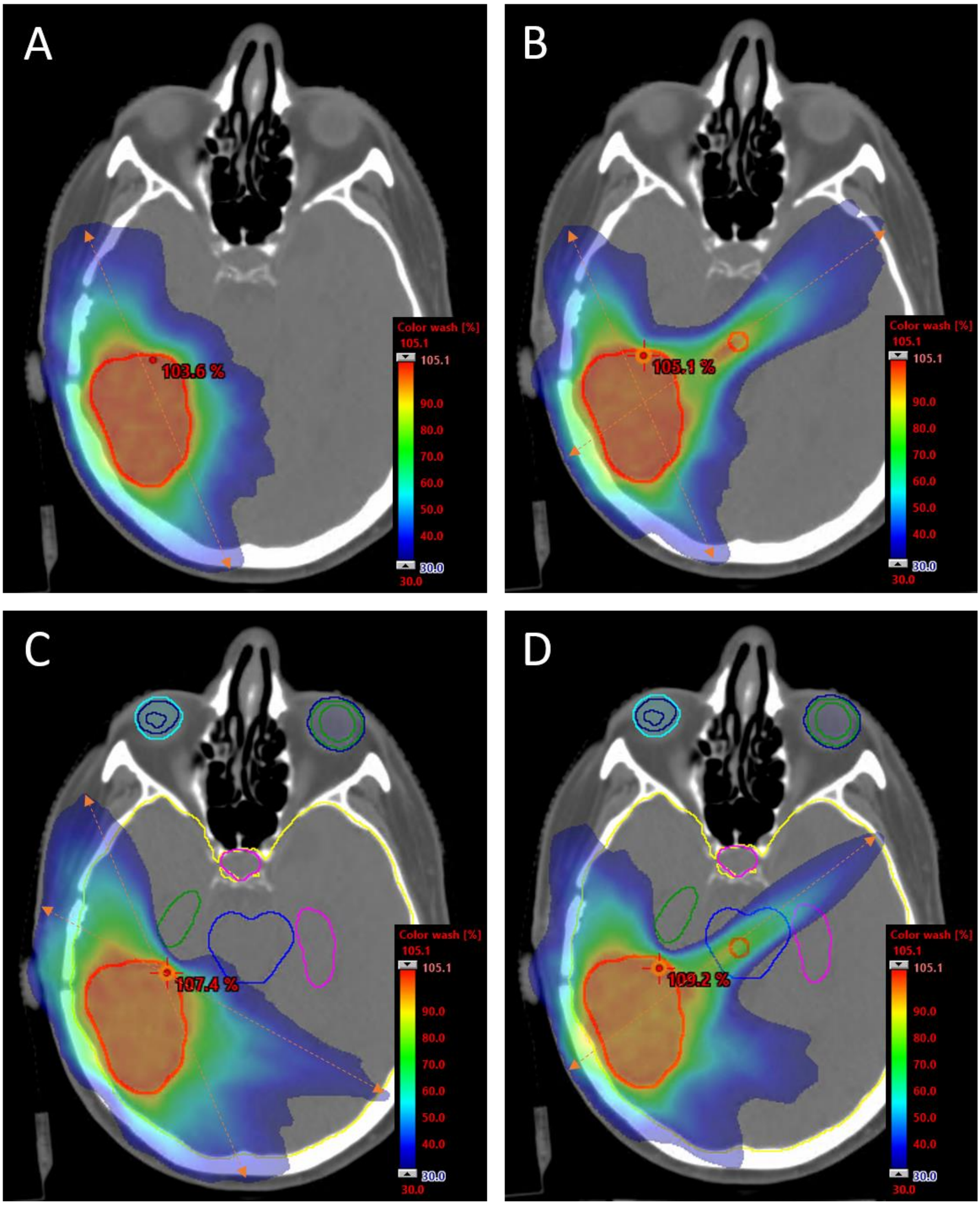

Figure 157: Planning results of experiment 1. In panel A the dose distribution of the reference target without taking OARs into account is shown. The orange arrow shows the widest dose and therefor represents the dominant beam direction. In panel B the planning result of outlier at location 10 is depicted. We can see an additional dominant beam direction forming perpendicular to the initial dominant beam direction. In panel C and D the OARs and their constraints are taken into account during the optimization and dose calculation.
