# Supplementary material for: Impact of random outliers in auto-segmented targets on radiotherapy treatment plans for glioblastoma
Source: Radiat Oncol. 2022 Oct 22;17:170. doi: 10.1186/s13014-022-02137-9 (PMC9587574; doi:10.1186/s13014-022-02137-9)
Supplement: Supplementary file 2 — Additional file 2. Results deep learning segmented outliers. [file 13014_2022_2137_MOESM2_ESM.pdf]

Additional File B:

Results Deep Learning Segmented Outliers

Outlier distribution for GTV and Edema structures per DL model.

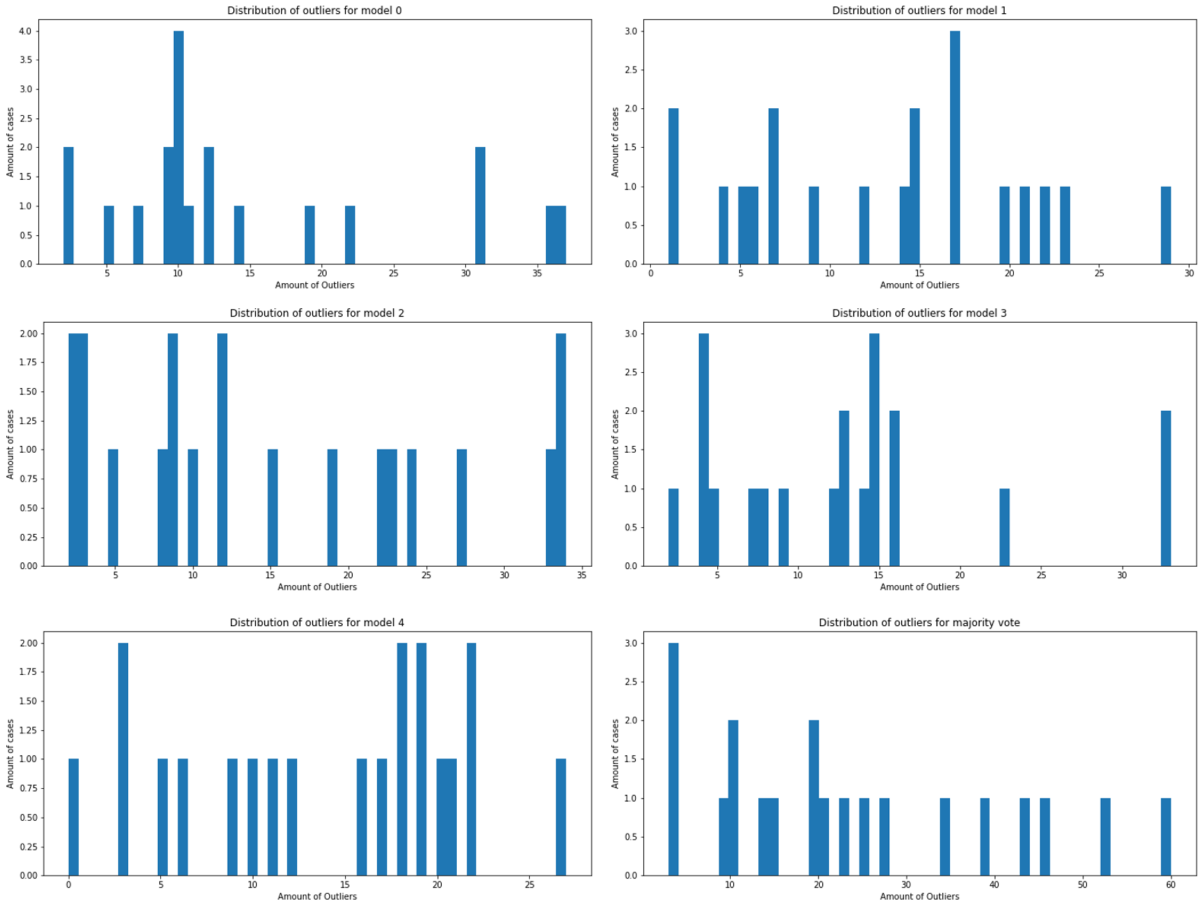

Figure 1: Distribution of the amount of outliers (both GTV and Edema) per case. All the different trained DL models (0-4) are shown. The votes for the majority vote ensembling are shown on the lower right.

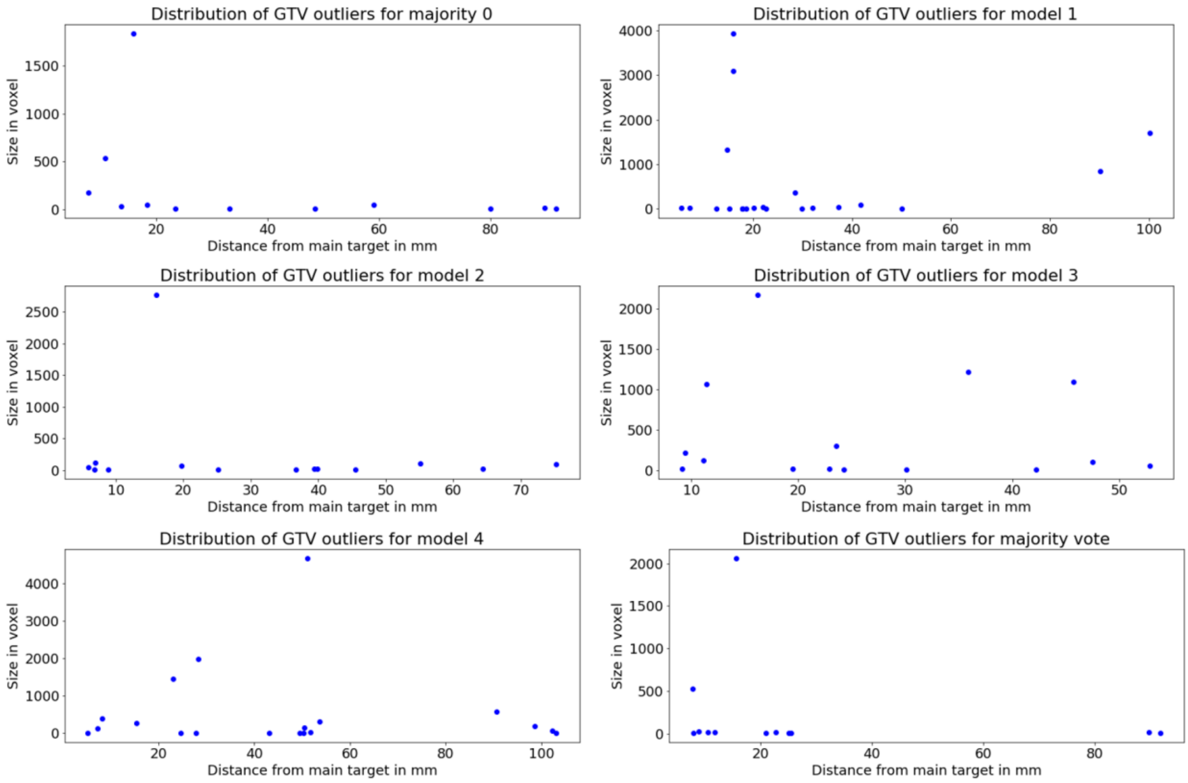

Figure 2: The distribution of the size and distance from the main target of all the outliers in the GTV structure for all separately trained DL models (0-4) and the majority vote ensembling at the bottom right.

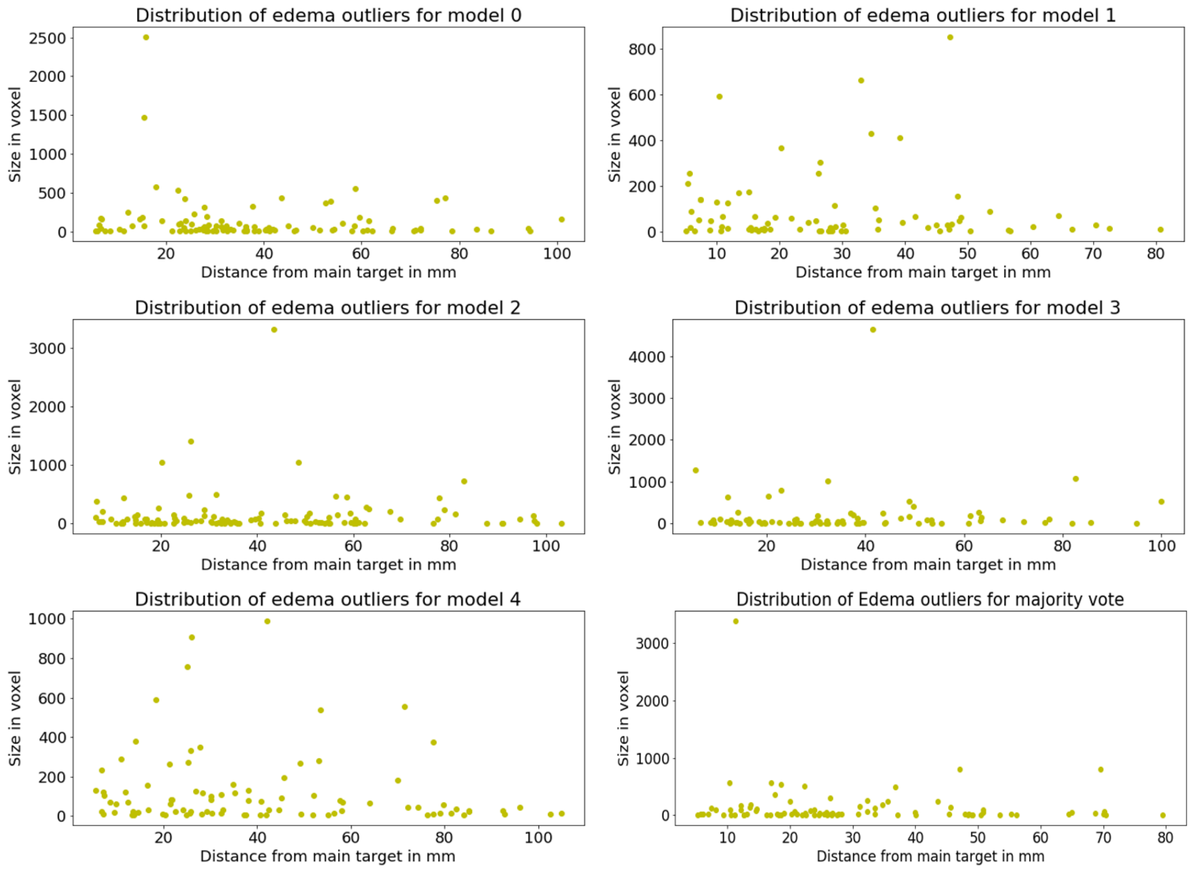

Figure 3: The distribution of the size and distance from the main target of all the outliers in the Edema structure for all separately trained DL models (0-4) and the majority vote ensembling at the bottom right.

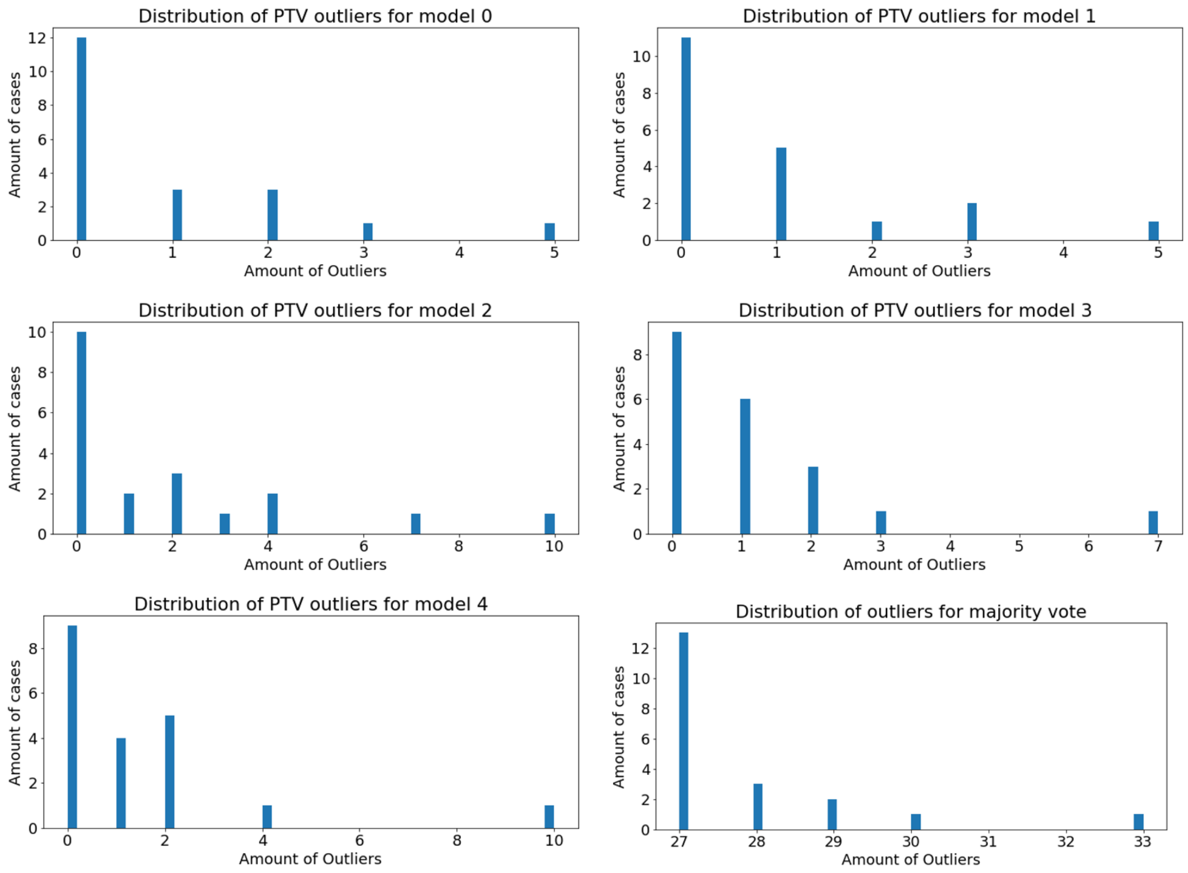

Figure 4: Distribution of the amount of outliers per case on the constructed PTV volume. All the different trained DL models (0-4) are shown. The votes for the majority vote ensembling are shown on the lower right.

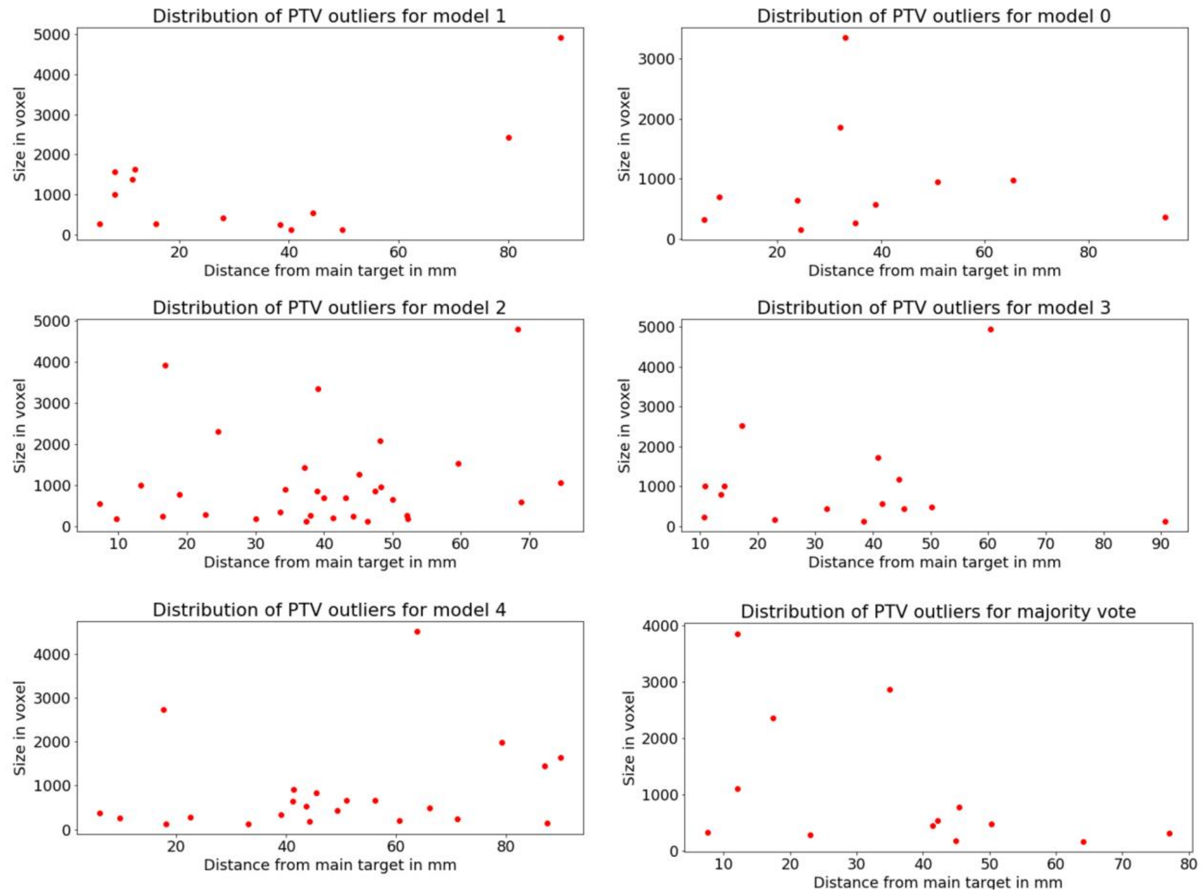

Figure 5: The distribution of the size and distance from the main target of all the outliers in the PTV structure for all separately trained DL models (0-4) and the majority vote ensembling at the bottom right.

Dosimetric results 5 selected cases.

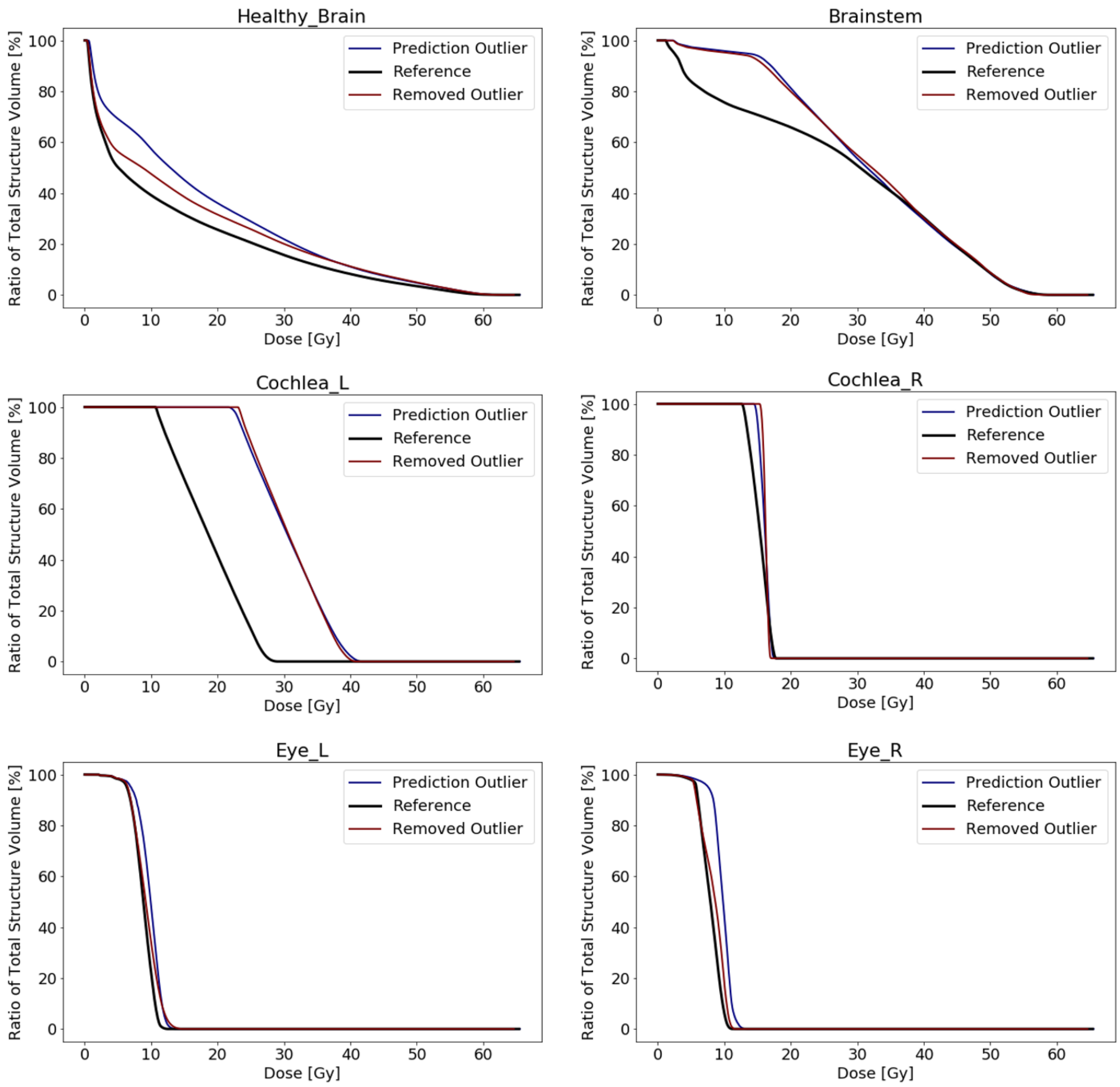

Figure 6: DVH curves of of the reference plan (black), the plan based on the DL predicted PTV containing the outlier (blue) and the predicted PTV with the outlier removed (red) for 6 different OARs for case 01.

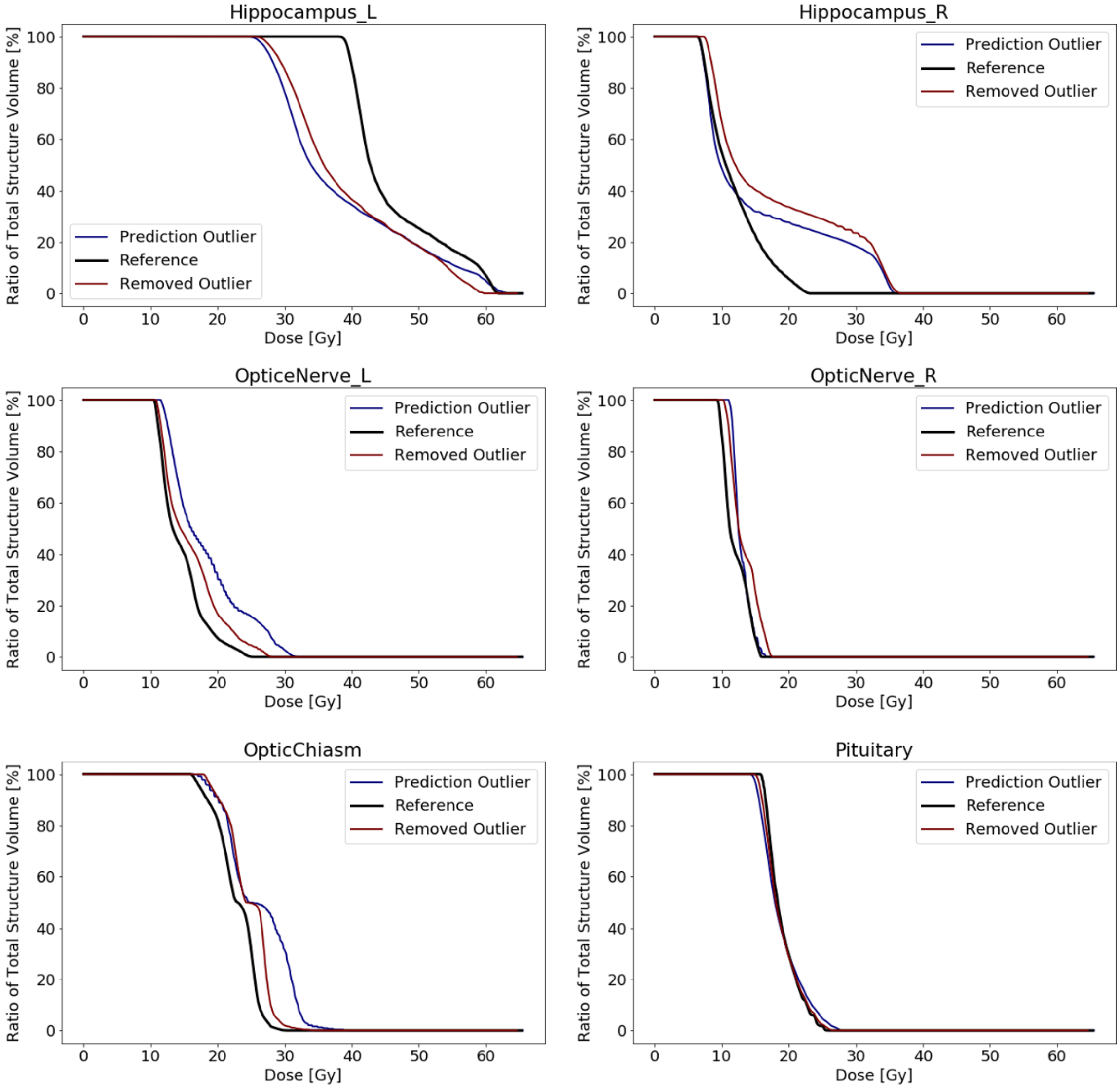

Figure 7: DVH curves of of the reference plan (black), the plan based on the DL predicted PTV containing the outlier (blue) and the predicted PTV with the outlier removed (red) for 6 different OARs for case 01.

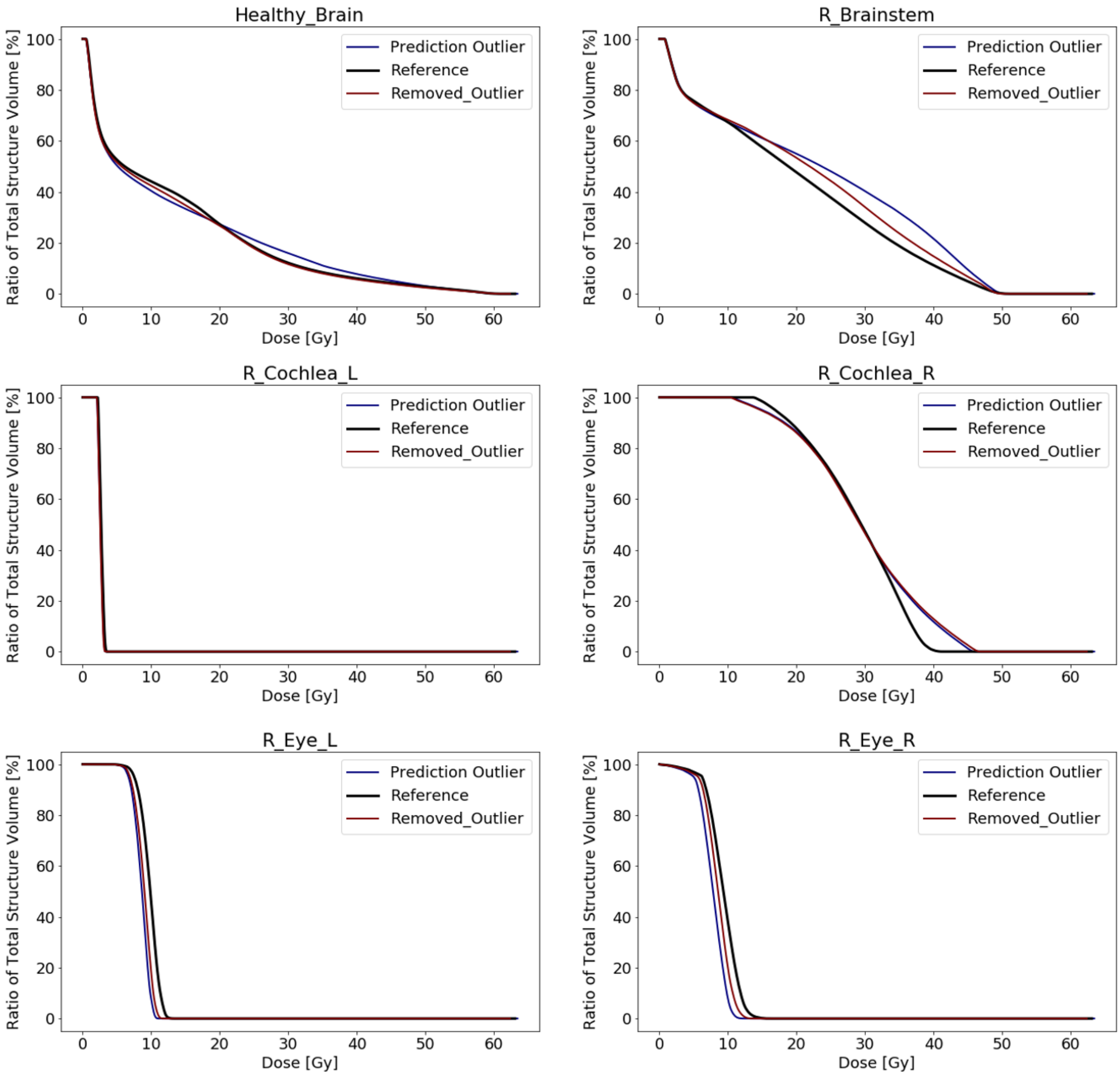

Figure 8: DVH curves of of the reference plan (black), the plan based on the DL predicted PTV containing the outlier (blue) and the predicted PTV with the outlier removed (red) for 6 different OARs for case 02.

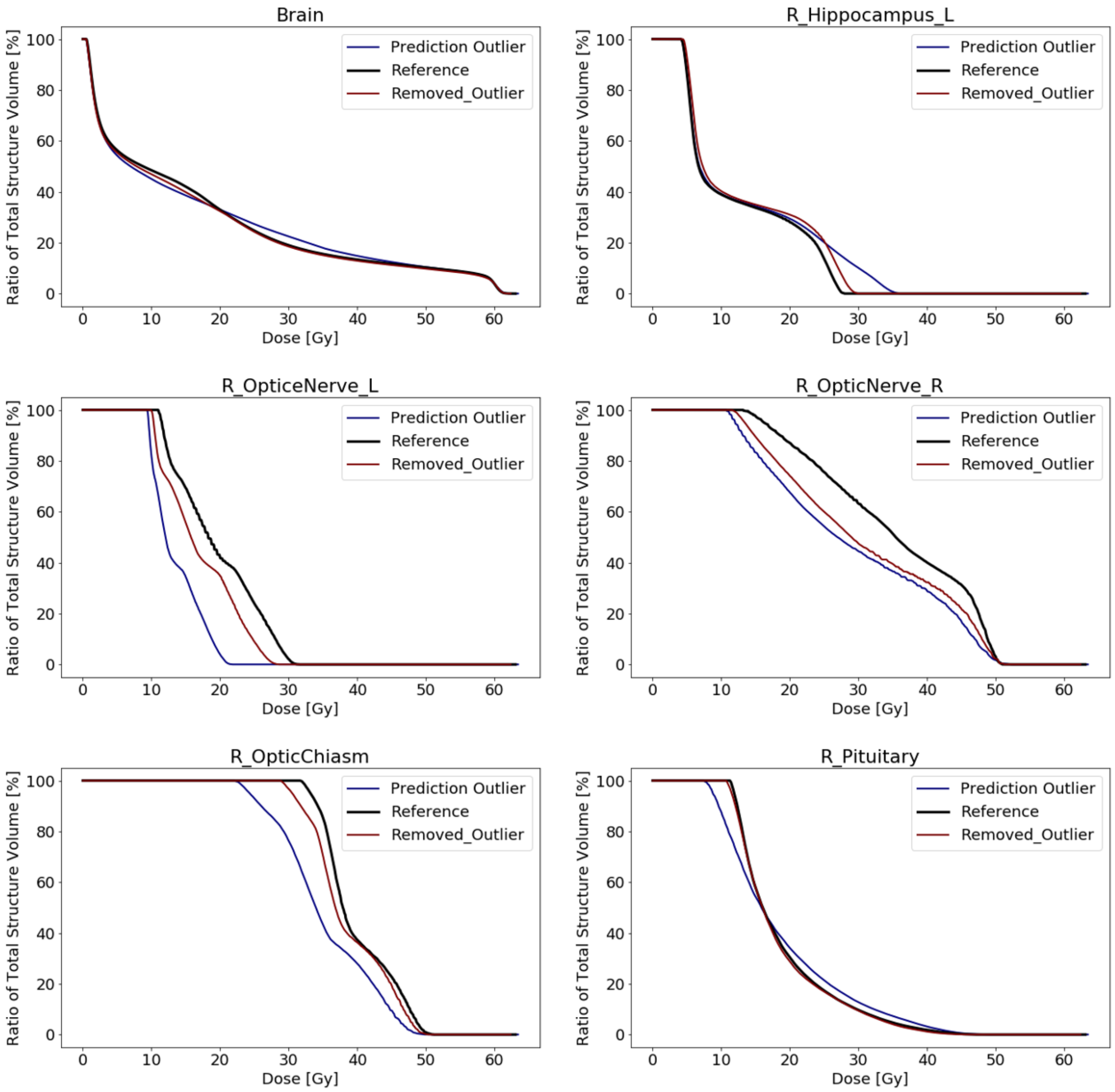

Figure 9: DVH curves of of the reference plan (black), the plan based on the DL predicted PTV containing the outlier (blue) and the predicted PTV with the outlier removed (red) for 6 different OARs for case 02.

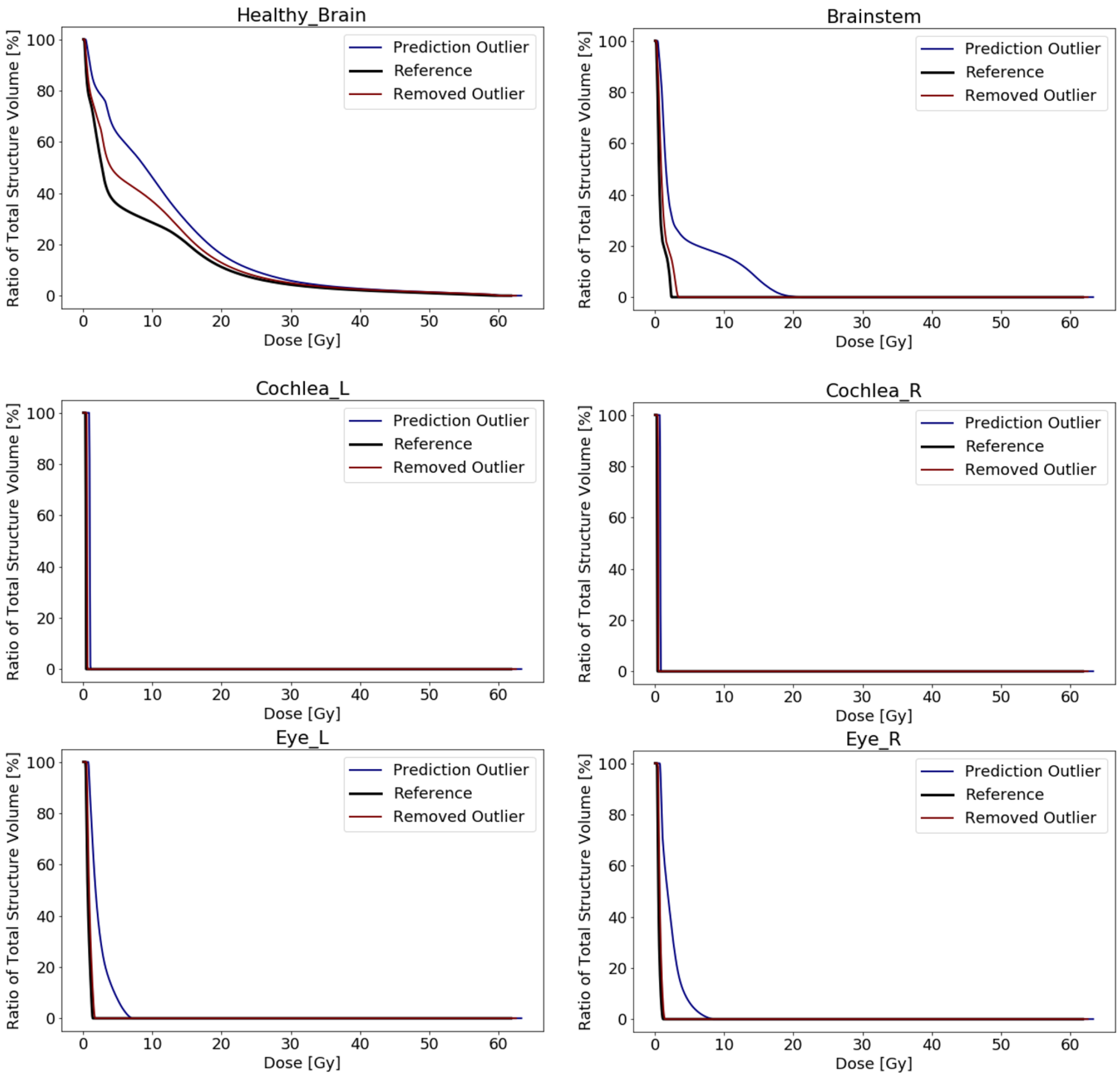

Figure 10: DVH curves of of the reference plan (black), the plan based on the DL predicted PTV containing the outlier (blue) and the predicted PTV with the outlier removed (red) for 6 different OARs for case 03.

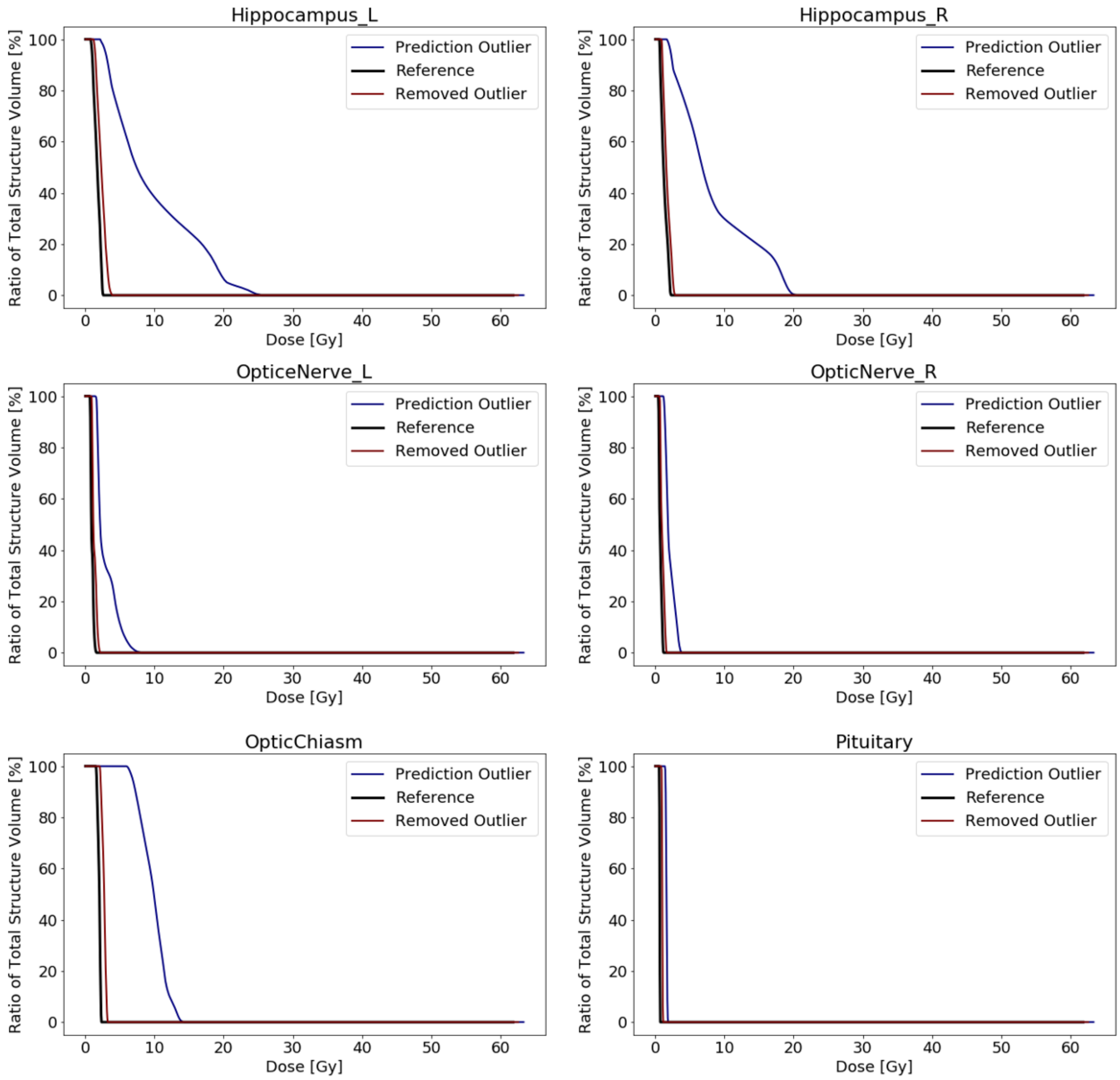

Figure 11: DVH curves of of the reference plan (black), the plan based on the DL predicted PTV containing the outlier (blue) and the predicted PTV with the outlier removed (red) for 6 different OARs for case 03.

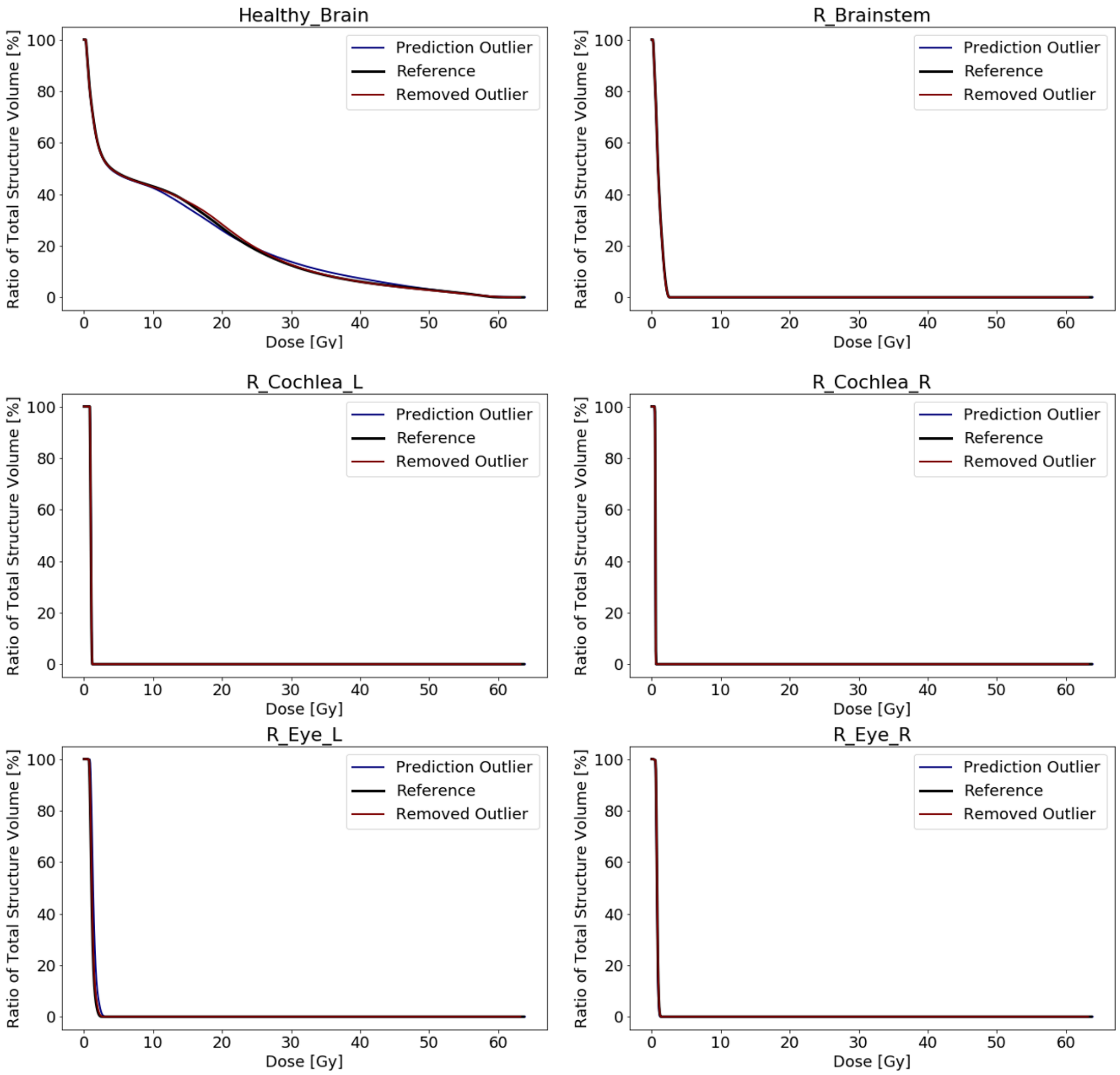

Figure 12: DVH curves of of the reference plan (black), the plan based on the DL predicted PTV containing the outlier (blue) and the predicted PTV with the outlier removed (red) for 6 different OARs for case 04.

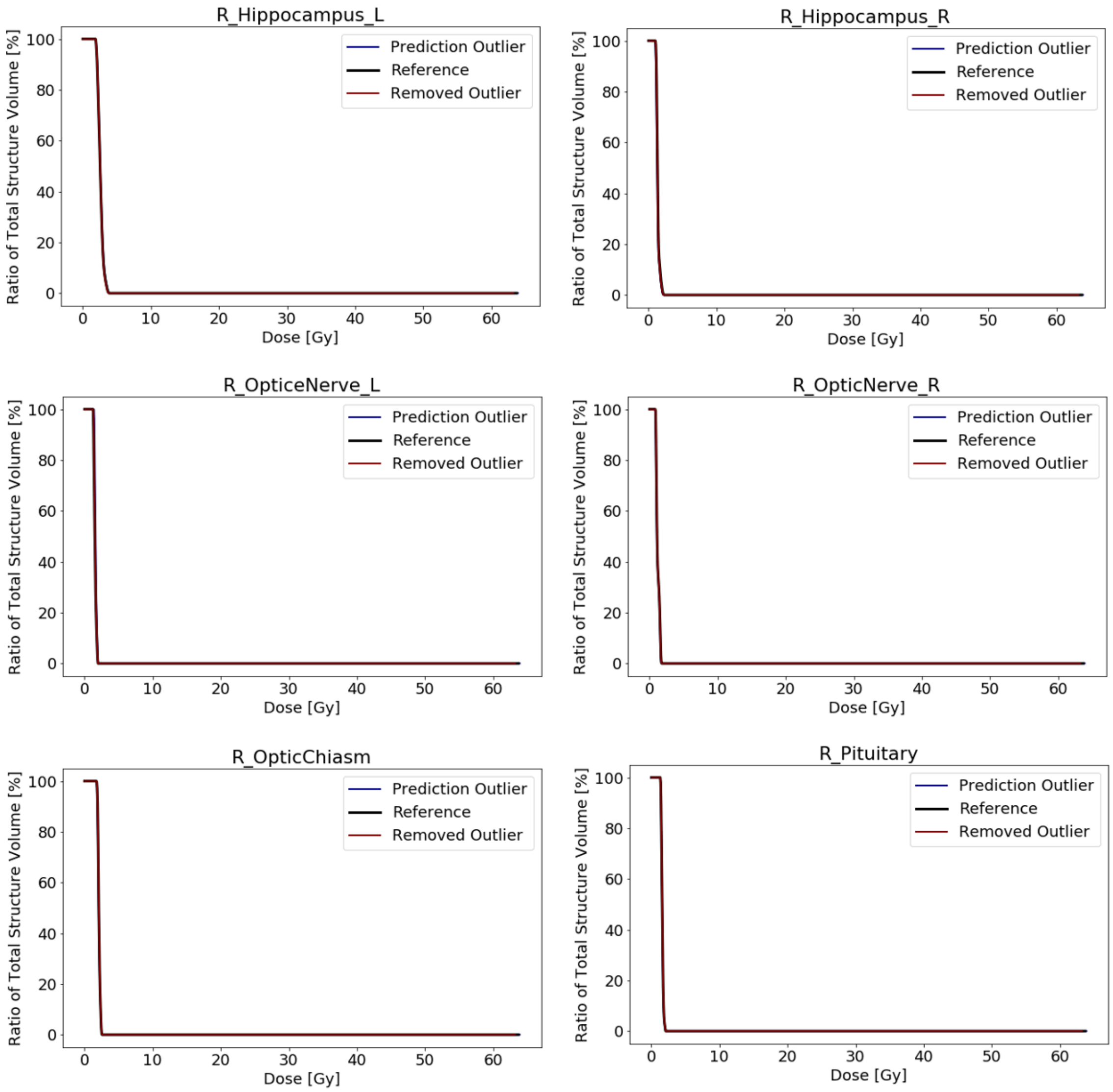

Figure 13: DVH curves of of the reference plan (black), the plan based on the DL predicted PTV containing the outlier (blue) and the predicted PTV with the outlier removed (red) for 6 different OARs for case 04.

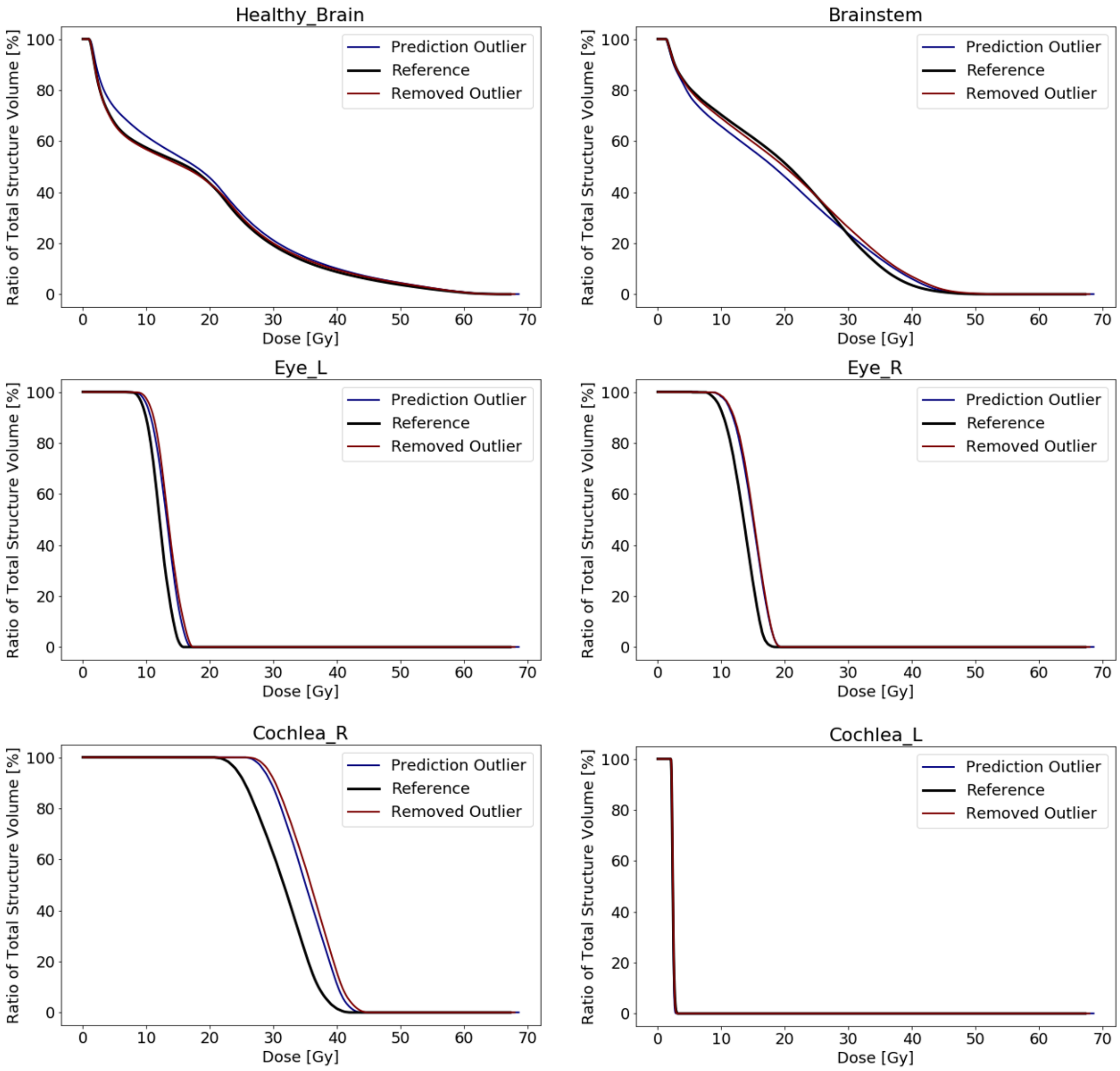

Figure 14: DVH curves of of the reference plan (black), the plan based on the DL predicted PTV containing the outlier (blue) and the predicted PTV with the outlier removed (red) for 6 different OARs for case 05.

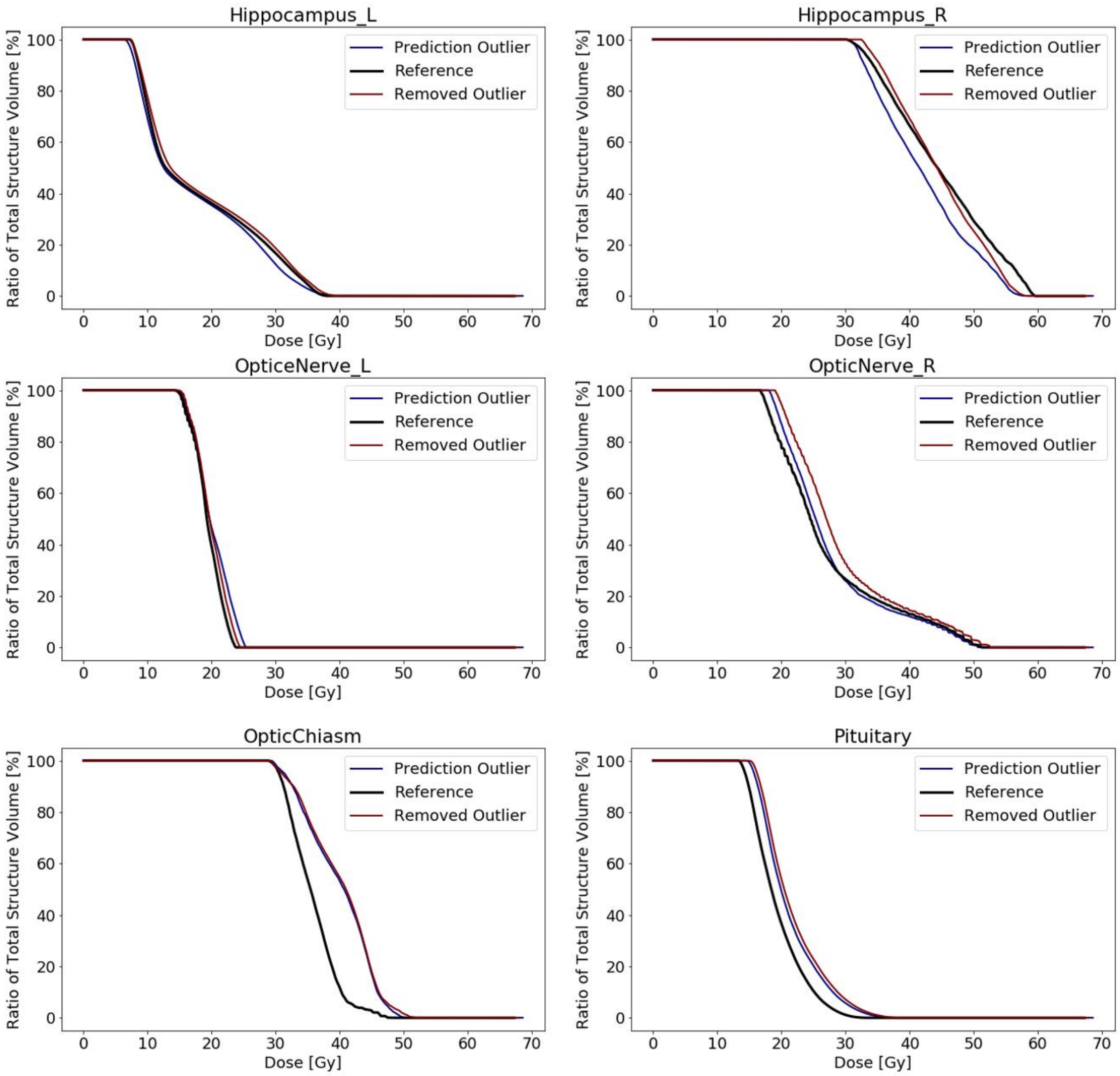

Figure 15: DVH curves of of the reference plan (black), the plan based on the DL predicted PTV containing the outlier (blue) and the predicted PTV with the outlier removed (red) for 6 different OARs for case 05.

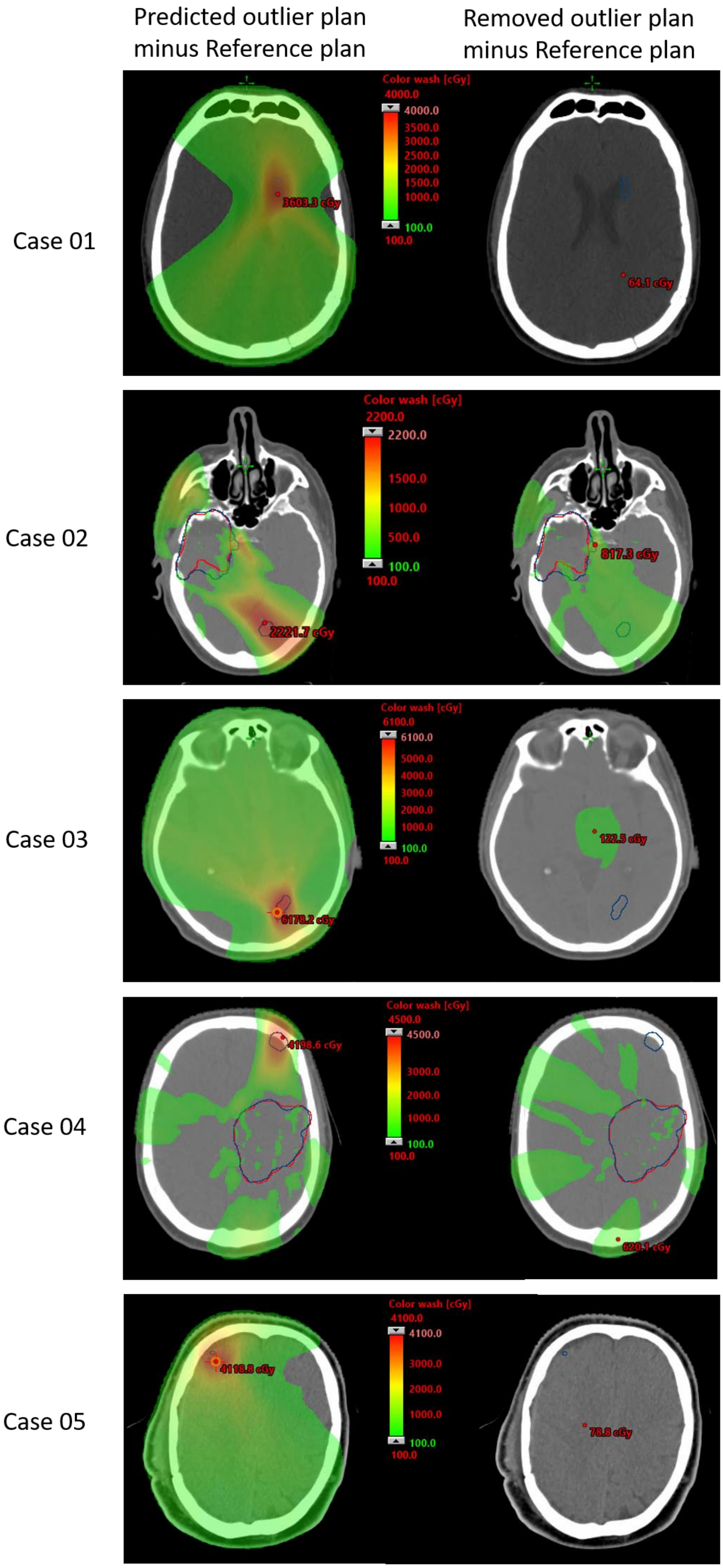
Predicted outlier plan minus Reference plan
Removed outlier plan minus Reference plan
Case 01
Color wash [cGy]
4000.0
3500.0
3000.0
2500.0
2000.0
1500.0
1000.0
100.0
3603.3 cGy
64.1 cGy
Case 02
Color wash [cGy]
2200.0
1500.0
1000.0
500.0
100.0
2221.7 cGy
817.3 cGy
Case 03
Color wash [cGy]
6100.0
5000.0
4000.0
3000.0
2000.0
1000.0
100.0
6178.2 cGy
122.5 cGy
Case 04
Color wash [cGy]
4500.0
3000.0
2000.0
1000.0
100.0
4198.6 cGy
620.1 cGy
Case 05
Color wash [cGy]
4100.0
3000.0
2000.0
1000.0
100.0
4118.8 cGy
78.8 cGy

Figure 16: Dose difference maps showing the increased dose of the plan with the outlier in the predicted PTV (left) and the plan with the outlier removed (right) with respect to the reference plan. For each case the scale of the Dose is different and is shown in centi-gray.

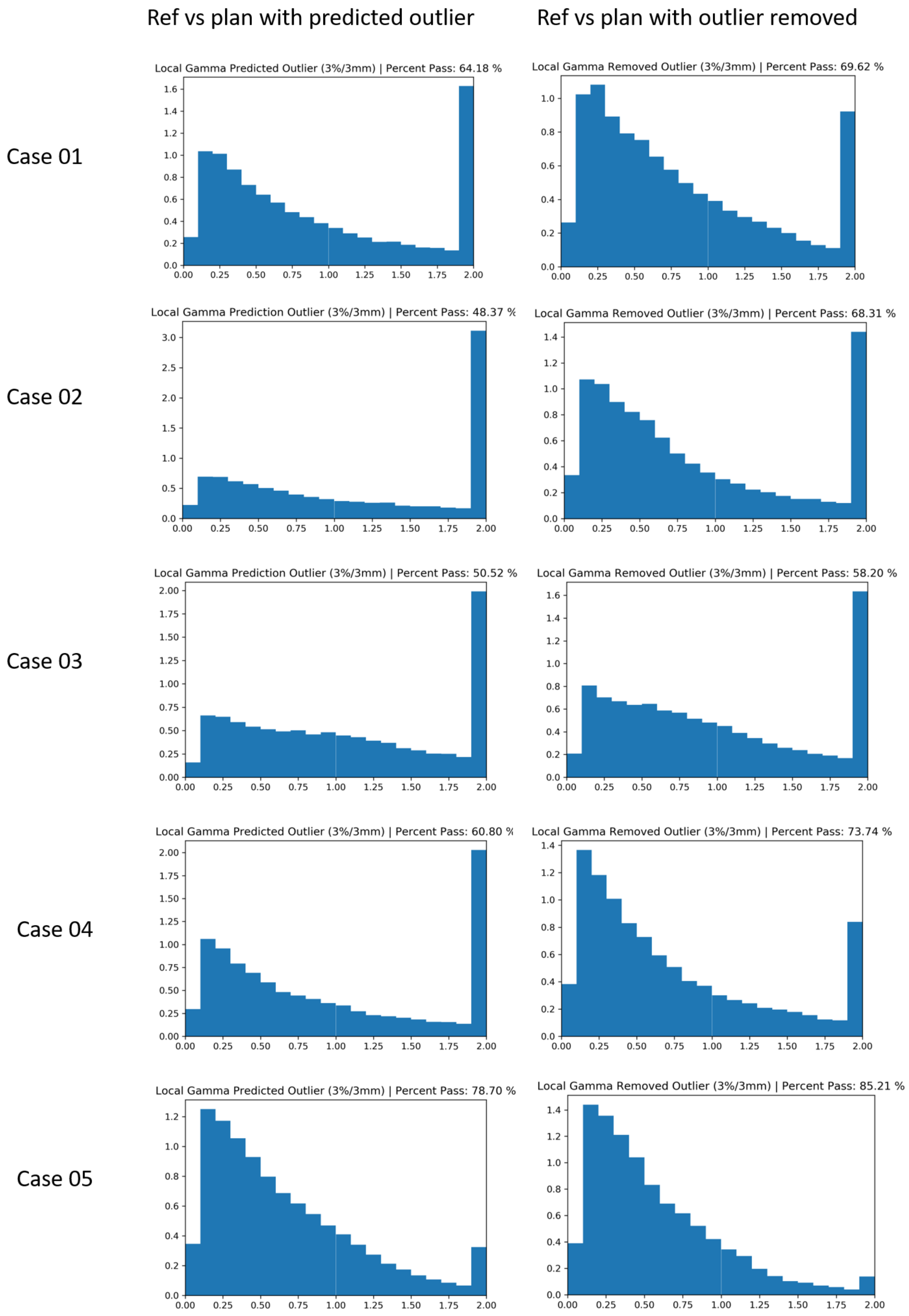

Figure 17: Results of the gamma analysis of both the plan with the outlier in the predicted PTV (left) and the plan with the

outlier removed (right) with respect to the reference plan. The criterion was 3% and 3 mm. Only doses above 20 Gy are considered in the analysis.
