# Supplementary material for: Impact of random outliers in auto-segmented targets on radiotherapy treatment plans for glioblastoma
Source: Radiat Oncol. 2022 Oct 22;17:170. doi: 10.1186/s13014-022-02137-9 (PMC9587574; doi:10.1186/s13014-022-02137-9)
Supplement: Supplementary file 3 — Additional file 3. Gifs of dosimetric results segmented outliers. [file 13014_2022_2137_MOESM3_ESM.pptx]

## Slide 1
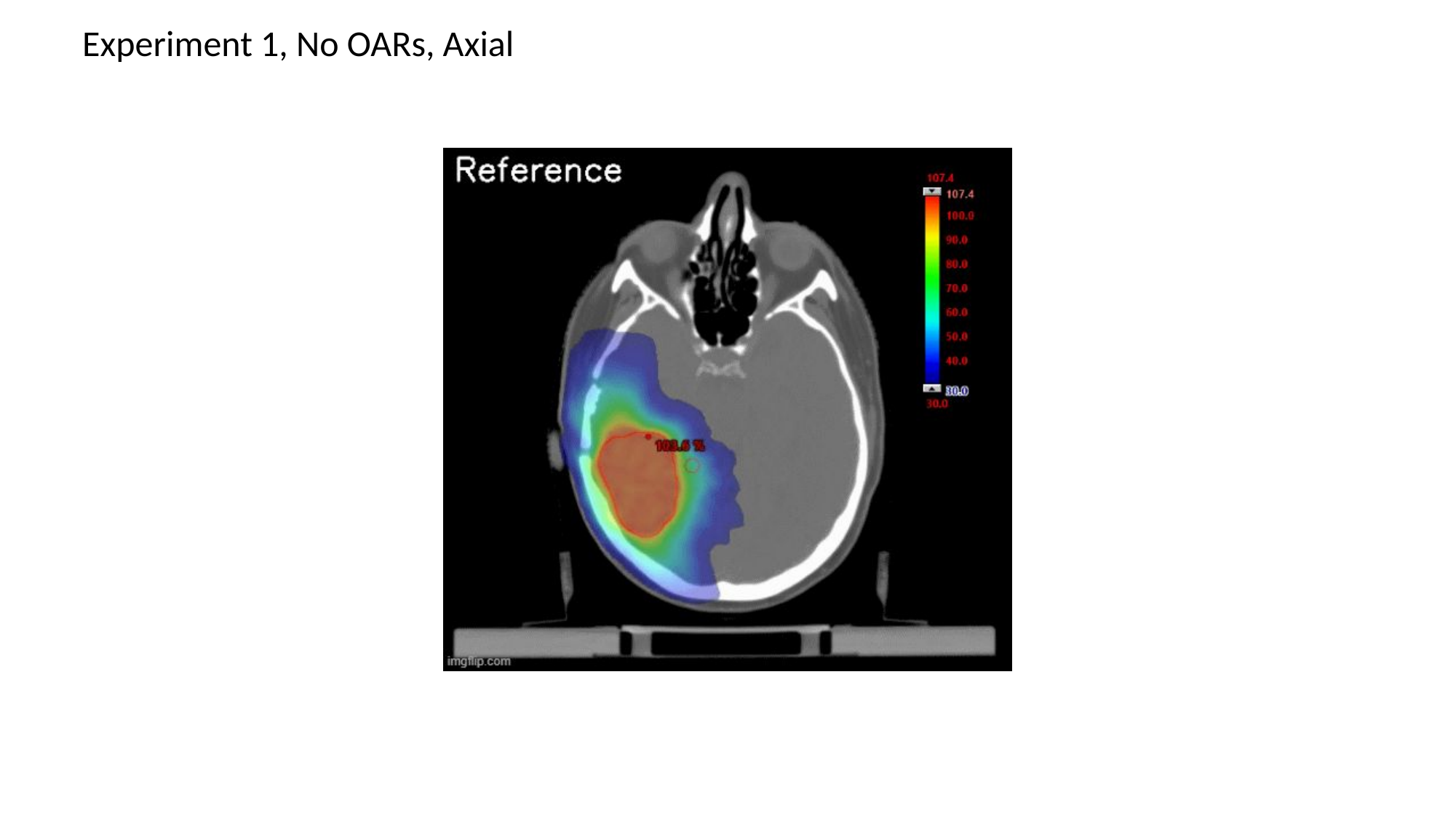

Experiment 1, No OARs, Axial

## Slide 2
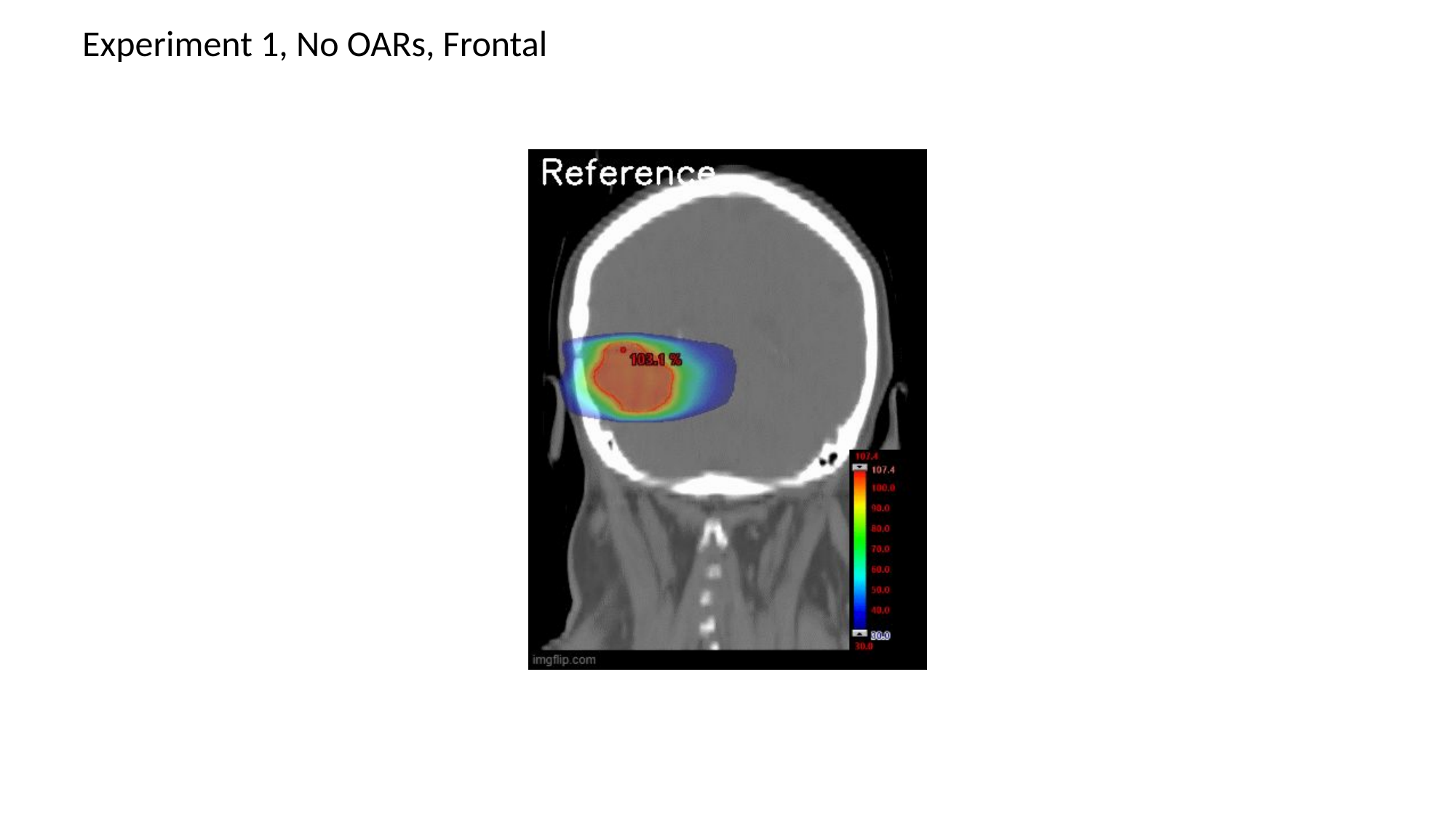

Experiment 1, No OARs, Frontal

## Slide 3
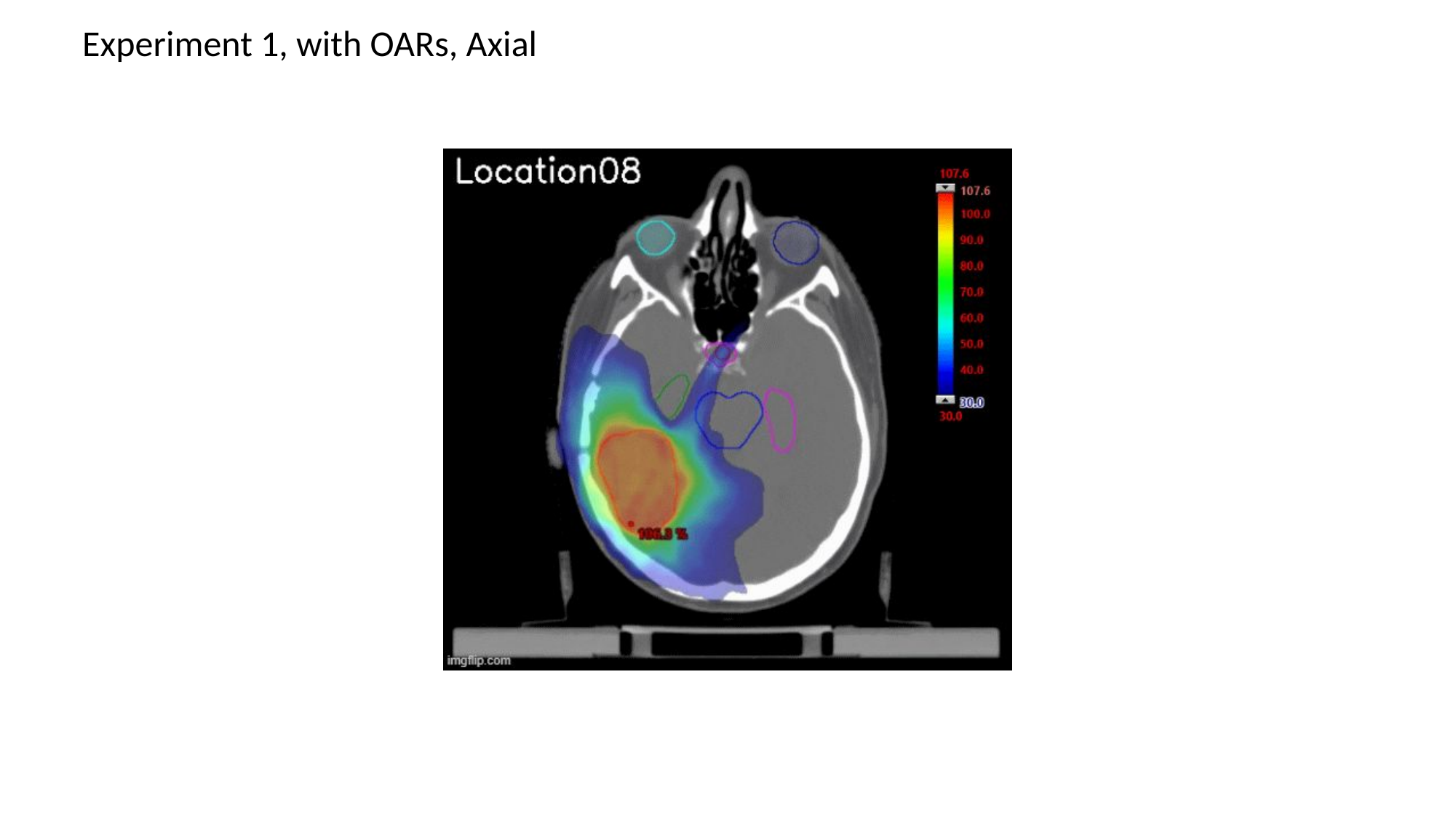

Experiment 1, with OARs, Axial

## Slide 4
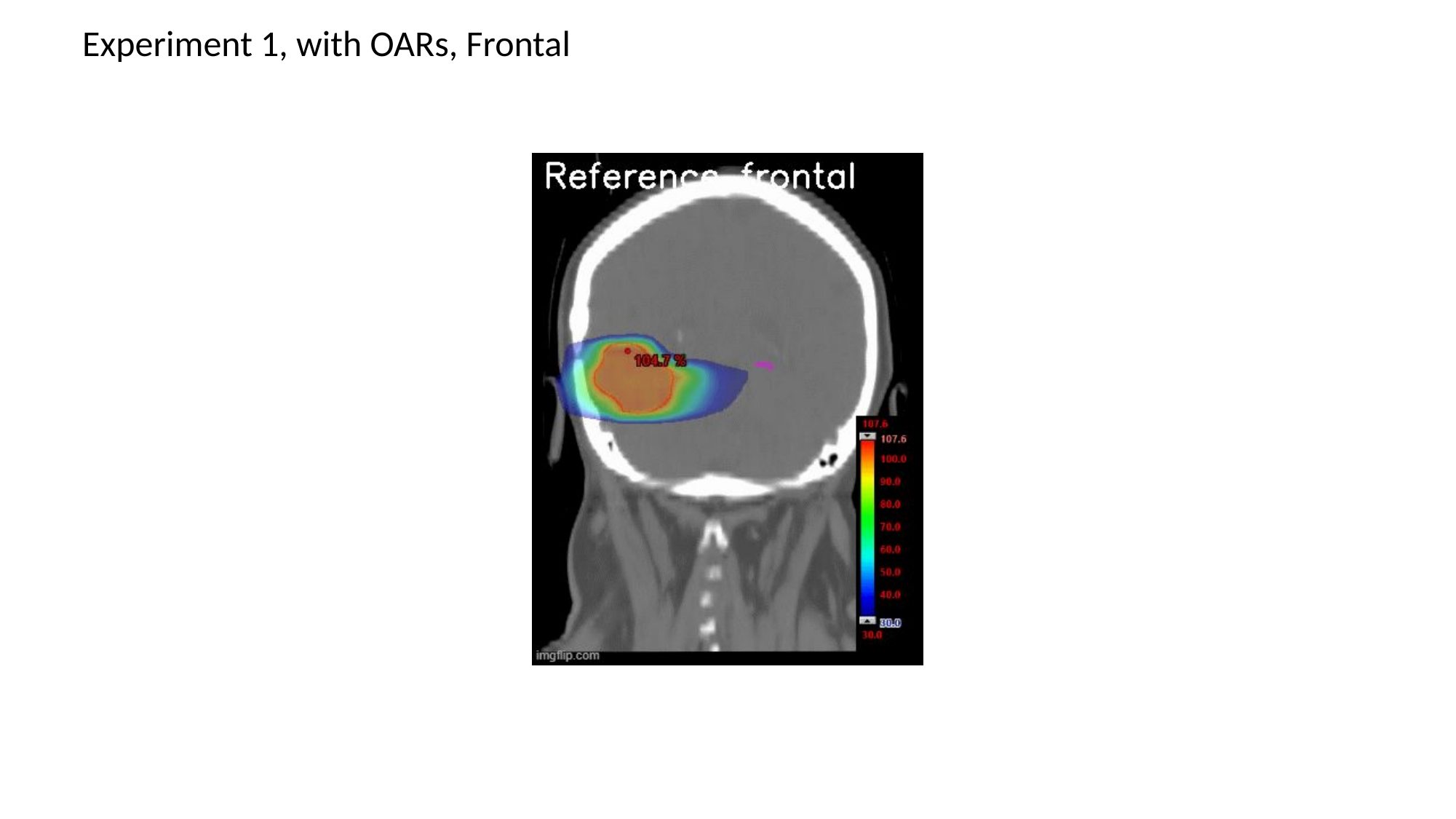

Experiment 1, with OARs, Frontal

## Slide 5
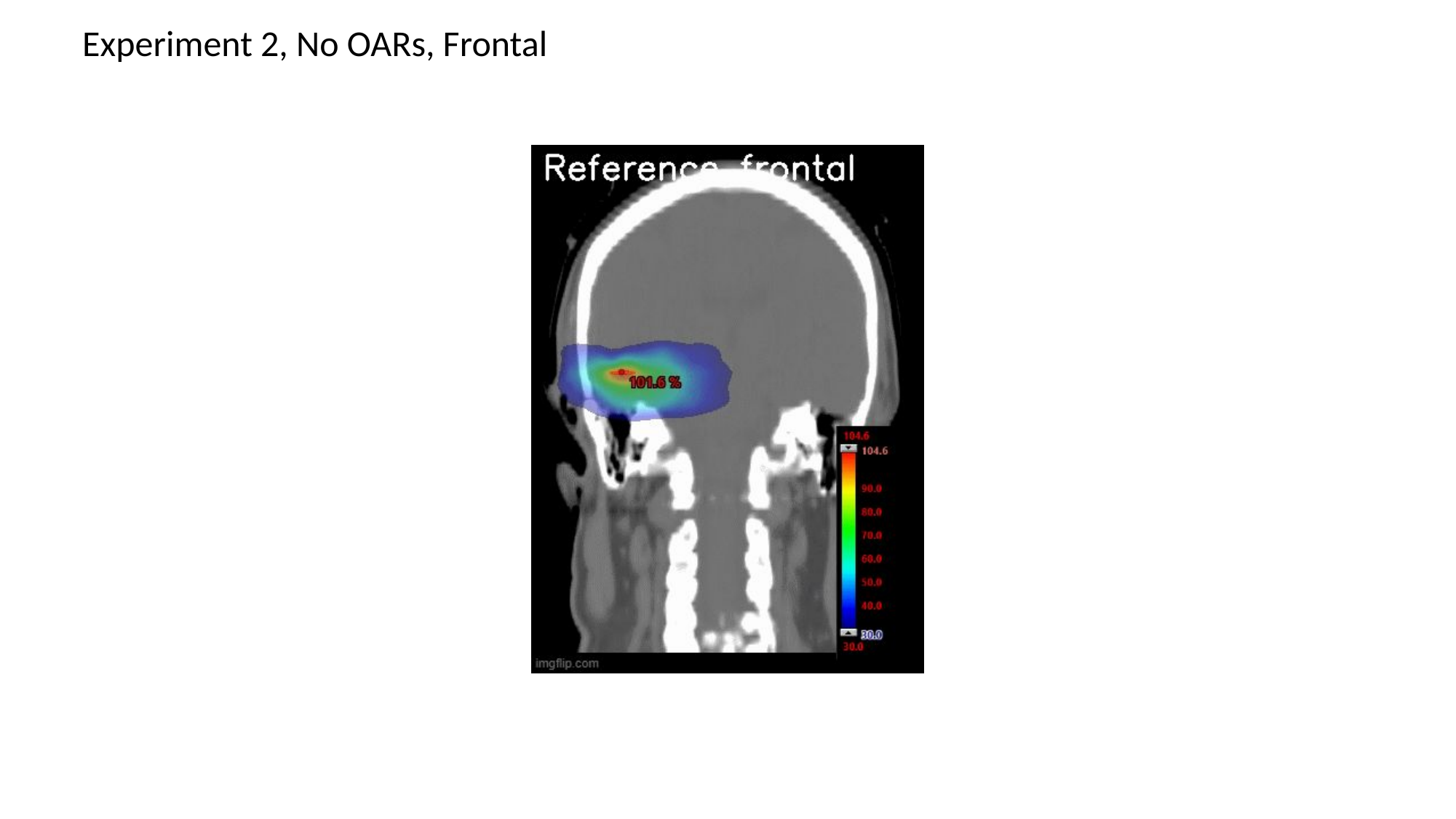

Experiment 2, No OARs, Frontal

## Slide 6
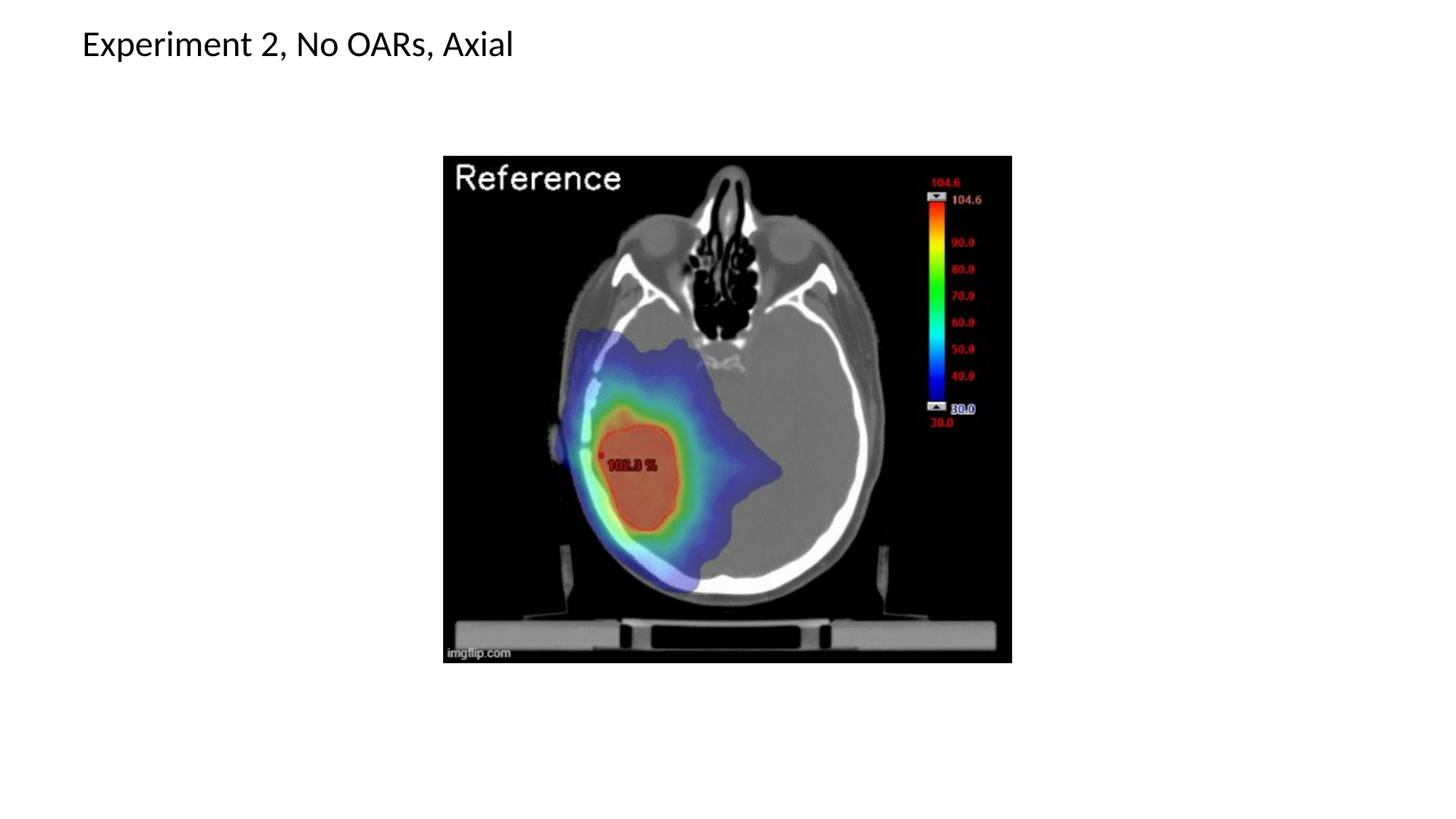

Experiment 2, No OARs, Axial

## Slide 7
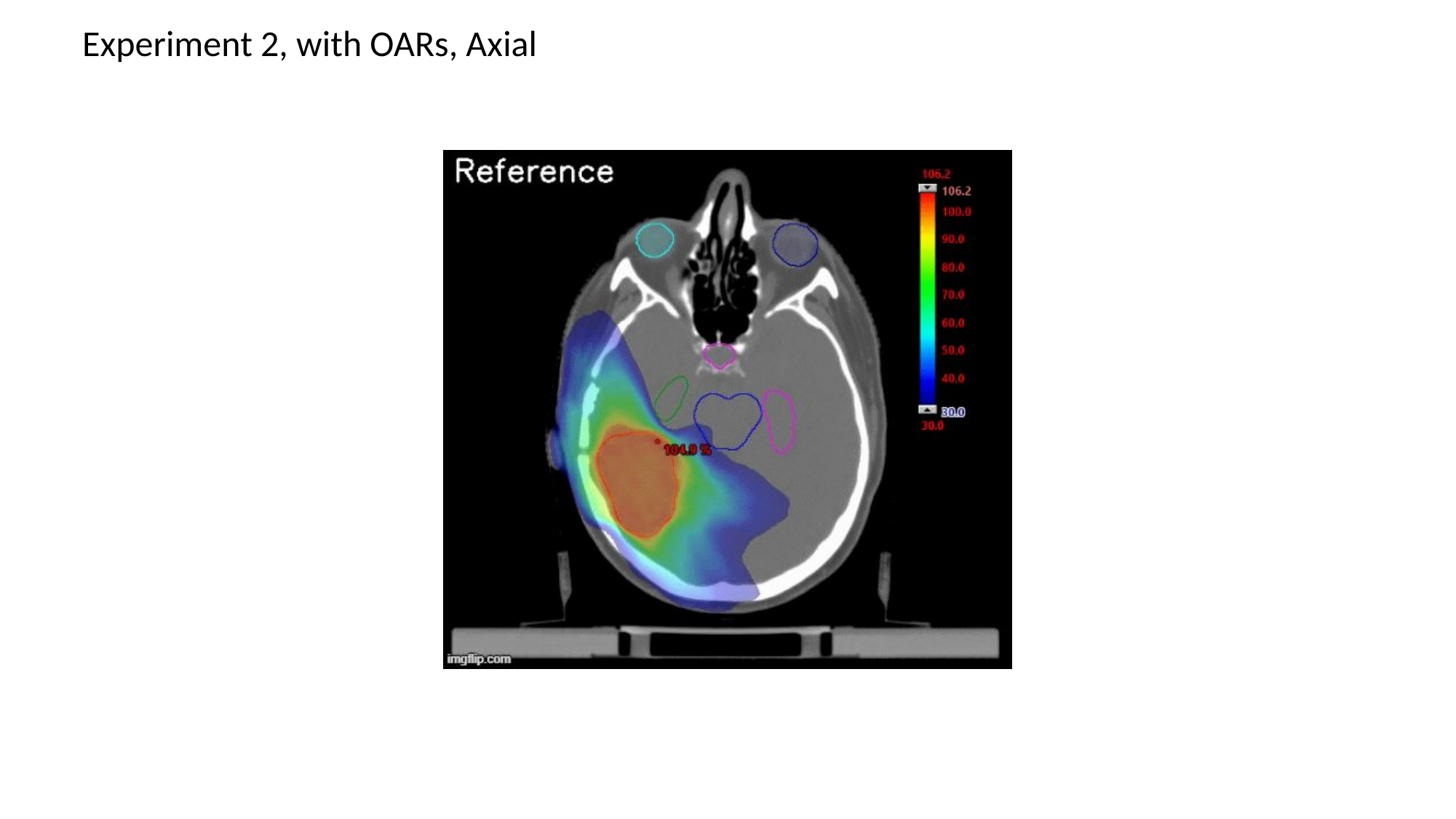

Experiment 2, with OARs, Axial

## Slide 8
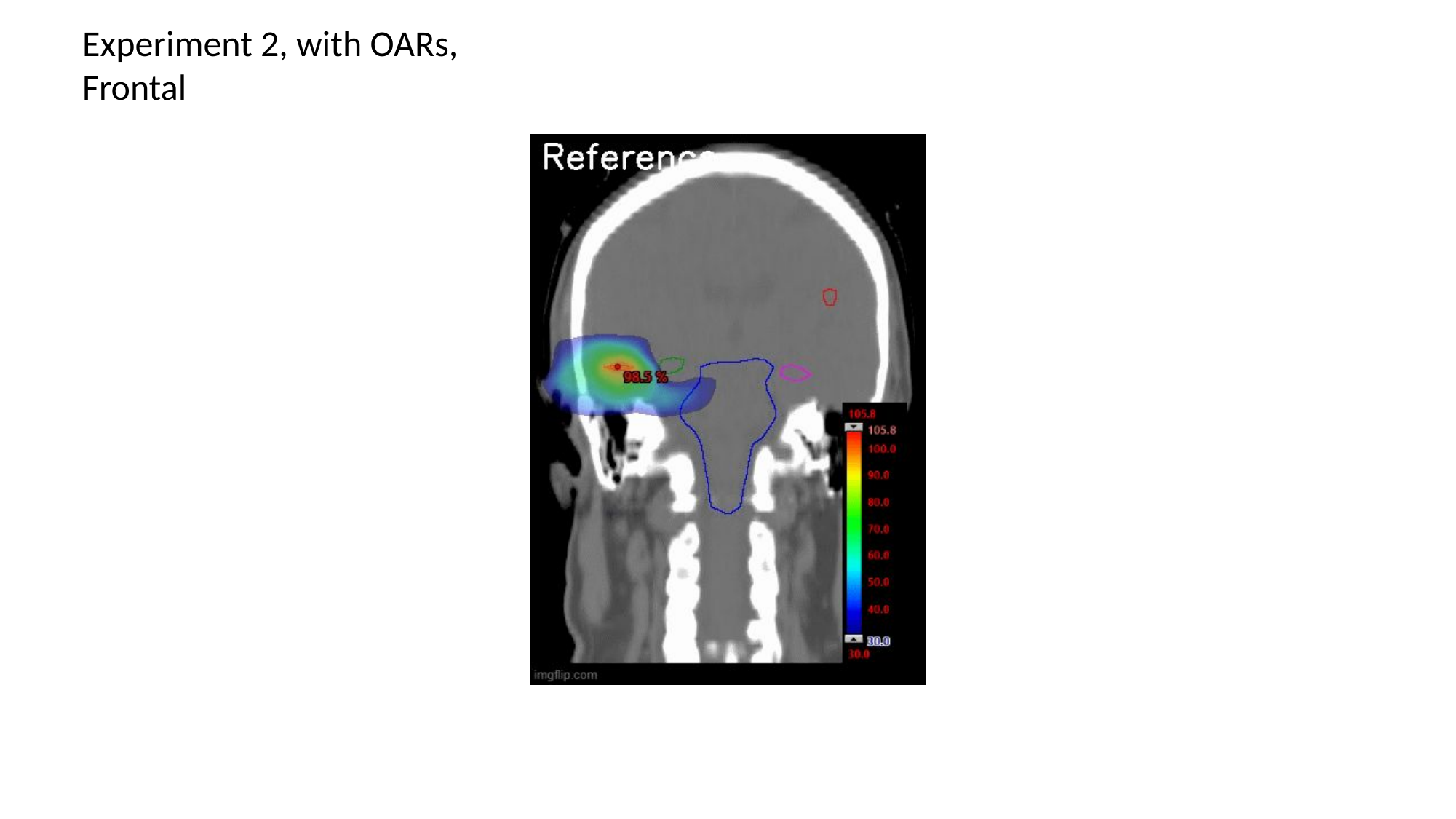

Experiment 2, with OARs, Frontal

## Slide 9
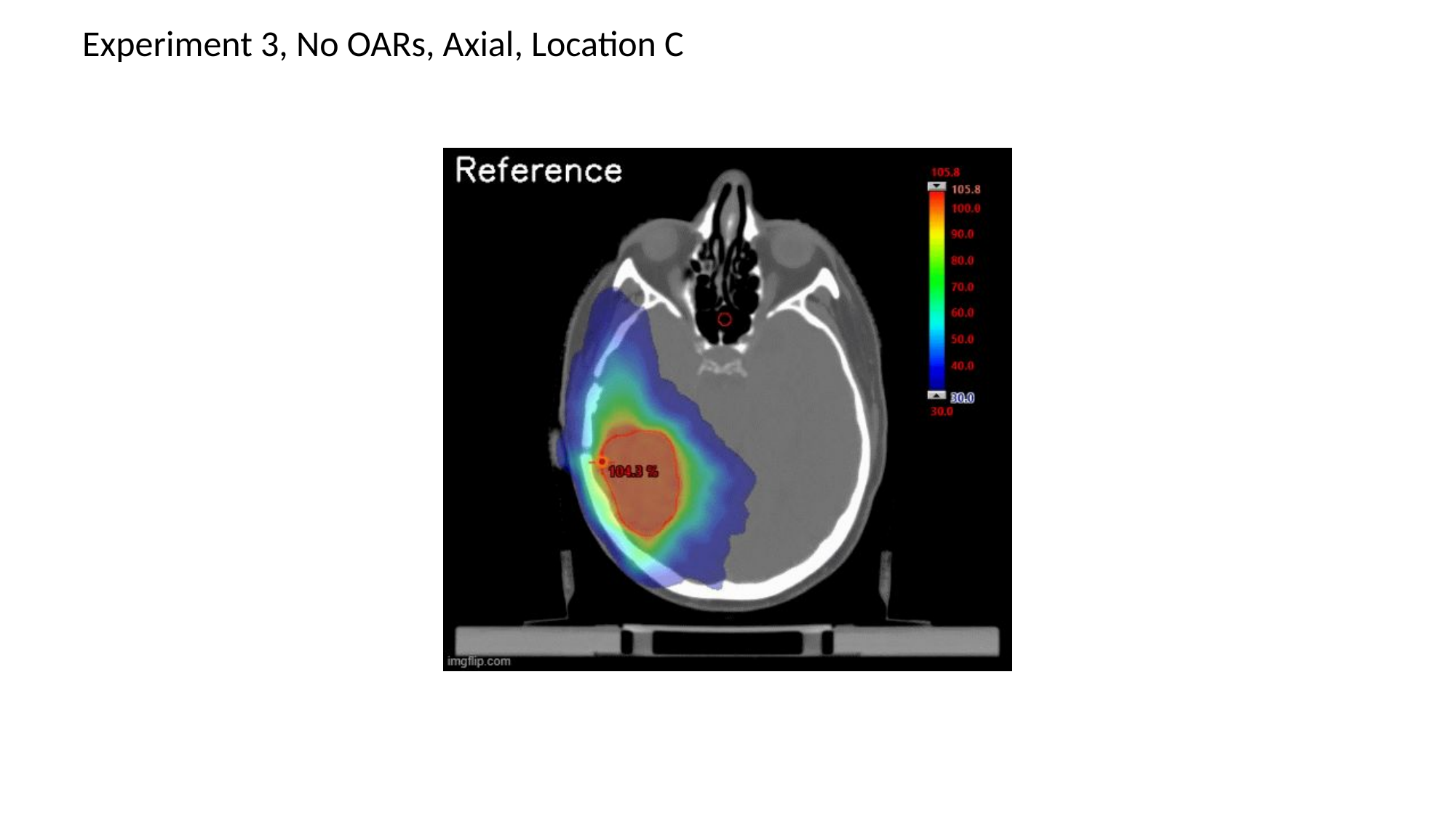

Experiment 3, No OARs, Axial, Location C

## Slide 10
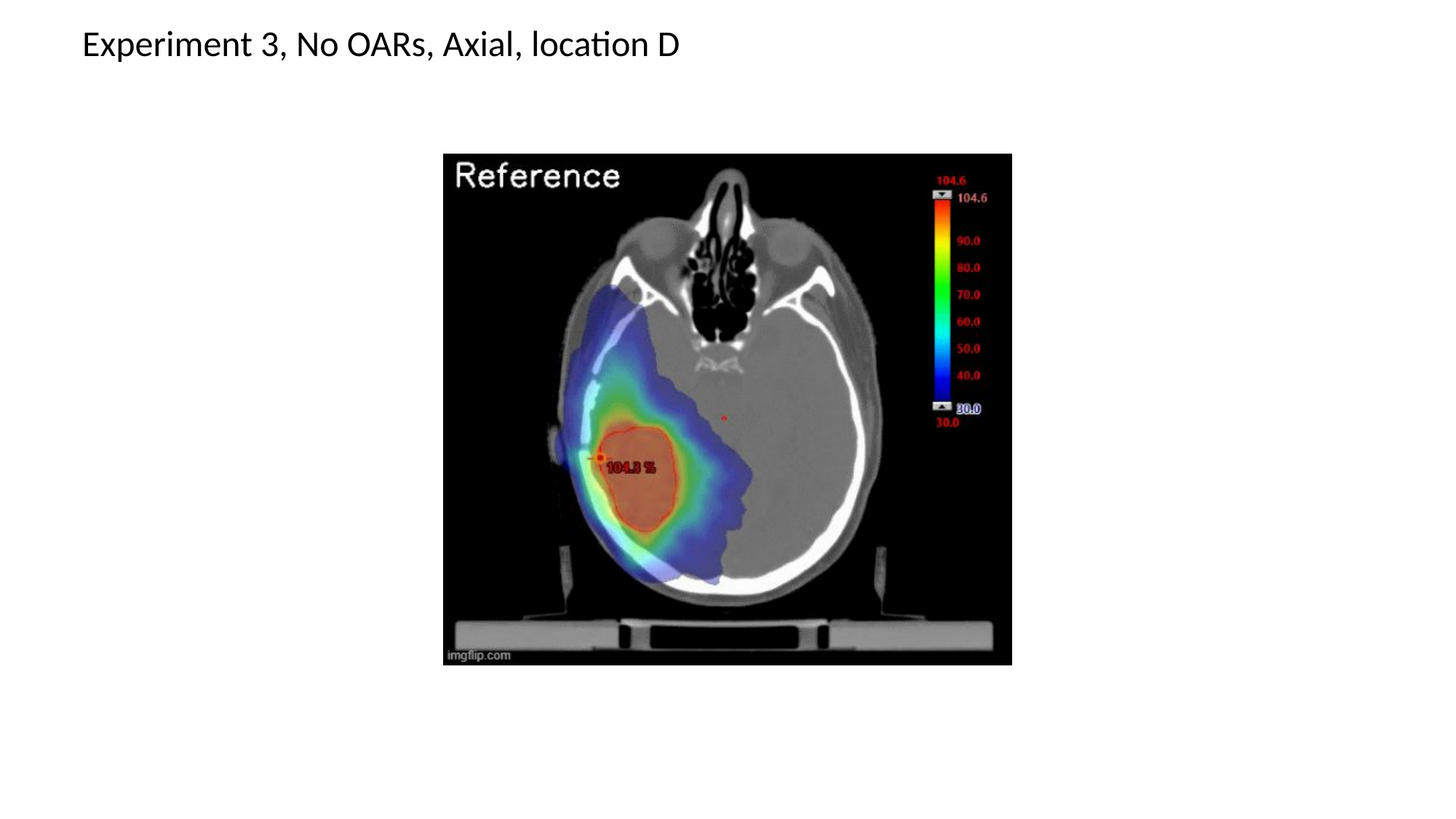

Experiment 3, No OARs, Axial, location D

## Slide 11
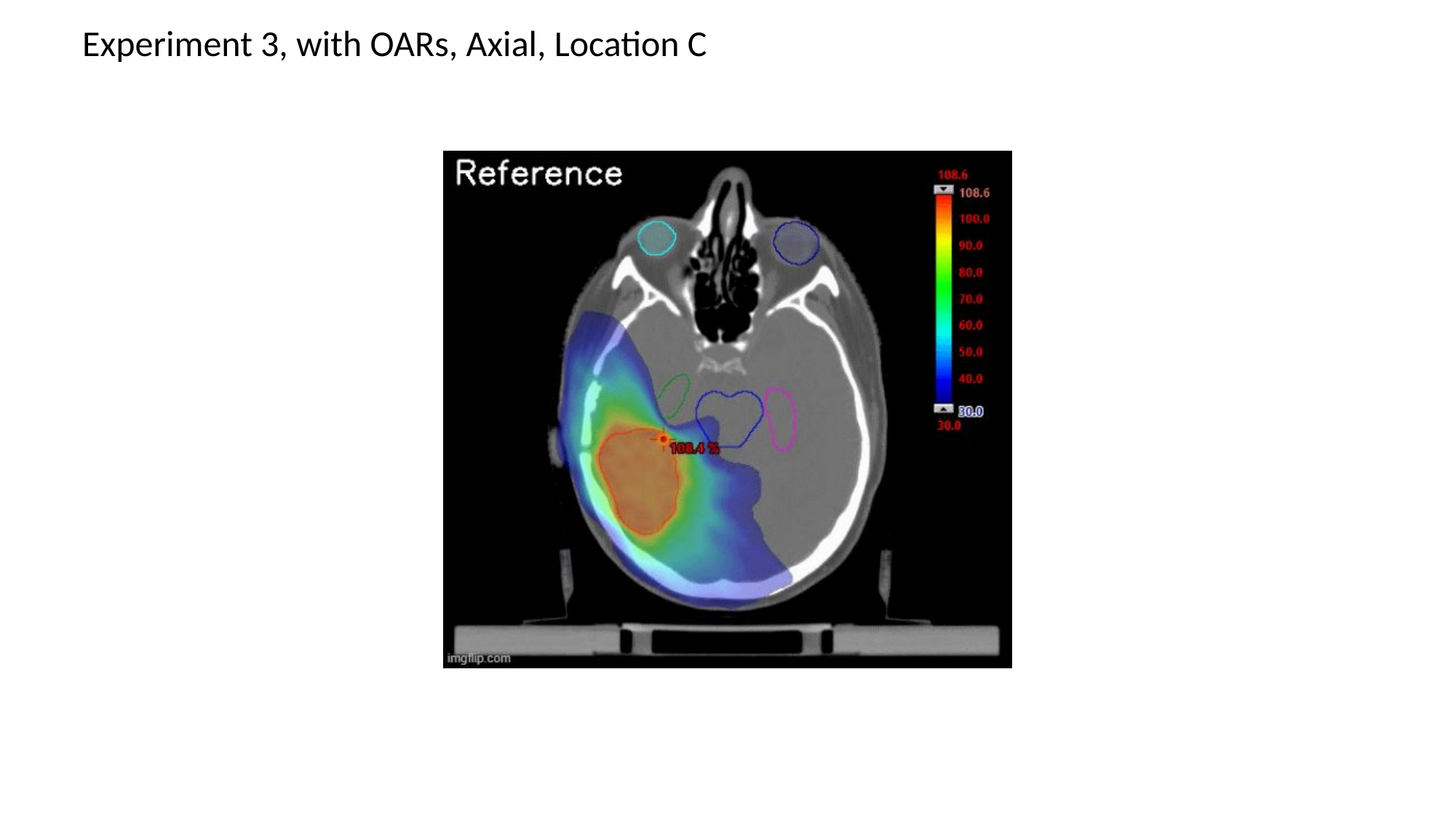

Experiment 3, with OARs, Axial, Location C

## Slide 12
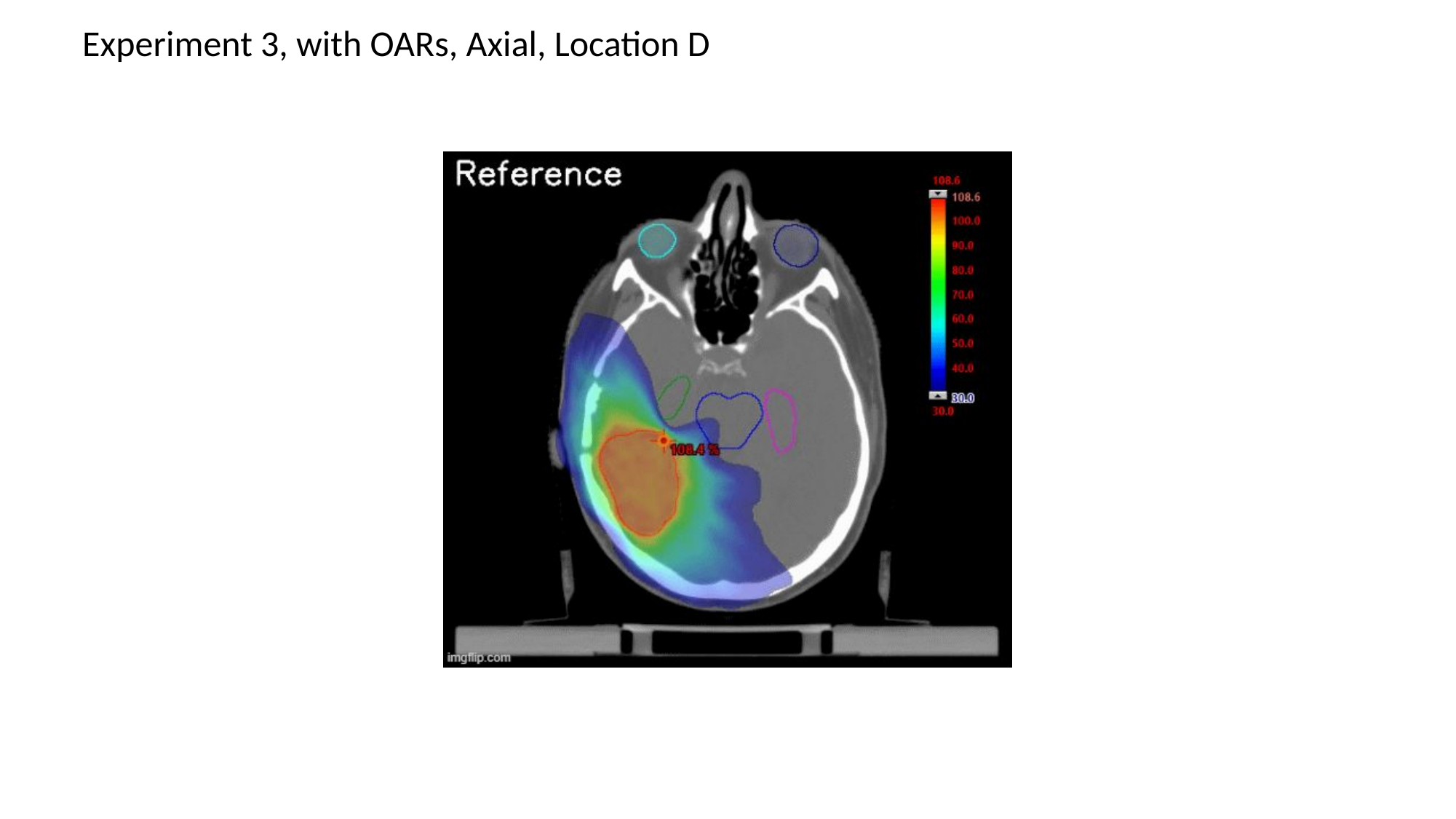

Experiment 3, with OARs, Axial, Location D

## Slide 13
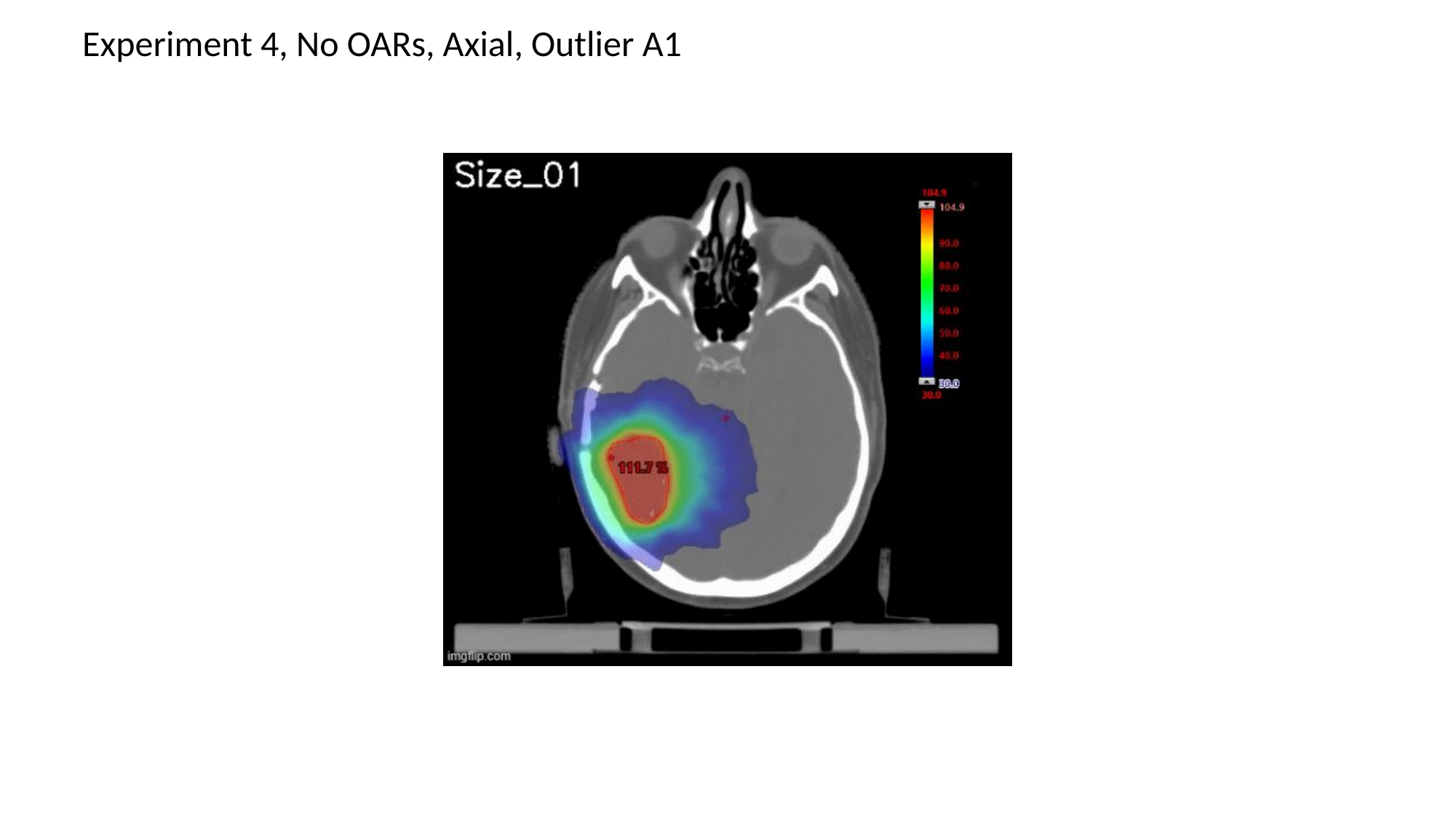

Experiment 4, No OARs, Axial, Outlier A1

## Slide 14
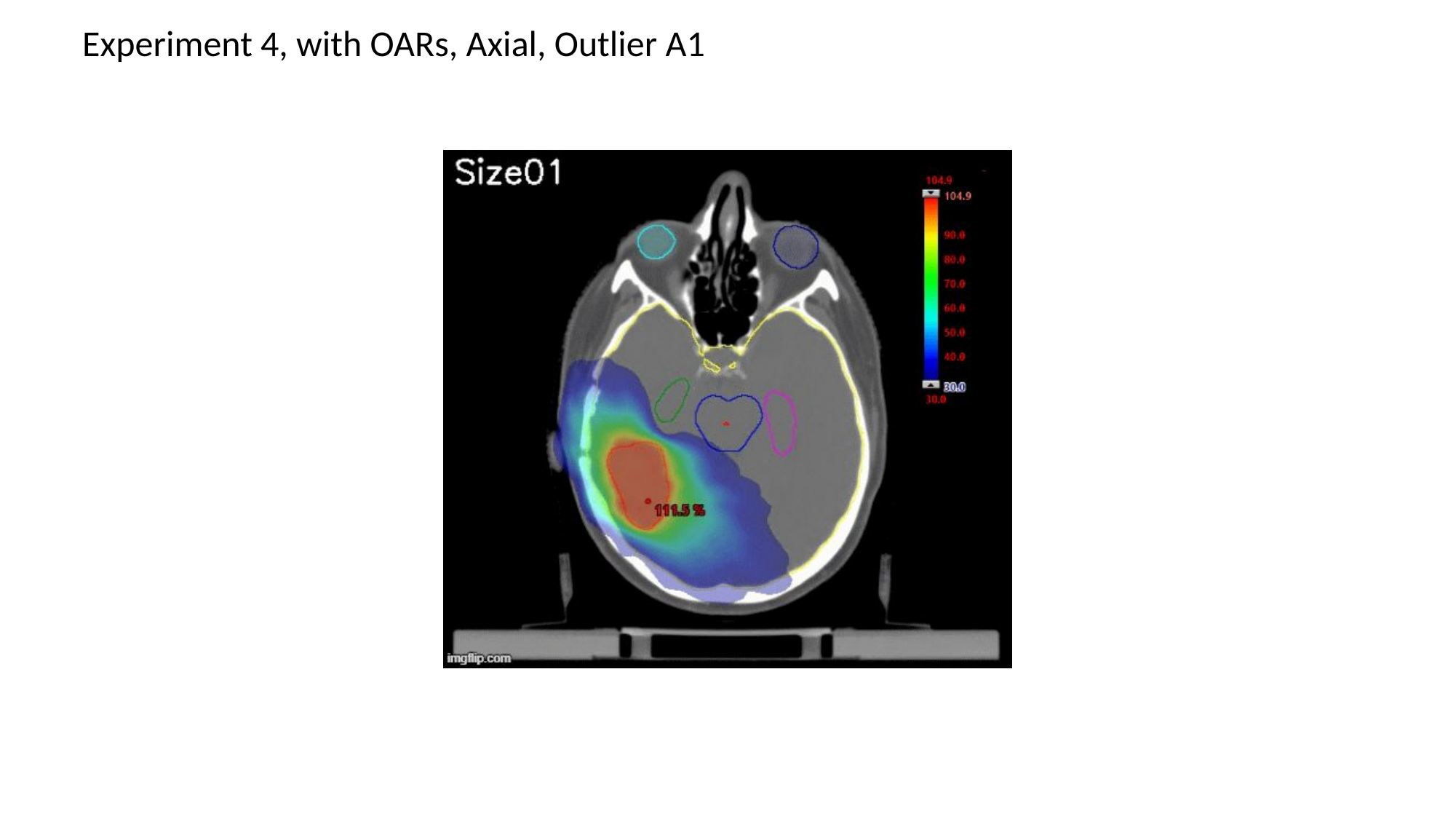

Experiment 4, with OARs, Axial, Outlier A1

## Slide 15
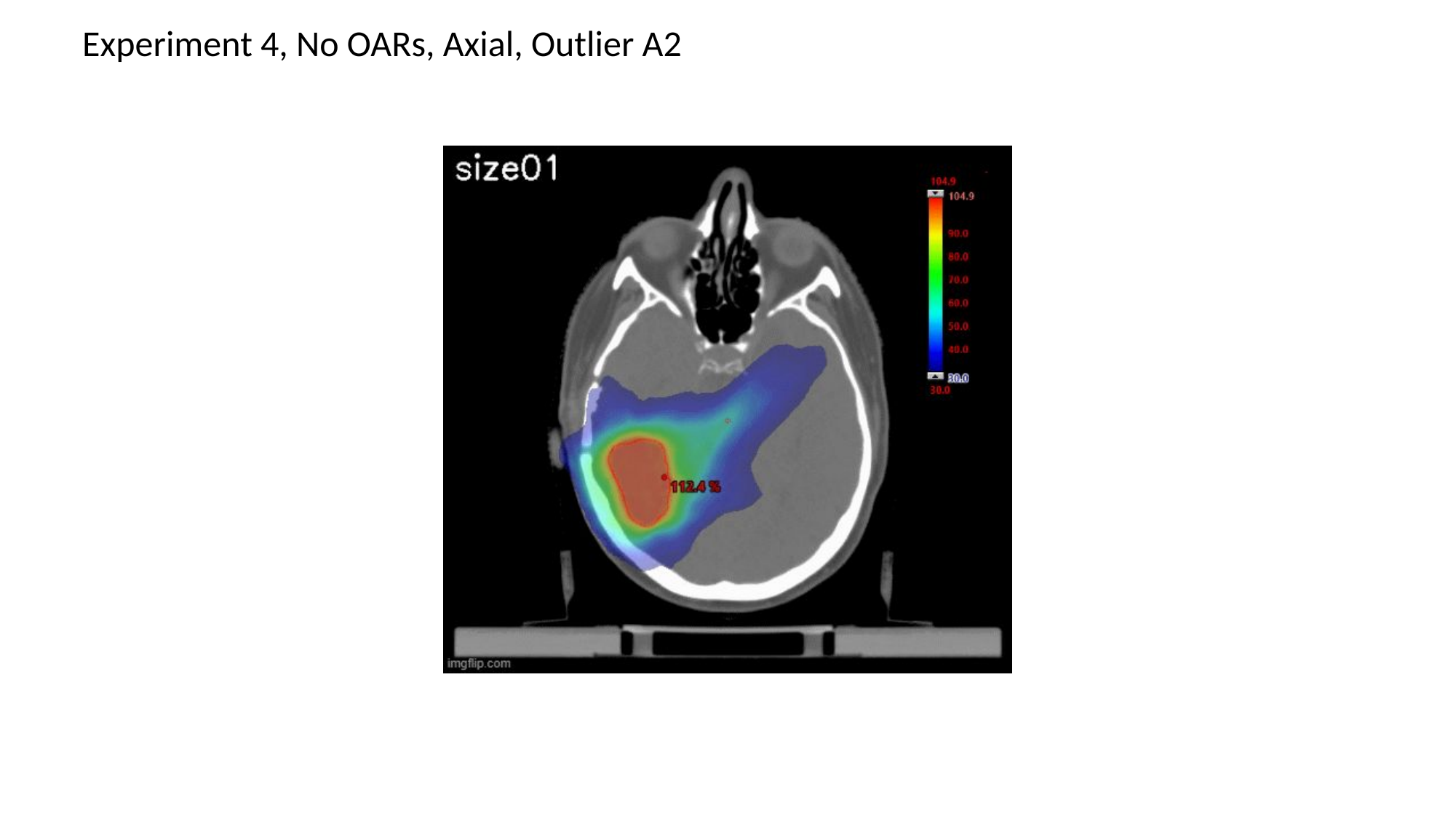

Experiment 4, No OARs, Axial, Outlier A2

## Slide 16
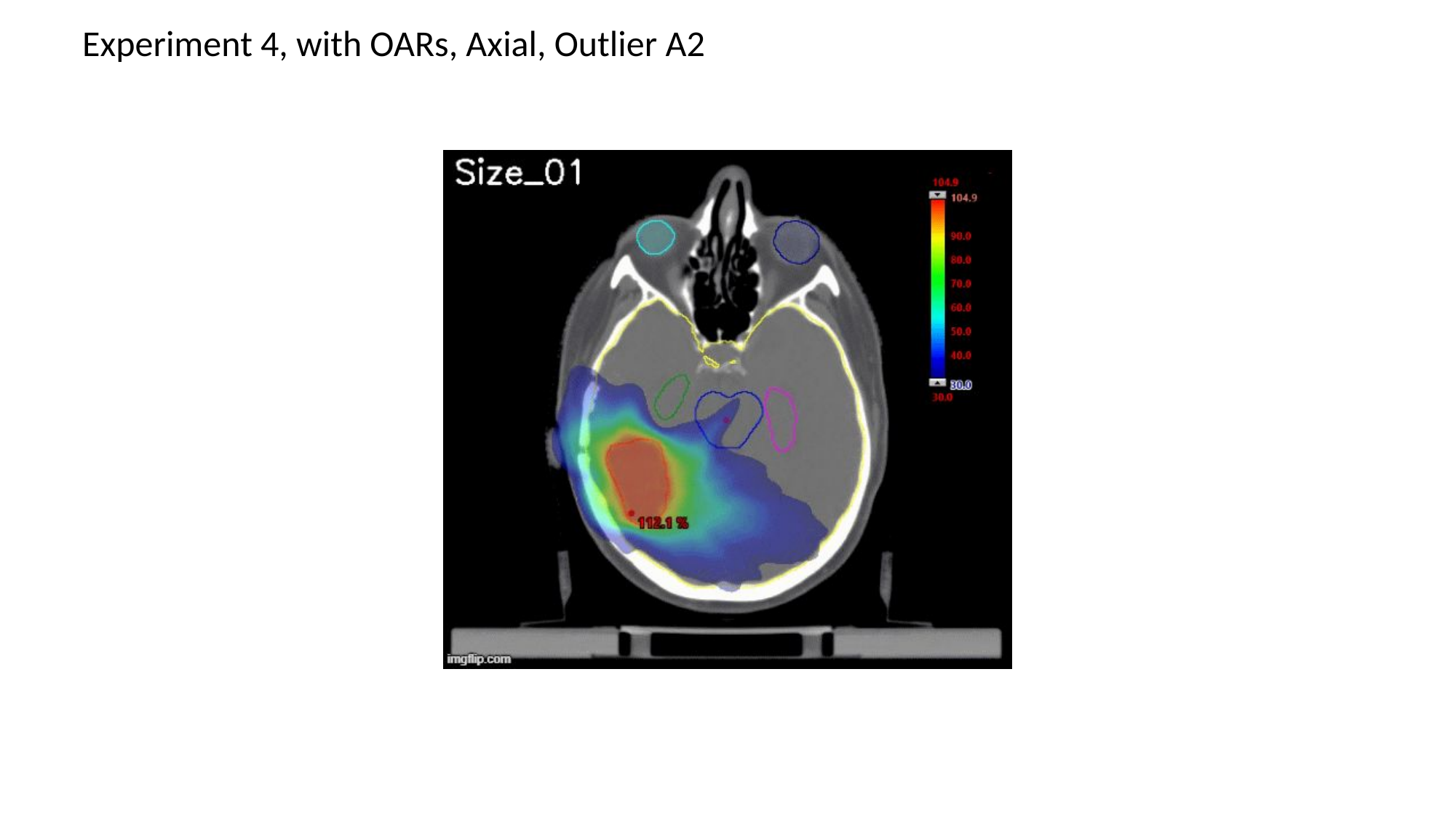

Experiment 4, with OARs, Axial, Outlier A2
